# Aryl radical-mediated N-heterocyclic carbene catalysis

**DOI:** 10.1038/s41467-021-24144-2

**Published:** 2021-06-22

**Authors:** Yuki Matsuki, Nagisa Ohnishi, Yuki Kakeno, Shunsuke Takemoto, Takuya Ishii, Kazunori Nagao, Hirohisa Ohmiya

**Affiliations:** 1 Division of Pharmaceutical Sciences, Graduate School of Medical Sciences, Kanazawa University, Kakuma-machi, Kanazawa, Japan; 2 JST, PRESTO, Kawaguchi, Saitama Japan

**Keywords:** Catalytic mechanisms, Organocatalysis, Synthetic chemistry methodology

## Abstract

There have been significant advancements in radical reactions using organocatalysts in modern organic synthesis. Recently, NHC-catalyzed radical reactions initiated by single electron transfer processes have been actively studied. However, the reported examples have been limited to catalysis mediated by alkyl radicals. In this article, the NHC organocatalysis mediated by aryl radicals has been achieved. The enolate form of the Breslow intermediate derived from an aldehyde and thiazolium-type NHC in the presence of a base undergoes single electron transfer to an aryl iodide, providing an aryl radical. The catalytically generated aryl radical could be exploited as an arylating reagent for radical relay-type arylacylation of styrenes and as a hydrogen atom abstraction reagent for α-amino C(sp^3^)–H acylation of secondary amides.

## Abstract (web summary)

Use of aryl halides as coupling precursors typically occurs through transition metal catalysis and/or photoredox chemistry, which requires some combination of light, metals, and oxidants or reductants. Here, the authors show a method to generate aryl radicals from halides using only an NHC organocatalyst.

## Introduction

**Fig. 1:**
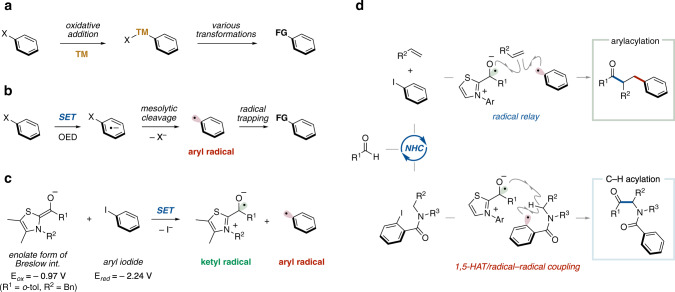
Activation of aryl halides and aryl radical-mediated NHC catalysis. **a** Oxidative addition of aryl halide to transition metal. TM, transition metal. **b** Organic electron donor induced aryl radical generation (recent advance). SET, single electron transfer. OED, organic electron donor. **c** Generation of aryl radical through NHC catalysis (working hypothesis). **d** Arylacylation of alkene and C(sp^3^)–H acylation through NHC catalysis (this work). 1,5-HAT, 1,5-hydrogen atom transfer.

Aryl halides (Ar–X) have been broadly utilized as versatile reagents for chemical reactions due to their availability and bench stability. Specifically, oxidative addition of the C(sp^2^)–X bond in an aryl halide to a transition-metal complex enables the generation of an arylmetal species that induces various transformations (Fig. 1a)^1,2^. The generation of an aryl radical^3,4^ through single electron reduction of an aryl halide followed by mesolytic cleavage of the C(sp^2^)–X bond has emerged as an alternative approach for the utilization of aryl halides as chemical reagents (Fig. 1b). Due to the high reduction potential of aryl halides, metal sources such as samarium salts^5^, transition-metal complexes^6^ and metal-based visible light photoredox catalysts^7^ have been mainly utilized as single electron donors. Recently, organic electron donors^8–11^ including visible light organic photoredox catalysts^12–14^ have taken the place of metals to provide simple and rapid access to aryl radicals via single electron transfer (SET). These approaches enable the generation of aryl radicals without using metal sources, but still require either a stoichiometric amount of the organic electron donor or light irradiation.

There has been significant advancement in the use of radical reactions catalyzed by N-heterocyclic carbenes (NHC) with SET from a Breslow intermediate, based on enzymatic pyruvate transformations^15–18^. Recently, we developed NHC-catalyzed decarboxylative radical cross-coupling between aldehydes and aliphatic carboxylic acid derived-redox active esters^19,20^. The reaction involves SET from the enolate form of the Breslow intermediate derived from an aldehyde and NHC in the presence of a base to the redox active ester, followed by radical–radical coupling between the resultant Breslow intermediate-derived radical and alkyl radical. Thus, the enolate form of the Breslow intermediate can serve as a single electron donor and an acyl radical equivalent. Since this report, a series of NHC-catalyzed radical cross-coupling reactions have been developed by us and other groups^21–33^. However, these reported examples have been limited to catalysis mediated by C(sp^3^)-centered radicals. To expand the scope of radical NHC catalysis, we questioned whether an aryl radical could be generated and utilized (Fig. 1c). Considering the gap in redox potential between the enolate form of the Breslow intermediate (*E*_ox_ = −0.97 V vs. SCE)^34^ and iodobenzene (*E*_red_ = −2.24 V vs. SCE)^35^, the corresponding electron transfer seems to be thermodynamically unfavored. However, two kinetic features, (1) a small reorganization energy of the enolate form of the Breslow intermediate^34^ and (2) a fast mesolytic cleavage of the radical anion derived from aryl iodide^35^, encouraged us to pursue this research program.

Here, we report an aryl radical-mediated NHC organocatalysis method. The aryl radical that is generated catalytically through thermodynamically unfavored SET from the enolate form of the Breslow intermediate to an aryl iodide could be exploited as an arylating reagent for radical relay-type arylacylation of styrenes (Fig. 1d, top) and as a hydrogen atom abstraction reagent for α-amino C(sp^3^)–H acylation of secondary amides (Fig. 1d, bottom). It should be noted that this protocol eliminates the requirement for metals, oxidants/reductants and light.

## Results and discussion

### Development of the reaction and screening of conditions

To test the generation of an aryl radical by the radical NHC catalysis, we designed intramolecular arylacylation reactions with benzaldehyde (**1a**) and aryl electrophiles **2a**–**d** bearing a cinnamyl tether group that can trap the aryl radical immediately (Table 1)^36^. Based on our previous studies on the NHC-catalyzed decarboxylative radical cross-coupling between aldehydes and redox-active esters^19^, we used a thiazolium salt (**N1**)^37^ possessing a N-2,6-diisopropylphenyl substituent and a seven-membered backbone structure as the NHC precursor and Cs_2_CO_3_ as the base. The reaction with aryl iodide **2a** as a substrate proceeded to afford **3aa** in 39% isolated yield with recovery of **2a** (entry 1). The reactions with other azolium salts resulted in no product formation (data not shown). Neither bromide nor chloride leaving groups resulted in product formation (entries 2 and 3). The reaction using the corresponding aryldiazonium tetrafluoroborate **2d** did not afford the desired coupling product **3aa** (entry 4). This might be due to the low reduction potential of the aryldiazonium salt (*E*_red_ = −0.2 V vs. SCE), which would cause a second electron transfer from the Breslow-derived radical intermediate^38^.

**Table 1:** Screening of reaction conditions.


Entry	Change from standard conditions	Yield (%) of 3aa
1	X = I (**2a**)	39
2	X = Br (**2b**)	0
3	X = Cl (**2c**)	0
4	X = N_2_BF_4_ (**2d**)	0

Reaction was carried out with **1a** (0.3 mmol), **2** (0.2 mmol), **N1** (10 mol %), Cs_2_CO_3_ (0.22 mmol) in DMSO (0.4 mL) at 60 °C for 12 h.

### Radical relay-type arylacylation of styrenes

**Fig. 2:**
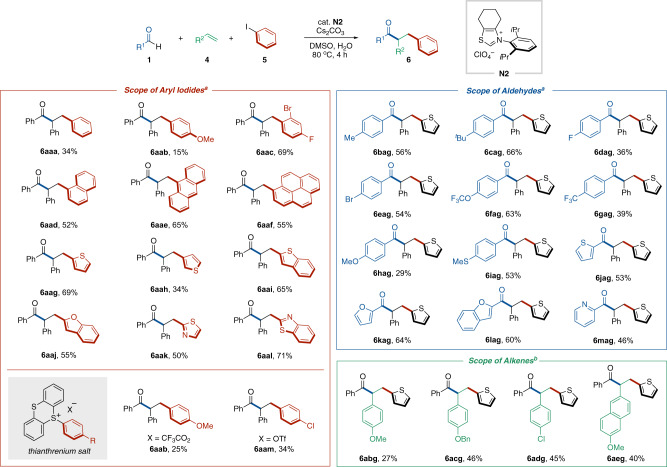
Substrate scope of radical relay-type arylacylation of styrenes. **a** Reaction was carried out with **1** (0.2 mmol), **4** (2 mmol), **5** (0.3 mmol), **N2** (30 mol %), Cs_2_CO_3_ (0.24 mmol), and H_2_O (0.2 mmol) in DMSO (0.4 mL) at 80 °C for 4 h. **b** Reaction was carried out with **1** (0.1 mmol), **4** (1 mmol), **5** (0.15 mmol), **N2** (30 mol %), Cs_2_CO_3_ (0.12 mmol), and H_2_O (0.1 mmol) in DMSO (0.2 mL) at 80 °C for 4 h.

**Fig. 3:**
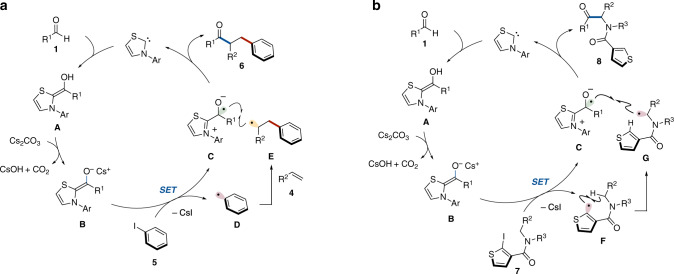
Possible pathways. **a** Catalytic cycle for arylacylation of alkene. **b** Catalytic cycle for C(sp^3^)–H acylation.

The successful example of an NHC-catalyzed reaction involving the generation of an aryl radical from aryl iodide and subsequent intramolecular radical addition to an alkene prompted us to explore intermolecular arylacylation using aryl iodides, alkenes, and aldehydes through a radical relay mechanism (see Table 1, entry 1). Though slight modifications of the reaction conditions, e.g., addition of water, use of *N*-2,6-diisopropylphenyl-substituted six-membered ring fused thiazolium-type NHC catalyst and increase of the amount of NHC catalyst, were required, the desired intermolecular arylacylation was achieved (Supplementary Fig. 1). Specifically, the reaction using aldehydes **1**, styrenes **4** and aryl iodides **5** occurred in the presence of a catalytic amount of thiazolium salt **N2** as the NHC precursor and stoichiometric amounts of Cs_2_CO_3_ and water in DMSO solvent at 80 °C to afford the three-component coupling products, α,β-diarylated ketones **6** (Fig. 2). The crude materials consisted of the product **6**, unreacted substrates and trace amounts of unidentified compounds. Two-component coupling stemming from **1** and **5** was not observed. The NHC-catalyzed radical relay process would involve SET from the enolate form of the Breslow intermediate to aryl iodide **5**, radical addition of the resulting aryl radical to styrene **4**, and subsequent radical–radical coupling between the Breslow intermediate-derived ketyl radical and the secondary benzylic radical (Fig. 3a)^20^. The role of H_2_O is unclear. It might increase the solubility of Cs_2_CO_3_ and facilitate the formation of the Breslow intermediate from aldehyde by acceleration of proton transfer-involved step.

As described above, the product derived from the two-component coupling between the aryl radical and the Breslow intermediate-derived ketyl radical in this system was not observed. Even under the reaction conditions without alkenes, the two-component coupling product was not formed. The dominant of the radical addition to styrene might be due to the high reactivity of aryl radical. Additionally, competitive reaction processes, such as the C–H abstraction from formyl C–H bond of aldehyde and the radical addition to another arenes or an enolate form of Breslow intermediate would be much faster than the two-component coupling. These unproductive processes would induce the decomposition of the ketyl radical, which would explain the requirement of high catalyst loading on the reaction conditions.

With the optimal reaction conditions in hand, the scope of each reaction component was evaluated and the results are summarized in Fig. 2. Various aryl iodides served as the arylating reagent in the reaction using benzaldehyde (**1a**) and styrene (**4a**) (Table 2, left). The reaction with iodobenzene or *p*-iodoanisole resulted in the desired product formation albeit with low reaction efficiency (**6aaa** and **6aab**). When an aryl substrate possessing fluorine, bromine, and iodine substituents was subjected to the optimal reaction conditions, selective mesolytic cleavage of the C(sp^2^)–I bond occurred to afford the multicomponent coupling product (**6aac**). Notably, this reaction allowed the use of π-extended molecules, such as 1-iodonaphthalene, 9-iodoanthracene and 1-iodopyrene (**6aad**–**6aaf**). 2-Iodothiophene exhibited higher reactivity than 3-iodothiophene (**6aag** vs **6aah**). Benzothiophene and benzofuran were tolerated in this reaction (**6aai** and **6aaj**). Electron-deficient thiazole-derived iodoheteroarenes participated in this protocol (**6aak** and **6aal**). The heteroarene-based aryl iodides including pyridines and quinolines also worked as substrates to afford the desired products in low yields (data not shown). Recently, arylthianthrenium salts have been treated as aryl radical precursors by visible-light photoredox catalysis^39^. This NHC organocatalysis could also generate aryl radicals from the thianthrenium salts and undergo the arylacylation reaction (**6aab** and **6aam**).

**Table 2:** Effects and properties of directing groups.

Amide substrate				
yield (%) of **8**^a^	74	24	27	42
E_pa_ (V vs. SCE)	−1.52	−1.99	−1.61	−2.19
BDE of C(sp^2^)–I (kcal/mol)	62.3	60.4	64.2	58.8
ΔG^‡^_C–H_ (kcal/mol)	4.2	7.5	6.1	7.2

^a^Reaction was carried out with **1a** (0.4 mmol), **7** (0.2 mmol), **N1** (20 mol %), and Cs_2_CO_3_ (0.24 mmol) in DMSO (0.4 mL) at 80 °C for 6 h.

A broad array of aromatic aldehydes was amenable to the reaction with 2-iodothiophene (**5g**) and styrene (**4a**) (Fig. 2, right). Alkyl and halogen substituents at the *para* position on the aromatic ring of the aldehydes did not inhibit the reaction (**6bag**–**6eag**). Both electron-withdrawing and electron-donating functional groups on the aromatic aldehydes were tolerated (**6fag**–**6iag**). Various heteroaromatic rings were incorporated into the acyl unit (**6jag**–**6mag**). Aliphatic aldehydes did not participate in the reaction even though a thiazolilum catalyst^22^ possessing an *N*-neopentyl group and a seven-membered backbone structure was used instead of **N1** (data not shown). This might be due to the slower formation of Breslow intermediate of aliphatic aldehydes using a neopentyl-derived NHC than that of aromatic aldehydes using **N1**^22^. A C–H abstraction from the formyl C–H bond of aliphatic aldehyde would be also considered.

Next, the scope of alkenes was explored (Fig. 2, right). Though inferior to non-substituted styrene, *p*-methoxy-, ben zyloxy-, and chloro-substituted styrenes provided the corresponding ketone products in moderate yields (**6abg**–**6adg**). A naphthalene-conjugated alkene was also a good substrate (**6aeg**). On the other hand, when aliphatic alkenes were subjected to the reaction conditions, three-component coupling was not observed (data not shown).

### C(sp^3^)–H acylation of secondary amides

**Fig. 4:**
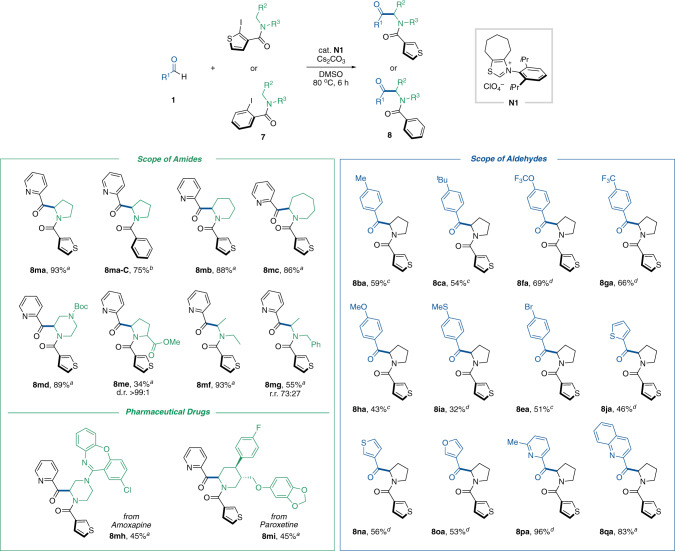
Substrate scope of C(sp^3^)–H acylation of secondary amides. **a** Reaction was carried out with **1** (0.4 mmol), **2** (0.2 mmol), **N1** (10 mol %), and Cs_2_CO_3_ (0.22 mmol) in DMSO (0.4 mL) at 80 °C for 6 h. **b** Reaction was carried out with **1** (0.4 mmol), **2** (0.2 mmol), **N1** (5 mol %), and Cs_2_CO_3_ (0.21 mmol) in DMSO (0.4 mL) at 80 °C for 6 h. **c** Reaction was carried out with **1** (0.6 mmol), **2** (0.2 mmol), **N1** (20 mol %), and Cs_2_CO_3_ (0.24 mmol) in DMSO (0.4 mL) at 80 °C for 6 h. **d** Reaction was carried out with **1** (0.4 mmol), **2** (0.2 mmol), **N1** (20 mol %), and Cs_2_CO_3_ (0.24 mmol) in DMSO (0.4 mL) at 80 °C for 6 h.

We turned our attention to exploiting the catalytically generated aryl radical as a hydrogen atom abstraction reagent^40,41^. During the survey of the scope of aryl iodides in the arylacylation of alkenes shown in Fig. 2, 2-iodothiophene was found to have better reactivity than other aryl iodides. This result inspired us to incorporate the 2-iodothiophene moiety into the directing group that serves as the intramolecular hydrogen atom abstraction reagent for the NHC-catalyzed C(sp^3^)–H acylation using aldehydes. Specifically, the reaction between 2-pyridine carboxaldehyde (**1** **m**) and secondary amide (**7a**) having a 2-iodothiophene moiety occurred in the presence of a catalytic amount of the *N*-2,6-diisopropylphenyl-substituted seven-membered ring fused thiazolium salt **N1**^37^ as the NHC precursor and Cs_2_CO_3_ in DMSO at 80 °C to afford the corresponding acylation product **8ma** in 93% isolated yield (Fig. 4). In this NHC-catalyzed process, the resultant α-amino C(sp^3^)-centered radical, derived by SET from the enolate form of the Breslow intermediate to the 2-iodothiophene moiety in the substrate and subsequent 1,5-hydrogen atom transfer, could participate in radical–radical coupling with the Breslow intermediate-derived ketyl radical (see Fig. 1d, bottom and Fig. 3b). **N2** possessing a six-membered ring backbone exhibited comparable reactivity (Supplementary Fig. 2). The use of 2-iodobenzene-based directing group also afforded the corresponding acylation product **8ma-C** (vide infra for mechanistic considerations for C(sp^3^)–H acylation of secondary amides). The directing group could be removed by Schwartz’s reagent (Supplementary Fig. 9).

The scope of amides in this protocol was investigated with the use of 2-pyridine carboxaldehyde (**1m**) as a coupling partner (Fig. 4, left). The reactions with cyclic amides derived from pyrrolidine, piperidine and azepane proceeded efficiently to afford the desired α-acylated products in high yields (**8ma**–**8mc**). Although a piperazine derivative was also a suitable substrate (**8md**), a morpholine scaffold was not tolerated (data not shown). The acyl group could be incorporated into amino acids such as proline with high diastereoselectivity (**8me**). In addition to cyclic amides, acyclic amides underwent this organocatalyzed acylation (**8mf** and **8mg**). To demonstrate the power and broad functional group tolerance of this protocol, the C(sp^3^)–H acylation of pharmaceutical drugs was conducted. Secondary amine-based drugs such as Amoxapine and Paroxetine resulted in product formation without being disturbed by coexistent functional groups (**8mh** and **8mi**). In our previous report on the NHC-catalyzed decarboxylative alkylation^19^, α-aminoalkyl radical generated from α-amino acid-derived redox active ester can couple with a Breslow intermediate-derived radical to afford the α-aminoketone. Importantly, however, secondary amide substrates presented here would be more abundant and structurally diverse than α-amino acids. Thus, this protocol allowed to couple α-amino alkyl radicals derived from various amides such as cyclic amides, acyclic amides and pharmaceutical drugs.

Subsequently, we explored the scope of aldehydes with a pyrrolidine substrate **7a** (Fig. 4, right). The reactions of *p* tolualdehyde or *p*-*tertiary*-butyl benzaldehyde gave the corresponding α-aminoketones in moderate yields (**8ba** and **8ca**). A variety of functional groups including trifluoromethoxy, trifluoromethyl, methoxy, and thioether were tolerated well (**8fa**–**8ia**). The aryl bromide group survived the reductive reaction conditions (**8ea**). Ketones having electron-rich heteroaryl substituents such as thiophene or furan were also constructed (**8ja**, **8na**, and **8oa**). Similar to 2-pyridinecarboxaldehyde, the reactions with 6-methyl-2-pyridinecarboxyaldehyde and 2-quinolinecaboxaldehyde gave the corresponding products in exceptionally high yields (**8pa** and **8qa**).

### Mechanistic considerations for C(sp^3^)–H acylation of secondary amides

To understand how the 2-iodothiophene-based directing group imparted the high reactivity, we evaluated four different directing groups from two viewpoints, the efficiencies of aryl radical generation and hydrogen abstraction (Table 2). Screening of directing groups such as 2-iodothiophene (**7a**), 3-iodothiophene (**7a**-**A**), 2-iodofuran (**7a**-**B**), and 2-iodobenzene (**7a**-**C**) was conducted for the reaction of **7** with benzaldehyde (**1a**) in the presence of **N1** catalyst and Cs_2_CO_3_ in DMSO at 80 °C (Table 2, 1st line). As expected, 2-iodothiophene (**7a**) was the most effective among those examined. Next, we examined the reduction potential of amide substrates and their C(sp^2^)–I bond dissociation energies (BDEs). Cyclic voltammetry experiments revealed that **7a** having a 2-iodothiophene moiety was the most reducible (Table 2, 2nd line and Supplementary Figs. 3–6). The BDE values obtained by DFT calculations indicated that these C(sp^2^)–I bonds tended to be easily cleaved and that **7a**-**C** would undergo more facile mesolytic cleavage of the C(sp^2^)–I bond after single electron reduction compared with the other substrates (Table 2, 3rd line, and Supplementary Fig. 7). This provided a good explanation for the improved yield for the acylation reaction of **7a**-**C** than **7a**-**A** and **7a**-**B** in spite of the higher reduction potential (**7a**-**C**: –2.19 V vs. **7a**-**A**: –1.99 V and **7a**-**B**: –1.61 V). Furthermore, DFT calculations of the activation energy of the α-amino C(sp^3^)–H abstraction step were carried out (Table 2, 4th line and Supplementary Fig. 8). 2-Iodothiophene derivative (**7a**) showed the lowest activation energy for the C(sp^3^)–H abstraction. Based on these results, we assumed that the 2-iodothiophene-based directing group might have an advantage in terms of the facile generation of the aryl radical by the thermodynamically less unfavored SET and the subsequent fast C(sp^3^)–H abstraction.

To see whether or not the intermediacy of electron donor-acceptor (EDA) complex facilitated the thermodynamically unfavored SET, we conducted UV/Vis absorption studies using known precursor of Breslow intermediate and iodobenzene (Supplementary Fig. 10). As a result, the significant shift of the absorption was not observed. Moreover, the reaction under light irradiation conditions to facilitate the SET process did not give the desired product at all (data not shown). These results didn’t support the intermediacy of the EDA complex.

In summary, this achievement expanded the scope of radical NHC catalysis, enabling the generation and utilization of aryl radicals^42^. The enolate form of the Breslow intermediate derived from an aldehyde and a thiazolium-type NHC in the presence of a base undergoes SET to the aryl iodide, producing an aryl radical. Although the SET event is thermodynamically unfavored, the small reorganization energy of the enolate form of the Breslow intermediate and the fast mesolytic cleavage of the C(sp^2^)–I bond makes the pathway kinetically feasible. The catalytically generated aryl radical could undergo addition to styrenes or intramolecular hydrogen atom abstraction to form a C(sp^3^)-centered radical, which engaged in the subsequent radical–radical coupling with the Breslow intermediate-derived ketyl radical. The NHC catalysis presented here provides a strategy for an aryl radical-mediated organocatalytic reaction. Studies for expanding this strategy toward the synthesis of complex molecules are currently underway in our laboratory.

## Methods

### The reaction to produce 6aag in Fig. 2 is representative

Thiazolium salt **N2** (23.9 mg, 0.06 mmol) was placed in a Schlenk tube containing a magnetic stirring bar. The tube was sealed with a Teflon^®^-coated silicon rubber septum, and then evacuated and filled with nitrogen. Degassed DMSO (400 μL) and H_2_O (3.6 μL, 0.2 mmol) were added to the tube. Next, benzaldehyde (**1a**) (20.3 μL, 0.2 mmol), styrene (**4a**) (229 μL, 2.0 mmol) and 2-iodothiophene (**5** **g**) (38.2 μL, 0.3 mmol) were added, followed by Cs_2_CO_3_ (78.2 mg, 0.24 mmol). After 4 h stirring at 80 °C, the reaction mixture was treated with saturated NH_4_Cl aqueous solution (400 μL), then extracted with diethyl ether (four times) and dried over sodium sulfate. After filtration, the resulting solution was evaporated under reduced pressure. After the volatiles were removed under reduced pressure, flash column chromatography on silica gel (100:0–90:10, hexane/EtOAc) gave **6aag** (40.3 mg, 0.14 mmol) in 69% yield.

### The reaction to produce 8ma in Fig. 4 is representative

Thiazolium salt **N1** (8.3 mg, 0.02 mmol) and amide **7a** (61.4 mg, 0.2 mmol) were placed in a Schlenk tube containing a magnetic stirring bar. The tube was sealed with a Teflon^®^-coated silicon rubber septum, and then evacuated and filled with nitrogen. Cs_2_CO_3_ (71.7 mg, 0.22 mmol), degassed DMSO (400 μL) and 2-pyridinecarboxaldehyde (**1m**) (42.8 mg, 0.4 mmol) were added to the vial. After 6 h stirring at 80 °C, the reaction mixture was treated with saturated NH_4_Cl aqueous solution (400 μL), then extracted with diethyl ether (4 times) and dried over sodium sulfate. After filtration through a short plug of aluminum oxide (1 g) with diethyl ether as an eluent, the resulting solution was evaporated under reduced pressure. After the volatiles were removed under reduced pressure, flash column chromatography on silica gel (100:0–50:50, hexane/EtOAc) gave **8ma** (53.0 mg, 0.19 mmol) in 93% yield.

## Supplementary information

Supplementary Information

## Data Availability

The authors declare that the data supporting the findings of this study are available within the paper or its Supplementary Information files and from the corresponding author upon reasonable request.

## References

[1] Stille JK, Lau KSY (1977). Mechanisms of oxidative addition of organic halides to Group 8 transition-metal complexes. Acc. Chem. Res..

[2] Miyaura N, Suzuki A (1955). Palladium-catalyzed cross-coupling reactions of organoboron compounds. Chem. Rev..

[3] Galli C (1988). Radical reactions of arenediazonium ions: an easy entry into the chemistry of the aryl radical. Chem. Rev..

[4] Ghosh I, Marzo L, Das A, Shaikh R, König B (2016). Visible light mediated photoredox catalytic arylation reactions. Acc. Chem. Res..

[5] Krief A, Laval A-M (1999). Coupling of organic halides with carbonyl compounds promoted by SmI_2_, the Kagan reagent. Chem. Rev..

[6] Tsou TT, Kochi JK (1979). Mechanism of oxidative addition. Reaction of nickel(0) complexes with aromatic halides. J. Am. Chem. Soc..

[7] Nguyen JD, D’Amato EM, Narayanam JMR, Stephenson CRJ (2012). Engaging unactivated alkyl, alkenyl and aryl iodides in visible-light-mediated free radical reactions. Nat. Chem..

[8] Broggi J, Terme T, Vanelle P (2014). Organic electron donors as powerful single‐electron reducing agents in organic synthesis. Angew. Chem., Int. Ed..

[9] Smith AJ, Poole DL, Murphy JA (2019). The role of organic electron donors in the initiation of BHAS base-induced coupling reactions between haloarenes and arenes. Sci. China. Chem..

[10] Li M (2017). Transition-metal-free radical C(sp^3^)–C(sp^2^) and C(sp^3^)–C(sp^3^) coupling enabled by 2-Azaallyls as super-electron-donors and coupling-partners. J. Am. Chem. Soc..

[11] Liu B, Lim C-H, Miyake GM (2017). Visible-light-promoted C−S cross-coupling via intermolecular charge transfer. J. Am. Chem. Soc..

[12] Ghosh I, Ghosh T, Bardagi JI, König B (2014). Reduction of aryl halides by consecutive visible light-induced electron transfer processes. Science.

[13] Discekici EH (2015). Read de Alaniz, J. A highly reducing metal-free photoredox catalyst: design and application in radical dehalogenations. Chem. Commun..

[14] Kim H, Kim H, Lambert TH, Lin S (2020). Reductive electrophotocatalysis: merging electricity and light to achieve extreme reduction potentials. J. Am. Chem. Soc..

[15] Ishii T, Nagao K, Ohmiya H (2020). Recent advances in radical N-heterocyclic carbene catalysis. Chem. Sci..

[16] Song R, Chi YR (2019). N-heterocyclic carbene catalyzed radical coupling of aldehydes with redox-active esters. Angew. Chem., Int. Ed..

[17] Ohmiya H (2020). N-Heterocyclic carbene-based catalysis enabling cross-coupling reactions. ACS Catal..

[18] Guin J, De Sarkar S, Grimme S, Studer A (2008). Biomimetic carbene-catalyzed oxidations of aldehydes using TEMPO. Angew. Chem., Int. Ed..

[19] Ishii T, Kakeno Y, Nagao K, Ohmiya H (2019). N-heterocyclic carbene-catalyzed decarboxylative alkylation of aldehydes. J. Am. Chem. Soc..

[20] Ishii T, Ota K, Nagao K, Ohmiya H (2019). N-Heterocyclic carbene-catalyzed radical relay enabling vicinal alkylacylation of alkenes. J. Am. Chem. Soc..

[21] Ota K, Nagao K, Ohmiya H (2020). N-Heterocyclic carbene-catalyzed radical relay enabling synthesis of δ-ketocarbonyls. Org. Lett..

[22] Kakeno Y, Kusakabe M, Nagao K, Ohmiya H (2020). Direct synthesis of dialkyl ketones from aliphatic aldehydes through radical N-heterocyclic carbene catalysis. ACS Catal..

[23] Li J-L (2020). Radical acylfluoroalkylation of olefins through N‐heterocyclic carbene organocatalysis. Angew. Chem., Int. Ed..

[24] Kim I, Im H, Lee H, Hong S (2020). N-Heterocyclic carbene-catalyzed deaminative cross-coupling of aldehydes with Katritzky pyridinium salts. Chem. Sci..

[25] Dai L, Xia Z-H, Gao Y-Y, Gao Z-H, Ye S (2019). Visible-light-driven n-heterocyclic carbene catalyzed γ- and ε-alkylation with alkyl radicals. Angew. Chem., Int. Ed..

[26] Mavroskoufis A (2020). N‐Heterocyclic carbene catalyzed photoenolization/diels–alder reaction of acid fluorides. Angew. Chem., Int. Ed..

[27] Davies AV, Fitzpatrick KP, Betori RC, Scheidt KA (2020). Combined photoredox and carbene catalysis for the synthesis of ketones from carboxylic acids. Angew. Chem., Int. Ed..

[28] Meng QY, Doben N, Studer A (2020). Cooperative NHC and photoredox catalysis for the synthesis of β-trifluoromethylated alkyl aryl ketones. Angew. Chem., Int. Ed..

[29] Liu M-S, Shu W (2020). Catalytic, metal-free amide synthesis from aldehydes and imines enabled by a dual-catalyzed umpolung strategy under redox-neutral conditions. ACS Catal..

[30] Liu K, Studer A (2021). Direct α‑acylation of alkenes via n‑heterocyclic carbene, sulfinate, and photoredox cooperative triple catalysis. J. Am. Chem. Soc..

[31] Ren S-C (2021). Carbene-catalyzed alkylation of carboxylic esters via direct photoexcitation of acyl azolium intermediates. ACS Catal..

[32] Barik S, Biju AT (2020). N-Heterocyclic carbene (NHC) organocatalysis using aliphatic aldehydes. Chem. Commun..

[33] Dai L, Ye S (2021). Recent advances in *N*-heterocyclic carbene-catalyzed radical reactions. Chin. Chem. Lett..

[34] Nakanishi I, Itoh S, Fukuzumi S (1999). Electron-transfer properties of active aldehydes of thiamin coenzyme models, and mechanism of formation of the reactive intermediates. Chem. Eur. J..

[35] Pause L, Robert M, Savéant J-M (1999). Can single-electron transfer break an aromatic carbon−heteroatom bond in one step? A novel example of transition between stepwise and concerted mechanisms in the reduction of aromatic iodides. J. Am. Chem. Soc..

[36] Rueping M, Leiendecker M, Das A, Poissona T, Buia L (2011). Potassium tert-butoxide mediated Heck-type cyclization/isomerization–benzofurans from organocatalytic radical cross-coupling reactions. Chem. Commun..

[37] Piel, I., Pawelczyk, M. D., Hirano, K., Fröhlich, R., Glorius, F. A Family of thiazolium salt derived N‐heterocyclic carbenes (NHCs) for organocatalysis: synthesis, investigation and application in cross‐benzoin condensation. *Eur. J. Org. Chem*. 5475–5484 (2011).

[38] LeStrat F, Murphy JA, Hughes M (2002). Direct electroreductive preparation of indolines and indoles from diazonium salts. Org. Lett..

[39] Berger F (2019). Site-selective and versatile aromatic C−H functionalization by thianthrenation. Nature.

[40] Snieckus V, Cuevas JC, Sloan CP, Liu H, Curran DP (1990). Intramolecular α-amidoyl to Aryl 1,5-hydrogen atom transfer reactions. Heteroannulation and α-nitrogen functionalization by radical translocation. J. Am. Chem. Soc..

[41] Yoshikai N, Mieczkowski A, Matsumoto A, Ilies L, Nakamura E (2010). Iron-catalyzed C−C bond formation at α-position of aliphatic amines via C−H bond activation through 1,5-hydrogen transfer. J. Am. Chem. Soc..

[42] Liu, W., et al. Mesoionic carbene-Breslow intermediates as super electron donors: application to the metal-free arylacylation of alkenes. *Chem. Catal.* **1**, 10.1016/j.checat.2021.03.004 (2021).

