# Supplementary material for: Aryl radical-mediated N-heterocyclic carbene catalysis
Source: Nat Commun. 2021 Jun 22;12:3848. doi: 10.1038/s41467-021-24144-2 (PMC8219674; doi:10.1038/s41467-021-24144-2)
Supplement: Supplementary file 1 — Supplementary Information [file 41467_2021_24144_MOESM1_ESM.pdf]

Aryl Radical-Mediated N-Heterocyclic Carbene Catalysis

Yuki Matsuki,[1] Nagisa Ohnishi,[1] Yuki Kakeno,[1] Shunsuke Takemoto,[1] Takuya Ishii,[1] Kazunori Nagao,[1]* and Hirohisa Ohmiya[1,2]*

[1] Division of Pharmaceutical Sciences, Graduate School of Medical Sciences, Kanazawa University, Kakuma-machi, Kanazawa 920-1192, Japan
[2] JST, PRESTO, 4-1-8 Honcho, Kawaguchi, Saitama, 332-0012, Japan
These authors contributed equally: Yuki Matsuki, Nagisa Ohnishi
*E-mail: Kazunori Nagao : nkazunori@p.kanazawa-u.ac.jp
Hirohisa Ohmiya: ohmiya@p.kanazawa-u.ac.jp

Table of Contents

■ **Supplementary Methods** ■

1. Instrumentation and Chemicals S2
2. Characterization Data for Aryl Thianthrenium Salt S3
3. Procedure for Intramolecular Arylacylation of Alkenes S3
4. Characterization Data for Cyclized Product S4
5. Procedure for Intermolecular Arylacylation of Styrenes S4
6. Characterization Data for Three-Component Coupling Products S4–S14
7. Characterization Data for Amide Substrates S15–S18
8. Procedure for C(sp^3)–H Acylation of Secondary Amides S18
9. Characterization Data for α-Aminoketones S19–S31
10. Effects of Reaction Components on Intermolecular Arylacylation of Styrenes S32
11. Effects of Reaction Components on C–H Acylation S32
12. Cyclic Voltammetry (CV) Experiments S33–S34
13. Computational Studies
    - 13.1. Calculation of Bond Dissociaton Energy (BDE) S34–S40
    - 13.2. Calculation of Gibbs Energy Barrier on α-Amino C(sp^3)–H Abstraction Step ($\Delta G^{\ddagger}_{C\text{–}H}$) S40–S48
14. Deprotection of Directing Group S49–50
15. UV-Vis Absorption Measurement S51

■ **NMR Spectra** ■ S52–S203

■ **Supplementary References** ■ S204–S205

■ **Supplementary Methods** ■

1. Instrumentation and Chemicals

NMR spectra were recorded on a JNM-ECS400, operating at 400 MHz for ^{1}H NMR and 100.5 MHz for ^{13}C NMR, and JNM-ECA600, operating at 600 MHz for ^{1}H NMR and 150.9 MHz for ^{13}C NMR, and Bruker AVANCE NEO 400N spectrometer, operating at 400 MHz for ^{1}H NMR, 100.6 MHz for ^{13}C NMR. Chemical shifts are reported in δ ppm. DART-Mass and ESI-Mass spectra were measured with JMS-T100TD (JEOL Ltd.). TLC analyses were performed on commercial glass plates bearing 0.25-mm layer of Merck Silica gel $60F_{254}$. Silica gel (Wakosil® 60, 64~210 µm) was used for column chromatography. Biotage Selekt and LaboACE LC-5060 (for Gel Permeation Chromatography) were used for purification. IR spectra were measured with a Thermo Scientific iD7 ATR Accessory for the Thermo Scientific Nicolet iS5 FT-IR Spectrometer. Melting points were measured on a Stanford Research Systems MPA100.

All reactions were carried out under nitrogen atmosphere. Materials were obtained from commercial suppliers or prepared according to standard procedures unless otherwise noted. Cs_2CO_3 was purchased from FUJIFILM Wako Pure Chemical Co., stored under nitrogen, and used as received. DMSO was purchased from FUJIFILM Wako Pure Chemical Co., stored under nitrogen, and used as received. Thiazolium salt **N1** and **N2** were prepared by the reported procedure.[1] Aldehydes **1a**, **1e** and **1h** were purchased from Nacalai Tesque Inc., **1b**, **1d, 1p** and **1q** were purchased from FUJIFILM Wako Pure Chemical Co., **1c**, **1f**, **1g**, **1i**, **1j**, **1k**, **1l**, and **1m** were purchased from Tokyo Chemical Industry Co., **1n** and **1o** were purchased from Sigma-Aldrich Japan., stored under nitrogen, and used as received. Styrene **4a** was purchased from Tokyo Chemical Industry Co., Styrene derivatives **4b**–**4e** were prepared by Wittig reaction from the corresponding aldehydes. Aryl iodides **5a** and **5g** were purchased from FUJIFILM Wako Pure Chemical Co., **5b** was purchased from Sigma-Aldrich Japan., **5c** was purchased from Combi-Blocks., stored under nitrogen, and used as received. Aryl iodides **2a**, **5d**–**5f** and **5h**–**5l** were prepared by the reported procedure.[2–7] The aryl thianthrenium salts were prepared according to the reported procedure.[8] Amide substrates **7a**, **7b**, **7e**, **7h**, **7i** and **7a**-**A**–**C** were prepared from the corresponding secondary amines and carboxylic acids through condensation reactions with pivaloyl chloride.[9] **7c**, **7d**, **7f** and **7g** were prepared from the corresponding secondary amines and carboxylic acids through condensation reactions with pivaloyl chloride.[10]

2. Characterization Data for Aryl Thianthrenium Salt

5-(4-Methoxyphenyl)-5H-thianthren-5-ium 2,2,2-trifluoroacetate (5b-S)

Under an ambient atmosphere, a 20 ml two-necked round-bottom flask was charged sequentially with anisole (91.2 mg, 0.85 mmol), thianthrene-S-oxide (197 mg, 0.85 mmol), and dry MeCN (3.4 ml, 0.25 M). After all solids had dissolved, the solution was cooled to –78 °C, and trifluoroacetic anhydride (0.36 ml, 0.54 g, 2.55 mmol) was added in one portion at –78 °C. The mixture was allowed to warm to 0 °C over a period of 2 h. After stirring overnight, the solution was diluted with DCM (8.5 ml) and poured into a separatory funnel. The organic layer was washed with 85 ml water. The solvent was removed under reduced pressure, and the oily residue was purified by flash chromatography on silica gel (Biotage Selekt, 90:10, DCM/MeOH). The oily precipitate was dried in vacuo to afford the desired thianthrenium salt **5b-S** (219 mg, 59 %) as highly viscous and colorless oil.

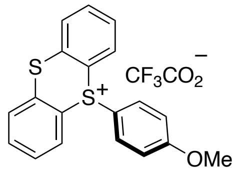

5b-S

Colorless oil. **IR** (neat) 705, 758, 796, 828, 1020, 1123, 1178, 1264 cm^{-1}. **^{1}H NMR** (400 MHz, $CDCl_3$) δ 3.80 (s, 3H), 6.94 (d, J = 9.2 Hz, 2H), 7.25 (d, J = 6.8 Hz, 2H), 7.73–7.83 (m, 6H), 8.53 (d, J = 7.8 Hz, 2H) **^{13}C NMR** (150.9 MHz, $CDCl_3$) δ 55.7, 113.6, 116.1 (q, $J_{C\text{–}F}$ = 290 Hz), 116.4, 119.2, 130.0, 130.2, 130.3, 134.2, 134.6, 136.0, 160.3 (q, $J_{C\text{–}F}$ = 27.2 Hz) 163.6. **HRMS–ESI** (*m/z*): [M–CF_3CO_2]$^+$ calcd for $C_{19}H_{15}OS_2$, 323.0559; found, 323.0553.

3. Procedure for Intramolecular Arylacylation of Alkenes

The reaction in Table 1, entry 1 is representative. Thiazolium salt **N1** (8.3 mg, 0.02 mmol) was placed in a schlenk tube containing a magnetic stirring bar. The vial was sealed with a Teflon®-coated silicon rubber septum, and then the vial was evacuated and filled with nitrogen. Degassed DMSO (400 µL) was added to the vial. Next, benzaldehyde **1a** (30.5 µL, 0.3 mmol) and 1-(cinnamyloxy)-2-iodobenzene **2a** (67.2 mg, 0.2 mmol) were added. Then, Cs_2CO_3 (71.7 mg, 0.22 mmol) was added. After 12 h stirring at 60 ˚C, the reaction mixture was treated with saturated NH_4Cl aqueous solution (400 µL), then extracted with diethylether (4 times) and dried over sodium sulfate. After filtration, the resulting solution was evaporated under reduced pressure. After volatiles were removed under reduced pressure, flash column chromatography on silica gel (100:0–90:10, hexane/EtOAc) gave **3aa** (24.5 mg, 0.08 mmol) in 39 % yield.

4. Characterization Data for Cyclized Product

2-(2,3-Dihydrobenzofuran-3-yl)-1,2-diphenylethan-1-one (3aa)

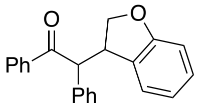

3aa

The product **3aa** was purified by flash chromatography on silica gel (Biotage Selekt, 100:0–90:10, hexane/EtOAc) (Table 1, entry 1, 24.5 mg, 0.08 mmol, 39% isolated yield). The ratio (1:1) of diasteromers was determined by ^{1}H-NMR analysis. White solid. **M.p.** 120–126 °C. **IR** (neat) 698, 749, 1232, 1449, 1460, 1480, 1677, 3028, 3059 cm^{-1}. **^{1}H NMR** (400 MHz, $CDCl_3$) δ 4.20–4.38 (m, 2H), 4.45–4.51 (m, 0.50 × 1H), 4.70 (d, J = 10.4 Hz, 0.50 × 1H), 4.78 (m, 1H), 5.86 (d, J = 7.2 Hz, 0.50 × 1H), 6.55 (t, J = 7.2 Hz, 0.50 × 1H), 6.73–6.80 (m, 0.50 × 1H + 1H), 7.03–7.45 (m, 0.50 × 1H + 9H), 7.89–7.96 (m, 2H). **^{13}C NMR** (100.6 MHz, $CDCl_3$) δ 44.7, 45.4, 58.0, 59.1, 68.0, 74.0, 109.4, 109.7, 119.7, 120.5, 125.5, 126.0, 126.3, 126.7, 127.7, 127.8, 127.8, 128.3, 128.6, 128.6, 128.7, 128.8, 128.9, 129.0, 129.1, 129.3, 133.1, 133.3, 136.3, 136.6, 136.7, 136.8, 160.2, 160.3, 199.0, 199.0. **HRMS–EI** (*m/z*): [M]$^+$ calcd for $C_{22}H_{18}O_2$, 314.1307; found, 314.1310.

5. Procedure for Intermolecular Arylacylation of Styrenes

The reaction to produce 6aag in Fig. 2 is representative. Thiazolium salt **N2** (23.9 mg, 0.06 mmol) was placed in a Schlenk tube containing a magnetic stirring bar. The tube was sealed with a Teflon®-coated silicon rubber septum, and then evacuated and filled with nitrogen. Degassed DMSO (400 μL) and H_2O (3.6 μL, 0.2 mmol) were added to the tube. Next, benzaldehyde (**1a**) (20.3 μL, 0.2 mmol), styrene (**4a**) (229 μL, 2.0 mmol) and 2-iodothiophene (**5g**) (38.2 μL, 0.3 mmol) were added, followed by Cs_2CO_3 (78.2 mg, 0.24 mmol). After 4 h stirring at 80 °C, the reaction mixture was treated with saturated NH_4Cl aqueous solution (400 μL), then extracted with diethyl ether (4 times) and dried over sodium sulfate. After filtration, the resulting solution was evaporated under reduced pressure. After the volatiles were removed under reduced pressure, flash column chromatography on silica gel (100:0–90:10, hexane/EtOAc) gave **6aag** (40.3 mg, 0.14 mmol) in 69% yield.

6. Characterization Data for Three-Component Coupling Products

1,2,3-Triphenylpropan-1-one (6aaa)

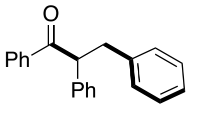

6aaa

The product **6aaa** was purified by flash chromatography on silica gel (Biotage Selekt, 100:0–90:10, hexane/EtOAc) (Fig. 2, 19.5 mg, 0.07 mmol, 34% isolated yield). White solid. The spectrum data of **6aaa** was consistent with the literature.[11]

3-(4-Methoxyphenyl)-1,2-diphenylpropan-1-one (6aab)

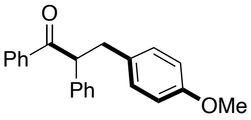

6aab

The product **6aab** was purified by flash chromatography on silica gel (Biotage Selekt, 100:0–80:20, hexane/EtOAc) (Fig. 2, with aryl iodide **5b**, 9.5 mg, 0.03 mmol, 15% isolated yield; with aryl thianthrenium salt **5b-S**, 15.8 mg, 0.05 mmol, 25% isolated yield;). The spectrum data of **6aab** was consistent with the literature.[11]

3-(2-Bromo-4-fluorophenyl)-1,2-diphenylpropan-1-one (6aac)

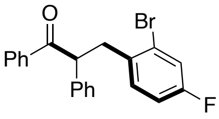

6aac

The product **6aac** was purified by flash chromatography on silica gel (Biotage Selekt, 100:0–90:10, hexane/EtOAc) (Fig. 2, 52.7 mg, 0.14 mmol, 69% isolated yield). Pale yellow oil. **IR** (neat) 569, 694, 754, 858, 1030, 1176, 1227, 1446, 1484, 1679 cm^{-1}. **^{1}H NMR** (600 MHz, $CDCl_3$) δ 3.13 (dd, J = 13.2, 7.2 Hz, 1H), 3.62 (dd, J = 13.2, 7.2 Hz, 1H), 4.98 (t, J = 7.2 Hz, 1H), 6.79 (m, 1H), 6.98 (t, J = 7.2 Hz, 1H), 7.20 (m, 1H), 7.23–7.27 (m, 5H), 7.34 (t, J = 7.8 Hz, 1H), 7.45 (t, J = 7.2 Hz, 1H), 7.89 (d, J = 7.8 Hz, 2H). **^{13}C NMR** (150.9 MHz, $CDCl_3$) δ 39.5, 53.2, 114.2 (d, J = 20.1 Hz), 119.7 (d, $J_{C–F}$ = 24.6 Hz), 124.2 (d, $J_{C–F}$ = 8.7 Hz), 127.3, 128.1, 128.5, 128.7, 129.0, 132.8 (d, $J_{C–F}$ = 7.2 Hz), 133.0, 134.6 (d, $J_{C–F}$ = 2.9 Hz), 136.5, 138.7, 161.0 (d, $J_{C–F}$ = 248.6 Hz), 198.8. **^{19}F NMR** (376.5 MHz, $CDCl_3$) δ –114.5. **HRMS–DART** (*m/z*): $[M+ NH_4]^+$ calcd for $C_{21}H_{20}BrFNO$, 400.0707; found, 400.0702.

3-(Naphthalen-1-yl)-1,2-diphenylpropan-1-one (6aad)

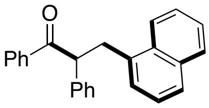

6aad

The product **6aad** was purified by flash chromatography on silica gel (Biotage Selekt, 100:0–90:10, hexane/EtOAc) (Fig. 2, 35.0 mg, 0.10 mmol, 52% isolated yield). White solid. The spectrum data of **6aad** was consistent with the literature.[11]

3-(Anthracen-9-yl)-1,2-diphenylpropan-1-one (6aae)

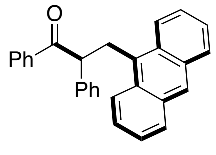

6aae

The product **6aae** was purified by flash chromatography on silica gel (Biotage Selekt, 100:0–90:10, hexane/EtOAc) (Fig. 2, 50.2 mg, 0.13 mmol, 65% isolated yield). Pale yellow solid. **M.p.** 156–162 °C. **IR** (neat) 699, 732, 756, 1205, 1342, 1446, 1597, 1678 cm^{-1}. **^{1}H NMR** (400 MHz, $CDCl_3$) δ 4.09 (dd, J = 14.4, 6.4 Hz, 1H), 4.59 (dd, J = 14.4, 6.4 Hz, 1H), 5.02 (t, J = 6.4 Hz, 1H), 7.03–7.10 (m, 5H), 7.22–7.26 (m, 2H), 7.33–7.41 (m, 5H), 7.78 (d, J = 7.2 Hz, 2H), 7.95 (d, J = 9.2 Hz, 2H), 8.03 (d, J = 8.4 Hz, 2H), 8.32 (s, 1H). **^{13}C NMR** (100.5 MHz, $CDCl_3$) δ 31.5, 55.4, 124.5, 124.7, 125.4, 126.4, 127.1, 128.2, 128.4, 128.7, 128.8, 129.0, 130.1, 131.4, 132.1, 132.8, 136.5, 139.2, 199.7. **HRMS–DART** (*m/z*): $[M+ NH_4]^+$ calcd for $C_{29}H_{26}NO$, 404.2009; found, 404.2011.

1,2-Diphenyl-3-(pyren-1-yl)propan-1-one (6aaf)

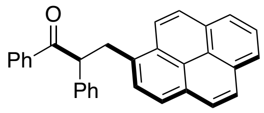

6aaf

The product **6aaf** was purified by flash chromatography on silica gel (Biotage Selekt, 100:0–90:10, hexane/EtOAc) (Fig. 2, 45.1 mg, 0.11 mmol, 55% isolated yield). Pale yellow solid. **M.p.** 146–152 °C. **IR** (neat) 683, 697, 720, 754, 843, 1182, 1447, 1597, 1678 cm^{-1}. **^{1}H NMR** (600 MHz, $CDCl_3$) δ 3.78 (dd, J = 13.8, 6.6 Hz, 1H), 4.33 (dd, J = 13.8, 6.6 Hz, 1H), 5.09 (t, J = 6.6 Hz, 1H), 7.18–7.28 (m, 7H), 7.37 (t, J = 7.2 Hz, 1H), 7.65 (d, J = 7.8 Hz, 1H), 7.85 (d, J = 6.6 Hz, 2H), 7.94–8.00 (m, 4H), 8.09 (d, J = 9.0 Hz, 1H), 8.15 (t, J = 7.8 Hz, 2H), 8.25 (d, J = 9.0 Hz, 1H). **^{13}C NMR** (150.9 MHz, $CDCl_3$) δ 37.4, 55.7, 123.1, 124.6, 124.8 125.0, 125.8, 126.7, 127.2, 127.5, 128.2, 128.4, 128.7, 128.8, 129.0, 130.0, 130.8, 131.3, 132.8, 133.8, 136.6, 139.2, 199.3. **HRMS–DART** (*m/z*): $[M+ NH_4]^+$ calcd for $C_{31}H_{26}NO$, 428.2009; found, 428.2006.

1,2-Diphenyl-3-(thiophen-2-yl)propan-1-one (6aag)

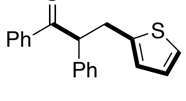

6aag

The product **6aag** was purified by flash chromatography on silica gel (Biotage Selekt, 100:0–90:10, hexane/EtOAc) (Fig. 2, 40.3 mg, 0.14 mmol, 69% isolated yield). Pale yellow solid. The spectrum data of **6aag** was consistent with the literature.[11]

1,2-Diphenyl-3-(thiophen-3-yl)propan-1-one (6aah)

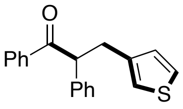

6aah

The product **6aah** was purified by flash chromatography on silica gel (Biotage Selekt, 100:0–90:10, hexane/EtOAc) (Fig. 2, 19.9 mg, 0.07 mmol, 34% isolated yield). White solid. **M.p.** 114–117 °C. **IR** (neat) 699, 758, 776, 1251, 1447, 1492, 1597, 1679 cm^{-1}. **^{1}H NMR** (600 MHz, $CDCl_3$) δ 3.09 (dd, J = 14.4, 7.2 Hz, 1H), 3.59 (dd, J = 14.4, 7.2 Hz, 1H), 4.80 (t, J = 7.2 Hz, 1H), 6.81–6.83 (m, 2H), 7.16 (dd, J = 4.8, 3.0 Hz, 1H), 7.20 (m, 1H), 7.26–7.29 (m, 4H), 7.36 (t, J = 7.8 Hz, 2H), 7.46 (t, J = 7.2 Hz, 1H), 7.91 (d, J = 8.4 Hz, 2H). **^{13}C NMR** (150.9 MHz, $CDCl_3$) δ 34.5, 55.2, 121.6, 125.1, 127.2, 128.2, 128.5, 128.5, 128.7, 128.9, 132.9, 136.7, 139.1, 140.0, 199.2. **HRMS–DART** (*m/z*): [M+ NH_4]$^+$ calcd for $C_{19}H_{20}NOS$, 310.1260; found, 310.1259.

3-(Benzo[b]thiophen-2-yl)-1,2-diphenylpropan-1-one (6aai)

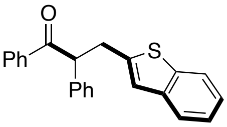

6aai

The product **6aai** was purified by flash chromatography on silica gel (Biotage Selekt, 100:0–90:10, hexane/EtOAc) (Fig. 2, 44.5 mg, 0.13 mmol, 65% isolated yield). Pale yellow solid. **M.p.** 124–128 °C. **IR** (neat) 698, 726, 747, 1176, 1244, 1435, 1447, 1597, 1678 cm^{-1}. **^{1}H NMR** (400 MHz, $CDCl_3$) δ 3.34 (dd, J = 14.4, 6.8 Hz, 1H), 3.89 (dd, J = 14.4, 6.8 Hz, 1H), 4.95 (t, J = 6.8 Hz, 1H), 6.92 (s, 1H), 7.20–7.38 (m, 9H), 7.46 (t, J = 7.2 Hz, 1H), 7.59 (d, J = 6.8 Hz, 1H), 7.70 (d, J = 7.6 Hz, 1H), 7.95 (d, J = 7.6 Hz, 2H). **^{13}C NMR** (150.9 MHz, $CDCl_3$) δ 35.1, 55.5, 122.0, 122.3, 122.9, 123.6, 124.0, 127.4, 128.2, 128.5, 128.8, 129.1, 133.0, 136.4, 138.5, 139.5, 139.9, 143.2, 198.5. **HRMS–DART** (*m/z*): [M+ NH_4]$^+$ calcd for $C_{23}H_{22}NOS$, 360.1417; found, 360.1411.

3-(Benzofuran-2-yl)-1,2-diphenylpropan-1-one (6aaj)

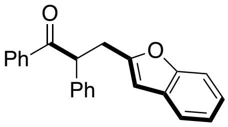

6aaj

The product **6aaj** was purified by flash chromatography on silica gel (Biotage Selekt, 100:0–90:10, hexane/EtOAc) (Fig. 2, 35.9 mg, 0.11 mmol, 55% isolated yield). Yellow solid. **M.p.** 64–69 °C. **IR** (neat) 696, 751, 948, 1178, 1252, 1454, 1597, 1681 cm^{-1}. **^{1}H NMR** (400 MHz, $CDCl_3$) δ 3.22 (dd, J = 15.2, 7.2 Hz, 1H), 3.73 (dd, J = 15.2, 7.2 Hz, 1H), 5.12 (t, J = 7.2 Hz, 1H), 6.27 (s, 1H), 7.11–7.20 (m, 3H), 7.24–7.30 (m, 4H), 7.34–7.40 (m, 4H), 7.45 (t, J = 7.2 Hz, 1H), 7.96 (d, J = 6.8 Hz, 2H). **^{13}C NMR** (100.5 MHz, $CDCl_3$) δ 32.9, 52.1, 103.7, 110.7, 120.4, 122.4, 123.3, 127.4, 128.0, 128.5, 128.7, 128.8, 129.0, 133.0, 136.3, 138.5, 154.6, 156.4, 198.4. **HRMS–DART** (*m/z*): [M+ NH_4]$^+$ calcd for $C_{23}H_{22}NO_2$, 344.1645; found, 344.1647.

1,2-Diphenyl-3-(thiazol-2-yl)propan-1-one (6aak)

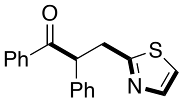

6aak

The product **6aak** was purified by flash chromatography on silica gel (Biotage Selekt, 100:0–90:10, hexane/EtOAc) (Fig. 2, 29.3 mg, 0.10 mmol, 50% isolated yield). Pale yellow solid. **M.p.** 106–111 °C. **IR** (neat) 698, 758, 1107, 1248, 1447, 1496, 1597, 1680 cm^{-1}. **^{1}H NMR** (600 MHz, $CDCl_3$) δ 3.44 (dd, J = 15.0, 7.2 Hz, 1H), 3.97 (dd, J = 15.0, 7.2 Hz, 1H), 5.28 (t, J = 7.2 Hz, 1H), 7.11 (d, J = 3.6 Hz, 1H), 7.21 (m, 1H), 7.26–7.31 (m, 4H), 7.36 (t, J = 7.8 Hz, 2H), 7.46 (t, J = 7.2 Hz, 1H), 7.63 (d, J = 3.6 Hz, 1H), 7.96 (d, J = 7.8 Hz, 2H). **^{13}C NMR** (150.9 MHz, $CDCl_3$) δ 37.0, 53.5, 118.4, 127.6, 128.1, 128.3, 128.4, 128.6, 128.8, 128.9, 129.0, 129.2, 132.9, 136.2, 138.2, 142.2, 168.0, 198.4. **HRMS–DART** (*m/z*): $[M+ NH_4]^+$ calcd for $C_{18}H_{19}N_2OS$, 294.0947; found, 294.0945.

3-(Benzo[d]thiazol-2-yl)-1,2-diphenylpropan-1-one (6aal)

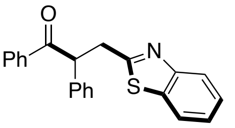

6aal

The product **6aal** was purified by flash chromatography on silica gel (Biotage Selekt, 100:0–90:10, hexane/EtOAc) (Fig. 2, 48.7 mg, 0.14 mmol, 71% isolated yield). Light green solid. **M.p.** 91–96 °C. **IR** (neat) 698, 729, 758, 944, 1116, 1241, 1435, 1447, 1597, 1679 cm^{-1}. **^{1}H NMR** (400 MHz, $CDCl_3$) δ 3.52 (dd, J = 15.2, 7.2 Hz, 1H), 4.07 (dd, J = 15.2, 7.2 Hz, 1H), 5.39 (t, J = 7.2 Hz, 1H), 7.18–7.47 (m, 10H), 7.76 (d, J = 7.6 Hz, 1H), 7.90 (d, J = 7.6 Hz, 1H), 7.97–7.99 (m, 2H). **^{13}C NMR** (100.5 MHz, $CDCl_3$) δ 38.1, 53.2, 121.4, 122.5, 124.7, 125.7, 127.5, 128.2, 128.5, 128.9, 129.2, 133.0, 135.3, 136.2, 138.1, 153.0, 169.2, 198.2. **HRMS–DART** (*m/z*): $[M+H]^+$ calcd for $C_{22}H_{18}NOS$, 344.1104; found, 344.1106.

3-(4-Chlorophenyl)-1,2-diphenylpropan-1-one (6aam)

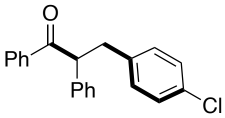

6aam

The product **6aam** was purified by flash chromatography on silica gel (Biotage Selekt, 100:0–90:10, hexane/EtOAc) (Fig. 2, 21.8 mg, 0.07 mmol, 34% isolated yield). The spectrum data of **6aam** was consistent with the literature.[11]

2-Phenyl-3-(thiophen-2-yl)-1-(p-tolyl)propan-1-one (6bag)

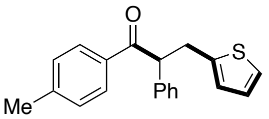

6bag

The product **6bag** was purified by flash chromatography on silica gel (Biotage Selekt, 100:0–90:10, hexane/EtOAc) (Fig. 2, 34.3 mg, 0.11 mmol, 56% isolated yield). Yellow solid. **M.p.** 67–71 °C. **IR** (neat) 698, 820, 1174, 1235, 1260, 1604, 1674 cm^{-1}. **^{1}H NMR** (400 MHz, $CDCl_3$) δ 2.33 (s, 3H), 3.27 (dd, J = 14.4, 6.4 Hz, 1H), 3.78 (dd, J = 14.8, 6.4 Hz, 1H), 4.83 (t, J = 6.4 Hz, 1H), 6.68 (d, J = 2.8 Hz, 1H), 6.80–6.83 (m, 1H), 7.04–7.05 (m, 1H), 7.15 (d, J = 8.4 Hz, 2H), 7.20 (m, 1H), 7.27–7.29 (m, 4H), 7.84 (d, J = 8.4 Hz, 2H). **^{13}C NMR** (100.5 MHz, $CDCl_3$) δ 21.6, 34.1, 55.9, 123.5, 125.6, 126.6, 127.2, 128.2, 128.8, 128.9, 129.2, 134.0, 138.9, 142.2, 143.8, 198.4. **HRMS–DART** (*m/z*): $[M+H]^+$ calcd for $C_{20}H_{19}OS$, 307.1151; found, 307.1154.

1-[4-(Tert-butyl)phenyl]-2-phenyl-3-(thiophen-2-yl)propan-1-one (6cag)

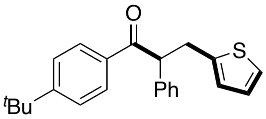

6cag

The product **6cag** was purified by flash chromatography on silica gel (Biotage Selekt, 100:0–90:10, hexane/EtOAc) (Fig. 2, 46.0 mg, 0.13 mmol, 66% isolated yield). Pale yellow solid. **M.p.** 86–91 °C. **IR** (neat) 698, 1108, 1237, 1268, 1604, 1677, 2963 cm^{-1}. **^{1}H NMR** (400 MHz, $CDCl_3$) δ 1.27 (s, 9H), 3.26 (dd, J = 14.8, 7.2 Hz, 1H), 3.78 (dd, J = 14.4, 7.2 Hz, 1H), 4.85 (t, J = 7.2 Hz, 1H), 6.68 (d, J = 2.4 Hz, 1H), 6.82 (m, 1H), 7.05 (m, 1H), 7.21 (m, 1H), 7.25–7.31 (m, 4H), 7.38 (d, J = 8.8 Hz, 2H), 7.89 (d, J = 8.8 Hz, 2H). **^{13}C NMR** (150.9 MHz, $CDCl_3$) δ 31.0, 34.2, 35.0, 55.9, 123.6, 125.5, 125.6, 126.6, 127.3, 128.2, 128.7, 128.9, 133.9, 138.9, 142.2, 156.7, 198.3. **HRMS–DART** (*m/z*): $[M+H]^+$ calcd for $C_{23}H_{25}OS$, 349.1621; found, 349.1624.

1-(4-Fluorophenyl)-2-phenyl-3-(thiophen-2-yl)propan-1-one (6dag)

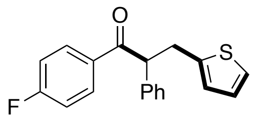

6dag

The product **6dag** was purified by flash chromatography on silica gel (Biotage Selekt, 100:0–90:10, hexane/EtOAc) (Fig. 2, 22.3 mg, 0.07 mmol, 36% isolated yield). Yellow oil. **IR** (neat) 569, 695, 838, 1154, 1203, 1228, 1595, 1678 cm^{-1}. **^{1}H NMR** (400 MHz, $CDCl_3$) δ 3.27 (dd, J = 14.8, 7.2 Hz, 1H), 3.78 (dd, J = 14.8, 7.2 Hz, 1H), 4.79 (t, J = 7.2 Hz, 1H), 6.68 (d, J = 2.4 Hz, 1H), 6.83 (m, 1H), 7.01–7.07 (m, 3H), 7.20–7.31 (m, 5H), 7.94–7.97 (m, 2H). **^{13}C NMR** (100.5 MHz, $CDCl_3$) δ 34.1, 56.1, 123.7, 125.7, 126.7, 127.5, 128.1, 129.1, 131.4 (d, $J_{C\text{–}F}$ = 9.6 Hz), 132.9 (d, $J_{C\text{–}F}$ = 2.9 Hz), 138.5, 141.9, 165.5 (d, $J_{C\text{–}F}$ = 255 Hz), 197.2. **^{19}F NMR** (376.5 MHz, $CDCl_3$) δ –105.1. **HRMS–DART** (*m/z*): $[M+NH_4]^+$ calcd for $C_{19}H_{19}FNOS$, 328.1166; found, 328.1165.

1-(4-Bromophenyl)-2-phenyl-3-(thiophen-2-yl)propan-1-one (6eag)

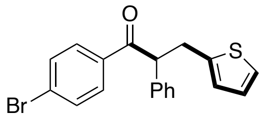

6eag

The product **6eag** was purified by flash chromatography on silica gel (Biotage Selekt, 100:0–90:10, hexane/EtOAc) (Fig. 2, 40.0 mg, 0.11 mmol, 54% isolated yield). Pale yellow solid. **M.p.** 89–93 °C. **IR** (neat) 699, 1010, 1071, 1397, 1583, 1680 cm^{-1}. **^{1}H NMR** (400 MHz, $CDCl_3$) δ 3.26 (dd, J = 14.8, 7.2 Hz, 1H), 3.78 (dd, J = 14.8, 7.2 Hz, 1H), 4.77 (t, J = 7.2 Hz, 1H), 6.68 (m, 1H), 6.83 (m, 1H), 7.06 (m, 1H), 7.20–7.31 (m, 5H), 7.48–7.50 (m, 2H), 7.78 (d, J = 8.8 Hz, 2H). **^{13}C NMR** (100.5 MHz, $CDCl_3$) δ 34.0, 56.2, 123.7, 125.7, 126.7, 127.5, 128.1, 128.2, 129.1, 130.2, 131.8, 135.2, 138.3, 141.8, 197.8. **HRMS–DART** (*m/z*): $[M+H]^+$ calcd for $C_{19}H_{16}BrOS$, 371.0100; found, 371.0102.

2-Phenyl-3-(thiophen-2-yl)-1-[4-(trifluoromethoxy)phenyl]propan-1-one (6fag)

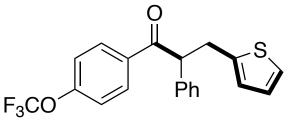

6fag

The product **6fag** was purified by flash chromatography on silica gel (Biotage Selekt, 100:0–80:20, hexane/EtOAc) (Fig. 2, 47.4 mg, 0.13 mmol, 63% isolated yield). Pale yellow oil. **IR** (neat) 693, 1014, 1065, 1111, 1124, 1320, 1685 cm^{-1}. **^{1}H NMR** (400 MHz, $CDCl_3$) δ 3.28 (dd, J = 15.2, 7.2 Hz, 1H), 3.80 (dd, J = 15.2, 7.2 Hz, 1H), 4.82 (t, J = 7.2 Hz, 1H), 6.69 (d, J = 2.4 Hz,1H), 6.84 (m, 1H), 7.07 (m, 1H), 7.21–7.32 (m, 5H), 7.62 (d, J = 8.0 Hz, 2H), 8.00 (d, J = 7.6 Hz, 2H). **^{13}C NMR** (100.5 MHz, $CDCl_3$) δ 34.0, 56.6, 123.0 (q, $J_{C–F}$ = 271.4 Hz), 123.8, 125.6 (q, $J_{C–F}$ = 3.9 Hz), 125.8, 126.7, 127.7, 128.2, 129.0, 129.2, 134.1 (q, $J_{C–F}$ = 32.6 Hz) 137.9, 139.2, 141.6, 197.9. **^{19}F NMR** (376.5 MHz, $CDCl_3$) δ –63.2. **HRMS–DART** (*m/z*): $[M+NH_4]^+$ calcd for $C_{20}H_{19}F_3NO_2S$, 394.1083; found, 394.1080.

2-Phenyl-3-(thiophen-2-yl)-1-[4-(trifluoromethyl)phenyl]propan-1-one (6gag)

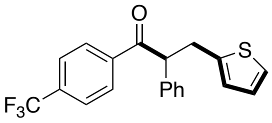

6gag

The product **6gag** was purified by flash chromatography on silica gel (Biotage Selekt, 100:0–90:10, hexane/EtOAc) (Fig. 2, 28.0 mg, 0.08 mmol, 39% isolated yield). Yellow oil. **IR** (neat) 694, 1065, 1111, 1124, 1166, 1320, 1685 cm^{-1}. **^{1}H NMR** (400 MHz, $CDCl_3$) δ 3.28 (dd, J = 14.4, 7.2 Hz, 1H), 3.80 (dd, J = 14.4, 7.2 Hz, 1H), 4.82 (t, J = 7.2 Hz, 1H), 6.69 (d, J = 2.4 Hz, 1H), 6.84 (m, 1H), 7.07 (m, 1H), 7.21–7.32 (m, 5H), 7.62 (d, J = 8.4 Hz, 2H), 8.00 (d, J = 8.4 Hz, 2H). **^{13}C NMR** (100.5 MHz, $CDCl_3$) δ 34.0, 56.6, 123.5 (q, $J_{C–F}$ = 258.2 Hz), 123.8, 125.6 (q, $J_{C–F}$ = 3.8 Hz), 125.8, 126.7, 127.7, 128.2, 129.0, 129.2, 134.1 (q, $J_{C–F}$ = 32.2 Hz), 137.9, 139.2, 141.6, 197.9. **^{19}F NMR** (376.5 MHz,

$CDCl_3$) δ –63.2. **HRMS–DART** (*m/z*): $[M+NH_4]^+$ calcd for $C_{20}H_{19}F_3NOS$, 378.1134; found, 378.1134.

1-(4-Methoxyphenyl)-2-phenyl-3-(thiophen-2-yl)propan-1-one (6hag)

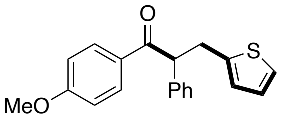

6hag

The product **6hag** was purified by flash chromatography on silica gel (Biotage Selekt, 100:0–80:20, hexane/EtOAc) (Fig. 2, 18.7 mg, 0.06 mmol, 29% isolated yield). Yellow oil. **IR** (neat) 697, 1027, 1165, 1236, 1256, 1596, 1668 cm^{-1}. **^{1}H NMR** (600 MHz, $CDCl_3$) δ 3.26 (dd, J = 15.0, 7.2 Hz, 1H), 3.76–3.80 (m, 4H), 4.80 (t, J = 7.2 Hz, 1H), 6.68 (d, J = 3.0 Hz, 1H), 6.81–6.85 (m, 3H), 7.05 (d, J = 5.4 Hz, 1H), 7.19–7.21 (m, 1H), 7.25–7.29 (m, 4H), 7.93 (d, J = 8.4 Hz, 2H). **^{13}C NMR** (150.9 MHz, $CDCl_3$) δ 34.1, 55.4, 55.6, 113.7, 123.5, 125.6, 126.6, 127.2, 128.1, 128.9, 129.5, 131.0, 139.1, 142.3, 163.3, 197.2. **HRMS–DART** (*m/z*): $[M+H]^+$ calcd for $C_{20}H_{19}O_2S$, 323.1100; found, 323.1104.

1-[4-(Methylthio)phenyl]-2-phenyl-3-(thiophen-2-yl)propan-1-one (6iag)

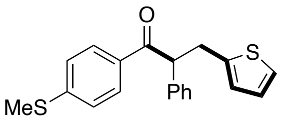

6iag

The product **6iag** was purified by flash chromatography on silica gel (Biotage Selekt, 100:0–90:10, hexane/EtOAc) (Fig. 2, 35.8 mg, 0.11 mmol, 53% isolated yield). Pale yellow solid. **M.p.** 81–86 °C. **IR** (neat) 699, 819, 1093, 1238, 1401, 1587, 1672 cm^{-1}. **^{1}H NMR** (400 MHz, $CDCl_3$) δ 2.45 (s, 3H), 3.26 (dd, J = 14.8, 6.8 Hz, 1H), 3.78 (dd, J = 14.8, 6.8 Hz, 1H), 4.80 (t, J = 6.8 Hz, 1H), 6.68 (d, J = 2.4 Hz,1H), 6.82 (m, 1H), 7.05 (m, 1H), 7.16 (d, J = 8.4 Hz, 2H), 7.21 (m, 1H), 7.25–7.28 (m, 4H), 7.85 (d, J = 8.0 Hz, 2H). **^{13}C NMR** (150.9 MHz, $CDCl_3$) δ 14.6, 34.1, 55.8, 123.6, 124.9, 125.6, 126.6, 127.3, 128.2, 129.0, 129.1, 132.8, 138.8, 142.1, 145.9, 197.7. **HRMS–DART** (*m/z*): $[M+H]^+$ calcd for $C_{20}H_{19}OS_2$, 339.0872; found, 339.0873.

2-Phenyl-1,3-di(thiophen-2-yl)propan-1-one (6jag)

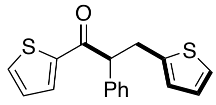

6jag

The product **6jag** was purified by flash chromatography on silica gel (Biotage Selekt, 100:0–90:10, hexane/EtOAc) (Fig. 2, 31.6 mg, 0.11 mmol, 53% isolated yield). Pale yellow solid. **M.p.** 77–80 °C. **IR** (neat) 697, 1167, 1234, 1410, 1493, 1509, 1668 cm^{-1}. **^{1}H NMR** (400 MHz, $CDCl_3$) δ 3.25 (dd, J = 14.8, 7.2 Hz, 1H), 3.77 (dd, J = 14.8, 7.2 Hz, 1H), 4.63 (t, J = 7.2 Hz, 1H), 6.68 (m, 1H), 6.82 (m, 1H), 7.05 (m, 1H), 7.18–7.25 (m, 2H), 7.28 (t, J = 4.4 Hz, 4H), 7.50 (m, 1H), 8.00 (m, 1H). **^{13}C NMR**

(100.5 MHz, $CDCl_3$) δ 33.8, 57.8, 123.6, 125.7, 126.1, 126.6, 127.4, 127.4, 128.1, 129.0, 132.8, 138.7, 141.6, 141.9, 193.0. **HRMS–DART** (*m/z*): $[M+H]^+$ calcd for $C_{17}H_{15}OS_2$, 299.0559; found, 299.0557.

1-(Furan-2-yl)-2-phenyl-3-(thiophen-2-yl)propan-1-one (6kag)

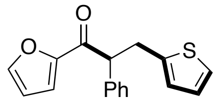

6kag

The product **6kag** was purified by flash chromatography on silica gel (Biotage Selekt, 100:0–90:10, hexane/EtOAc) (Fig. 2, 36.1 mg, 0.13 mmol, 64% isolated yield). Orange solid. **M.p.** 75–79 °C. **IR** (neat) 698, 762, 881, 1271, 1464, 1566, 1670 cm^{-1}. **^{1}H NMR** (600 MHz, $CDCl_3$) δ 3.28 (dd, J = 14.4, 7.2 Hz, 1H), 3.78 (dd, J = 14.4, 7.2 Hz, 1H), 4.67 (t, J = 7.2 Hz, 1H), 6.44 (dd, J = 3.6, 1.8 Hz, 1H), 6.71 (d, J = 3.6 Hz, 1H), 6.83 (m, 1H), 7.06 (d, J = 5.4 Hz, 1H), 7.15 (d, J = 3.6 Hz, 1H), 7.24 (m, 1H), 7.29 (t, J = 7.2 Hz, 2H), 7.34 (d, J = 7.8 Hz, 2H), 7.51 (d, J = 0.6 Hz, 1H), **^{13}C NMR** (150.9 MHz, $CDCl_3$) δ 33.1, 56.0, 112.3, 118.1, 123.6, 125.7, 126.7, 127.4, 128.3, 128.8, 138.2, 141.8, 146.6, 152.3, 187.8. **HRMS–DART** (*m/z*): $[M+H]^+$ calcd for $C_{17}H_{15}O_2S$, 283.0787; found, 283.0791.

1-(Benzofuran-2-yl)-2-phenyl-3-(thiophen-2-yl)propan-1-one (6lag)

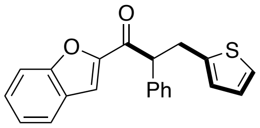

6lag

The product **6lag** was purified by flash chromatography on silica gel (Biotage Selekt, 100:0–90:10, hexane/EtOAc) (Fig. 2, 39.9 mg, 0.12 mmol, 60% isolated yield). Yellow solid. **M.p.** 82–88 °C. **IR** (neat) 698, 752, 1139, 1158, 1282, 1552, 1675 cm^{-1}. **^{1}H NMR** (400 MHz, $CDCl_3$) δ 3.32 (dd, J = 14.8, 7.2 Hz, 1H), 3.84 (dd, J = 14.8, 7.2 Hz, 1H), 4.81 (t, J = 7.2 Hz, 1H), 6.74 (d, J = 3.2 Hz, 1H), 6.83 (dd, J = 5.2, 3.2 Hz, 1H), 7.05 (d, J = 5.2 Hz, 1H), 7.21–7.26 (m, 2H), 7.30 (t, J = 7.6 Hz, 2H), 7.38–7.44 (m, 3H), 7.47 (s, 1H), 7.52 (d, J = 8.4 Hz, 1H), 7.62 (d, J = 7.6 Hz, 1H). **^{13}C NMR** (150.9 MHz, $CDCl_3$) δ 33.1, 56.5, 112.4, 113.9, 113.9, 123.2, 123.7, 123.7, 123.8, 125.8, 126.7, 126.9, 127.6, 128.3, 128.3, 128.9, 138.0, 141.6, 152.0, 155.6, 189.6. **HRMS–DART** (*m/z*): $[M+H]^+$ calcd for $C_{21}H_{17}O_2S$, 330.0944; found, 330.0943.

2-Phenyl-1-(pyridin-2-yl)-3-(thiophen-2-yl)propan-1-one (6mag)

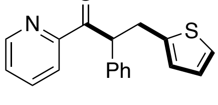

6mag

The product **6mag** was purified by flash chromatography on silica gel (Biotage Selekt, 100:0–90:10, hexane/EtOAc) (Fig. 2, 27.0 mg, 0.09 mmol, 46% isolated yield). Colorless oil. **IR** (neat) 618, 698, 754, 767, 995, 1333, 1434, 1693 cm^{-1}. **^{1}H NMR** (600 MHz, $CDCl_3$) δ 3.35 (dd, J = 15.0, 7.2 Hz, 1H), 3.79 (dd, J = 15.0, 7.2 Hz, 1H), 5.75 (t, J = 7.2 Hz, 1H), 6.74 (d, J = 3.6 Hz, 1H), 6.81 (dd, J =

5.4, 3.6 Hz, 1H), 7.03 (d, J = 5.4 Hz, 1H), 7.17 (t, J = 7.2 Hz, 1H), 7.24–7.26 (m, 2H), 7.37 (m, 1H), 7.41 (d, J = 7.8 Hz, 2H), 7.74 (m, 1H), 7.79 (d, J = 7.8 Hz, 1H), 8.04 (d, J = 4.8 Hz, 1H). **^{13}C NMR** (150.9 MHz, $CDCl_3$) δ 33.0, 52.7, 122.7, 123.4, 125.4, 126.5, 127.0, 127.0, 128.5, 128.9, 136.7, 138.3, 142.3, 148.9, 152.7, 200.2. **HRMS–DART** (*m/z*): $[M+H]^+$ calcd for $C_{18}H_{16}NOS$, 294.0947; found, 294.0942.

2-(4-Methoxyphenyl)-1-phenyl-3-(thiophen-2-yl)propan-1-one (6abg)

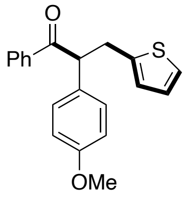

6abg

The product **6abg** was purified by flash chromatography on silica gel (Biotage Selekt, 100:0–80:20, hexane/EtOAc) (Fig. 2, 0.1 mmol scale, 8.7 mg, 0.03 mmol, 27% isolated yield). Pale yellow oil. **IR** (neat) 690, 1033, 1178, 1251, 1447, 1510, 1608, 1678 cm^{-1}. **^{1}H NMR** (400 MHz, $CDCl_3$) δ 3.25 (dd, J = 14.4, 7.2 Hz, 1H), 3.72–3.78 (m, 4H), 4.80 (t, J = 7.2 Hz, 1H), 6.68 (d, J = 3.6 Hz, 1H), 6.79–6.84 (m, 3H), 7.06 (m, 1H), 7.18–7.21 (m, 2H), 7.36 (t, J = 7.6 Hz, 2H), 7.47 (m, 1H), 7.92 (d, J = 6.8 Hz, 2H). **^{13}C NMR** (100.5 MHz, $CDCl_3$) δ 34.1, 55.2 (×2C), 114.4, 123.6, 125.6, 126.6, 128.5, 128.7, 129.3, 131.4, 132.9, 136.6, 142.2, 158.8, 199.0. **HRMS–DART** (*m/z*): $[M+ NH_4]^+$ calcd for $C_{20}H_{22}NO_2S$, 340.1366; found, 340.1369.

2-[4-(Benzyloxy)phenyl]-1-phenyl-3-(thiophen-2-yl)propan-1-one (6acg)

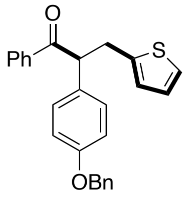

6acg

The product **6acg** was purified by flash chromatography on silica gel (Biotage Selekt, 100:0–90:10, hexane/EtOAc) (Fig. 2, 0.1 mmol scale, 18.3 mg, 0.05 mmol, 46% isolated yield). Pale yellow solid. **M.p.** 77–83 °C. **IR** (neat) 692, 738, 828, 1025, 1176, 1243, 1508, 1677 cm^{-1}. **^{1}H NMR** (600 MHz, $CDCl_3$) δ 3.25 (dd, J = 15.0, 7.2 Hz, 1H), 3.75 (dd, J = 15.0, 7.2 Hz, 1H), 4.80 (t, J = 7.2 Hz, 1H), 4.99 (s, 2H), 6.68 (s, 1H), 6.82 (t, J = 4.2 Hz, 1H), 6.89 (d, J = 8.4 Hz, 2H), 7.05 (d, J = 4.8 Hz, 1H), 7.20 (d, J = 8.0 Hz, 2H), 7.28 (m, 1H), 7.35–7.40 (m, 6H), 7.46 (t, J = 7.6 Hz, 1H), 7.92 (d, J = 7.8 Hz, 2H). **^{13}C NMR** (150.9 MHz, $CDCl_3$) δ 34.1, 55.1, 70.0, 115.3, 123.6, 125.6, 126.6, 127.5, 128.0, 128.5, 128.6, 128.7, 129.3, 130.9, 132.9, 136.6, 136.8, 142.2, 158.1, 199.0. **HRMS–DART** (*m/z*): $[M+ NH_4]^+$ calcd for $C_{26}H_{26}NO_2S$, 416.1679; found, 416.1672.

2-(4-Chlorophenyl)-1-phenyl-3-(thiophen-2-yl)propan-1-one (6agd)

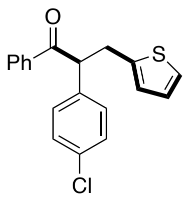

6adg

The product **6adg** was purified by flash chromatography on silica gel (Biotage Selekt, 100:0–90:10, hexane/EtOAc) (Fig. 2, 0.1 mmol scale, 14.7 mg, 0.05 mmol, 45% isolated yield). Pale yellow solid. **M.p.** 79–83 °C. **IR** (neat) 689, 719, 816, 1014, 1092, 1447, 1489, 1679 cm^{-1}. **^{1}H NMR** (600 MHz, $CDCl_3$) δ 3.27 (dd, J = 15.0, 7.2 Hz, 1H), 3.74 (dd, J = 15.0, 7.2 Hz, 1H), 4.82 (t, J = 7.2 Hz, 1H), 6.67 (d, J = 3.6 Hz, 1H), 6.83 (dd, J = 5.4, 3.6 Hz, 1H), 7.07 (dd, J = 5.4, 1.2 Hz, 1H), 7.20–7.26 (m, 4H), 7.38 (t, J = 7.8 Hz, 2H), 7.49 (t, J = 7.2 Hz, 1H), 7.90 (d, J = 7.2 Hz, 2H). **^{13}C NMR** (150.9 MHz, $CDCl_3$) δ 34.0, 55.3, 123.8, 125.8, 126.7, 128.6, 128.7, 129.1, 129.6, 133.2, 133.3, 136.3, 137.0, 141.5, 198.5. **HRMS–DART** (*m/z*): $[M+ H]^+$ calcd for $C_{19}H_{16}ClOS$, 327.0605; found, 327.0602.

2-(6-Methoxynaphthalen-2-yl)-1-phenyl-3-(thiophen-2-yl)propan-1-one (6aeg)

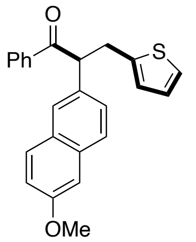

6aeg

The product **6aeg** was purified by flash chromatography on silica gel (Biotage Selekt, 100:0–80:20, hexane/EtOAc) (Fig. 2, 0.1 mmol scale, 14.9 mg, 0.04 mmol, 40% isolated yield). Yellow oil. **IR** (neat) 687, 751, 850, 1030, 1204, 1222, 1264, 1603, 1676 cm^{-1}. **^{1}H NMR** (600 MHz, $CDCl_3$) δ 3.36 (dd, J = 15.0, 7.2 Hz, 1H), 3.84–3.89 (m, 4H), 4.98 (t, J = 7.2 Hz, 1H), 6.70 (s, 1H), 6.81–6.82 (m, 1H), 7.05–7.07 (m, 2H), 7.11 (m, 1H), 7.33–7.39 (m, 3H), 7.44 (m, 1H), 7.65–7.68 (m, 3H), 7.96 (d, J = 6.6 Hz, 2H). **^{13}C NMR** (150.9 MHz, $CDCl_3$) δ 34.2, 55.3, 56.0, 105.5, 119.1, 123.6, 125.7, 126.6, 126.7, 127.0, 127.6, 128.5, 128.7, 129.0, 129.3, 132.9, 133.7, 133.8, 136.6, 142.1, 157.8, 198.9. **HRMS–DART** (*m/z*): $[M+ H]^+$ calcd for $C_{24}H_{21}O_2S$, 373.1257; found, 373.1259.

7. Characterization Data for Amide Substrates

(2-Iodothiophen-3-yl)(pyrrolidin-1-yl)methanone (7a)

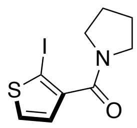

7a

Pale Yellow Solid. **M.p.** 81–82 °C. **IR** (neat) 711, 838, 1436, 1522, 1613, 2874, 2948, 2968, 3070, 3478 cm^{-1}. **^{1}H NMR** (400 MHz, $CDCl_3$) δ 1.88–2.01 (m, 4H), 3.30 (t, J = 6.4 Hz, 2H), 3.65 (t, J = 6.8 Hz, 2H), 6.90 (d, J = 5.6 Hz, 1H), 7.43 (d, J = 5.6 Hz, 1H). **^{13}C NMR** (100.6 MHz, $CDCl_3$) δ 24.5, 26.0, 45.8, 48.4, 73.5, 126.9, 132.1, 144.3, 165.2. **HRMS–DART** (*m/z*): $[M+H]^+$ calcd for $C_9H_{11}INOS$; 307.9601, found 307.9601.

(2-Iodothiophen-3-yl)(piperidin-1-yl)methanone (7b)

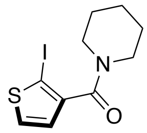

7b

Pale Yellow Solid. **M.p.** 47–50 °C. **IR** (neat) 720, 852, 1266, 1465, 1522, 1616, 2853, 2934, 2992, 3070 cm^{-1}. **^{1}H NMR** (400 MHz,$CDCl_3$) δ 1.57 (m, 2H), 1.68 (m, 4H), 3.24–3.27 (m, 2H), 3.73 (m, 2H), 6.84 (d, J = 5.6 Hz, 1H), 7.43 (d, J = 5.6 Hz, 1H). **^{13}C NMR** (100.6 MHz,$CDCl_3$) δ 24.5, 25.6, 26.7, 42.9, 48.2, 73.3, 127.1, 132.1, 143.3, 165.4. **HRMS–DART** (*m/z*): $[M+H]^+$ calcd for $C_{10}H_{13}INOS$; 321.9757, found 321.9751.

Azepan-1-yl(2-iodothiophen-3-yl)methanone (7c)

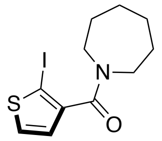

7c

Pale Yellow Solid. **M.p.** 66–69 °C. **IR** (neat) 722, 1152, 1276, 1300, 1428, 1523, 1617, 2852, 2924, 3069 cm^{-1}. **^{1}H NMR** (400 MHz, $CDCl_3$) δ 1.58–1.68 (m, 6H), 1.82–1.88 (m, 2H), 3.29–3.32 (m, 2H), 3.69 (t, J = 6.0 Hz, 2H), 6.84 (d, J = 5.6 Hz, 1H), 7.43 (d, J = 5.6 Hz, 1H). **^{13}C NMR** (100.6 MHz, $CDCl_3$) δ 26.6, 27.3, 27.8, 29.2, 45.8, 49.2, 73.2, 126.9, 132.1, 143.9, 166.9. **HRMS–DART** (*m/z*): $[M+H]^+$ calcd for $C_{11}H_{15}INOS$; 335.9914, found 339.9911.

***tert*-Butyl 4-(2-Iodothiophene-3-carbonyl)piperazine-1-carboxylate (7d)**

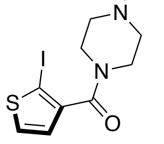

7d

Yellow Solid. **M.p.** 153–157 °C. **IR** (neat) 752, 1164, 1259, 1418, 1630, 1688, 2861, 2927, 2975, 3097 cm^{-1}. **^{1}H NMR** (400 MHz, $CDCl_3$) δ 1.47 (s, 9H), 3.20 (m, 2H), 3.45 (m, 2H), 3.54 (m, 2H), 3.76 (m, 2H), 6.86 (d, J = 5.6 Hz, 1H), 7.47 (d, J = 5.6 Hz, 1H). **^{13}C NMR** (100.6 MHz, $CDCl_3$) δ 28.3, 41.8, 43.7, 46.9, 73.9, 80.4, 127.1, 132.6, 142.3, 154.4, 165.7. **HRMS–DART** (*m/z*): [M+H]$^+$ calcd for $C_{14}H_{20}IN_2O_3S$; 423.0234, found 423.0230.

Methyl (2-Iodothiophene-3-carbonyl)prolinate (7e)

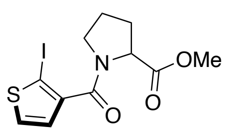

7e

Pale Yellow Oil. **IR** (neat) 719, 1171, 1201, 1428, 1623, 1738, 2878, 2950, 3101, 3471 cm^{-1}. Peaks for two rotamers (74:26) were given: **^{1}H NMR** (400 MHz, $CDCl_3$) δ 1.88–2.14 (m, 3H), 2.32 (m, 1H), 3.38–3.57 (m, 0.74 × 2H), 3.58 (s, 0.26 × 3H), 3.77–3.82 (m, 0.74 × 1H + 2H), 4.29 (m, 0.26 × 1H), 4.68 (m, 0.74 × 1H), 6.81(d, J = 5.6 Hz, 0.26 × 1H), 6.95 (d, J = 5.6 Hz, 0.74 × 1H), 7.40 (d, J = 5.6 Hz, 0.26 × 1H), 7.44 (d, J = 5.6 Hz, 0.74 × 1H). Peaks for the minor rotamer were enclosed in parenthesis: **^{13}C NMR** (100.6 MHz, $CDCl_3$) δ (22.9), 24.9, 29.4, (31.3), (46.3), 48.7, 52.2, (52.4), 58.7, (60.7), (73.9), 74.1, 127.0, (127.2), 132.1, (132.1), 142.9, (143.5), 165.1, (165.6), (172.2), 172.2. **HRMS–DART** (*m/z*): [M+H]$^+$ calcd for $C_{11}H_{13}INO_3S$; 365.9655, found 365.9656.

***N,N*-Diethyl-2-iodothiophene-3-carboxamide (7f)**

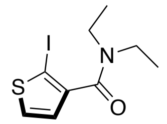

7f

Yellow Solid. **M.p.** 70–73 °C. **IR** (neat) 843, 1278, 1433, 1456, 1472, 1620, 2871, 2932, 2972, 3070 cm^{-1}. **^{1}H NMR** (400 MHz, $CDCl_3$) δ 1.09 (t, J = 7.2 Hz, 3H), 1.28 (t, J = 7.2 Hz, 3H), 3.21 (q, J = 7.2 Hz, 2H), 3.57 (q, J = 7.2 Hz, 2H), 6.85 (d, J = 5.2 Hz, 1H), 7.44 (d, J = 5.2 Hz, 1H). **^{13}C NMR** (100.6 MHz, $CDCl_3$) δ 12.8, 14.3, 39.2, 43.0, 73.2, 126.8, 132.1, 143.5, 166.4. **HRMS–DART** (*m/z*): [M+H]$^+$ calcd for $C_9H_{13}INOS$; 309.9757, found 309.9759.

***N*-Benzyl-*N*-ethyl-2-iodothiophene-3-carboxamide (7g)**

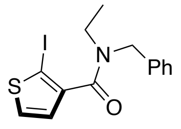

7g

Pale Yellow Solid. **M.p.** 76–78 °C. **IR** (neat) 702, 727, 1113, 1429, 1451, 1626, 2933, 2973, 3028, 3065 cm^{-1}. Peaks for two rotamers. (51:49) were given: **^{1}H NMR** (400 MHz, $CDCl_3$) δ 1.05 (t, J = 7.2 Hz, 0.51 × 3H), 1.24 (t, J = 7.2 Hz, 0.49 × 3H), 3.14 (q, J = 7.2 Hz, 0.51 × 2H), 3.52 (q, J = 7.2 Hz, 0.49 × 2H), 4.33 (s, 0.49 × 2H), 4.79 (s, 0.51 × 2H), 6.86 (d, J = 5.6 Hz, 0.49 × 1H), 6.90 (d, J = 5.6 Hz, 0.51 × 1H), 7.15 (d, J = 7.2 Hz, 1H), 7.28–7.46 (m, 5H). Peaks for the minor rotamer were

enclosed in parenthesis: **^{13}C NMR** (100.6 MHz, $CDCl_3$) δ(12.3), 13.7, 39.4, (42.3), 46.8, (51.7), 73.4, (74.0), (126.9), 127.0, (127.1), 127.4, (127.6), 128.3, 128.5, (128.7), 132.3 (132.3), (136.5), 136.9, (142.8), 143.1, (166.9), 167.1. **HRMS–DART** (*m/z*): $[M+H]^+$ calcd for $C_{14}H_{15}INOS$; 371.9914, found 371.9914.

{4-(2-Chlorodibenzo[*b,f*][1,4]oxazepin-11-yl)piperazin-1-yl}(2-iodothiophen-3-yl)methanone (7h)

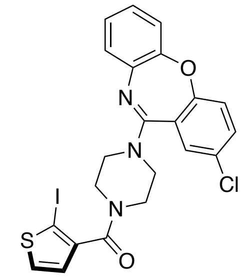

7h

White Solid. **M.p.** 138–140 °C. **IR** (neat) 750, 1013, 1239, 1469, 1587, 1629, 2854, 2915, 3001, 3969 cm^{-1}. **^{1}H NMR** (400 MHz, $CDCl_3$) δ 3.47–3.92 (m, 8H), 6.88 (d, J = 5.6 Hz, 1H), 7.02 (m, 1H), 7.08–7.15 (m, 3H), 7.20 (d, J = 8.8 Hz, 1H), 7.33 (m, 1H), 7.41 (dd, J = 8.8, 2.4 Hz, 1H), 7.47 (d, J = 5.6 Hz, 1H). **^{13}C NMR** (100.6 MHz, $CDCl_3$) δ 41.6, 46.7, 47.4, 48.0, 74.0, 120.1, 122.8, 124.7, 125.0, 125.8, 127.1, 127.2, 128.7, 130.4, 132.6, 132.8, 139.6, 142.3, 151.6, 158.6, 159.3, 165.7. **HRMS–DART** (*m/z*): $[M+H]^+$ calcd for $C_{22}H_{18}ClIN_3O_2S$; 549.9847, found 549.9849.

{(3*S*,4*R*)-3-[(Benzo[*d*][1,3]dioxol-5-yloxy)methyl]-4-(4-fluorophenyl)piperidin-1-yl}(2-iodothio phen-3-yl)methanone (7i)

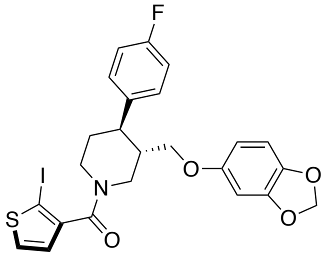

7i

White Solid. **M.p.** 162–163 °C. **IR** (neat) 746, 1037, 1180, 1442, 1486, 1502, 1569, 1623, 2917, 3004 cm^{-1}. Peaks for two rotamers were given: **^{1}H NMR** (400 MHz, $CDCl_3$) δ 1.81–1.98 (m, 2H), 2.15 (m, 1H), 2.75–3.00 (m, 2H), 3.16 (m, 1H), 3.40 (m, 0.50 × 1H), 3.53 (m, 1H), 3.70 (m, 1H), 3.82 (d, J = 12.8 Hz, 0.50 × 1H), 4.90 (d, J = 12.8 Hz, 0.50 × 1H), 5.04 (d, J = 12.8 Hz, 0.50 × 1H), 5.89 (s, 2H), 6.00 (d, J = 8.4 Hz, 0.50 × 1H), 6.18 (m, 1H), 6.39 (s, 0.50 × 1H), 6.62 (dd, J = 16.0, 8.4 Hz, 1H), 6.90 (m, 1H), 6.99 (m, 2H), 7.13–7.17 (m, 2H), 7.48 (d, J = 5.2 Hz, 1H). Peaks for two rotamers were given: **^{13}C NMR** (100.6 MHz, $CDCl_3$) δ 33.5, 34.6, 41.9, 42.5, 42.8, 43.6, 44.0, 45.1, 47.6, 50.4, 68.1, 68.4, 73.7, 73.8, 97.8, 97.9, 101.0, 105.3, 105.5, 107.8, 115.5 (d, J_{C-F} = 21.0 Hz), 115.6 (d, J_{C-F} = 21.1 Hz), 126.9, 127.0, 128.6 (d, J_{C-F} = 8.3 Hz), 128.7 (d, J_{C-F} = 9.6 Hz), 132.3, 132.3, 138.4, 141.7, 142.7, 142.8, 148.1, 153.5, 154.1, 161.6 (d, J_{C-F} = 245.3 Hz), 165.5 (only observed peaks). **^{19}F**

NMR (376.5 MHz, $CDCl_3$) δ –115.8, –115.6. **HRMS–DART** (*m/z*): $[M+H]^+$ calcd for $C_{24}H_{22}FINO_4S$; 566.0293, found 566.0292.

(3-Iodothiophen-2-yl)(pyrrolidin-1-yl)methanone (7a-A)

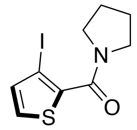

7a-A

Pale Yellow Oil. **IR** (neat) 737, 862, 1392, 1440, 1613, 2873, 2948, 2968, 3071, 3488 cm^{-1}. **^{1}H NMR** (400 MHz, $CDCl_3$) δ 1.92–2.00 (m, 4H), 3.38–3.42 (m, 2H), 3.64–3.68 (m, 2H), 7.05 (m, 1H), 7.72 (m, 1H). **^{13}C NMR** (100.6 MHz, $CDCl_3$) δ 24.5, 26.0, 46.2, 48.9, 78.6, 127.6, 135.3, 137.7, 162.6. **HRMS–DART** (*m/z*): $[M+H]^+$ calcd for $C_9H_{11}INOS$; 307.9601, found 307.9600.

(2-Iodofuran-3-yl)(pyrrolidin-1-yl)methanone (7a-B)

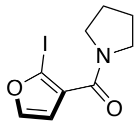

7a-B

Pale Yellow Oil. **IR** (neat) 736, 888, 1143, 1416, 1479, 1606, 2875, 2970, 3109, 3470 cm^{-1}. **^{1}H NMR** (400 MHz, $CDCl_3$) δ 1.91–1.97 (m, 4H), 3.47 (t, J = 6.0 Hz, 2H), 3.61 (t, J = 6.0 Hz, 2H), 6.50 (m, 1H), 7.56 (m, 1H). **^{13}C NMR** (100.6 MHz, $CDCl_3$) δ 24.2, 26.1, 45.9, 48.5, 91.3, 111.5, 128.6, 147.6, 162.6. **HRMS–DART** (*m/z*): $[M+H]^+$ calcd for $C_9H_{11}INO_2$; 291.9829, found 291.9823.

(2-Iodophenyl)(pyrrolidin-1-yl)methanone (7a-C)

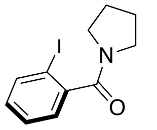

7a-C

The spectrum data of **7a-C** was consistent with the literature.[9]

8. Procedure for C(sp^3)–H Acylation of Secondary Amides

The reaction to produce 8ma in Fig. 4 is representative. Thiazolium salt **N1** (8.3 mg, 0.02 mmol) and amide **7a** (61.4 mg, 0.2 mmol) were placed in a Schlenk tube containing a magnetic stirring bar. The tube was sealed with a Teflon®-coated silicon rubber septum, and then evacuated and filled with nitrogen. Cs_2CO_3 (71.7 mg, 0.22 mmol), degassed DMSO (400 μL) and 2-pyridinecarboxaldehyde (**1m**) (42.8 mg, 0.4 mmol) were added to the vial. After 6 h stirring at 80 °C, the reaction mixture was treated with saturated NH_4Cl aqueous solution (400 μL), then extracted with diethyl ether (4 times) and dried over sodium sulfate. After filtration through a short plug of aluminum oxide (1 g) with diethyl ether as an eluent, the resulting solution was evaporated under reduced pressure. After the volatiles were removed under reduced pressure, flash column chromatography on silica gel (100:0–50:50, hexane/EtOAc) gave **8ma** (53.0 mg, 0.19 mmol) in 93% yield.

9. Characterization Data for α-Aminoketones

2-[(Thiophene-3-carbonyl)prolyl]pyridine (8ma)

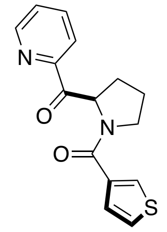

8ma

The product **8ma** was purified by flash chromatography on silica gel (100:0–50:50, hexane/EtOAc) (Fig. 4, 53.0 mg, 0.19 mmol, 93% isolated yield). Pale Yellow Oil. **IR** (neat) 740, 995, 1359, 1434, 1606, 1704, 2874, 2972, 3085, 3474 cm^{-1}. Peaks for two rotamers (83:17) were given: ^{1}**H NMR** (400 MHz, $CDCl_3$) δ 1.97–2.13 (m, 3H), 2.53 (m, 1H), 3.82–3.95 (m, 2H), 6.09 (m, 1H), 7.09–7.15 (m, 0.17 × 2H), 7.30 (m, 0.83 × 1H), 7.36 (m, 0.17 × 1H), 7.42–7.49 (m, 0.83 × 1H + 1H), 7.77–7.86 (m, 0.83 × 1H + 1H), 7.94 (m, 0.17 × 1H), 8.10 (d, J = 8.0 Hz, 0.83 × 1H), 8.60 (m, 0.17 × 1H), 8.69 (d, J = 4.4 Hz, 0.83 × 1H). Peaks for the minor rotamer were enclosed in parenthesis: ^{13}**C NMR** (100.6 MHz, $CDCl_3$) δ (22.4), 25.7, 29.3, (31.4), (47.4), 49.9, 61.4, (63.1), (122.5), 122.6, 125.1, (125.5), (125.8), (127.1), 127.2, (127.5), 127.9, 128.0, 136.8, (137.0), 137.1, (137.8), 148.7, (148.9), (151.3), 152.3, 163.5, (165.5), 198.3, (198.8). **HRMS–DART** (*m/z*): $[M+H]^+$ calcd for $C_{15}H_{15}N_2O_2S$; 287.0849, found 287.0849.

2-(Benzoylprolyl)pyridine (8ma-C)

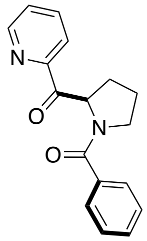

8ma-C

The product **8ma-C** was purified by flash chromatography on silica gel (Biotage Selekt, 100:0–50:50, hexane/EtOAc) (Fig. 4, 42.1 mg, 0.15 mmol, 75% isolated yield). Yellow oil. **IR** (neat) 658, 701, 719, 744, 996, 1413, 1576, 1671, 1705, 2978 cm^{-1}. Peaks for two rotamers (82:18) were given: ^{1}**H NMR** (400 MHz, $CDCl_3$) δ 1.95–2.05 (m, 3H), 2.42–2.59 (m, 1H), 3.62–3.93 (m, 2H), 5.86 (d, J = 7.6 Hz, 0.18 × 1H), 6.08 (t, J = 6.0 Hz, 0.82 × 1H), 7.15 (s, 0.18 × 3H), 7.31 (s, 0.18 × 2H), 7.39–7.41 (m, 0.82 × 3H), 7.47–7.50 (m, 0.82 × 1H), 7.61 (d, J = 7.2 Hz, 0.82 × 2H), 7.76 (t, J = 7.6 Hz, 0.18 × 1H), 7.83–7.89 (m, 1H), 8.11 (d, J = 8.0 Hz, 0.82 × 1H), 8.51 (d, J = 4.4 Hz, 0.18 × 1H), 8.71 (d, J = 4.4 Hz, 0.82 × 1H). Peaks for the minor rotamer were enclosed in parenthesis: ^{13}**C NMR** (100.6 MHz, $CDCl_3$) δ (22.5), 25.6, 29.6, (31.3), (47.1), 50.3, 61.1, (63.3), (122.3), 122.6, (126.5), 127.2, 127.3, (127.4), (128.0), 128.1, (129.2), 129.9, 136.4, (136.8), 137.3, (148.7), 148.7, (151.2), 152.3, 168.9, (170.4), 198.2, (198.9) (only observed peaks). **HRMS–DART** (*m/z*): $[M+H]^+$ calcd for $C_{17}H_{17}N_2O_2$; 281.1285, found 281.1285.

(2-Picolinoylpiperidin-1-yl)(thiophen-3-yl)methanone (8mb)

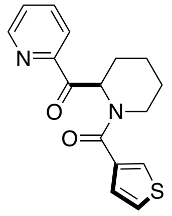

8mb

The product **8mb** was purified by flash chromatography on silica gel (100:0–60:40, hexane/EtOAc) (Fig. 4, 53.0 mg, 0.18 mmol, 88% isolated yield). Pale Yellow solid. **M.p.** 114–115 °C. **IR** (neat) 741, 981, 1219, 1423, 1617, 1700, 2858, 2938, 3100, 3477 cm^{-1}. Peaks for two rotamers (74:26) were given: **^{1}H NMR** (400 MHz, $CDCl_3$) δ 1.43–1.78 (m, 4H), 1.89 (m, 0.26 × 1H), 2.06 (m, 0.74 × 1H), 2.28 (m, 0.26 × 1H), 2.41 (m, 0.74 × 1H), 3.39 (m, 0.26 × 1H), 3.71–3.88 (m, 0.74 × 2H), 4.74 (m, 0.26 × 1H), 6.00 (m, 0.26 × 1H), 6.47 (m, 0.74 × 1H), 7.07 (m, 0.26 × 1H), 7.21 (m, 1H), 7.32 (m, 1H), 7.47 (m, 1H), 7.55 (m, 0.74 × 1H), 7.85 (dt, J = 7.6, 1.6 Hz, 1H), 8.04 (m, 1H), 8.53 (m, 0.26 × 1H), 8.70 (m, 0.74 × 1H). Peaks for the minor rotamer were enclosed in parenthesis: **^{13}C NMR** (100.6 MHz, $CDCl_3$) δ 20.7, (24.9), 25.5, 26.6, (27.7), (40.0), 46.0, 55.3, (59.9), 122.4, (125.0), 125.5, (125.9), 126.4, 127.1 (× 2C), (127.3), 136.7, 136.8, (137.1), 148.8, (151.7), 152.3, 167.2, 199.7, (200.0) (only observed peaks). **HRMS–DART** (*m/z*): $[M+H]^+$ calcd for $C_{16}H_{17}N_2O_2S$; 301.1005, found 301.1007.

(2-Picolinoylazepan-1-yl)(thiophen-3-yl)methanone (8mc)

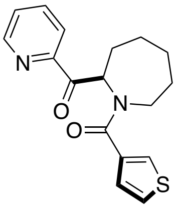

8mc

The product **8mc** was purified by flash chromatography on silica gel (100:0–50:50, hexane/EtOAc) (Fig. 4, 53.9 mg, 0.17 mmol, 86% isolated yield). Pale Yellow Solid. **M.p.** 103–105 °C. **IR** (neat) 742, 1218, 1275, 1425, 1523, 1614, 1702, 2854, 2927, 3105 cm^{-1}. Peaks for two rotamers (76:24) were given: **^{1}H NMR** (400 MHz, $CDCl_3$) δ 1.32–2.06 (m, 7H), 2.63 (m, 1H), 3.15 (m, 0.24 × 1H), 3.57 (m, 0.76 × 1H), 3.98 (m, 0.76 × 1H), 4.56 (m, 0.24 × 1H), 5.79 (m, 0.24 × 1H), 6.07 (m, 0.76 × 1H), 6.98 (m, 0.24 × 1H), 7.16 (m, 0.24 × 2H), 7.22 (m, 0.76 × 1H), 7.29 (m, 0.76 × 1H), 7.46 (m, 1H), 7.54 (m, 0.76 × 1H), 7.84 (m, 1H), 8.04 (m, 1H), 8.52 (m, 0.24 × 1H), 8.70 (m, 0.76 × 1H). Peaks for the minor rotamer were enclosed in parenthesis: **^{13}C NMR** (100.6 MHz, $CDCl_3$) δ (25.8), 26.8, (28.5), 28.9, 29.3, (29.8), 31.5, (31.8), (44.0), 47.3, 61.1, (63.0), 122.4, (122.6), (124.6), 125.4, (125.9), 126.2, (126.5), 127.0, 127.2, (127.4), 136.8, 136.8, (137.0), (137.1), 148.7, (148.9), (151.5), 152.4, 167.2, (167.6), 199.9, (200.4). **HRMS–DART** (*m/z*): $[M+H]^+$ calcd for $C_{17}H_{19}N_2O_2S$; 315.1162, found 315.1168.

tert-Butyl 3-Picolinoyl-4-(thiophene-3-carbonyl)piperazine-1-carboxylate (8md)

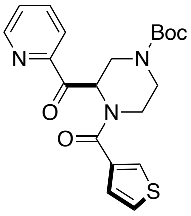

8md

The product **8md** was purified by flash chromatography on silica gel (100:0–50:50, hexane/EtOAc) (Fig. 4, 71.8 mg, 0.18 mmol, 89% isolated yield). White Solid. **M.p.** 54–59 °C. **IR** (neat) 747, 996, 1129, 1166, 1419, 1627, 1694, 2932, 2976, 3101 cm^{-1}. Peaks for two rotamers (70:30) were given: **^{1}H NMR** (400 MHz, $CDCl_3$) δ 1.06 (s, 0.70 × 9H), 1.25-1.47 (m, 0.30 × 9H), 2.94-4.95 (m, 6H), 5.91 (m, 0.30 × 1H), 6.44 (m, 0.70 × 1H), 7.04 (m, 0.30 × 1H), 7.19–7.27 (m, 1H), 7.34 (m, 1H), 7.51 (m, 1H), 7.61 (m, 0.70 × 1H), 7.88 (m, 1H), 8.06 (m, 1H), 8.56 (m, 0.30 × 1H), 8.72 (m, 0.70 × 1H). Peaks for two rotamers were given: **^{13}C NMR** (100.6 MHz, $CDCl_3$) δ 27.6, 28.0, 28.3, 39.4, 42.7, 43.2, 44.0, 45.0, 45.8, 46.4, 55.9, 60.6, 79.9, 80.2, 122.3, 122.5, 125.8, 125.9, 126.1, 126.3, 126.4, 126.6, 126.9, 127.0, 127.4, 127.6, 135.9, 136.9, 137.2, 148.8, 151.4, 151.8, 153.7, 154.4, 167.1, 196.8, 197.2 (only observed peaks). **HRMS–DART** (*m/z*): [M+H]$^+$ calcd for $C_{20}H_{24}N_3O_4S$; 402.1482, found 402.1484.

Methyl 5-Picolinoyl-1-(thiophene-3-carbonyl)pyrrolidine-2-carboxylate (8me)

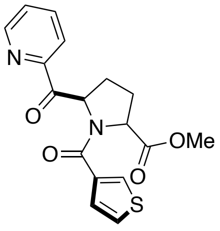

8me

The product **8me** was purified by flash chromatography on silica gel (100:0–50:50, hexane/EtOAc) (Fig. 4, 23.4 mg, 0.068 mmol, 34% isolated yield). The diasteromeric ratio (>20:1) was determined by ^{1}H-NMR analysis. Pale Yellow Oil. **IR** (neat) 743, 1200, 1349, 1428, 1523, 1627, 1705, 1741, 2953, 3105 cm^{-1}. Peaks for two rotamers (54:46) were given: **^{1}H NMR** (400 MHz, $CDCl_3$) δ 2.03–2.28 (m, 0.46 × 1H + 2H), 2.49 (m, 1H), 2.67 (m, 0.54 × 1H), 3.65 (s, 0.46 × 3H), 3.80 (s, 0.54 × 3H), 4.80 (d, J = 7.6 Hz, 0.46 × 1H), 4.97 (d, J = 8.4 Hz, 0.54 × 1H), 6.31 (d, J = 8.4 Hz, 1H), 7.09 (m, 0.54 × 1H), 7.15 (m, 0.54 × 1H), 7.25 (m, 0.46 × 1H), 7.30 (m, 0.46 × 1H), 7.48-7.51 (m, 0.54 × 1H + 1H), 7.56 (m, 0.46 × 1H), 7.79–7.93 (m, 0.54 × 1H + 1H), 8.11 (d, J = 8.0 Hz, 0.46 × 1H), 8.63 (d, J = 4.4 Hz, 0.54 × 1H), 8.71 (d J = 4.8 Hz, 0.46 × 1H). Peaks for two rotamers were given: **^{13}C NMR** (100.6 MHz, $CDCl_3$) δ 26.9, 27.1, 30.0, 30.3, 52.3, 52.5, 60.5, 61.6, 61.8, 63.1, 122.6, 122.7, 125.5, 125.8, 126.5, 126.6, 127.2, 127.3, 127.4, 127.7, 136.7, 136.9, 136.9, 137.1, 148.9, 149.0, 151.1, 152.0, 164.9, 165.2, 172.6, 172.8, 197.5, 198.3. **HRMS–DART** (*m/z*): [M+H]$^+$ calcd for $C_{17}H_{17}N_2O_4S$; 345.0904, found 345.0905.

N-Ethyl-*N*-[1-oxo-1-(pyridin-2-yl)propan-2-yl]thiophene-3-carboxamide (8mf)

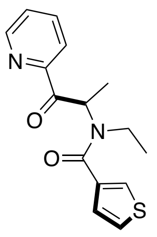

8mf

The product **8mf** was purified by flash chromatography on silica gel (100:0–50:50, hexane/EtOAc) (Fig. 4, 53.5 mg, 0.19 mmol, 93% isolated yield). Pale Yellow Solid. **M.p.** 54–57 °C. **IR** (neat) 741, 995, 1222, 1279, 1436, 1613, 1703, 2937, 2979, 3100 cm^{-1}. Peaks for two rotamers (7:3) were given: **^{1}H NMR** (400 MHz, $CDCl_3$) δ 1.10–1.31 (m, 3H), 1.65–1.71 (m, 3H), 3.22–3.72 (m, 2H), 5.14 (m, 0.70 × 1H), 5.97 (m, 0.30 × 1H), 6.97–7.51 (m, 4H), 7.81 (m, 0.70 × 1H), 7.95 (m, 1H), 8.22 (d, *J* = 7.6 Hz, 0.30 × 1H), 8.59 (m, 1H). Peaks for the minor rotamer were enclosed in parenthesis: **^{13}C NMR** (100.6 MHz, $CDCl_3$) δ 14.2 15.4, (38.5), 44.2, 57.7, 122.3, 122.6, 125.3, 125.6, (126.3), (126.6), 127.8, (136.4), 136.9, 137.1, (147.7), (148.6), 149.4, 151.6, (154.0), 166.3, 196.9, (198.7) (only observed peaks). **HRMS–DART** (*m/z*): $[M+H]^+$ calcd for $C_{15}H_{17}N_2O_2S$; 289.1005, found 289.1004.

N-Benzyl-*N*-[1-oxo-1-(pyridin-2-yl)propan-2-yl]thiophene-3-carboxamide (8mg-major) *N*-Ethyl-*N*-[2-oxo-1-phenyl-2-(pyridin-2-yl)ethyl]thiophene-3-carboxamide (8mg-minor)

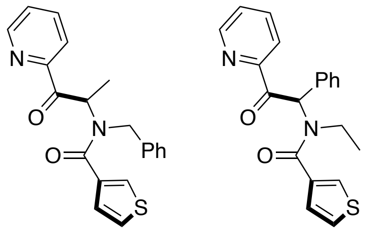

8mg-major **8mg-minor**

The product **8mg** was purified by flash chromatography on silica gel (100:0–50:50, hexane/EtOAc) and Gel Permeation Chromatography (Fig. 4, **major**: 28.1 mg, 0.080 mmol, 40% isolated yield; **minor**: 10.6 mg, 0.030 mmol, 15% isolated yield).

8mg-major: Pale Yellow Oil. **IR** (neat) 697, 743, 974, 1421, 1435, 1621, 1704, 2940, 2989, 3061 cm^{-1}. Peaks for two rotamers (7:3) were given: **^{1}H NMR** (400 MHz, $CDCl_3$) δ 1.45 (d, *J* = 7.2 Hz, 3H), 4.61–5.01 (m, 2H), 5.56 (m, 0.70 × 1H), 6.09 (m, 0.30 × 1H), 7.06–7.26 (m, 3H), 7.26–7.44 (m, 6H), 7.69–7.83 (m, 0.30 × 1H + 1H), 8.02 (m, 0.70 × 1H), 8.62 (m, 1H). Peaks for the minor rotamer were enclosed in parenthesis: **^{13}C NMR** (100.6 MHz, $CDCl_3$) δ 14.5, 53.1, 57.7, (122.4), 122.8, 125.7, 126.4, 126.8, 127.0, 127.2, 127.5, (127.9), (128.2), 128.7, 128.8, (136.0), 137.0, (137.2), 138.2, 148.2, (149.5), 153.3, 167.4, 199.1 (only observed peaks). **HRMS–DART** (*m/z*): $[M+H]^+$ calcd for $C_{20}H_{19}N_2O_2S$; 351.1162, found 351.1161.

8mg-minor: Pale Yellow Oil. **IR** (neat) 613, 743, 995, 1281, 1437, 1584, 1620, 1769, 2976, 3056 cm^{-1}. **^{1}H NMR** (400 MHz, $CDCl_3$) δ 0.91 (t, *J* = 7.2 Hz, 3H), 3.37–3.68 (m, 2H), 6.98 (m, 1H), 7.18 (dd, *J* = 1.2, 5.2 Hz, 1H), 7.28–7.50 (m, 8H), 7.80 (t, *J* = 7.6 Hz, 1H), 8.07 (d, *J* = 8.0 Hz 1H), 8.56 (m, 1H). **^{13}C NMR** (100.6 MHz, $CDCl_3$) δ 15.2, 43.2, 64.2, 122.6, 125.8, 125.9, 126.8, 126.9, 128.3,

128.9, 130.3, 134.5, 136.9, 137.0, 148.5, 153.2, 167.8, 196.0. **HRMS–DART** (*m/z*): $[M+H]^+$ calcd for $C_{20}H_{19}N_2O_2S$; 351.1162, found 351.1163.

{4-(2-Chlorodibenzo[*b,f*][1,4]oxazepin-11-yl)-1-(thiophene-3-carbonyl)piperazin-2-yl}(pyridin-2-yl)methanone (8mh)

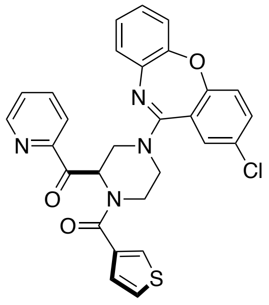

8mh

The product **8mh** was purified by flash chromatography on silica gel (100:0–50:50, hexane/EtOAc) (Fig. 4, 47.1 mg, 0.089 mmol, 45% isolated yield). **8mh** was purified by flash chromatography on silica gel (0–50% EtOAc/hexane). Pale Yellow solid. **M.p.** 174–180 °C. **IR** (neat) 737, 1240, 1347, 1425, 1470, 1560, 1587, 1604, 1704, 3059 cm^{-1}. Peaks for two rotamers (1:1) were given: **^{1}H NMR** (400 MHz, $CDCl_3$) δ 3.26–4.13 (m, 0.50 × 1H + 5H), 4.69–4.72 (m, 0.50 × 1H), 5.01 (bs, 1H), 5.97 (bs, 0.50 × 1H), 6.53–7.91 (m, 0.50 × 1H + 12H), 8.51–8.63 (m, 0.50 × 1H). Peaks for two rotamers were given: **^{13}C NMR** (100.6 MHz, $CDCl_3$) δ 29.7, 40.0, 40.1, 45.4, 45.7, 49.0, 56.0, 60.6, 70.6, 120.1, 120.4, 122.7, 124.5, 124.9, 125.4, 126.0, 126.3, 126.6, 127.0, 127.1, 128.5, 130.2, 132.6, 136.0, 136.9, 148.5, 151.6, 159.1, 167.2, 196.3 (only observed peaks). **HRMS–ESI** (*m/z*): $[M+H]^+$ calcd for $C_{28}H_{22}ClN_4O_3S$; 529.1096, found 529.1092.

{(4*R*,5*S*)-5-[(Benzo[*d*][1,3]dioxol-5-yloxy)methyl]-4-(4-fluorophenyl)-1-(thiophene-3-carbonyl)piperidin-2-yl}(pyridin-2-yl)methanone (8mi)

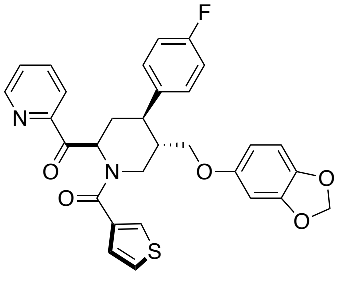

8mi

The product **8mi** was purified by flash chromatography on silica gel (100:0–50:50, hexane/EtOAc) (Fig. 4, 49.3 mg, 0.091 mmol, 45% isolated yield). White Solid. **M.p.** 75–80 °C. **IR** (neat) 727, 833, 908, 1180, 1486, 1509, 1622, 2244, 2884, 3054 cm^{-1}. Peaks for two rotamers and two diastereomers were given (the ratios were not determined): **^{1}H NMR** (400 MHz, $CDCl_3$) δ 1.61–2.19 (m, 3H), 2.30–3.05 (m, 3H), 3.36–4.39 (m, 5H), 5.64–6.38 (m, 0.50 × 1H + 5H), 6.59–6.65 (m, 1H), 6.77 (d, J = 5.2 Hz, 0.50 × 1H), 6.95–7.64 (m, 4H), 7.77–8.10 (m, 2H), 8.49 (d, J = 4.0 Hz, 0.50 × 1H), 8.65 (s, 0.50 × 1H). Peaks for two rotamers and two diastereomers were given: **^{13}C NMR** (100.6 MHz, $CDCl_3$) δ 33.5, 33.7, 34.5, 35.4, 38.3, 40.0, 40.5, 41.6, 41.7, 42.2, 42.5, 43.0, 44.0, 44.4, 45.0, 45.7, 47.4, 47.6,

48.8, 50.4, 52.2, 54.7, 57.5, 59.7, 65.5, 68.2, 68.4, 68.7, 73.7, 97.8, 97.9, 100.8, 100.9, 101.1, 101.1, 104.5, 105.4, 105.4, 105.5, 107.2, 107.5, 107.7, 107.8, 115.4 (d, $J_{C–F}$ = 20.9 Hz), 115.4 (d, $J_{C–F}$ = 21.0 Hz), 115.6 (d, $J_{C–F}$ = 21.1 Hz), 121.8, 122.5, 122.5, 125.7, 125.7, 125.9, 126.1, 126.5, 127.1, 127.2, 127.4, 128.6 (d, $J_{C–F}$ = 7.8 Hz), 128.8 (d, $J_{C–F}$ = 7.6 Hz), 128.8 (d, $J_{C–F}$ = 7.7 Hz), 129.0 (d, $J_{C–F}$ = 7.7 Hz), 132.3, 135.6, 136.0, 136.2, 136.5, 136.8, 136.9, 137.0, 137.2, 138.0 (d, $J_{C–F}$ = 3.0 Hz), 138.5 (d, $J_{C–F}$ = 3.0 Hz), 138.9, 141.3, 141.5, 141.7, 141.8, 147.4, 147.8, 148.1, 148.1, 148.4, 148.5, 148.8, 149.0, 151.4, 151.8, 152.6, 153.0, 153.6, 153.7, 154.1, 161.5 (ddd, $J_{C–F}$ = 244.8 Hz), 161.5 (ddd, $J_{C–F}$ = 245.0 Hz), 161.6 (ddd, $J_{C–F}$ = 245.4 Hz), 161.7 (ddd, $J_{C–F}$ = 246.1 Hz), 165.6, 167.0, 167.3, 167.9, 197.0, 199.7, 200.3 (only observed peaks). **^{19}F NMR** (376.5 MHz, $CDCl_3$) δ –116.0, –115.8, –115.6, –115.5. **HRMS–DART** (*m/z*): $[M+H]^+$ calcd for $C_{30}H_{26}FN_2O_5S$; 545.1541, found 545.1546.

[2-(4-Methylbenzoyl)pyrrolidin-1-yl](thiophen-3-yl)methanone (8ba)

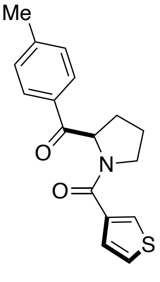

8ba

The product **8ba** was purified by flash chromatography on silica gel (100:0–70:30, hexane/EtOAc) and Gel Permeation Chromatography (Fig. 4, 35.4 mg, 0.12 mmol, 59% isolated yield). Pale Yellow Solid. **M.p.** 112–113 °C. **IR** (neat) 739, 1180, 1228, 1359, 1435, 1605, 1688, 2874, 2973, 3096 cm^{-1}. Peaks for two rotamers (83:17) were given: **^{1}H NMR** (400 MHz, $CDCl_3$) δ 1.93–2.16 (m, 3H), 2.32–2.42 (m, 4H), 3.80–3.94 (m, 2H), 5.38 (m, 0.17 × 1H), 5.68 (m, 0.83 × 1H), 7.12–7.15 (m, 0.17 × 2H), 7.22–7.31 (m, 3H), 7.43 (d, J = 5.2 Hz, 0.83 × 1H), 7.66–7.68 (m, 0.17 × 2H), 7.78 (m, 0.83 × 1H), 7.96 (d, J = 8.0 Hz, 0.83 × 2H). Peaks for the minor rotamer were enclosed in parenthesis: **^{13}C NMR** (100.6 MHz, $CDCl_3$) δ 21.6, (22.2), 25.4, 29.2, (31.3), (46.9), 49.7, 61.6, (63.9), 125.2, (125.7), (125.8), (126.9), 127.9, 128.1, (128.3), 128.6, 129.3, (129.5), 132.8, 137.1, 144.1, (144.6), 163.7, (165.6), 197.3, (197.6) (only observed peaks). **HRMS–DART** (*m/z*): $[M+H]^+$ calcd for $C_{17}H_{18}NO_2S$; 300.1053, found 300.1053.

{2-[4-(*tert*-Butyl)benzoyl]pyrrolidin-1-yl}(thiophen-3-yl)methanone (8ca)

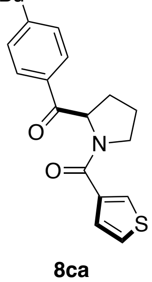

8ca

The product **8ca** was purified by flash chromatography on silica gel (100:0–50:50, hexane/EtOAc) (Fig. 4, 36.9 mg, 0.11 mmol, 54% isolated yield). Pale Yellow Solid. **M.p.** 153–157 °C. IR (neat) 739,

1228, 1361, 1435, 1606, 1688, 2871, 2963, 3089, 3450 cm^{-1}. Peaks for two rotamers (83:17) were given: **^{1}H NMR** (400 MHz, $CDCl_3$) δ 1.35 (s, 9H), 1.95–2.17 (m, 3H), 2.37 (m, 1H), 3.81–3.95 (m, 2H), 5.39 (m, 0.17 × 1H), 5.70 (m, 0.83 × 1H), 7.14–7.17 (m, 0.17 × 2H), 7.30 (m, 1H), 7.44–7.49 (m, 0.17 × 1H + 1H), 7.49–7.51 (d, J = 8.0 Hz, 0.83 × 2H), 7.71–7.73 (m, 0.17 × 2H), 7.78 (m, 0.83 × 1H), 7.99-8.01 (d, J = 8.0 Hz, 0.83 × 2H). Peaks for the minor rotamer were enclosed in parenthesis: **^{13}C NMR** (100.6 MHz, $CDCl_3$) δ (22.2), 25.5, (27.5), 29.2, 31.0, (31.2), (31.3), 35.1, (46.9), 49.7, 61.5, (64.1), 125.2, 125.6, (125.8), (127.0), 127.9, 128.1, (128.2), (128.3), 128.5, (130.5), 132.7, 137.1, 157.0, 163.7, 197.4 (only observed peaks). **HRMS–DART** (*m/z*): [M+H]$^+$ calcd for $C_{20}H_{24}NO_2S$; 342.1522, found 342.1520

Thiophen-3-yl{2-[4-(trifluoromethoxy)benzoyl]pyrrolidin-1-yl}methanone (8fa)

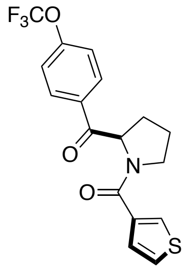

8fa

The product **8fa** was purified by flash chromatography on silica gel (100:0–50:50, hexane/EtOAc) (Fig. 4, 51.2 mg, 0.14 mmol, 69% isolated yield). Pale Yellow Solid. **M.p.** 84–86 °C. **IR** (neat) 1165, 1208, 1256, 1434, 1602, 1695, 2877, 2976, 3106, 3503 cm^{-1}. Peaks for two rotamers (91:9) were given: **^{1}H NMR** (400 MHz, $CDCl_3$) δ 1.94–2.09 (m, 2H), 2.15 (m, 1H), 2.37 (m, 1H), 3.83–3.96 (m, 2H), 5.36 (m, 0.09 × 1H), 5.64 (m, 0.91 × 1H), 7.10 (m, 0.09 × 1H), 7.18 (m, 0.09 × 1H), 7.31–7.33 (m, 0.91 × 1H + 2H), 7.43 (d, J = 5.2 Hz, 0.91 × 1H), 7.80 (m, 1H), 8.12 (d, J = 8.4 Hz, 2H). Peaks for the minor rotamer were enclosed in parenthesis: **^{13}C NMR** (100.6 MHz, $CDCl_3$) δ 22.3, 25.6, 29.0, (31.2), 46.9, (49.7), 61.5, (63.9), 120.2 (q, $J_{C–F}$ = 258.8 Hz), 120.5, 125.4, (125.6), (126.1), (126.7), 127.8, 128.4, 130.2, 130.5 133.8, 136.7, 152.7 (d, $J_{C–F}$ = 1.2 Hz), 163.7, 196.7 (only observed peaks). **^{19}F NMR** (376.5 MHz, $CDCl_3$) δ –57.6. **HRMS–DART** (*m/z*): [M+H]$^+$ calcd for $C_{17}H_{15}F_3NO_3S$; 370.0719, found 370.0719.

Thiophen-3-yl{2-[4-(trifluoromethyl)benzoyl]pyrrolidin-1-yl}methanone (8ga)

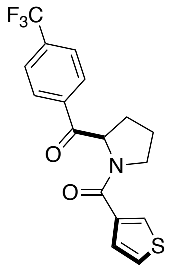

8ga

The product **8ga** was purified by flash chromatography on silica gel (100:0–60:40, hexane/EtOAc) and Gel Permeation Chromatography (Fig. 4, 46.6 mg, 0.13 mmol, 66% isolated yield). Pale Yellow Solid. **M.p.** 116–118 °C. **IR** (neat) 1066, 1130, 1227, 1324, 1435, 1609, 1697, 2877, 2976, 3106 cm–

[1]. Peaks for two rotamers (92:8) were given: **^{1}H NMR** (400 MHz, $CDCl_3$) δ 1.94–2.22 (m, 3H), 2.38 (m, 1H), 3.84–3.97 (m, 2H), 5.39 (m, 0.08 × 1H), 5.64 (m, 0.92 × 1H), 7.09 (m, 0.08 × 1H), 7.18 (m, 0.08 × 1H), 7.33 (m, 1H), 7.43 (d, J = 4.8 Hz, 0.92 × 1H), 7.69–7.85 (m, 0.08 × 1H + 3H), 8.16 (d, J = 8.0Hz, 0.92 × 2H). Peaks for the minor rotamer were enclosed in parenthesis: **^{13}C NMR** (100.6 MHz, $CDCl_3$) δ (22.3), 25.6, 28.9, (31.1), (46.9), 49.7, 61.8, (64.0), 123.5 (q, J_{C-F} = 272.7 Hz), 125.4, 125.7 (q, J_{C-F} = 3.6 Hz), (126.2), (126.7), 127.8, 128.4, 128.8, 134.4 (q, J_{C-F} = 32.7 Hz), 136.6, 138.4, 163.7, 197.4 (only observed peaks). **^{19}F NMR** (376.5 MHz, $CDCl_3$) δ –63.1. **HRMS–DART** (*m/z*): $[M+H]^+$ calcd for $C_{17}H_{15}F_3NO_2S$; 354.0770, found 354.0770.

[2-(4-Methoxybenzoyl)pyrrolidin-1-yl](thiophen-3-yl)methanone (8ha)

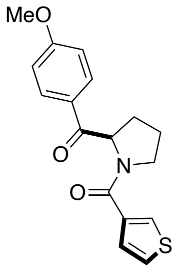

8ha

The product **8ha** was purified by flash chromatography on silica gel (100:0–50:50, hexane/EtOAc) and Gel Permeation Chromatography (Fig. 4, 25.7 mg, 0.086 mmol, 43% isolated yield). Pale Yellow Solid. **M.p.** 119–122 °C. IR (neat) 738, 1169, 1230, 1261, 1434, 1597, 1681, 2875, 2970, 3104 cm^{-1}. Peaks for two rotamers (83:17) were given: **^{1}H NMR** (400 MHz, $CDCl_3$) δ 1.93–2.17 (m, 3H), 2.35 (m, 1H), 3.80–3.94 (m, 5H), 5.36 (m, 0.17 × 1H), 5.68 (m, 0.83 × 1H), 6.91 (d, J = 8.4 Hz, 0.17 × 2H), 6.94 (d, J = 8.0 Hz, 0.83 × 2H), 7.12–7.15 (m, 0.17 × 2H), 7.30 (m, 1H), 7.59 (d, J = 5.2 Hz, 0.83 × 1H), 7.77–7.78 (m, 0.17 × 1H + 1H), 8.05 (d, J = 8.4 Hz, 0.83 × 2H). Peaks for the minor rotamer were enclosed in parenthesis: **^{13}C NMR** (100.6 MHz, $CDCl_3$) δ (22.3), 25.4, 29.3, (31.5), (47.0), 49.7, 55.5, 61.3, (63.7), 113.8, (114.0), 125.2, (125.7), (125.8), (126.8), (127.0), 127.9, 128.1, 128.2, (130.5), 130.8, 137.2, (137.8), 163.6, 163.7, (163.9), 196.2, (196.4) (only observed peaks). **HRMS–DART** (*m/z*): $[M+H]^+$ calcd for $C_{17}H_{18}NO_3S$; 316.1002, found 316.1003.

{2-[4-(Methylthio)benzoyl]pyrrolidin-1-yl}(thiophen-3-yl)methanone (8ia)

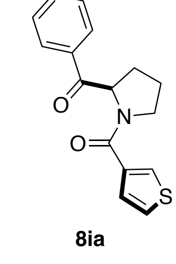

8ia

The product **8ia** was purified by flash chromatography on silica gel (100:0–50:50, hexane/EtOAc) and Gel Permeation Chromatography (Fig. 4, 21.2 mg, 0.064 mmol, 32% isolated yield). White Solid. **M.p.** 117–119 °C. **IR** (neat) 741, 1093, 1227, 1435, 1587, 1611, 1683, 2873, 2975, 3100 cm^{-1}. Peaks for two rotamers (85:15) were given: **^{1}H NMR** (400 MHz, $CDCl_3$) δ 1.91–2.17 (m, 3H), 2.36 (m,

1H), 2.53 (s, 3H), 3.81–3.94 (m, 2H), 5.35 (m, 0.15 × 1H), 5.66 (m, 0.85 × 1H), 7.10 (m, 0.15 × 1H), 7.11 (m, 0.15 × 1H), 7.16–7.32 (m, 3H), 7.43 (m, 0.85 × 1H), 7.68–7.70 (m, 0.15 × 2H), 7.78 (m, 0.85 × 1H), 7.96–7.98 (m, 0.85 × 2H). Peaks for the minor rotamer were enclosed in parenthesis: **^{13}C NMR** (100.6 MHz, $CDCl_3$) δ (14.6), 14.8, (22.3), 25.5, 29.2, (31.4), (47.0), 49.7, 61.4, 125.1, 125.3, (125.7), (125.9), 127.9, 128.2, (128.5), 128.9, 131.5, 137.1, 146.2, 163.7, 196.8 (only observed peaks). **HRMS–DART** (*m/z*): $[M+H]^+$ calcd for $C_{17}H_{18}NO_2S_2$; 332.0774, found 332.0774.

[2-(4-Bromobenzoyl)pyrrolidin-1-yl](thiophen-3-yl)methanone (8ea)

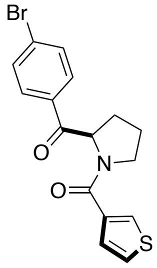

8ea

The product **8ea** was purified by flash chromatography on silica gel (100:0–60:40, hexane/EtOAc) and Gel Permeation Chromatography (Fig. 4, 37.2 mg, 0.10 mmol, 51% isolated yield). Pale Yellow Solid. **M.p.** 127–129 °C. **IR** (neat) 740, 1006, 1221, 1436, 1584, 1610, 1692, 2874, 2974, 3091 cm^{-1}. Peaks for two rotamers (89:11) were given: **^{1}H NMR** (400 MHz, $CDCl_3$) δ 1.92–2.19 (m, 3H), 2.35 (m, 1H), 3.82–3.95 (m, 2H), 5.34 (m, 0.11 × 1H), 5.61 (m, 0.89 × 1H), 7.08 (m, 0.11 × 1H), 7.17 (m, 0.11 × 1H), 7.32 (m, 1H), 7.43 (d, J = 4.8 Hz, 0.89 × 1H), 7.57–7.64 (m, 0.11 × 2H + 2H), 7.78 (m, 0.89 × 1H), 7.92 (d, J = 8.0 Hz, 0.89 × 2H). Peaks for the minor rotamer were enclosed in parenthesis: **^{13}C NMR** (100.6 MHz, $CDCl_3$) δ (22.3), 25.5, 29.0, (31.2), (46.9), 49.7, 61.5, (63.8), 125.4, (125.6), (126.1), (126.7), 127.9, 128.3, 128.4, (129.6), 130.0, 132.0, (132.2), 134.2, 136.8, 163.7, 197.1 (only observed peaks). **HRMS–DART** (*m/z*): $[M+H]^+$ calcd for $C_{16}H_{15}BrNO_2S$; 364.0001, found 364.0001.

Thiophen-2-yl[1-(thiophene-3-carbonyl)pyrrolidin-2-yl]methanone (8ja)

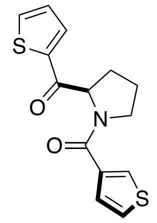

8ja

The product **8ja** was purified by flash chromatography on silica gel (100:0–60:40, hexane/EtOAc) and Gel Permeation Chromatography (Fig. 4, 26.9 mg, 0.092 mmol, 46% isolated yield). Pale Yellow Solid. **M.p.** 110–112 °C. **IR** (neat) 738, 1234, 1357, 1414, 1519, 1608, 1666, 2874, 2974, 3088 cm^{-1}. Peaks for two rotamers (86:14) were given: **^{1}H NMR** (400 MHz, $CDCl_3$) δ 1.97–2.22 (m, 3H), 2.37 (m, 1H), 3.81–3.95 (m, 2H), 5.17 (m, 0.14 × 1H), 5.49 (m, 0.86 × 1H), 7.12 (m, 0.14 × 2H), 7.17 (t, J = 4.2 Hz, 1H), 7.31 (m, 0.86 × 1H), 7.34 (m, 0.14 × 1H), 7.43 (d, J = 4.8 Hz, 0.86 × 1H), 7.55 (m, 0.14 × 1H), 7.67 (d, J = 5.2 Hz, 1H), 7.78 (m, 0.86 × 1H), 7.90 (d, J = 4.0 Hz, 0.86 × 1H). Peaks for the minor rotamer were enclosed in parenthesis: **^{13}C NMR** (100.6 MHz, $CDCl_3$) δ (22.5), 25.5, 29.5,

(32.0), (47.1), 49.7, 62.7, (64.8), 125.3, (125.9), (126.9), 127.9, 128.1, 128.3, (132.2), 132.4, 133.8, (134.5), 136.9, (137.5), 140.4, (141.6), 163.8, (165.7), 190.7, (191.2) (only observed peaks). **HRMS–DART** (*m/z*): $[M+H]^+$ calcd for $C_{14}H_{14}NO_2S_2$; 292.0461, found 292.0468.

Pyrrolidine-1,2-diylbis(thiophen-3-ylmethanone) (8na)

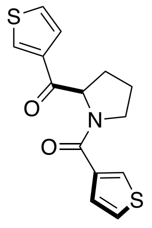

8na

The product **8na** was purified by flash chromatography on silica gel (100:0–60:40, hexane/EtOAc) and Gel Permeation Chromatography (Fig. 4, 32.4 mg, 0.11 mmol, 56% isolated yield). Pale Yellow Oil. **IR** (neat) 740, 1232, 1435, 1521, 1609, 1681, 2875, 2973, 3088, 3505 cm^{-1}. Peaks for two rotamers (86:14) were given: **^{1}H NMR** (400 MHz, $CDCl_3$) δ 1.98–2.17 (m, 3H), 2.34 (m, 1H), 3.80–3.93 (m, 2H), 5.17 (m, 0.14 × 1H), 5.50 (m, 0.86 × 1H), 7.12 (m, 0.14 × 1H), 7.18 (m, 0.14 × 1H), 7.30–7.35 (m, 2H), 7.43 (d, J = 4.8 Hz, 1H), 7.63 (d, J = 4.8 Hz, 0.86 × 1H), 7.78 (m, 0.86 × 1H), 7.91 (m, 0.14 × 1H), 8.23 (m, 0.86 × 1H). Peaks for the minor rotamer were enclosed in parenthesis: **^{13}C NMR** (100.6 MHz, $CDCl_3$) δ (22.3), 25.4, 29.1, (31.4), (47.0), 49.7, 62.8, (65.0), 125.3, (125.8), (125.8), 126.3, (126.8), (126.9), 127.2, 127.9, 128.2, (132.5), 132.6, (136.9), 137.7, (138.6), 140.0, 163.7, (165.6), 192.0, (192.4) (only observed peaks). **HRMS–DART** (*m/z*): $[M+H]^+$ calcd for $C_{14}H_{14}NO_2S_2$; 292.0461, found 292.0460.

[2-(Furan-3-carbonyl)pyrrolidin-1-yl](thiophen-3-yl)methanone (8oa)

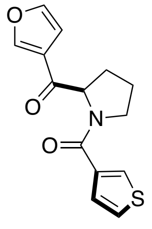

8oa

The product **8oa** was purified by flash chromatography on silica gel (100:0–60:40, hexane/EtOAc) and Gel Permeation Chromatography (Fig. 4, 29.3 mg, 0.11 mmol, 53% isolated yield). Pale Yellow Solid. **M.p.** 124–126 °C. **IR** (neat) 741, 1154, 1434, 1512, 1608, 1684, 2876, 2974, 3123, 3486 cm^{-1}. Peaks for two rotamers (87:13) were given: **^{1}H NMR** (400 MHz, $CDCl_3$) δ 1.95–2.05 (m, 2H), 2.13 (m, 1H), 2.30 (m, 1H), 3.79–3.92 (m, 2H), 4.92 (m, 0.13 × 1H), 5.24 (m, 0.87 × 1H), 6.69 (m, 0.13 × 1H), 6.84 (m, 0.87 × 1H), 7.12 (m, 0.13 × 1H), 7.20 (m, 0.13 × 1H), 7.32 (m, 1H), 7.44 (m, 0.87 × 1H + 1H), 7.77 (m, 0.87 × 1H), 7.86 (m, 0.13 × 1H), 8.21 (m, 0.87 × 1H). Peaks for the minor rotamer were enclosed in parenthesis: **^{13}C NMR** (100.6 MHz, $CDCl_3$) δ (22.4), 25.5, 29.1, (31.6), (47.1), 49.7, 63.3, (65.6), (108.6), 108.9, 125.3, 125.5, (125.9), (126.9), 127.8, 128.3, 136.8, 144.1, (144.3), (147.0), 147.5, 163.8, 192.7, (193.3) (only observed peaks). **HRMS–DART** (*m/z*): $[M+H]^+$ calcd for $C_{14}H_{14}NO_3S$; 276.0689, found 276.0684.

2-Methyl-6-[(thiophene-3-carbonyl)prolyl]pyridine (8pa)

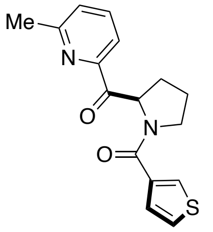

8pa

The product **8pa** was purified by flash chromatography on silica gel (100:0–60:40, hexane/EtOAc) (Fig. 4, 57.8 mg, 0.19 mmol, 96% isolated yield). Yellow Oil. **IR** (neat) 734, 811, 1357, 1430, 1605, 1703, 2874, 2973, 3103, 3469 cm^{-1}. Peaks for two rotamers (82:18) were given: **^{1}H NMR** (400 MHz, $CDCl_3$) δ 1.95–2.12 (m, 3H), 2.45–2.61 (m, 4H), 3.83–3.95 (m, 2H), 6.05 (m, 0.18 × 1H), 6.12 (m, 0.82 × 1H), 7.10–7.16 (m, 0.18 × 2H), 7.29–7.33 (m, 0.82 × 1H + 1H), 7.37 (m, 0.18 × 1H), 7.43 (d, J = 4.8 Hz, 0.82 × 1H), 7.69–7.72 (m, 0.18 × 1H + 1H), 7.77 (m, 0.82 × 1H), 7.90 (d, J = 8.0 Hz, 0.82 × 1H). Peaks for the minor rotamer were enclosed in parenthesis: **^{13}C NMR** (100.6 MHz, $CDCl_3$) δ (22.4), 24.4, 25.6, 29.3, (31.5), (47.4), 49.9, 61.5, (63.0), 119.6, 125.1, (125.3), (125.8), 126.8, (127.2), (127.2), 127.9 (×2C), 136.8, (136.9), 137.2, (137.8), (150.7), 151.6, 157.7, (157.9), 163.4, (165.3), 198.5, (199.2) (only observed peaks). **HRMS–DART** (*m/z*): $[M+H]^+$ calcd for $C_{16}H_{17}N_2O_2S$; 301.1005, found 301.1007.

2-[(Thiophene-3-carbonyl)prolyl]quinoline (8qa)

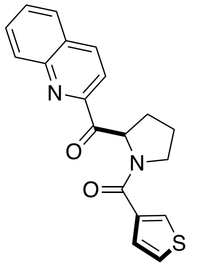

8qa

The product **8qa** was purified by flash chromatography on silica gel (100:0–60:40, hexane/EtOAc) (Fig. 4, 56.0 mg, 0.17 mmol, 83% isolated yield). Yellow solid. **M.p.** 117–119 °C. **IR** (neat) 740, 839, 1359, 1430, 1604, 1702, 2875, 2976, 3105, 3460 cm^{-1}. Peaks for two rotamers (84:16) were given: **^{1}H NMR** (400 MHz, $CDCl_3$) δ 1.94–2.16 (m, 3H), 2.63 (m, 1H), 3.87–4.01 (m, 2H), 6.31 (m, 1H), 7.05 (m, 0.16 × 1H), 7.18 (m, 0.16 × 1H), 7.30 (m, 0.84 × 1H), 7.43 (m, 1H), 7.66 (m, 1H), 7.76–7.80 (m, 0.84 × 1H + 1H), 7.87 (m, 1H), 8.00 (m, 0.16 × 1H), 8.12 (m, 0.16 × 1H), 8.16–8.21 (m, 0.84 × 2H), 8.27 (m, 1H). Peaks for the minor rotamer were enclosed in parenthesis: **^{13}C NMR** (100.6 MHz, $CDCl_3$) δ (22.5), 25.8, 29.5, (31.8), (47.5), 50.0, 61.5, (63.0), (118.4), 118.7, 125.1, (125.4), (125.9), (127.2), 127.6, (127.6), 127.9, 128.0, 128.5, (128.9), 129.6, 129.8, (130.1), (130.5), 130.6, 136.8, (137.1), 137.1, (137.8), (146.9), 147.0, (150.8), 151.8, 163.5, (165.4), 198.6, (199.1) (only observed peaks). **HRMS–DART** (*m/z*): $[M+H]^+$ calcd for $C_{19}H_{17}N_2O_2S$; 337.1005, found 337.1007.

(2-Benzoylpyrrolidin-1-yl)(thiophen-3-yl)methanone (8aa)

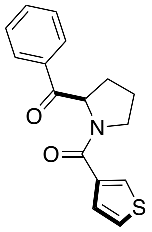

8aa

The yield (Table 2, 74%) of **8aa** in the crude was determined by ^{1}H-NMR analysis using 1,1,2,2-tetrachloroethane as an internal standard. The product **8aa** was purified by flash chromatography on silica gel (100:0–80:20, hexane/EtOAc) (34.1 mg, 0.12 mmol, 60% isolated yield). Pale Yellow Oil. **IR** (neat) 739, 1223, 1359, 1434, 1610, 1690, 2874, 2973, 3103, 3467 cm^{-1}. Peaks for two rotamers (86:14) were given: **^{1}H NMR** (400 MHz, $CDCl_3$) δ 1.95–2.18 (m, 3H), 2.38 (m, 1H), 3.82–3.96 (m, 2H), 5.39 (m, 0.14 × 1H), 5.70 (m, 0.86 × 1H), 7.11–7.16 (m, 0.14 × 2H), 7.31 (m, 1H), 7.43–7.51 (m, 0.86 × 1H + 2H), 7.59 (m, 1H), 7.76–7.80 (m, 0.14 × 1H + 1H), 8.05–8.07 (m, 0.86 × 2H). Peaks for the minor rotamer were enclosed in parenthesis: **^{13}C NMR** (100.6 MHz, $CDCl_3$) δ (22.3), 25.4, 29.1, (31.2), (46.9), 49.7, 61.7, (64.0), 125.3, (125.7), (125.9), (126.8), 127.9, 128.1, 128.5, 128.6, (128.8), 133.2, (133.6), 135.4, (137.0), 137.8, 163.7, 197.8 (only observed peaks). **HRMS–DART** (*m/z*): $[M+H]^+$ calcd for $C_{16}H_{16}NO_2S$; 286.0896, found 286.0895.

(2-Benzoylpyrrolidin-1-yl)(thiophen-2-yl)methanone (8aa-A)

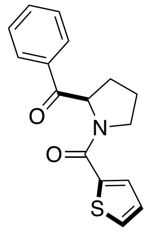

8aa-A

The yield (Table 2, 24%) of **8aa-A** in the crude was determined by ^{1}H-NMR analysis using 1,1,2,2-tetrachloroethane as an internal standard. The product **8aa-A** was purified by flash chromatography on silica gel (100:0–55:45, hexane/EtOAc) (11.4 mg, 0.040 mmol, 20% isolated yield). Pale Yellow Oil. **IR** (neat) 735, 1224, 1403, 1433, 1522, 1597, 1691, 2875, 2974, 3072 cm^{-1}. Peaks for two rotamers (89:11) were given: **^{1}H NMR** (400 MHz, $CDCl_3$) δ 2.00 (m, 1H), 2.11 (m, 1H), 2.22 (m, 1H), 2.35 (m, 1H), 3.92–4.13 (m, 2H), 5.73 (dd, J = 8.6, 4.4 Hz, 1H), 6.85 (m, 0.11 × 1H), 7.10 (m, 0.89 ×1H), 7.21 (m, 0.11 × 1H), 7.29 (m, 0.11 × 1H), 7.46–7.51 (m, 0.89 × 1H + 2H), 7.58 (m, 1H), 7.63 (d, J = 3.6 Hz, 0.89 × 1H), 7.93 (m, 0.11 × 2H), 8.05 (d, J = 7.6 Hz, 0.89 × 2H). **^{13}C NMR** (100.6 MHz, $CDCl_3$) δ 25.5, 28.8, 49.4, 62.4, 127.2, 128.5, 128.6, 130.2, 130.3, 133.3, 135.3, 138.6, 161.4, 197.8. **HRMS–DART** (*m/z*): $[M+H]^+$ calcd for $C_{16}H_{16}NO_2S$; 286.0896, found 286.0893.

(2-Benzoylpyrrolidin-1-yl)(furan-3-yl)methanone (8aa-B)

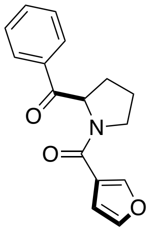

8aa-B

The yield (Table 2, 27%) of **8aa-B** in the crude was determined by ^{1}H-NMR analysis using 1,1,2,2-tetrachloroethane as an internal standard. The product **8aa-B** was purified by flash chromatography on silica gel (100:0–55:45, hexane/EtOAc) (13.0 mg, 0.048 mmol, 24% isolated yield). Pale Yellow Solid. **M.p.** 102–104 °C. **IR** (neat) 751, 904, 1224, 1431, 1564, 1616, 1691, 2877, 2975, 3142 cm^{-1}. Peaks for two rotamers (89:11) were given: **^{1}H NMR** (400 MHz, $CDCl_3$) δ 1.95–2.23 (m, 3H), 2,34 (m, 1H), 3.82–3.99 (m, 2H), 5.35 (m, 0.11 × 1H), 5.68 (dd, J = 8.8, 4.4 Hz, 0.89 ×1H), 6.46 (m, 0.11 × 1H), 6.78 (m, 0.89 × 1H), 7.25–7.26 (m, 0.11 × 2H), 7.43 (m, 0.89 × 1H), 7.47–7.52 (m, 0.11 × 1H + 2H), 7.60 (m, 1H), 7.90 (m, 1H), 8.04–8.06 (m, 0.89 × 2H). Peaks for the minor rotamer were enclosed in parenthesis: **^{13}C NMR** (100.6 MHz, $CDCl_3$) δ(22.0), 25.2, 28.9, (31.4), (47.1), 48.7, 61.8, (63.7), 110.7, 122.0, (128.3), 128.5, 128.6, (129.0), 133.3, 135.3, 142.8, 144.7, 162.0, 197.8 (only observed peaks). **HRMS–DART** (*m/z*): $[M+H]^+$ calcd for $C_{16}H_{16}NO_3$; 270.1125, found 270.1125.

Pyrrolidine-1,2-diylbis(phenylmethanone) (8aa-C)

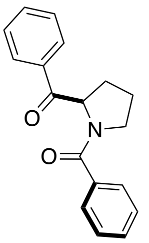

8aa-C

The yield (Table 2, 42%) of **8aa-C** in the crude was determined by ^{1}H-NMR analysis using 1,1,2,2-tetrachloroethane as an internal standard. The product **8aa-C** was purified by flash chromatography on silica gel (100:0–50:50, hexane/EtOAc) (22.1 mg, 0.079 mmol, 40% isolated yield). White Solid. **M.p.** 100–101 °C. **IR** (neat)701, 1224, 1416, 1447, 1600, 1626, 1692, 2876, 2975, 3059 cm^{-1}. Peaks for two rotamers (83:17) were given: **^{1}H NMR** (400 MHz, $CDCl_3$) δ 1.91–2.10 (m, 3H), 2.38 (m, 1H), 3.62 (m, 0.83 × 1H), 3.74 (m, 0.83 × 1H), 3.88–3.92 (m, 0.17 × 2H), 5.27 (m, 0.17 × 1H), 5.71 (dd, J = 8.8, 4.4 Hz, 0.83 × 1H), 7.20–7.23 (m, 0.17 × 3H), 7.30–7.63 (m, 0.83 × 1H + 7H), 8.05–8.07 (m, 0.83 × 2H). Peaks for the minor rotamer were enclosed in parenthesis: **^{13}C NMR** (100.6 MHz, $CDCl_3$) δ (22.4), 25.4, 29.4, (31.1), (46.7), 50.1, 61.3, (64.0), (126.5), 127.3, (128.0), 128.2, (128.3), 128.5, 128.6, (129.4), (129.8), 130.0, 133.3, (133.4), (134.2), 135.3, 136.3, (137.3), 169.2, (170.4), 197.7, (198.3). **HRMS–DART** (*m/z*): $[M+H]^+$ calcd for $C_{18}H_{18}NO_2$; 280.1332, found 280.1333.

10. Effects of Reaction Components on Intermolecular Arylacylation of Styrenes

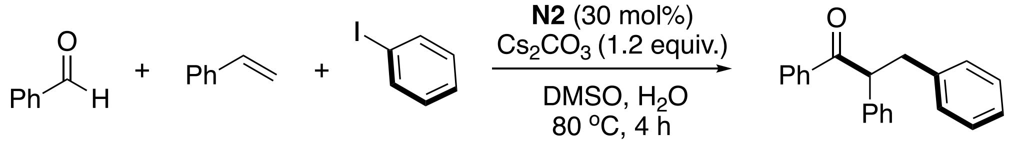

1a (0.2 mmol) **4a** (2.0 mmol) **5a** (0.3 mmol) **6aaa**

entry	changes from standard conditions	^{1}H-NMR yield of **6aaa** (%)
1	none	50 (34)[a]
2	**N1** instead of **N2**	30
3	without H_2O	36
4	Rb_2CO_3 instead of Cs_2CO_3	17
5	TBD instead of Cs_2CO_3	5
6	MTBD instead of Cs_2CO_3	trace

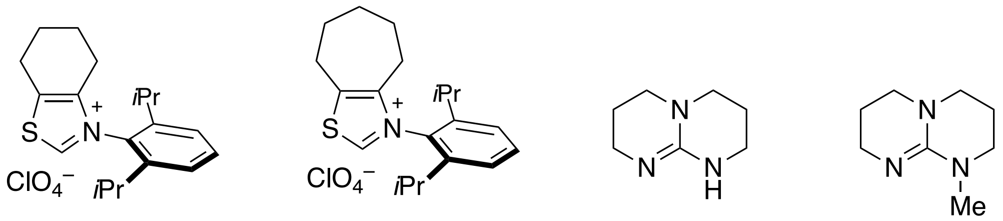

N2 **N1** **TBD** **MTBD**

Supplementary Fig. 1 | Effects of Reaction Components. [a] isolated yield.

11. Effects of Reaction Components on C–H Acylation

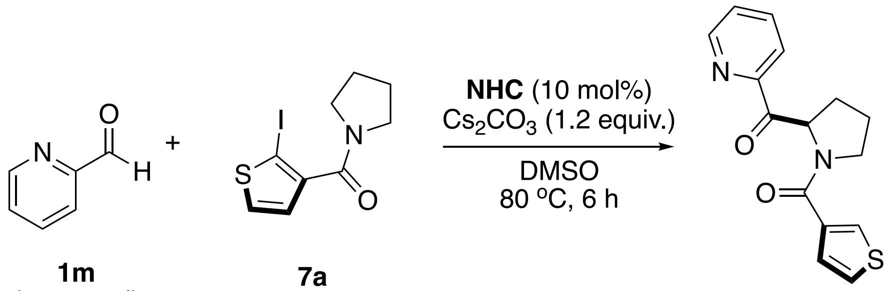

1m (0.4 mmol) **7a** (0.2 mmol) **8ma**

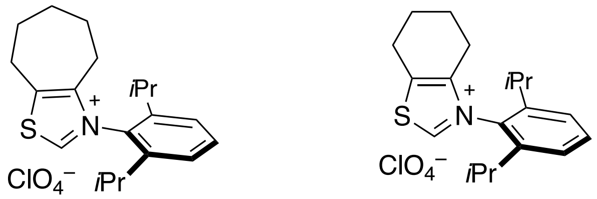

N1
>99% ^{1}H-NMR yield

N2
>99% ^{1}H-NMR yield

Supplementary Fig. 2 | Effects of NHC Catalysts.

12. Cyclic Voltammetry (CV) Experiments

CV measurements were carried out under nitrogen atmosphere in DMSO solutions with 0.1 M of tetrabutylammonium tetrafluoroborate (Bu_4NBF_4) as a supporting electrolyte. Measurements were made with a glassy carbon electrode (area = 0.07 cm^2), an Ag/AgCl reference electrode, and a Pt wire counter electrode. The concentration of the sample solution was fixed at 10 mM and the sweep rates were set to 100 mV/s.

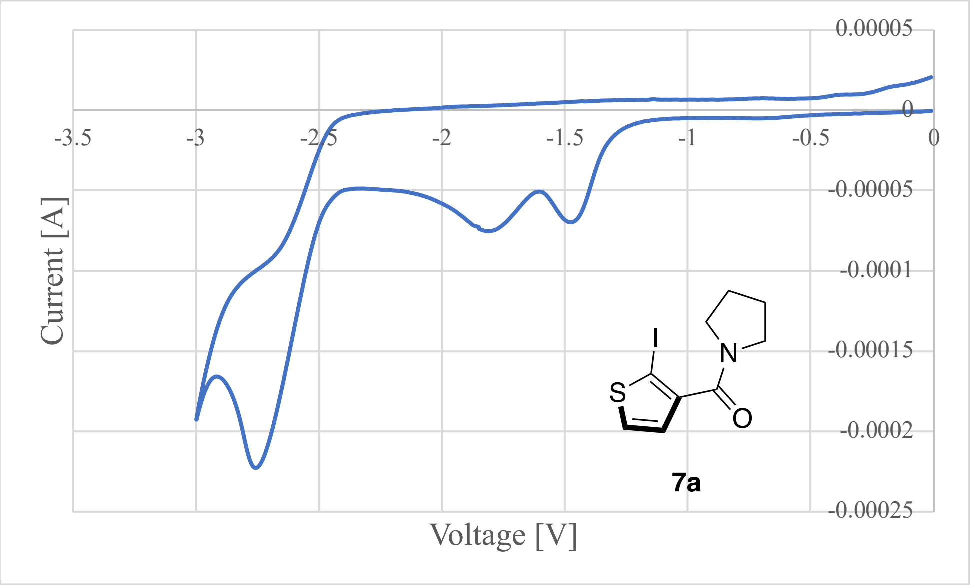

Supplementary Fig. 3 | Cyclic voltammogram of **7a**. The compound is unstable under the measurement conditions.

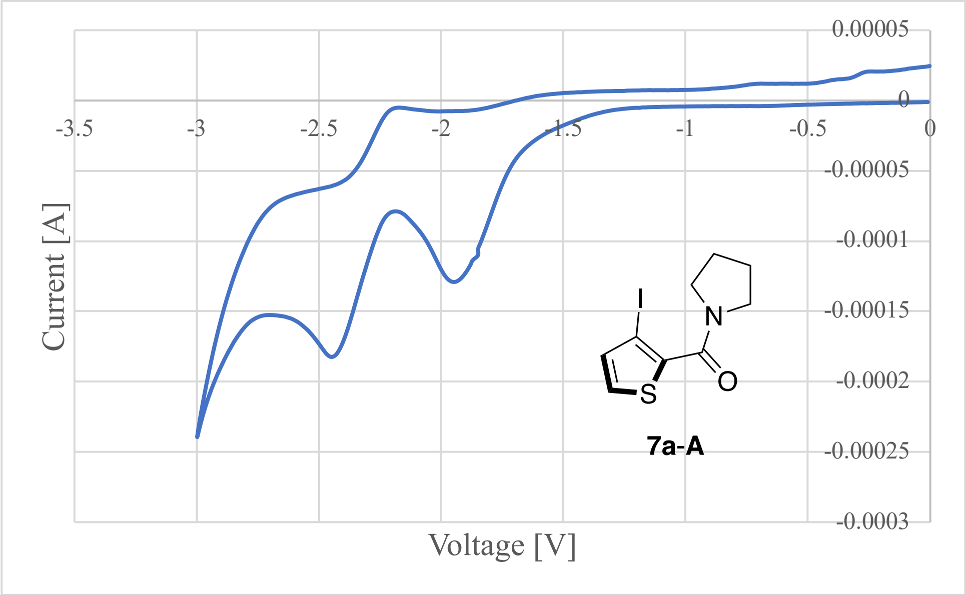

Supplementary Fig. 4 | Cyclic voltammogram of **7a-A**. The compound is unstable under the measurement conditions.

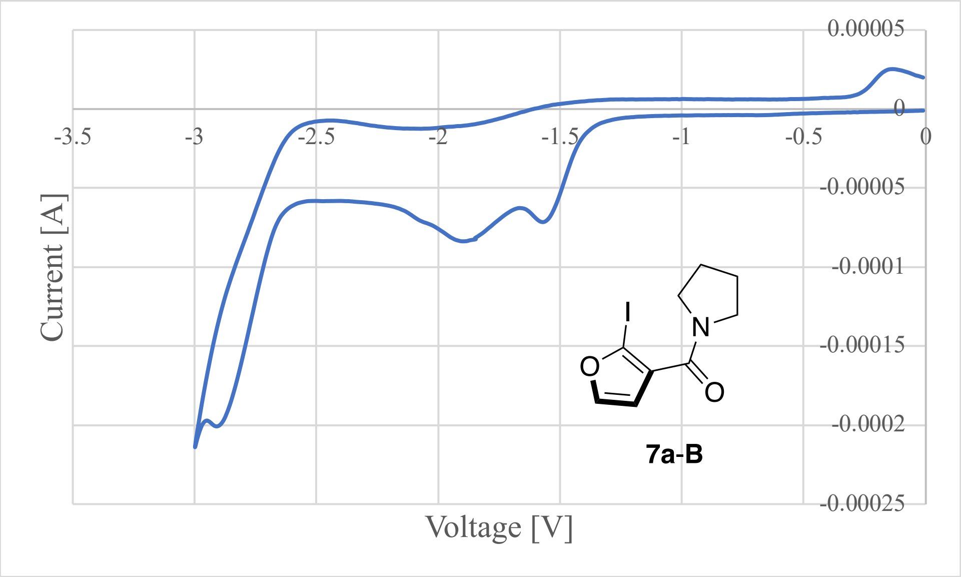

Supplementary Fig. 5 | Cyclic voltammogram of **7a-B**. The compound is unstable under the measurement conditions.

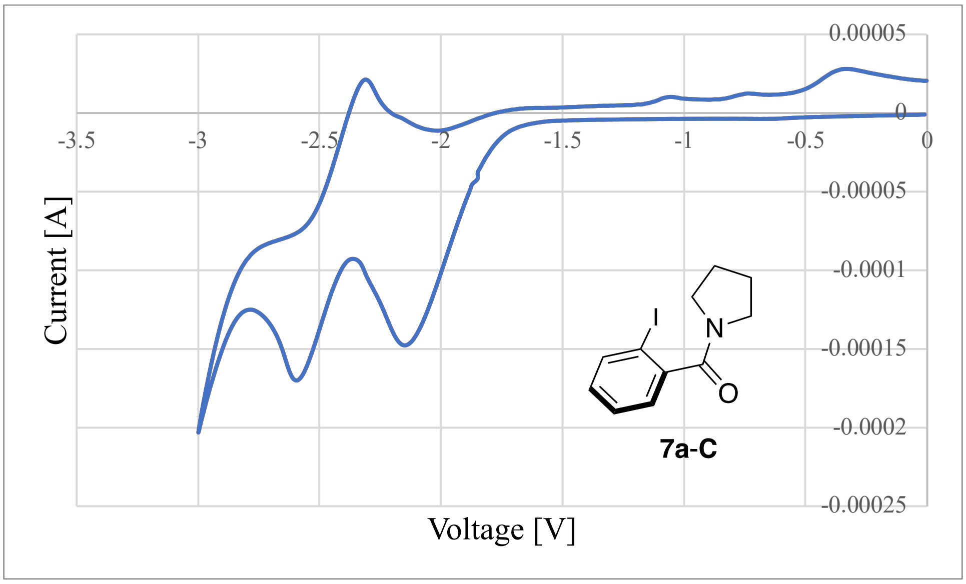

Supplementary Fig. 6 | Cyclic voltammogram of **7a-C**. The compound is unstable under the measurement conditions.

13. Computational Studies

All calculations were carried out with Gaussian 09, Revision D.01.[12]

13.1. Calculation of Bond Dissociaton Energy (BDE)

The BDE values of C–I bonds in amide substrates **7a** and **7a-A–C** were calucated according to the following equation.

$$\mathrm{BDE\ (C–I)} = \Delta H_{\mathrm{Ar\ radical}} + \Delta H_{\mathrm{I\ radical}} - \Delta H_{\mathrm{Ar–I}}$$

The density functional theory (DFT) functional UB3LYP[13] was used with the 6-31+G* basis set (I: LANL2DZ[14]) for geometry optimizations. We carried out vibrational frequency calculations with

6-311+G* to obtain thermal corrections to enthalpies (H) at 298.15 K (25 °C).

amide substrate	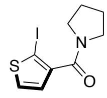 7a	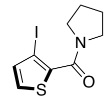 7a-A	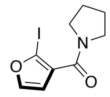 7a-B	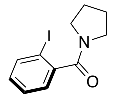 7a-C
$\Delta H_{\text{Ar radical}}$ (Hartree)	–877.009160	–877.014250	–554.017869	–556.217618
$\Delta H_{\text{I radical}}$ (Hartree)	–11.360259	–11.360259	–11.360259	–11.360259
$\Delta H_{\text{Ar–I}}$ (Hartree)	–888.468662	–888.470684	–565.480514	–567.671583
BDE (C–I) (kcal/mol)	62.3	60.4	64.2	58.8

Supplementary Fig. 7 | BDEs of C–I bonds

Optimization structure of I radical

I	-0.25990099	-0.24752475	0.00000000

Optimization structure of 7a Ar radical

C	3.37835900	0.52139100	-0.02334000
C	2.21890000	1.23956400	-0.05559400
C	1.02379000	0.42330400	-0.01305200
C	1.38888100	-0.88758200	0.05105000
S	3.07210200	-1.20716200	0.06335600
H	4.39834200	0.88421200	-0.04455800
H	2.15983500	2.32108200	-0.10665500
C	-0.33137100	1.05477100	-0.02747100
O	-0.43467600	2.28701600	-0.05265700
C	-1.48664500	-1.21646700	-0.01375700
C	-2.77230700	0.86416500	0.01777600
C	-2.96556100	-1.51624900	-0.31393300
H	-1.18821500	-1.62270500	0.96328700
H	-0.81452100	-1.63290900	-0.77224600
C	-3.70303000	-0.32048100	0.31201100
H	-2.98780600	1.32850400	-0.95412800
H	-2.80960300	1.65524900	0.77169300
H	-3.12832000	-1.53551600	-1.39878400
H	-3.27993200	-2.48350800	0.09061300
H	-4.70682400	-0.17266500	-0.09840300
H	-3.80163700	-0.46308000	1.39566300
N	-1.43194900	0.25218800	-0.00586500

Optimization structure of 7a

C	-1.86078700	2.84512500	0.41996600
C	-0.60802000	2.70798200	-0.10891500
C	-0.22142800	1.33978300	-0.31293700
C	-1.21996500	0.47324700	0.05903600
S	-2.62413500	1.30601100	0.66264200
H	-2.38424000	3.75748500	0.67597400
H	0.03754100	3.54175700	-0.36316700
C	1.09042600	0.98848900	-0.96453000
O	1.29849500	1.29663500	-2.14118000
C	1.97369300	0.11885300	1.25245300
C	3.33115200	0.01184500	-0.78357600
C	3.45478900	-0.04554800	1.62725500
H	1.40960700	-0.79705400	1.47211900
H	1.48708400	0.94979700	1.77034600
C	4.06318900	-0.69170900	0.36963700
H	3.84591900	0.92618200	-1.10677200
H	3.19578600	-0.61604100	-1.66932000
H	3.90874000	0.93760800	1.80526800
H	3.58801300	-0.64710900	2.53210100
H	5.14905200	-0.57055800	0.30561900
H	3.84536100	-1.76710800	0.35667000
N	2.02270500	0.36114900	-0.20151200
I	-1.25777200	-1.62265400	-0.13568700

Optimization structure of 7a-A Ar radical

C	-3.49808100	0.59544100	0.02914200
C	-1.06490600	-0.02828300	0.00358700
C	-1.33798200	1.30029800	0.07173900
H	-4.58160600	0.57108800	0.02443800
C	0.16834900	-0.85403200	-0.02273900
O	0.08298600	-2.08870900	-0.05191800
C	1.63336400	1.22851500	-0.01309400
C	2.61130600	-1.01552300	0.00989600
C	3.14017100	1.31548600	-0.30966600
H	1.39154800	1.67007000	0.96411400
H	1.02596400	1.73516500	-0.77025800
C	3.70001400	0.02390500	0.31050000
H	2.75835300	-1.49845400	-0.96573400

H	2.53607500	-1.80902500	0.75838000
H	3.30655700	1.31704200	-1.39416600
H	3.58878700	2.22585000	0.10034000
H	4.67308300	-0.26288600	-0.10031500
H	3.81643600	0.14526800	1.39494700
N	1.37207300	-0.21852500	-0.00736700
S	-2.60726900	-0.88948600	-0.04355500
C	-2.68958300	1.70846400	0.08910700
H	-3.04320100	2.73138900	0.14121700

Optimization structure of 7a-A

C	2.23936800	2.49976500	-0.72723800
C	0.28851200	1.24664700	0.21338100
C	1.33577700	0.44220000	-0.15937700
H	2.90535100	3.27261200	-1.08949400
C	-1.00420500	0.92543800	0.91112000
O	-1.20134700	1.30748100	2.06753800
C	-1.90364200	-0.09842300	-1.23327900
C	-3.21181500	-0.13072200	0.83790900
C	-3.38656800	-0.33129500	-1.56190000
H	-1.31385400	-1.00637700	-1.41317900
H	-1.45653200	0.71752900	-1.80766100
C	-3.94497800	-0.92305900	-0.25525100
H	-3.74834800	0.78477900	1.11947500
H	-3.03585600	-0.70086200	1.75500200
H	-3.87691600	0.62474100	-1.78500300
H	-3.51927800	-0.98839800	-2.42727600
H	-5.03251800	-0.83290600	-0.17203200
H	-3.69110100	-1.98828300	-0.18597500
N	-1.92857800	0.22533300	0.20550900
I	1.40123800	-1.66048100	0.12052200
S	0.68986400	2.92111700	-0.08030300
C	2.45589600	1.14921000	-0.69846100
H	3.36633700	0.66906800	-1.03698700

Optimization structure of 7a-B Ar radical

C	3.58450500	-0.14233500	0.02015900
C	2.65096800	0.84038300	-0.02901300
C	1.34594600	0.20522400	-0.00775500

C	1.65226400	-1.12557900	0.05391100
H	4.66265800	-0.17423200	0.02648500
H	2.81170500	1.90854500	-0.07500800
C	0.06497800	0.95311600	-0.03676100
O	0.07970700	2.18993400	-0.07318800
C	-1.28110900	-1.20527600	-0.00079500
C	-2.38706300	0.97720100	-0.00515300
C	-2.78136800	-1.38554900	-0.28848900
H	-1.00996700	-1.62002900	0.98093400
H	-0.65083000	-1.68755300	-0.75639500
C	-3.41283000	-0.11908900	0.31490700
H	-2.56593700	1.43703400	-0.98667800
H	-2.35573800	1.78422100	0.73189300
H	-2.95149700	-1.41339900	-1.37201000
H	-3.17432800	-2.31416200	0.13730400
H	-4.40241400	0.10406000	-0.09595900
H	-3.51852600	-0.23105400	1.40147800
N	-1.10411600	0.25349700	-0.01633400
O	2.95353500	-1.38449500	0.07271700

Optimization structure of 7a-B

C	1.76401900	2.76202800	-0.77612700
C	0.49467700	2.83063100	-0.29771700
C	0.12101300	1.49146700	0.08436400
C	1.22060700	0.72230000	-0.18552000
H	2.45684600	3.48231600	-1.18324200
H	-0.12591900	3.71129700	-0.20635800
C	-1.16414600	1.14139300	0.76775100
O	-1.48066100	1.71820800	1.81281100
C	-1.84994200	-0.36036800	-1.16700700
C	-3.24322800	-0.14695100	0.83389000
C	-3.29506500	-0.76400300	-1.49818300
H	-1.19050300	-1.23774700	-1.17057400
H	-1.43686000	0.37754700	-1.86000700
C	-3.86322200	-1.17225000	-0.12745600
H	-3.86020200	0.75550800	0.93380300
H	-3.06981000	-0.53541700	1.84200100
H	-3.84526400	0.09880000	-1.89465800
H	-3.34032400	-1.56463200	-2.24346200
H	-4.95712900	-1.16163900	-0.09370300

H	-3.53050600	-2.18554300	0.13100200
N	-1.96148600	0.20109200	0.19271700
I	1.68949700	-1.27759000	0.18366600
O	2.22796900	1.47412600	-0.70557100

Optimization structure of 7a-C Ar radical

C	2.25201000	1.12162400	-0.16420800
C	1.10967200	0.30989900	0.01355600
H	2.09557300	2.18845700	-0.29694800
C	-0.23567700	0.99144100	0.02442800
O	-0.29933100	2.22661300	0.03729000
C	-1.51720400	-1.22349900	-0.09303200
C	-2.67461500	0.92292700	0.09165900
C	-3.00678500	-1.40365200	-0.43255100
H	-1.26406000	-1.72165900	0.85291400
H	-0.85913900	-1.62271700	-0.87024200
C	-3.67821900	-0.22135900	0.28466100
H	-2.84911400	1.47339100	-0.84267200
H	-2.67494100	1.65527300	0.90371000
H	-3.15436700	-1.32147200	-1.51680600
H	-3.38565000	-2.38028600	-0.11460700
H	-4.66682300	0.02114900	-0.11764900
H	-3.79661100	-0.44520900	1.35256800
N	-1.37041300	0.23683100	0.03112100
C	1.36203400	-1.03590400	0.19739500
C	2.59831200	-1.64472800	0.21614900
C	3.53043300	0.56513500	-0.16086800
C	3.71302600	-0.81092800	0.02841400
H	2.71649600	-2.71438000	0.37044600
H	4.71371800	-1.23754100	0.03321900
H	4.39452900	1.20853900	-0.30371400

Optimization structure of 7a-C

C	-0.65380300	2.65263700	-0.33605500
C	-0.40333200	1.27142300	-0.38956300
H	0.09313400	3.33341000	-0.73646000
C	0.88007000	0.81319000	-1.04931700
O	0.98051200	0.81152600	-2.27850800
C	1.92069100	0.48736100	1.24147300

C	3.17887400	-0.01239300	-0.80100100
C	3.41794700	0.38737700	1.57133500
H	1.36894800	-0.37027300	1.65001700
H	1.45727500	1.40231200	1.62075500
C	3.97070200	-0.48413100	0.42901700
H	3.67706500	0.81399200	-1.32487400
H	2.99393200	-0.80128700	-1.53612400
H	3.87554900	1.38445300	1.54335300
H	3.59494200	-0.03673500	2.56485600
H	5.05140600	-0.37824200	0.29190300
H	3.76109000	-1.54228500	0.62934500
N	1.90368000	0.45842200	-0.23196000
C	-1.38555400	0.40023100	0.09446900
C	-2.58098700	0.88620500	0.63201200
C	-1.83895500	3.15015500	0.20397300
C	-2.80305900	2.26397600	0.69174500
H	-3.33565700	0.19684800	0.99612100
H	-3.73348600	2.63795600	1.11090100
H	-2.01189000	4.22236700	0.23703200
I	-1.13542900	-1.72565800	-0.01742600

13.2. Calculation of Gibbs Energy Barrier on α-Amino C(sp^3)–H Abstraction Step ($\Delta G^{\ddagger}_{C\text{–}H}$)

The density functional theory (DFT) functional UB3LYP was used with the 6-31+G* basis set for geometry optimizations. We carried out vibrational frequency calculations to obtain thermal corrections to enthalpies (H) at 298.15 K (25 °C). The solvation effect (DMSO) was evaluated with a conductor-like polarizable continuum model (CPCM).

Gibbs Free Energy	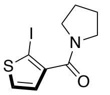 7a	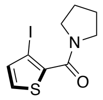 7a-A	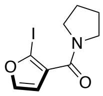 7a-B	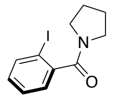 7a-C
**Int** (Hartree)	–877.068852	–877.074474	–554.076419	–556.277384
**TS** (Hartree)	–877.062170	–877.062470	–554.066718	–556.265990
**Product** (Hartree)	–877.109496	–877.110386	–554.120687	–556.310006
$\Delta G^{\ddagger}_{C\text{–}H}$ (kcal/mol)	4.2	7.5	6.1	7.2

Supplementary Fig. 8. | Gibbs Energy Barrier on α-Amino C(sp^3)–H Abstraction Step

Optimization structure of Int-7a

C	3.37835900	0.52139100	-0.02334000
C	2.21890000	1.23956400	-0.05559400

C	1.02379000	0.42330400	-0.01305200
C	1.38888100	-0.88758200	0.05105000
S	3.07210200	-1.20716200	0.06335600
H	4.39834200	0.88421200	-0.04455800
H	2.15983500	2.32108200	-0.10665500
C	-0.33137100	1.05477100	-0.02747100
O	-0.43467600	2.28701600	-0.05265700
C	-1.48664500	-1.21646700	-0.01375700
C	-2.77230700	0.86416500	0.01777600
C	-2.96556100	-1.51624900	-0.31393300
H	-1.18821500	-1.62270500	0.96328700
H	-0.81452100	-1.63290900	-0.77224600
C	-3.70303000	-0.32048100	0.31201100
H	-2.98780600	1.32850400	-0.95412800
H	-2.80960300	1.65524900	0.77169300
H	-3.12832000	-1.53551600	-1.39878400
H	-3.27993200	-2.48350800	0.09061300
H	-4.70682400	-0.17266500	-0.09840300
H	-3.80163700	-0.46308000	1.39566300
N	-1.43194900	0.25218800	-0.00586500

Optimization structure of TS-7a

C	3.31467100	0.40436000	-0.06239500
C	2.24770900	1.25368400	0.02152100
C	0.99300400	0.55404800	0.07797700
C	1.15397100	-0.80585000	-0.00386600
S	2.80846500	-1.27529100	-0.09390800
H	4.36655800	0.65339100	-0.13106900
H	2.32105600	2.33530100	0.04034900
C	-0.33880800	1.21321200	0.10807700
O	-0.49162300	2.42048400	-0.07732700
C	-1.32678800	-1.05438200	0.48887300
C	-2.76060200	0.77762800	-0.18447300
C	-2.52370400	-1.64701700	-0.24234800
H	-1.24339600	-1.32687800	1.54969300
H	-0.18716200	-1.37589900	0.07858100
C	-3.58027000	-0.52544000	-0.11017100
H	-2.69156200	1.17637900	-1.20430600
H	-3.15470000	1.57385700	0.45392500
H	-2.27432500	-1.81201400	-1.29874000

H	-2.84919500	-2.60134500	0.18198200
H	-4.34834500	-0.57258900	-0.88828100
H	-4.08381300	-0.60143800	0.86098800
N	-1.41895300	0.36722200	0.27412200

Optimization structure of Product-7a

C	3.31113300	0.51709800	0.44635600
C	2.11821700	1.17953300	0.47825200
C	1.02063900	0.38579000	-0.00324200
C	1.43215300	-0.86744500	-0.40359300
S	3.13509600	-1.08780700	-0.19755200
H	4.28407000	0.87268900	0.75963200
H	1.99183800	2.20017500	0.81913700
H	0.84308500	-1.65519800	-0.85216900
C	-0.33365500	0.99484000	-0.12742100
O	-0.45380500	2.21762600	-0.28418900
C	-1.58348500	-1.15769500	0.23194900
C	-2.78433100	0.79404400	-0.34142700
C	-3.02471400	-1.56620700	0.19080600
C	-3.77014800	-0.21174100	0.27166400
H	-2.91032200	0.89816400	-1.42670200
H	-2.82488800	1.79151700	0.09744500
H	-3.27236900	-2.08833300	-0.74905200
H	-3.29149000	-2.24919800	1.00618100
H	-4.73157700	-0.21641100	-0.25062100
H	-3.95688300	0.04594500	1.32064600
N	-1.45725100	0.19090200	-0.06774000
H	-0.77665600	-1.68106500	0.72395900

Optimization structure of Int-7a-A

C	-3.49808100	0.59544100	0.02914200
C	-1.06490600	-0.02828300	0.00358700
C	-1.33798200	1.30029800	0.07173900
H	-4.58160600	0.57108800	0.02443800
C	0.16834900	-0.85403200	-0.02273900
O	0.08298600	-2.08870900	-0.05191800
C	1.63336400	1.22851500	-0.01309400
C	2.61130600	-1.01552300	0.00989600
C	3.14017100	1.31548600	-0.30966600

H	1.39154800	1.67007000	0.96411400
H	1.02596400	1.73516500	-0.77025800
C	3.70001400	0.02390500	0.31050000
H	2.75835300	-1.49845400	-0.96573400
H	2.53607500	-1.80902500	0.75838000
H	3.30655700	1.31704200	-1.39416600
H	3.58878700	2.22585000	0.10034000
H	4.67308300	-0.26288600	-0.10031500
H	3.81643600	0.14526800	1.39494700
N	1.37207300	-0.21852500	-0.00736700
S	-2.60726900	-0.88948600	-0.04355500
C	-2.68958300	1.70846400	0.08910700
H	-3.04320100	2.73138900	0.14121700

Optimization structure of TS-7a-A

C	3.30234100	-0.78991500	-0.12731600
C	1.03069000	0.18062000	0.08642900
C	1.04143700	-1.18413800	0.00956900
H	4.37378500	-0.92939600	-0.21305000
C	-0.16812600	1.04294000	0.12233900
O	-0.13437800	2.26177400	-0.05211500
C	-1.47150500	-1.06655100	0.48274200
C	-2.62496400	0.96469100	-0.16623900
C	-2.72906300	-1.46950600	-0.27230300
H	-1.44589400	-1.35811700	1.54139300
H	-0.34984500	-1.53735500	0.08594200
C	-3.61959900	-0.21269400	-0.12780800
H	-2.49395600	1.37518900	-1.17515900
H	-2.90957700	1.79175600	0.49105900
H	-2.49292000	-1.64776600	-1.32986000
H	-3.19240900	-2.37587800	0.12867400
H	-4.37653100	-0.13565800	-0.91458600
H	-4.14159500	-0.23638900	0.83622800
N	-1.35623300	0.35908900	0.28526000
S	2.65661300	0.81741500	-0.01948000
C	2.33098600	-1.76620200	-0.09943900
H	2.53860000	-2.82830600	-0.16669500

Optimization structure of Product-7a-A

C	3.50216100	0.50372900	-0.12748500
C	1.07543700	-0.01692000	0.08346500
C	1.37942200	1.29579600	0.39247100
H	4.56914000	0.45412000	-0.30511900
C	-0.17260200	-0.80563100	0.15108300
O	-0.13315600	-2.03987500	0.27041400
C	-1.69531900	1.16108800	-0.22004600
C	-2.62985200	-0.93578300	0.33089000
C	-3.17704500	1.37767400	-0.18201500
H	-0.96029500	1.79482400	-0.69328900
H	0.64633000	2.01613500	0.73465900
C	-3.73731800	-0.06232300	-0.27746700
H	-2.74723000	-1.07155600	1.41349700
H	-2.53217400	-1.92271100	-0.12224800
H	-3.49335800	1.85489800	0.76115200
H	-3.52765000	2.02697600	-0.99302500
H	-4.69210600	-0.18882400	0.24156900
H	-3.88613900	-0.33263800	-1.32923700
N	-1.39287200	-0.15553400	0.07785400
S	2.52344400	-0.90304800	-0.33989600
C	2.76645900	1.59205500	0.27301600
H	3.19567900	2.56716400	0.47861300

Optimization structure of Int-7a-B

C	3.58450500	-0.14233500	0.02015900
C	2.65096800	0.84038300	-0.02901300
C	1.34594600	0.20522400	-0.00775500
C	1.65226400	-1.12557900	0.05391100
H	4.66265800	-0.17423200	0.02648500
H	2.81170500	1.90854500	-0.07500800
C	0.06497800	0.95311600	-0.03676100
O	0.07970700	2.18993400	-0.07318800
C	-1.28110900	-1.20527600	-0.00079500
C	-2.38706300	0.97720100	-0.00515300
C	-2.78136800	-1.38554900	-0.28848900
H	-1.00996700	-1.62002900	0.98093400
H	-0.65083000	-1.68755300	-0.75639500
C	-3.41283000	-0.11908900	0.31490700
H	-2.56593700	1.43703400	-0.98667800
H	-2.35573800	1.78422100	0.73189300

H	-2.95149700	-1.41339900	-1.37201000
H	-3.17432800	-2.31416200	0.13730400
H	-4.40241400	0.10406000	-0.09595900
H	-3.51852600	-0.23105400	1.40147800
N	-1.10411600	0.25349700	-0.01633400
O	2.95353500	-1.38449500	0.07271700

Optimization structure of TS-7a-B

C	3.43404500	-0.33453100	-0.11286800
C	2.68325600	0.79201500	0.01085100
C	1.31419000	0.34894200	0.08308100
C	1.35511600	-1.01512300	-0.03952500
H	4.49178000	-0.52721100	-0.21060100
H	3.03492600	1.81380100	0.04550800
C	0.06683200	1.13231800	0.12158500
O	0.01766800	2.35117200	-0.03881100
C	-1.12056200	-1.04816700	0.47713000
C	-2.38227400	0.90060400	-0.21281000
C	-2.38412100	-1.53383500	-0.21908200
H	-1.02301100	-1.32827200	1.53456700
H	-0.01223200	-1.48995600	0.03565300
C	-3.32447500	-0.31216800	-0.09882900
H	-2.27842800	1.25888600	-1.24517700
H	-2.69238900	1.75100500	0.40099900
H	-2.17200800	-1.74676500	-1.27518200
H	-2.79296500	-2.44241100	0.23255300
H	-4.10485400	-0.30088500	-0.86592800
H	-3.81789000	-0.31694900	0.88045600
N	-1.08725300	0.37382500	0.25979700
O	2.62305600	-1.45971400	-0.13726700

Optimization structure of Product-7a-B

C	-3.55794200	-0.07563100	-0.22272300
C	-2.57410900	0.84987300	-0.34876100
C	-1.34090600	0.18852200	0.00271600
C	-1.69381000	-1.09832400	0.32549500
H	-4.62574600	-0.06327500	-0.37886700
H	-2.67061300	1.88465200	-0.64374400
C	-0.04997100	0.91213700	0.07175900

O	-0.03118000	2.14789800	0.15273600
C	1.36308500	-1.15246000	-0.14448800
C	2.41139300	0.92454700	0.27010500
C	2.83137800	-1.44630600	-0.07239500
H	0.60886700	-1.76383900	-0.61921000
H	-1.15144700	-1.95359100	0.69526100
C	3.47314700	-0.04993600	-0.26189500
H	2.52437700	1.12557900	1.34307100
H	2.37801300	1.88354200	-0.24817000
H	3.11117100	-1.87072800	0.90653000
H	3.15340600	-2.17029900	-0.83018500
H	4.42957200	0.05927900	0.25812500
H	3.64527100	0.13664000	-1.32829600
N	1.13637400	0.20126300	0.04768500
O	-3.03520600	-1.27181500	0.19636500

Optimization structure of Int-7a-C

C	2.25201000	1.12162400	-0.16420800
C	1.10967200	0.30989900	0.01355600
H	2.09557300	2.18845700	-0.29694800
C	-0.23567700	0.99144100	0.02442800
O	-0.29933100	2.22661300	0.03729000
C	-1.51720400	-1.22349900	-0.09303200
C	-2.67461500	0.92292700	0.09165900
C	-3.00678500	-1.40365200	-0.43255100
H	-1.26406000	-1.72165900	0.85291400
H	-0.85913900	-1.62271700	-0.87024200
C	-3.67821900	-0.22135900	0.28466100
H	-2.84911400	1.47339100	-0.84267200
H	-2.67494100	1.65527300	0.90371000
H	-3.15436700	-1.32147200	-1.51680600
H	-3.38565000	-2.38028600	-0.11460700
H	-4.66682300	0.02114900	-0.11764900
H	-3.79661100	-0.44520900	1.35256800
N	-1.37041300	0.23683100	0.03112100
C	1.36203400	-1.03590400	0.19739500
C	2.59831200	-1.64472800	0.21614900
C	3.53043300	0.56513500	-0.16086800
C	3.71302600	-0.81092800	0.02841400
H	2.71649600	-2.71438000	0.37044600

H	4.71371800	-1.23754100	0.03321900
H	4.39452900	1.20853900	-0.30371400

Optimization structure of TS-7a-C

C	2.26558200	1.14669400	0.02460000
C	1.06707700	0.41475400	0.06761100
H	2.21741800	2.23160600	0.05190100
C	-0.25147000	1.13590000	0.09368200
O	-0.33739200	2.34971600	-0.10310300
C	-1.37584100	-1.07195600	0.48469900
C	-2.69832000	0.86563300	-0.12257100
C	-2.58839200	-1.56985000	-0.28587800
H	-1.35564700	-1.35166100	1.54699800
H	-0.20714800	-1.42900500	0.10171000
C	-3.58568000	-0.39736600	-0.12153500
H	-2.62338000	1.33276700	-1.11167300
H	-3.04373500	1.63350100	0.57687900
H	-2.33318100	-1.70643200	-1.34527900
H	-2.97408900	-2.51998800	0.09586200
H	-4.34054000	-0.36749200	-0.91366000
H	-4.11218700	-0.49075800	0.83576200
N	-1.36938100	0.35937100	0.27564800
C	1.13535300	-0.97221900	0.00228300
C	2.32992100	-1.67130700	-0.07798500
C	3.48346100	0.47025100	-0.05393000
C	3.52078400	-0.93003100	-0.10601900
H	2.35381300	-2.75751700	-0.12550300
H	4.47650700	-1.44591400	-0.17021600
H	4.41212300	1.03455000	-0.07724200

Optimization structure of Product-7a-C

C	2.09305400	1.03723200	0.57767200
C	1.09319900	0.30989100	-0.08767000
H	1.84468300	2.00987100	0.99143500
C	-0.25204400	0.95696100	-0.23630800
O	-0.34986300	2.16694400	-0.47933900
C	-1.53047500	-1.12776300	0.34205200
C	-2.71350000	0.78780200	-0.36579300
C	-2.97452100	-1.51883200	0.36277500

H	-0.69351300	-1.66400200	0.76217200
H	0.66842300	-1.48430300	-1.21834700
C	-3.69679900	-0.14953200	0.35252200
H	-2.87333700	0.81234200	-1.45122600
H	-2.72323300	1.81633200	-0.00296900
H	-3.25322100	-2.11009100	-0.52720500
H	-3.23340600	-2.13036400	1.23560800
H	-4.67206600	-0.17987300	-0.14259900
H	-3.85123100	0.19442400	1.38176200
N	-1.38778600	0.18263000	-0.09117900
C	1.42055400	-0.92498400	-0.66931200
C	2.72034900	-1.42811800	-0.57375600
C	3.38434000	0.52261900	0.69231900
C	3.70209500	-0.71206300	0.11667600
H	2.96593600	-2.37778700	-1.04248800
H	4.71085000	-1.10902800	0.19784800
H	4.14601700	1.08923600	1.22191600

14. Deprotection of Directing Group

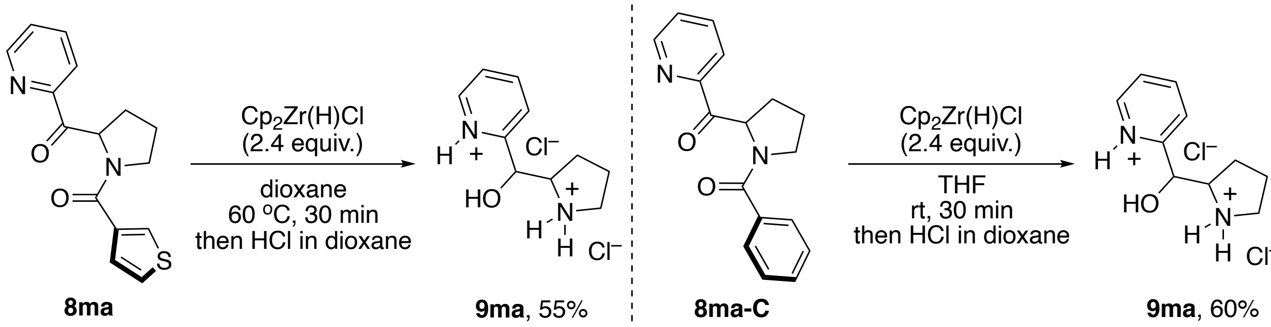

Supplementary Fig. 9 | Deprotection of Directing Group

Deprotection of 8ma. The deprotection is conducted by the reported procedure.[15] $Cp_2Zr(H)Cl$ (61.9 mg, 0.24 mmol) was suspended in dioxane (830 μL) under nitrogen at room temperature in a flame-dried round bottom flask. To this suspension was added **8ma** (28.6 mg, 0.1 mmol) in dioxane (500 μL) also at room temperature. After stirring at 60 °C for 30 min, ethanol was added to the reaction mixture, stirring for 5 min. After filtration with Celite, the filtrate solution was concentrated. The volaties were dissolved in DCM and acidified to pH 1–2 with 10% HCl aqueous solution. The water layer was washed with DCM (twice) and then basified to pH 9–10 with NaOH aqueous solution. The water layer was extracted with DCM (3 times) and the combined organic layer was dried over sodium sulfate. After filtration, the resulting solution was evaporated under reduced pressure. After the volatiles were treated with HCl in dioxane, the product **9ma** (13.7 mg, 0.055 mmol, 55% isolated yield) was purified by recrystallization from AcEt/EtOH.

Deprotection of 8ma-C. The deprotection is conducted by the reported procedure.[15] $Cp_2Zr(H)Cl$ (61.9 mg, 0.24 mmol) was suspended in THF (830 μL) under nitrogen at room temperature in a flame-dried round bottom flask. To this suspension was added **8ma-C** (28.0 mg, 0.1 mmol) in THF (500 μL) also at room temperature. After stirring for 30 min, ethanol was added to the reaction mixture, stirring for 5 min. After filtration with Celite, the filtrate solution was concentrated. The volaties were dissolved in DCM and acidified to pH 1–2 with 10% HCl aqueous solution. The water layer was washed with DCM (twice) and then basified to pH 9–10 with NaOH aqueous solution. The water layer was extracted with DCM (3 times) and the combined organic layer was dried over sodium sulfate. After filtration, the resulting solution was evaporated under reduced pressure. After the volatiles were treated with HCl in dioxane, the product **9ma** (15.0 mg, 0.060 mmol, 60%) was purified by recrystallization from AcEt/EtOH.

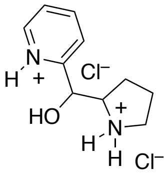

9ma

Off-white solid. **M.p.** 194–197 °C. **IR** (neat) 772, 972, 1087, 1120, 1457, 1612, 2070, 2216, 2476, 3356 cm^{-1}. **^{1}H NMR** (400 MHz, CD_3OD) δ 1.72–2.14 (m, 4H), 3.40–3.41 (m, 2H), 4.17 (m, 1H), 5.68 (m, 1H), 8.09 (m, 1H), 8.24 (m, 1H), 8.67 (m, 1H), 8.86 (m, 1H). **^{13}C NMR** (100.6 MHz, CD_3OD) δ 24.1, 24.8, 47.6, 63.8, 68.5, 126.4, 128.1, 143.3, 148.4, 155.8. **HRMS–ESI** (*m/z*): $[M+H–2HCl]^+$ calcd for $C_{10}H_{15}N_2O$, 179.1179; found, 179.1183.

15. UV-Vis Absorption Measurement

Procedure for Supplementary Fig. 10.

Preparation of 0.8 mM DMSO solution of **10a** (solution A).

Under nitrogen atmosphere, thiazolium salt **10a** (3.8 mg, 8 μmol) was dissolved in degassed DMSO (1 mL). Then, 200 μL of the solution added to the the quartz cuvette sealed with a silicon-based septum and diluted with degassed DMSO (1.8 mL) to make 0.8 mM solution of **10a**.

Preparation of 0.32 M DMSO solution of DBU (solution B).

Under nitrogen atmosphere, DBU (4.8 μL, 32 μmol) was dissolved in degassed DMSO (100 μL).

Preparation of 0.16 M DMSO solution of iodobenzene (solution C).

Under nitrogen atmosphere, iodobenzene (3.6 μL, 32 μmol) was dissolved in degassed DMSO (200 μL).

First, the solution A including thiazolium salt **10a**[16] was measured (Supplementary Fig. 10, yellow line). Then, TBAF (1.6 μL, 1.6 μmol, 1.0 M THF solution) was added to the solution A, and the resuting solution was measured (Supplementary Fig. 10, green line). Subsequently, 10 μl of solution B including DBU was added: the UV-Vis spectrum showed an absorption at λ_{max} = 380 nm, consistent with the formation of the enolate form of Breslow intermediate **10a-B** (Supplementary Fig. 10, red line)[17,18] Finally, 10 μl of solution C including iodobenzene was added: the significant shift of the absorption was not observed (Supplementary Fig. 10, blue line).

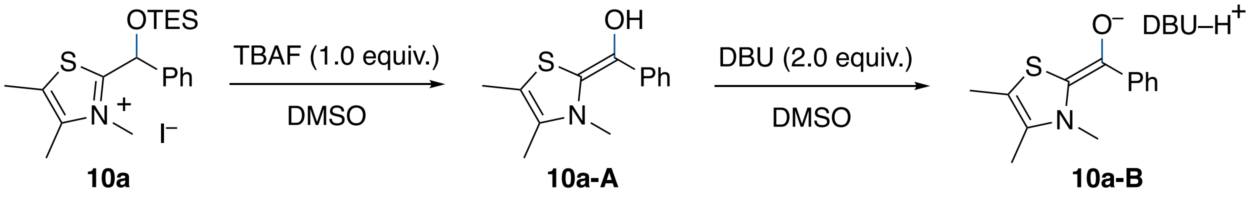

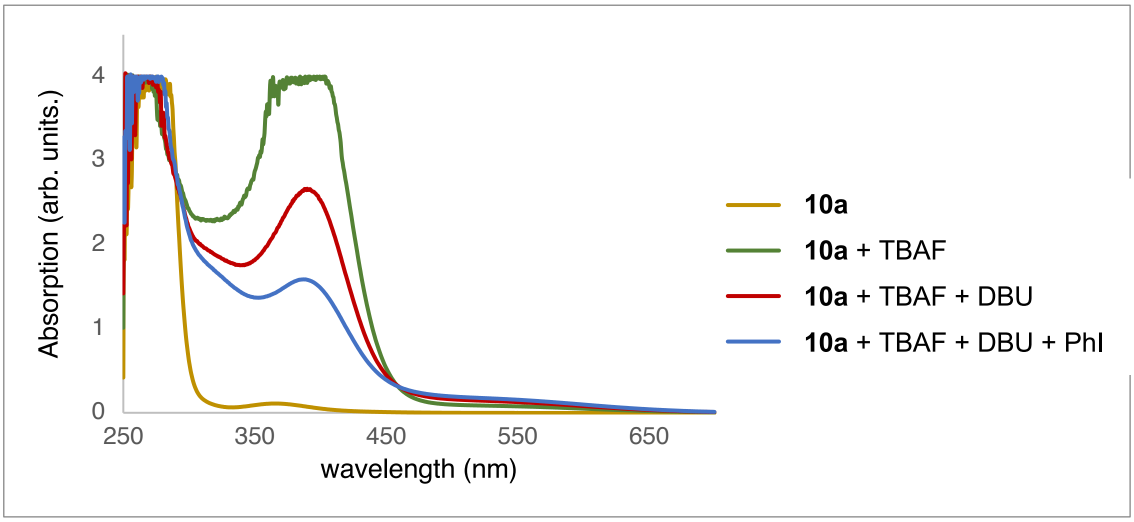

Supplementary Fig. 10 | UV-Vis spectrum of **10a**

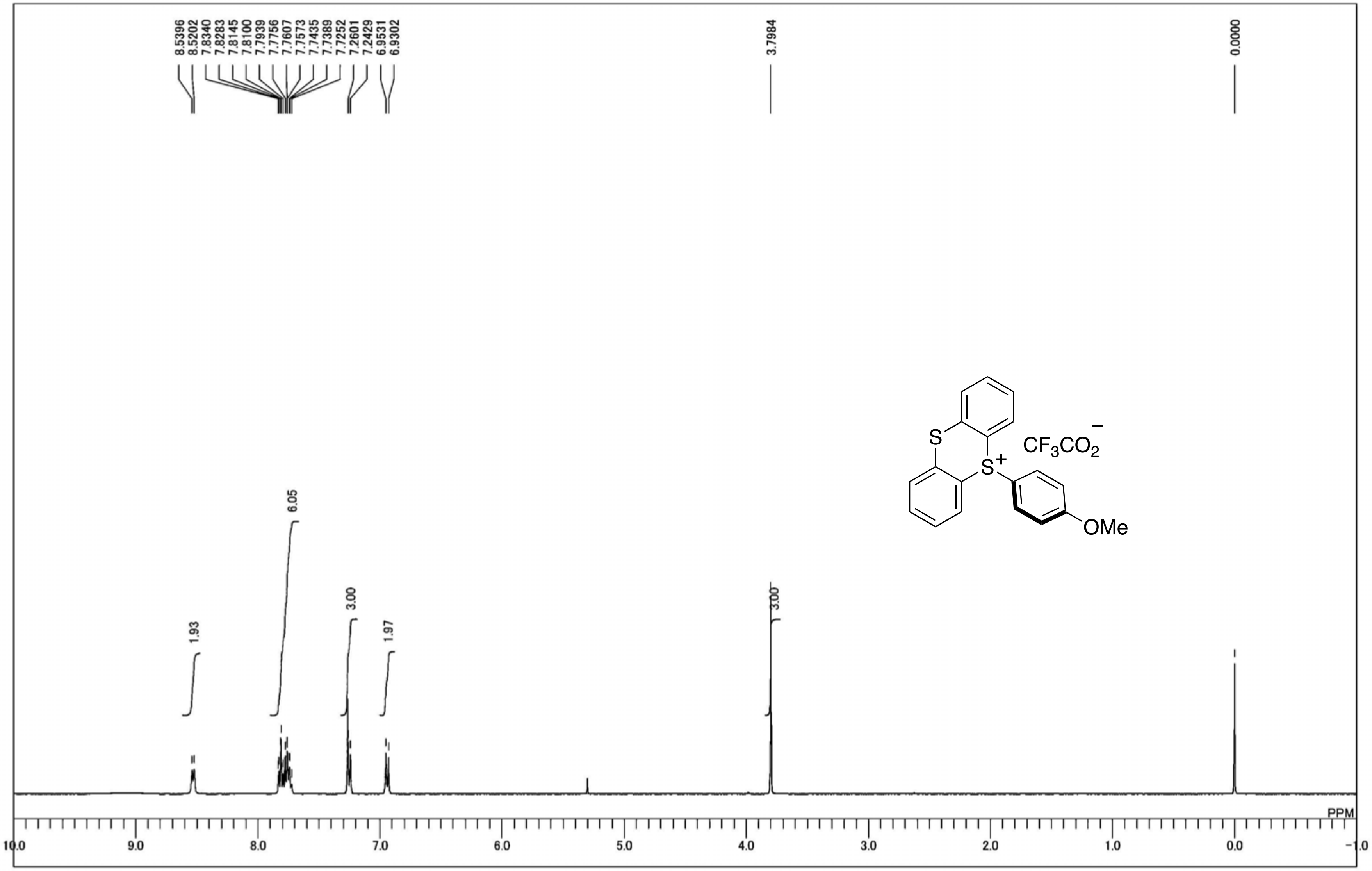

Supplementary Fig. 11 | ^{1}H NMR spectrum of **5b-S**

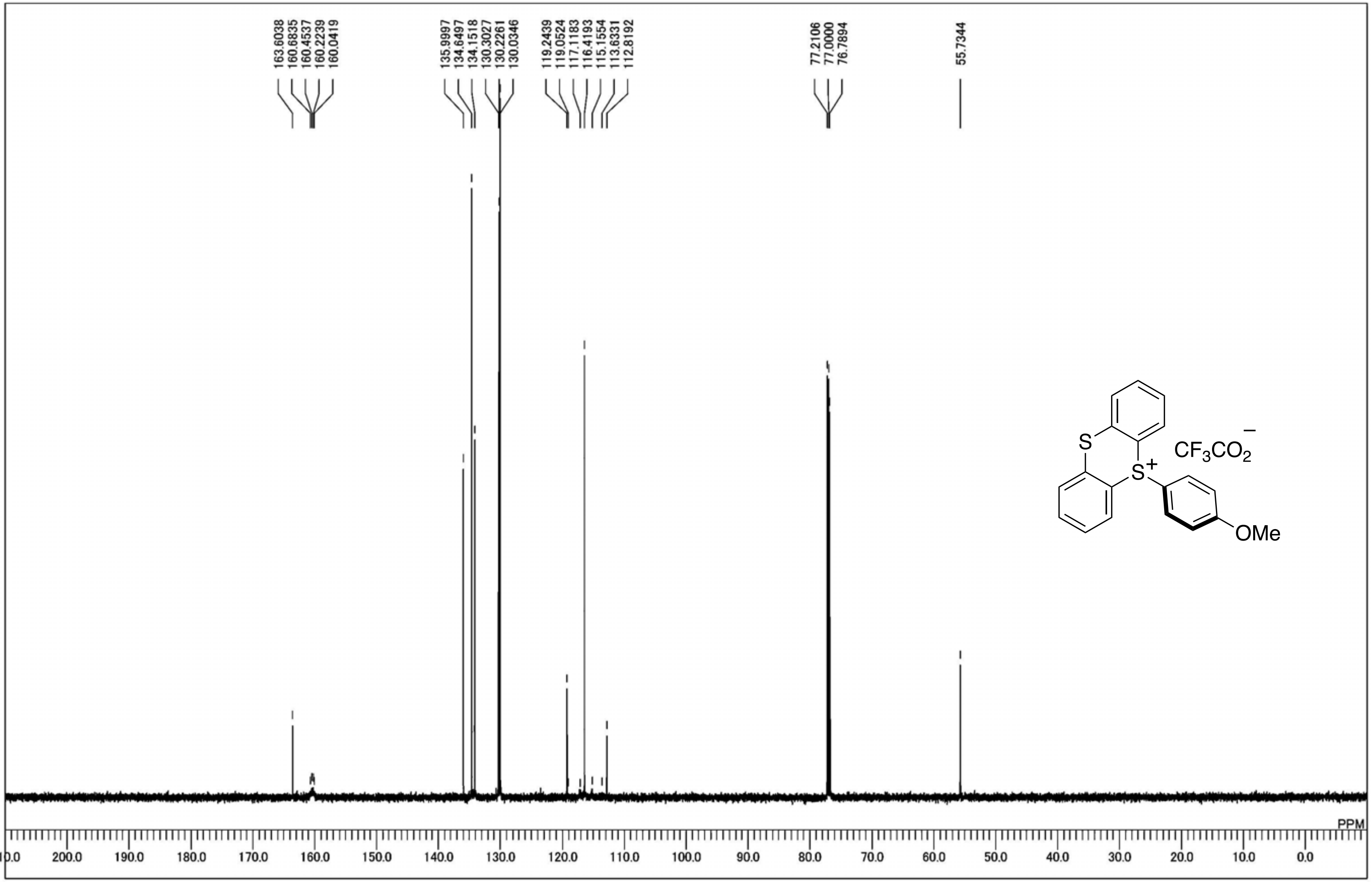

Supplementary Fig. 12 | ^{13}C NMR spectrum of **5b-S**

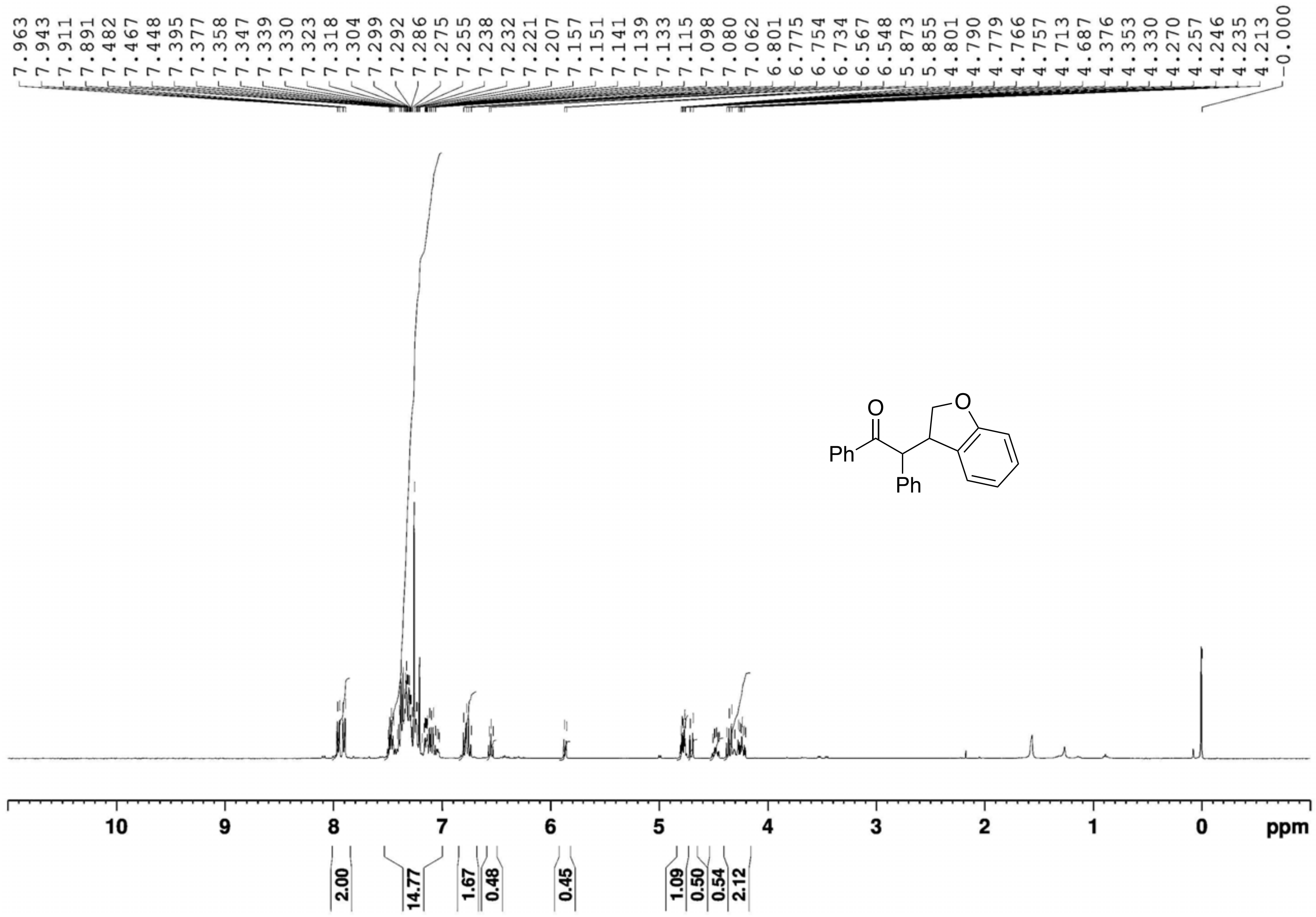

Supplementary Fig. 13 | ^{1}H NMR spectrum of **3aa**

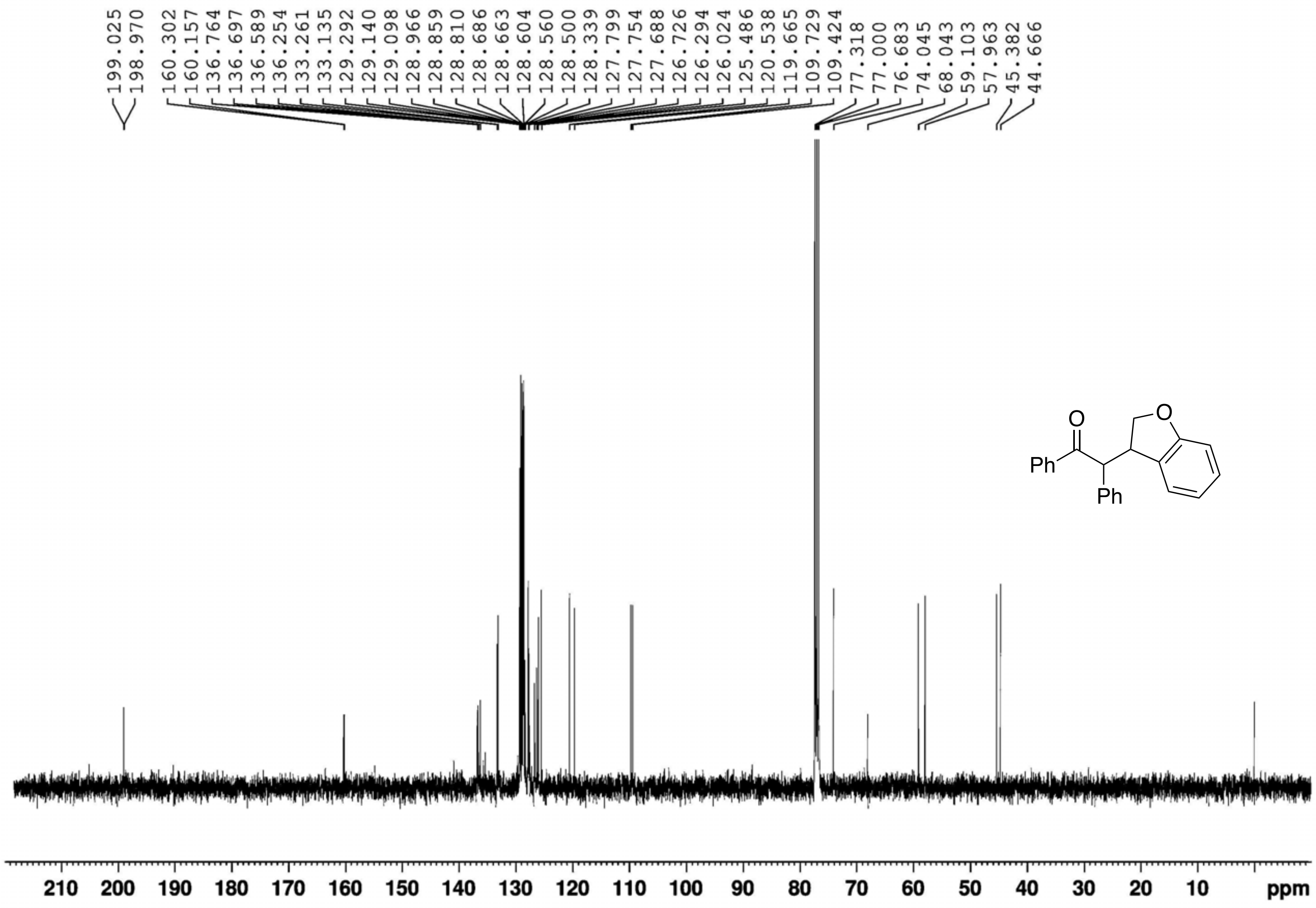

Supplementary Fig. 14 | ^{13}C NMR spectrum of **3aa**

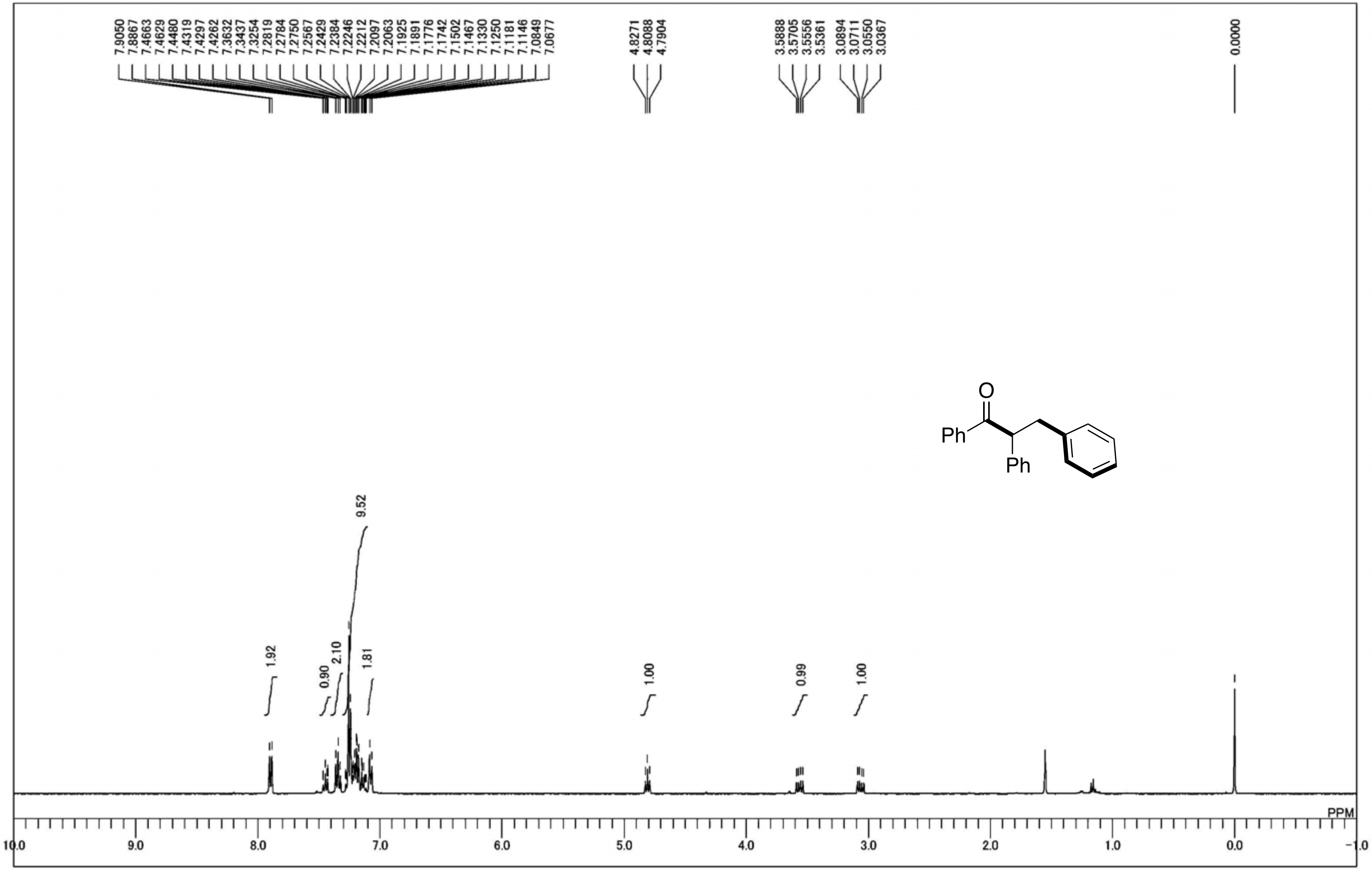

Supplementary Fig. 15 | ^{1}H NMR spectrum of **6aaa**

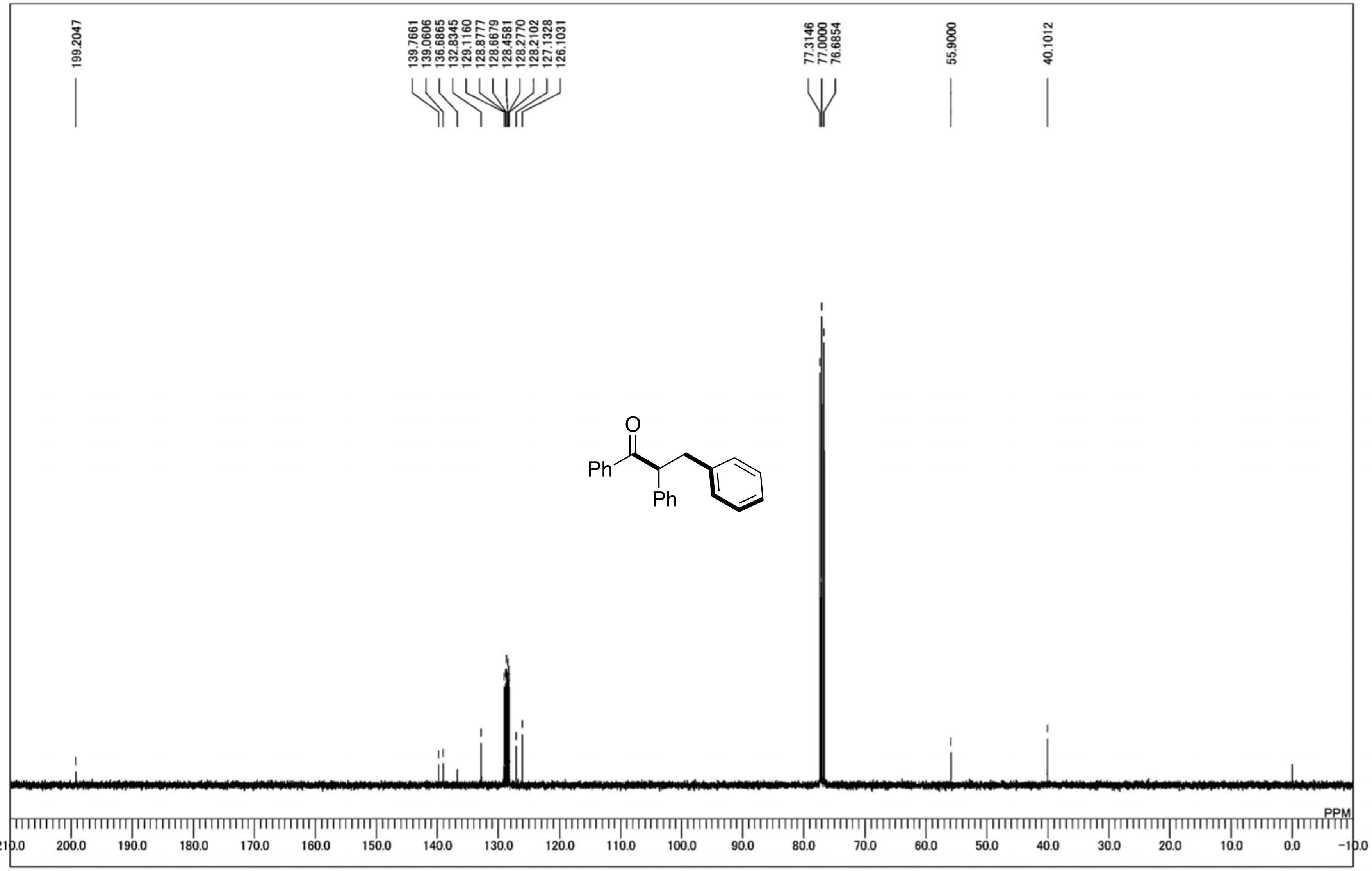

Supplementary Fig. 16 | ^{13}C NMR spectrum of **6aaa**

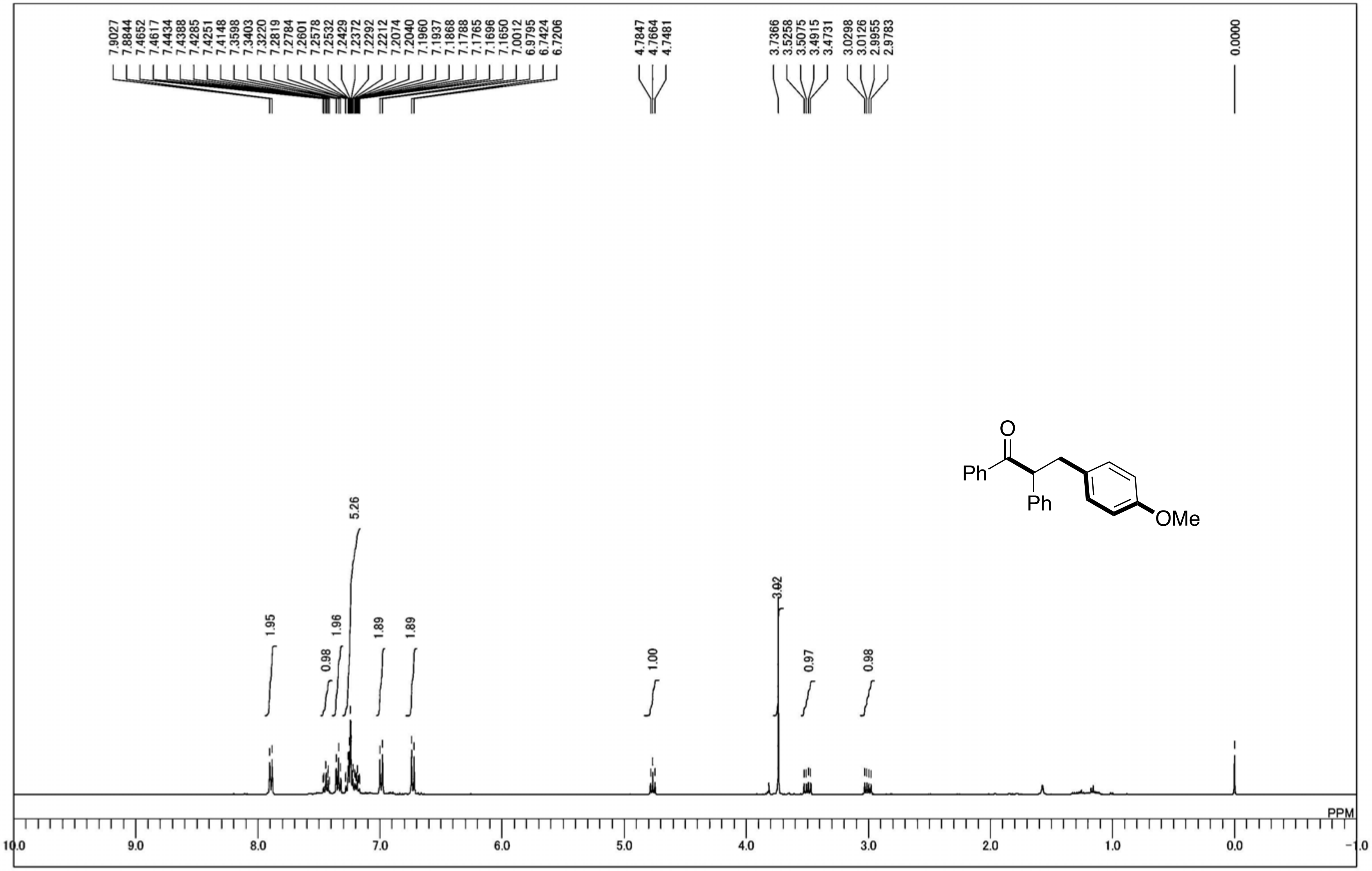

Supplementary Fig. 17 | ^{1}H NMR spectrum of **6aab**

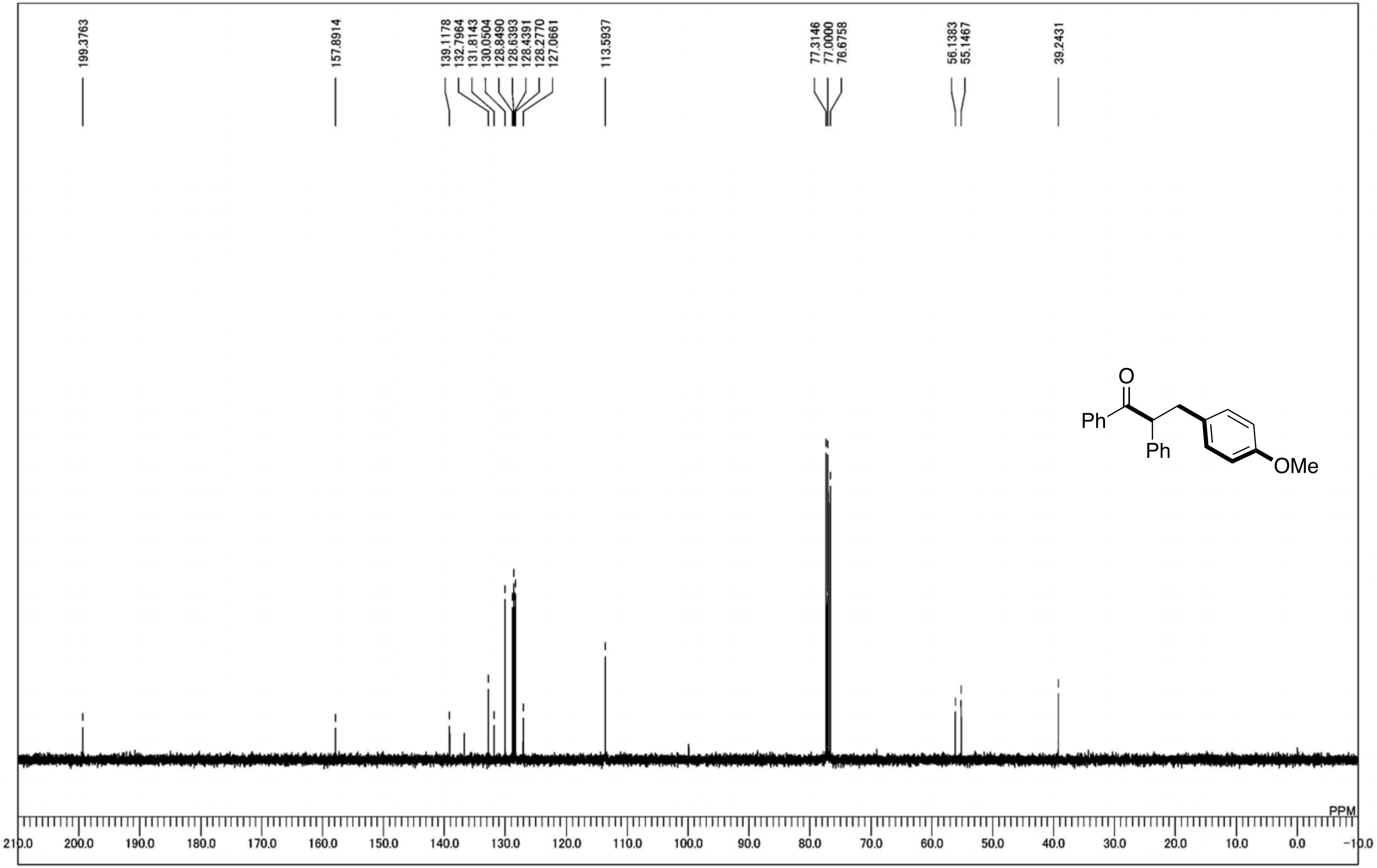

Supplementary Fig. 18 | ^{13}C NMR spectrum of **6aab**

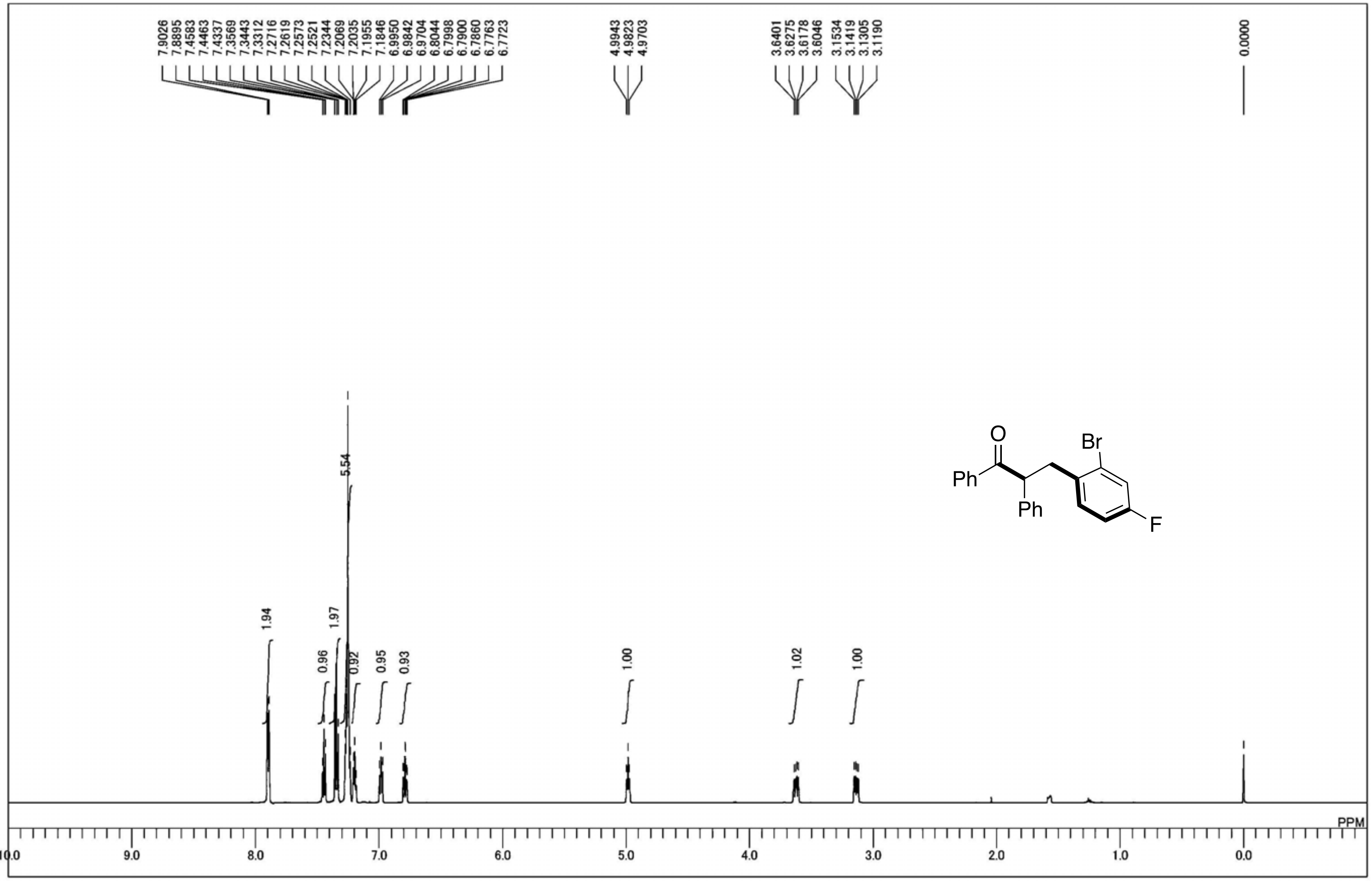

Supplementary Fig. 19 | ^{1}H NMR spectrum of **6aac**

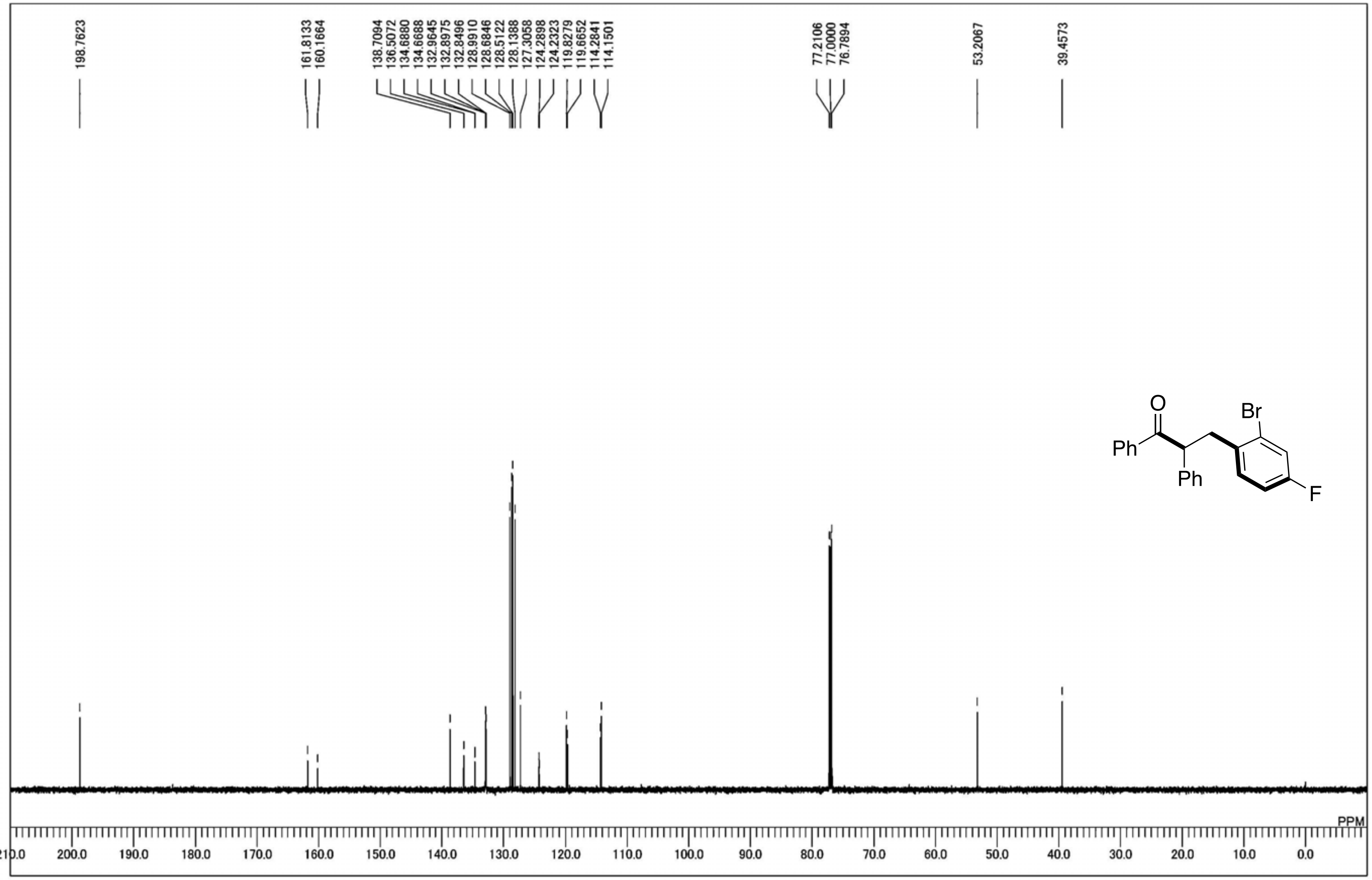

Supplementary Fig. 20 | ^{13}C NMR spectrum of **6aac**

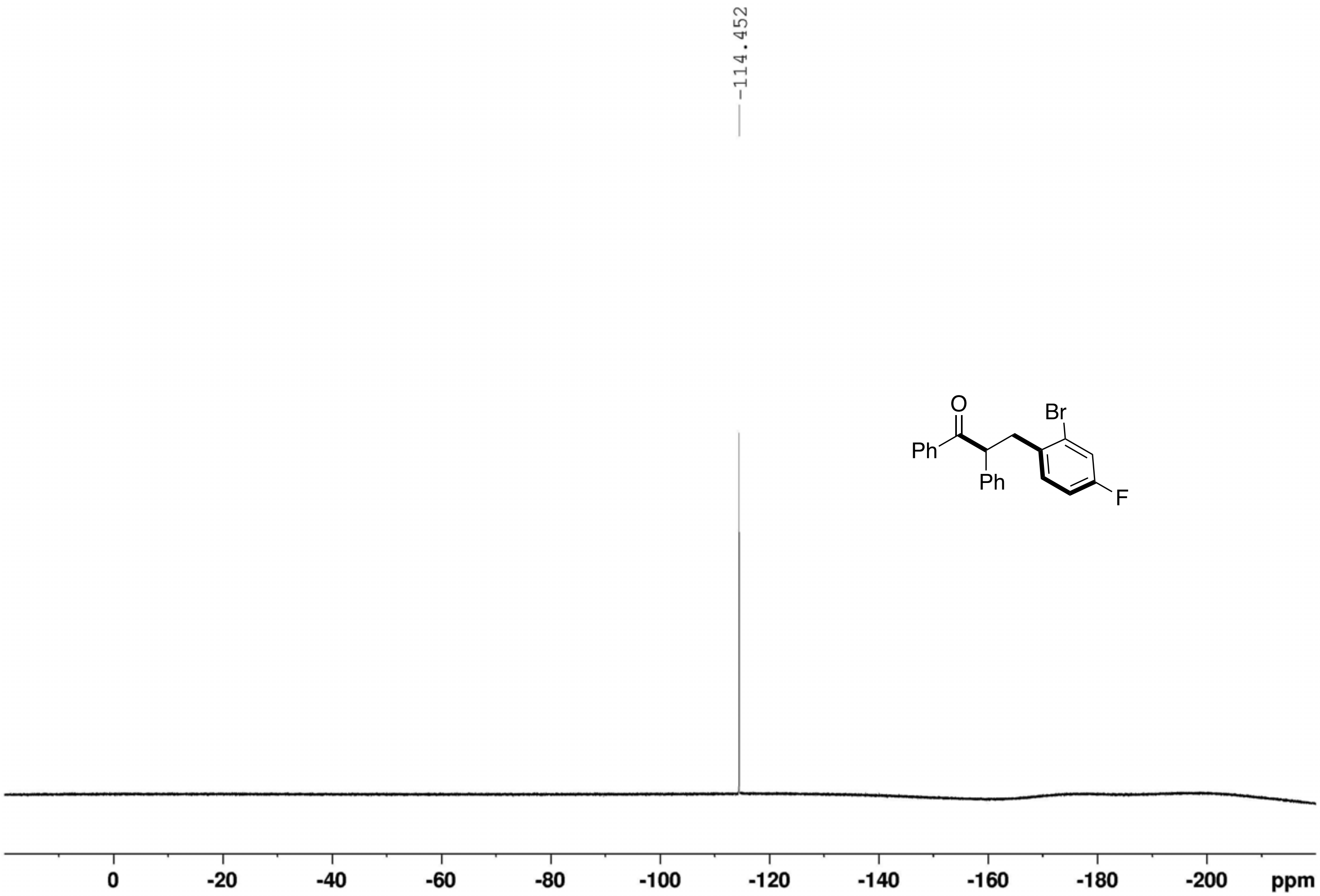

Supplementary Fig. 21 | ^{19}F NMR spectrum of **6aac**

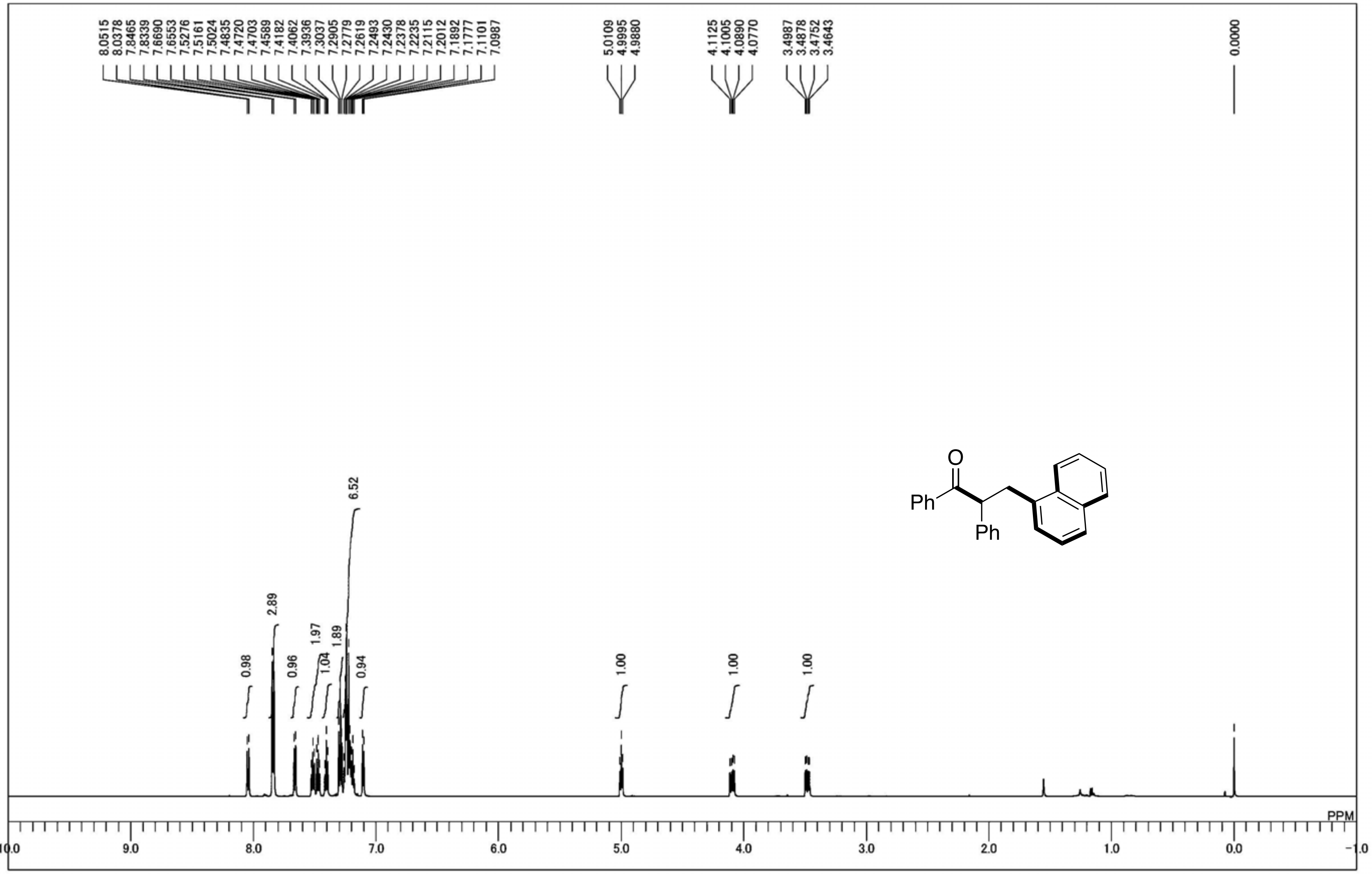

Supplementary Fig. 22 | ^{1}H NMR spectrum of **6aad**

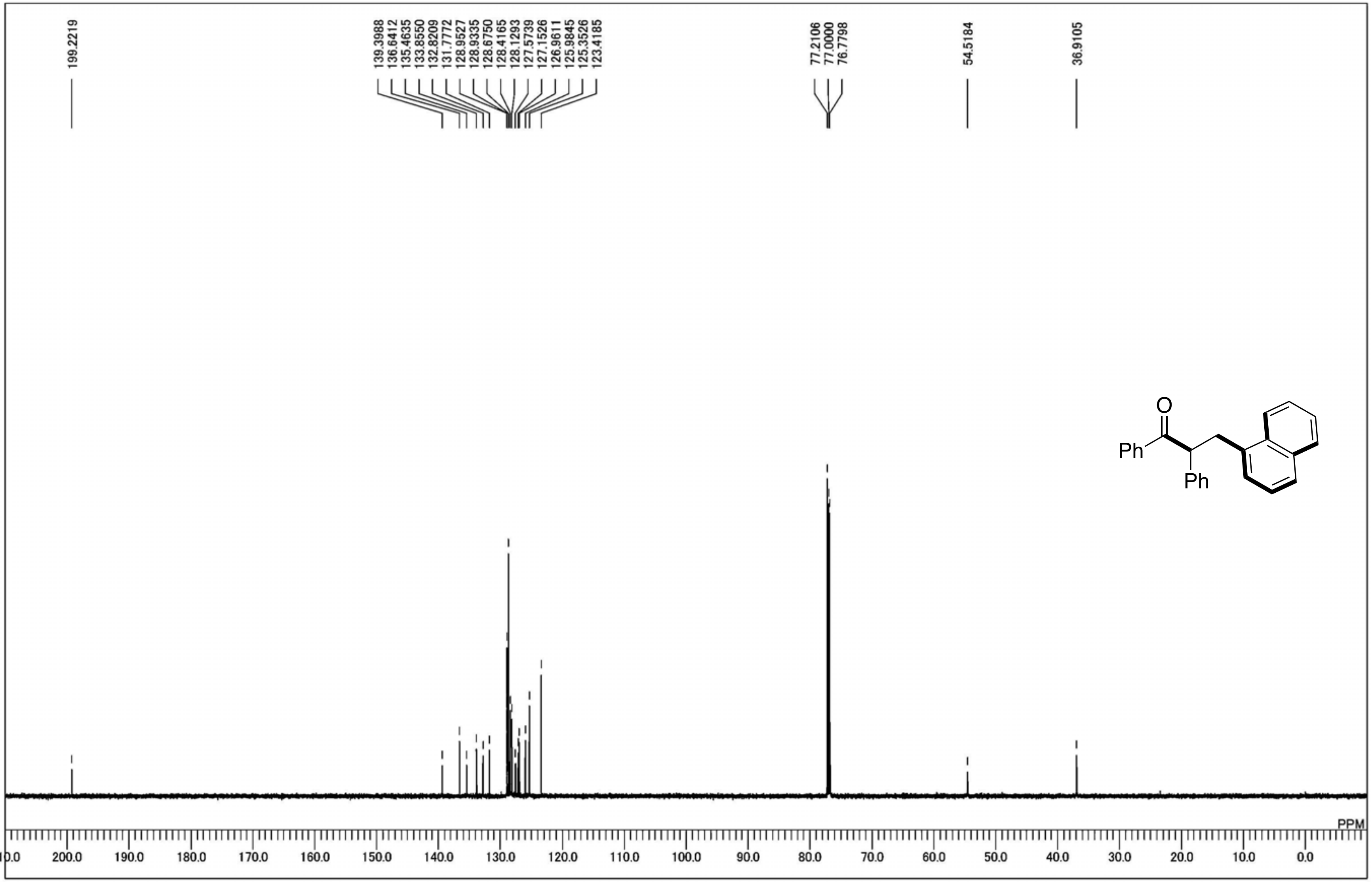

Supplementary Fig. 23 | ^{13}C NMR spectrum of **6aad**

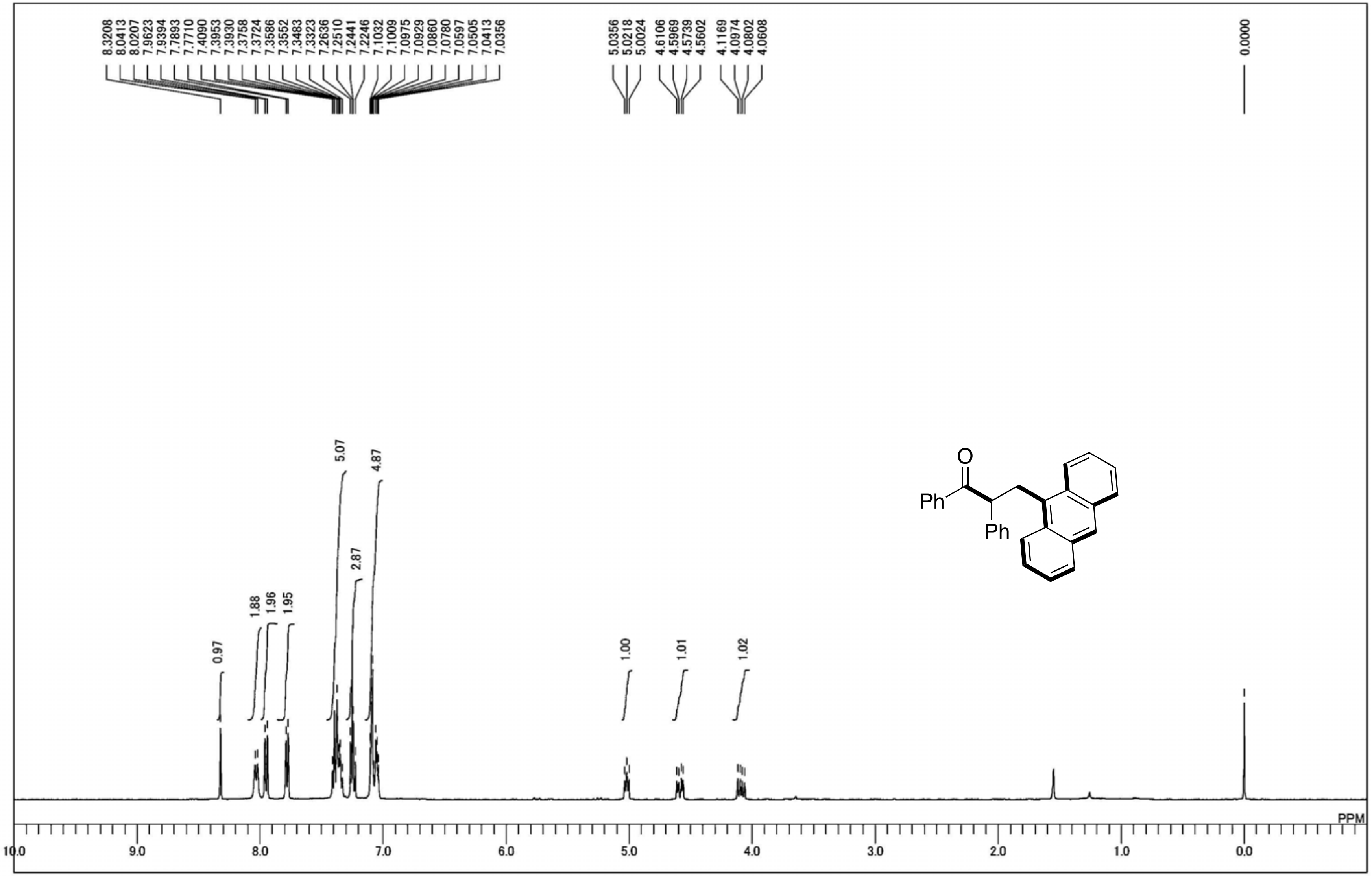

Supplementary Fig. 24 | ^{1}H NMR spectrum of **6aae**

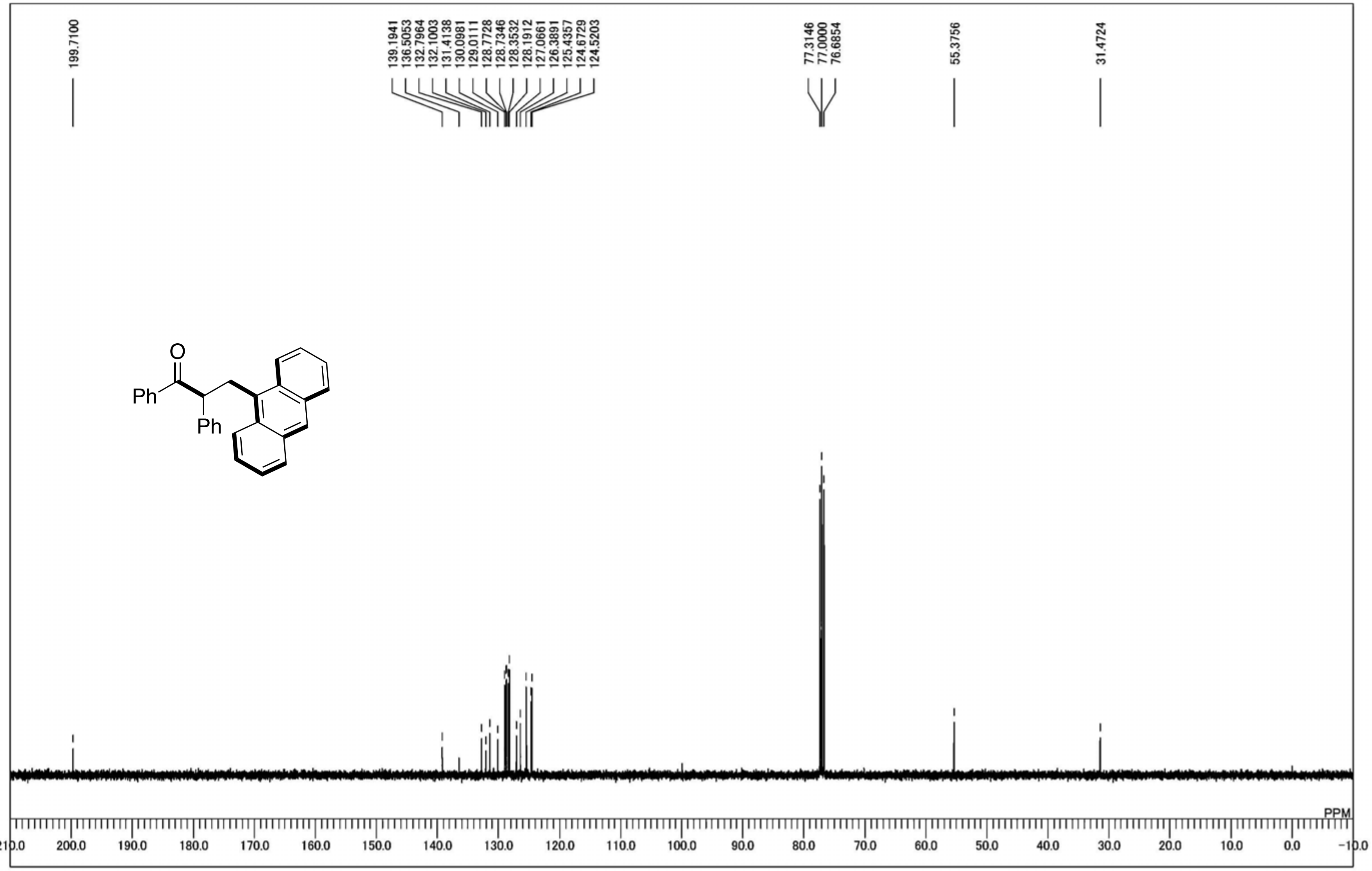

Supplementary Fig. 25 | ^{13}C NMR spectrum of **6aae**

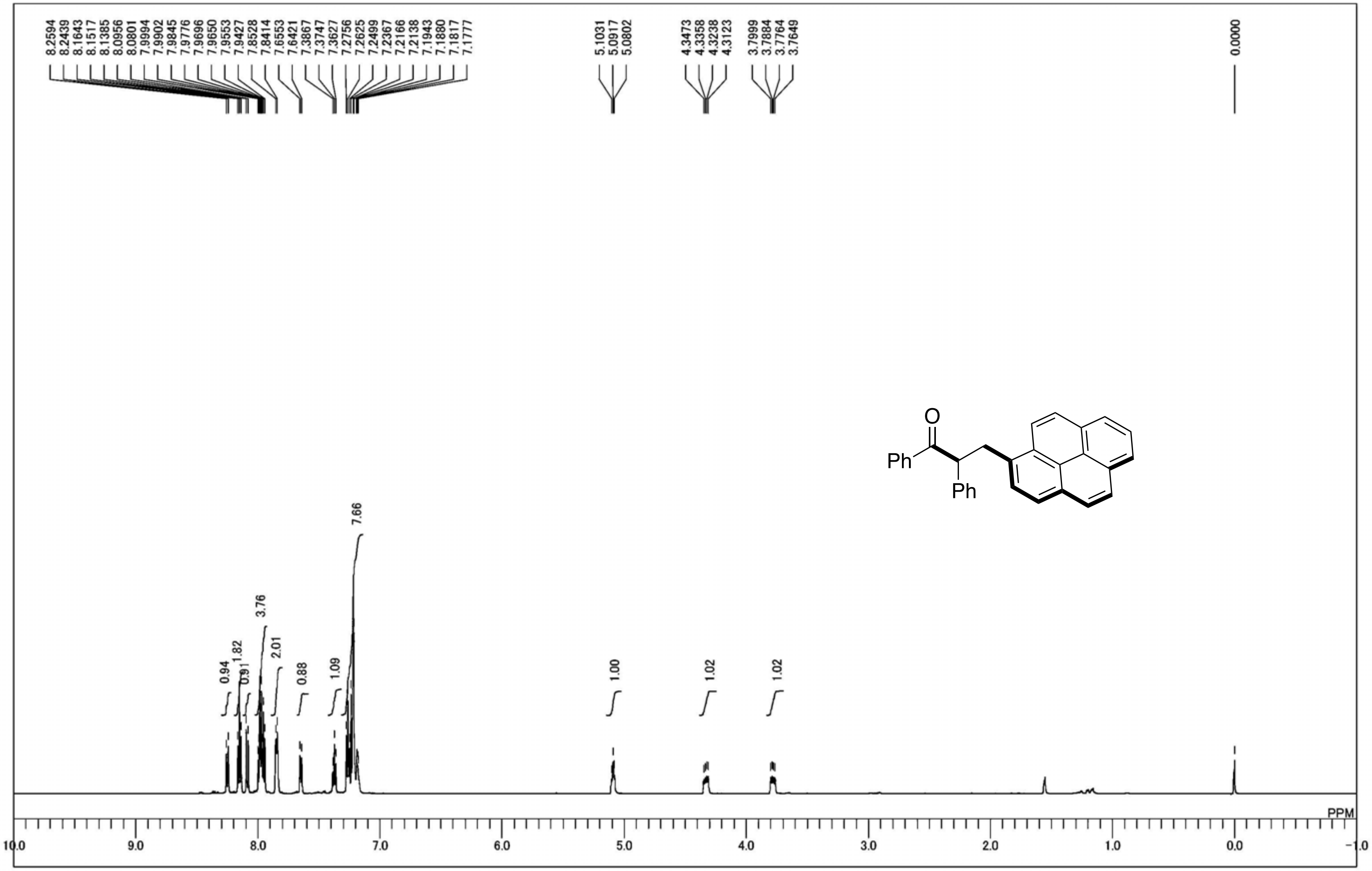

Supplementary Fig. 26 | ^{1}H NMR spectrum of **6aaf**

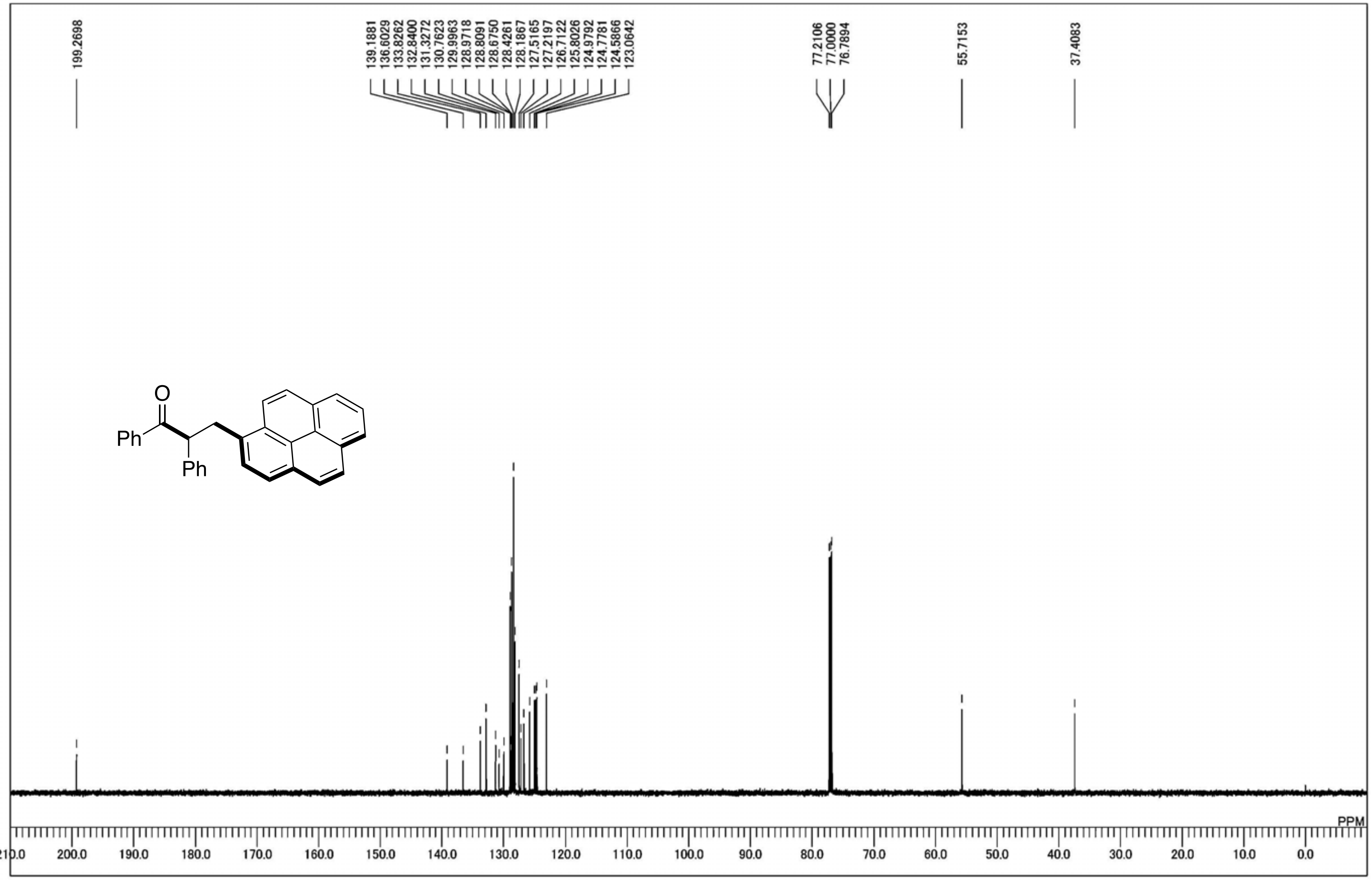

Supplementary Fig. 27 | ^{13}C NMR spectrum of **6aaf**

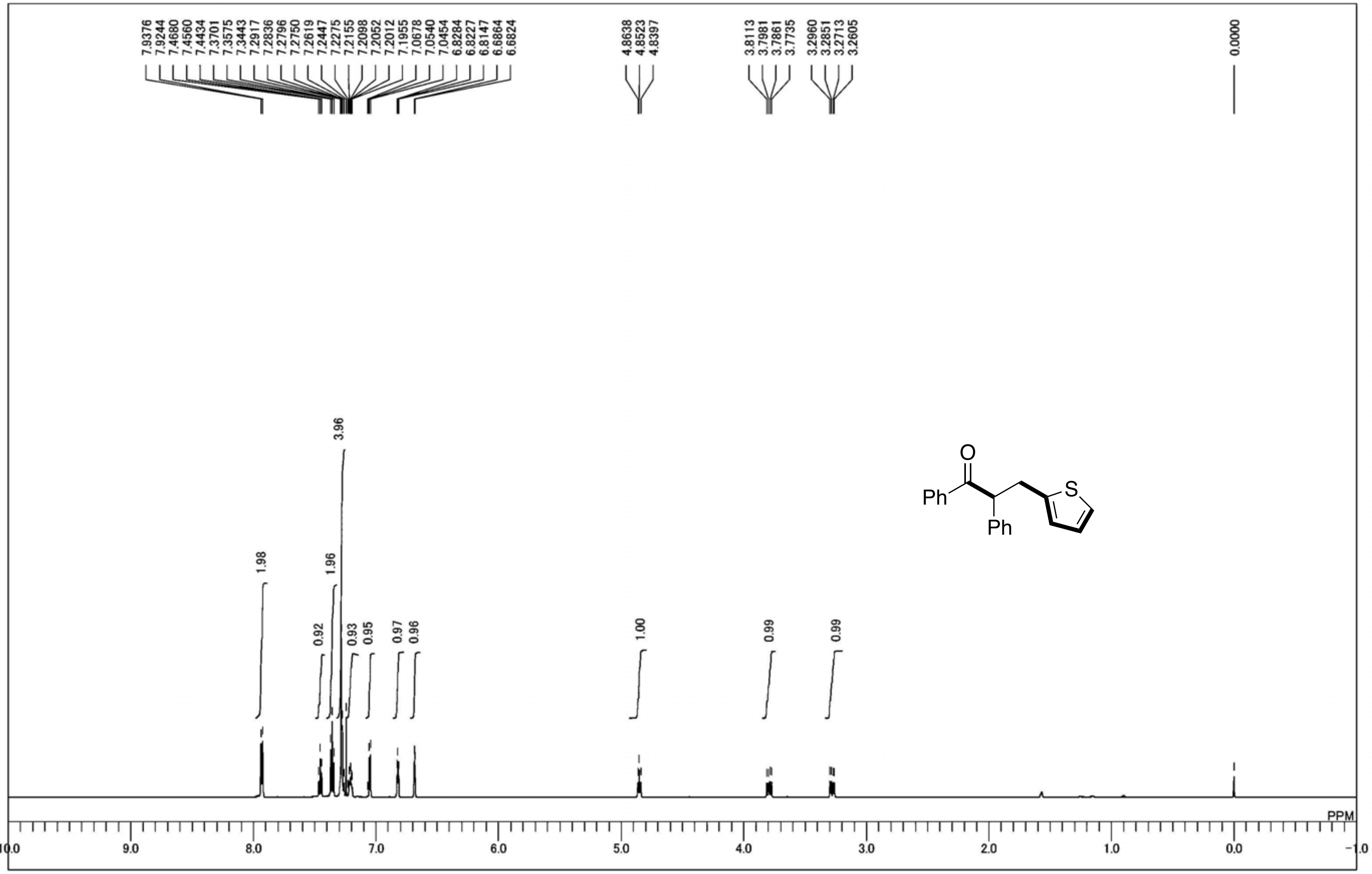

Supplementary Fig. 28 | ^{1}H NMR spectrum of **6aag**

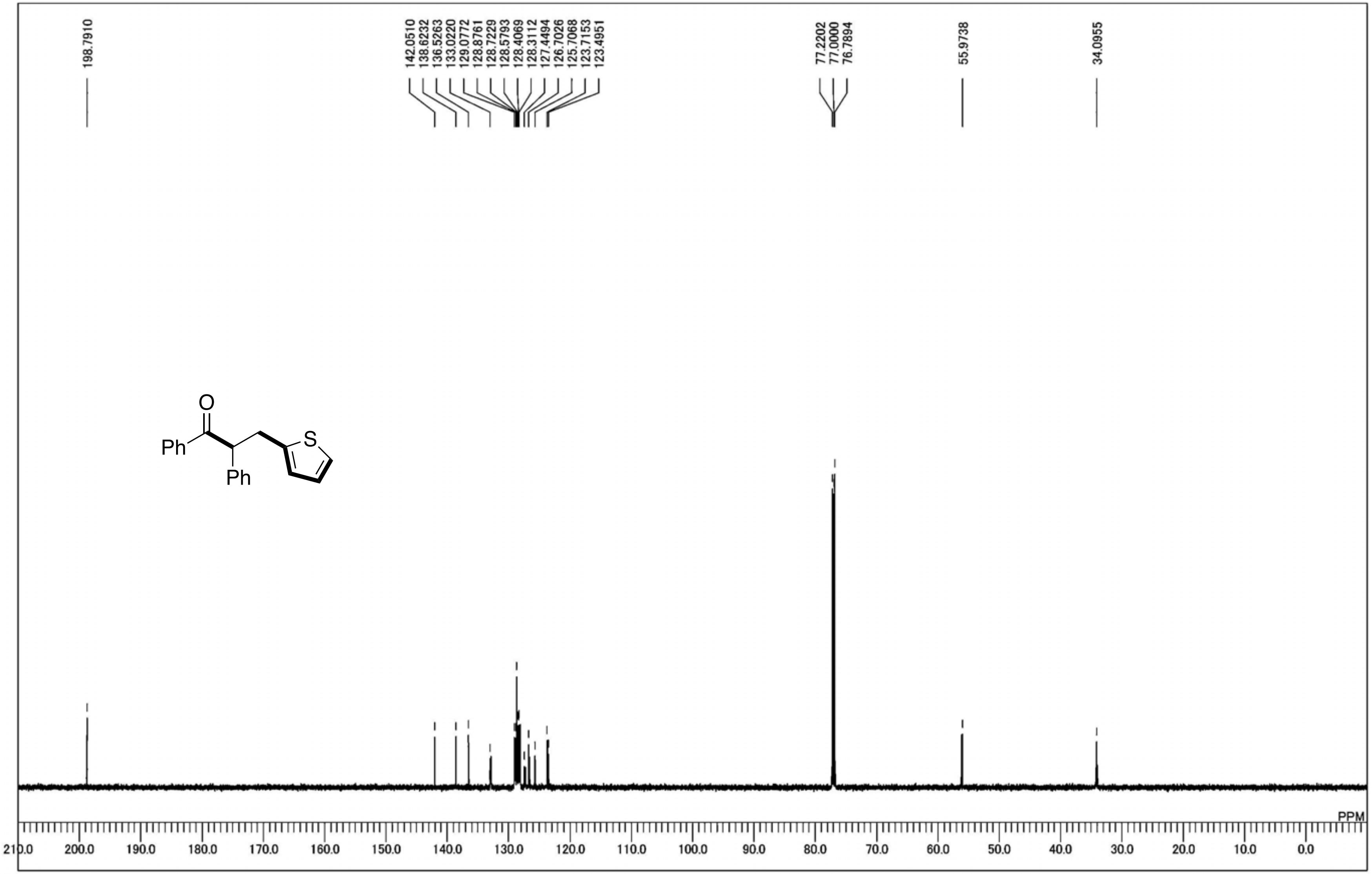

Supplementary Fig. 29 | ^{13}C NMR spectrum of **6aag**

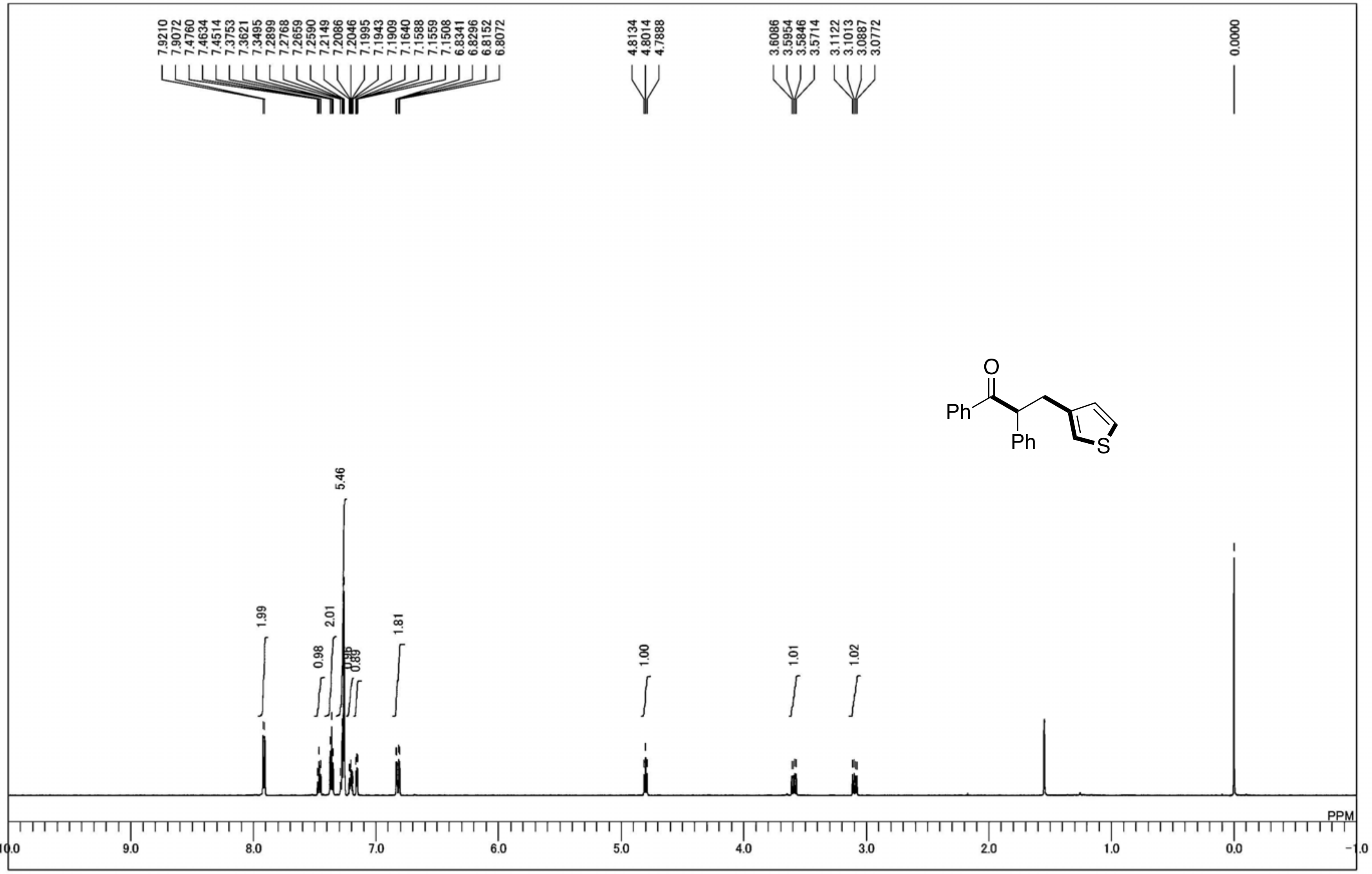

Supplementary Fig. 30 | ^{1}H NMR spectrum of **6aah**

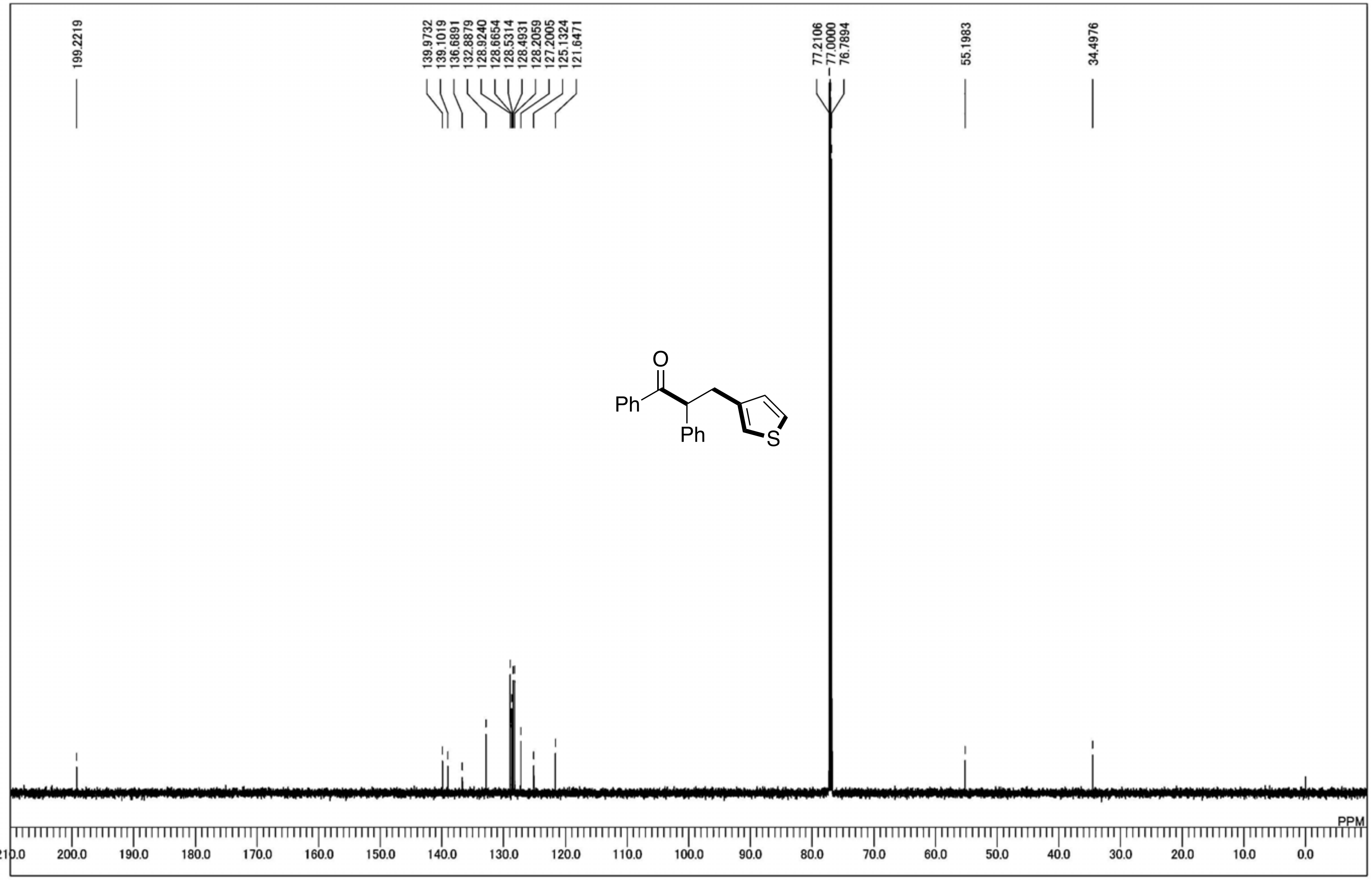

Supplementary Fig. 31 | ^{13}C NMR spectrum of **6aah**

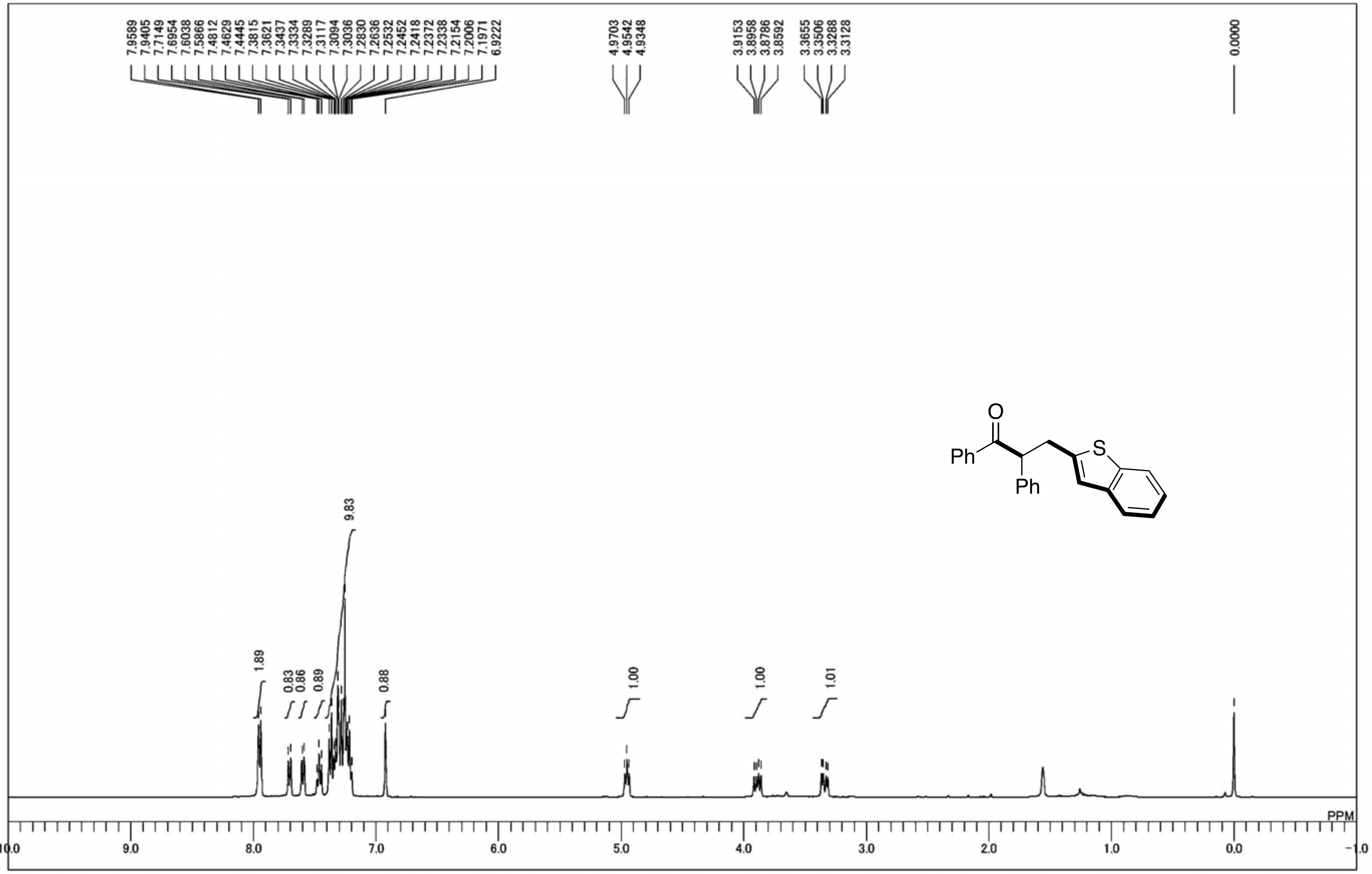

Supplementary Fig. 32 | ^{1}H NMR spectrum of **6aai**

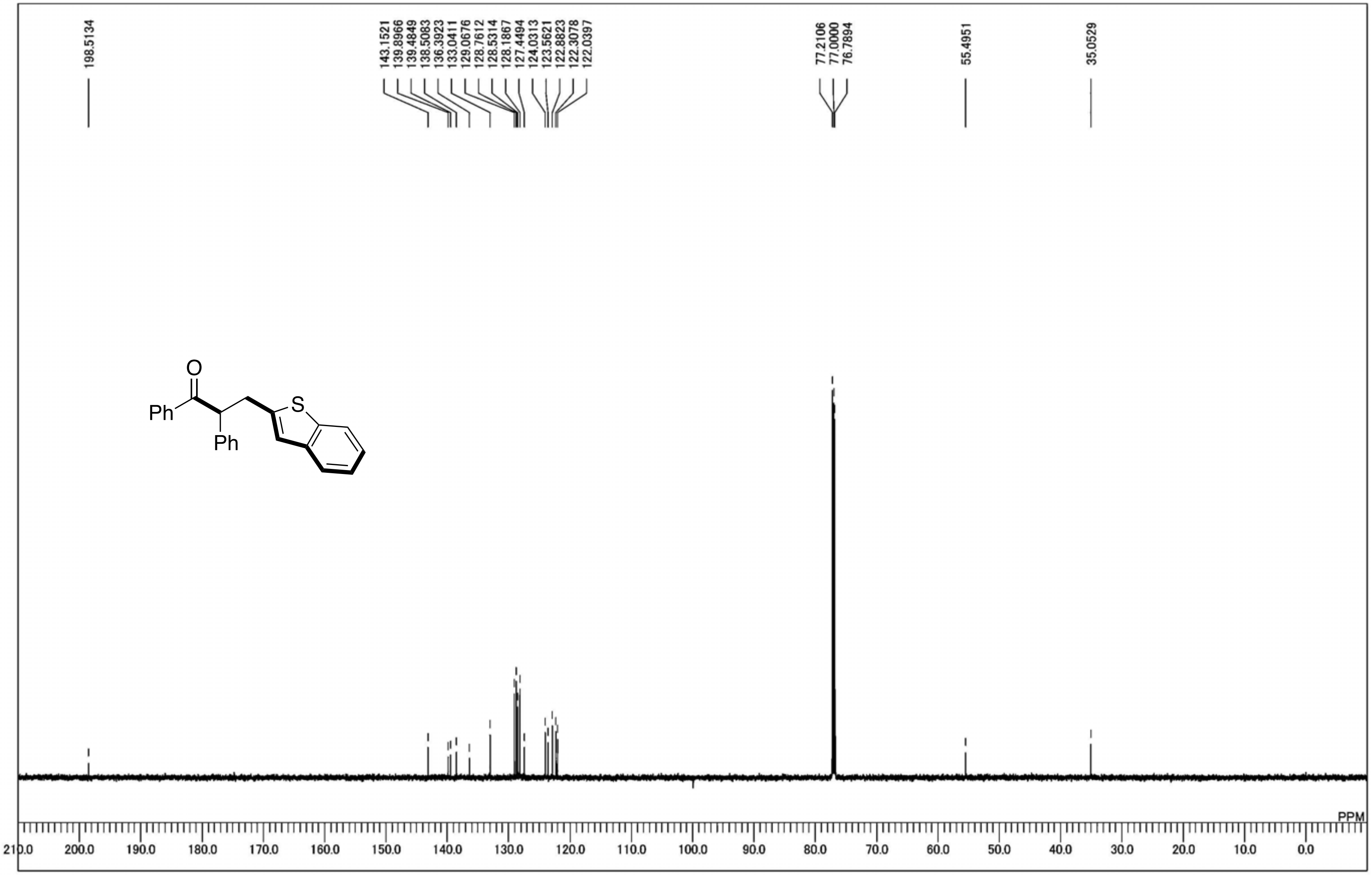

Supplementary Fig. 33 | ^{13}C NMR spectrum of **6aai**

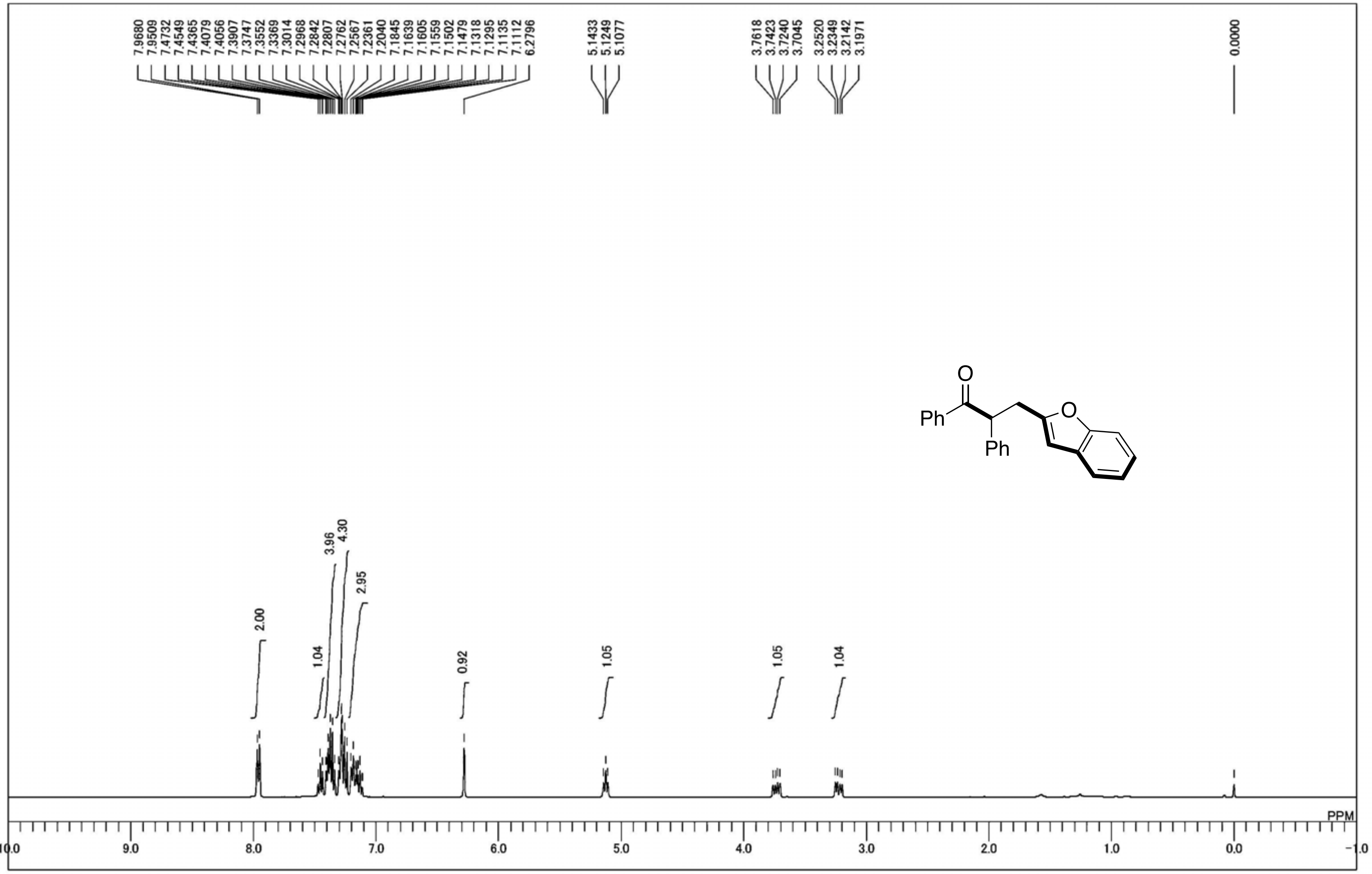

Supplementary Fig. 34 | ^{1}H NMR spectrum of **6aaj**

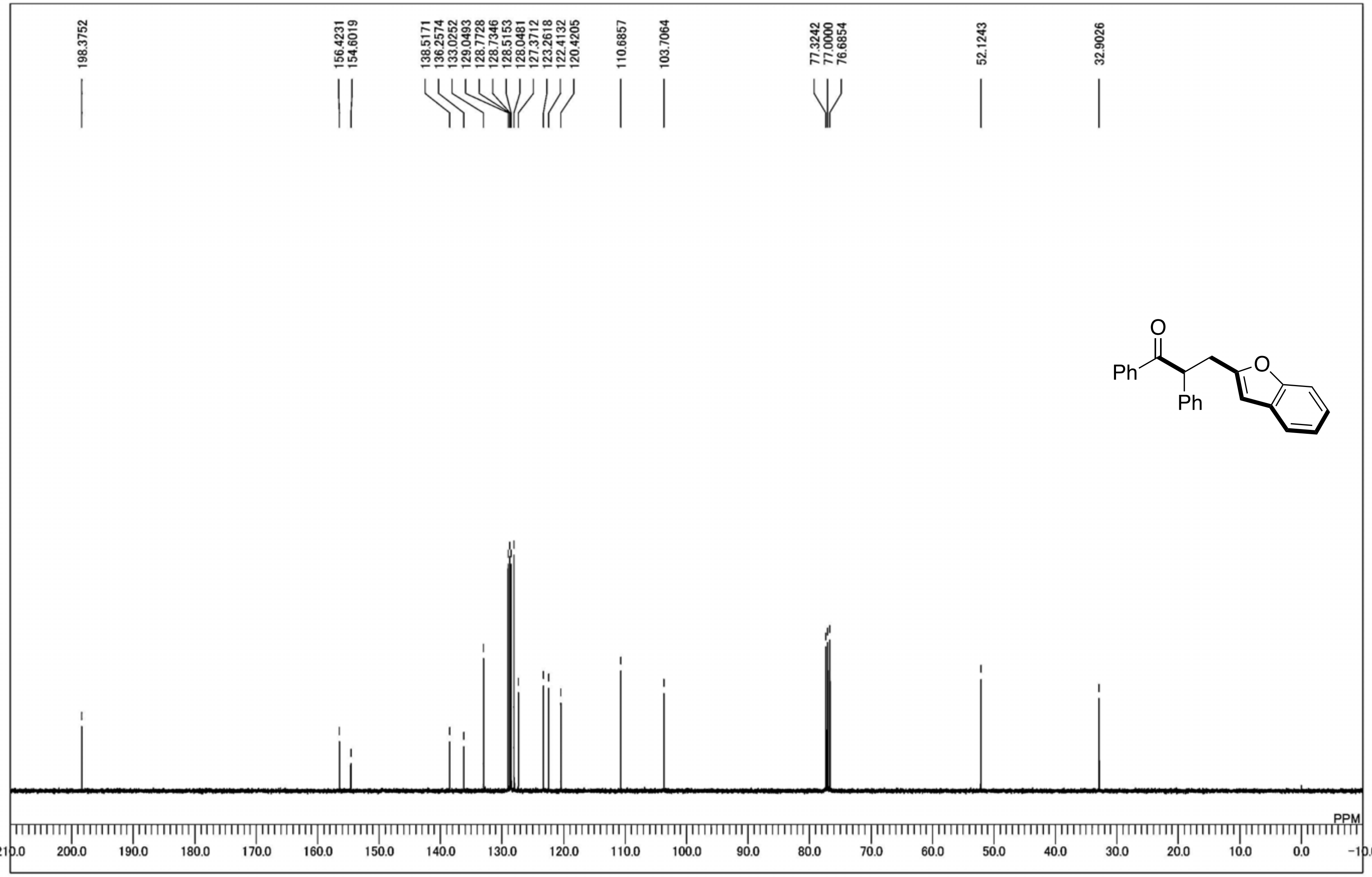

Supplementary Fig. 35 | ^{13}C NMR spectrum of **6aaj**

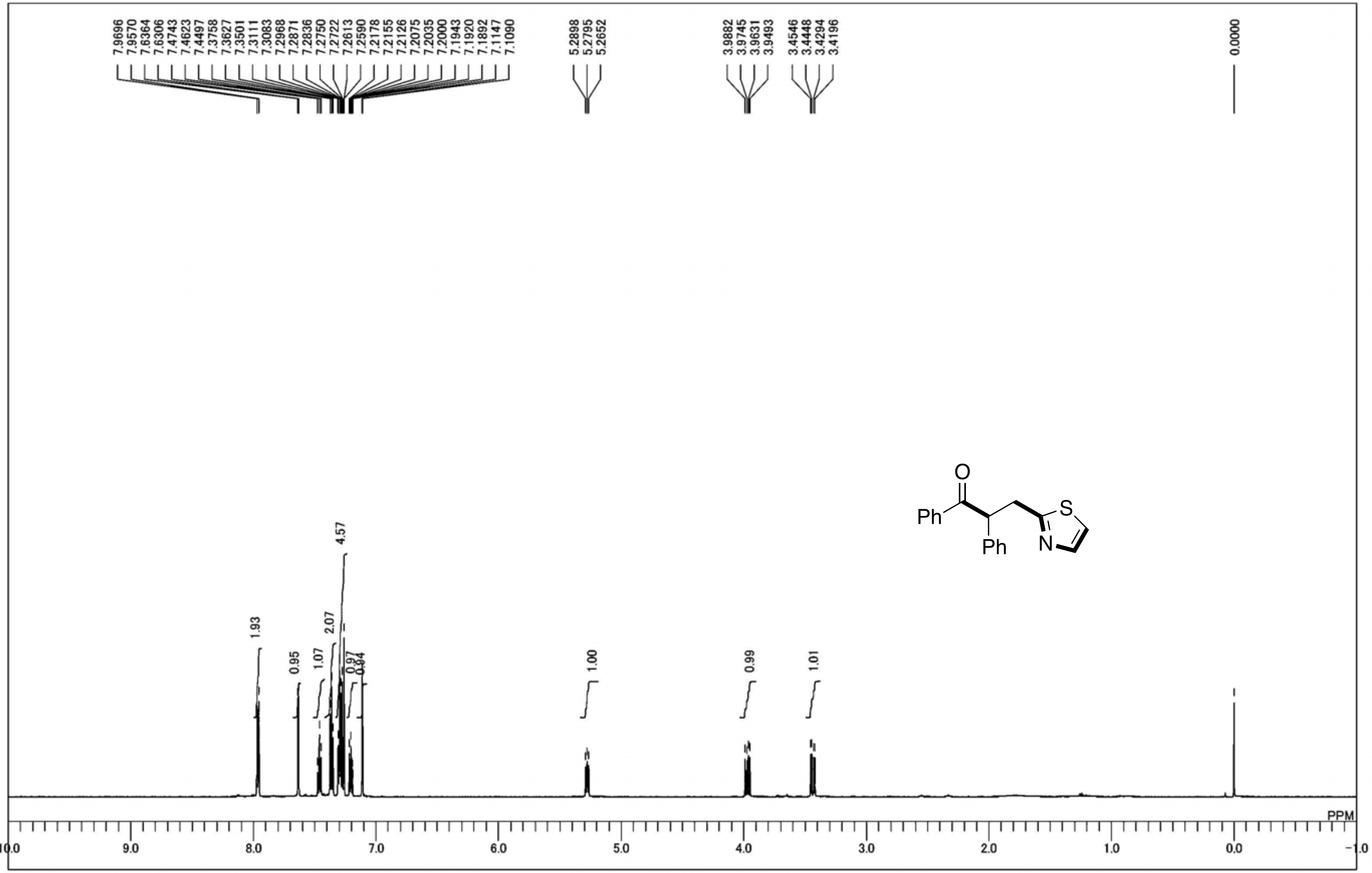

Supplementary Fig. 36 | ^{1}H NMR spectrum of **6aak**

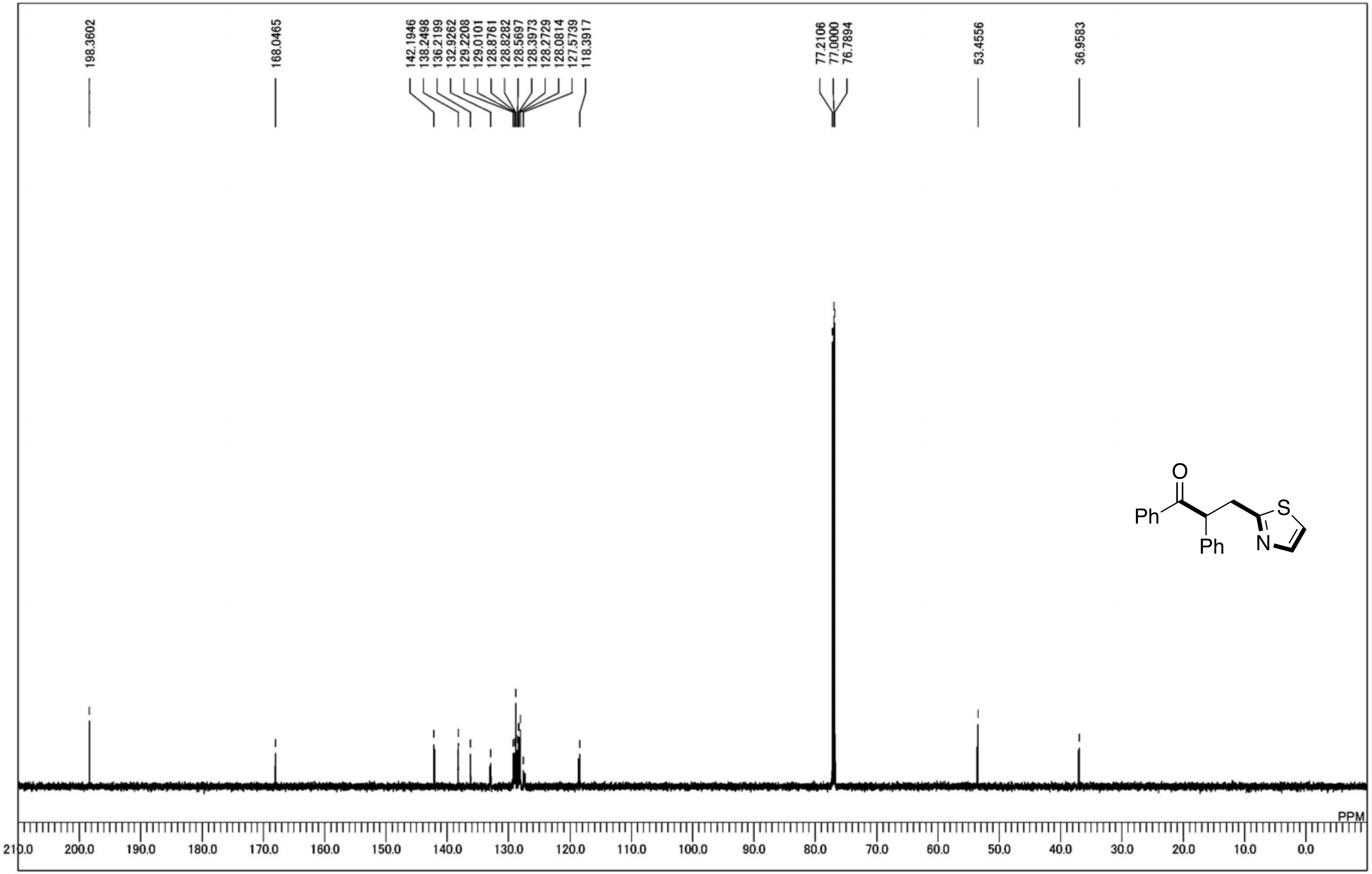

Supplementary Fig. 37 | ^{13}C NMR spectrum of **6aak**

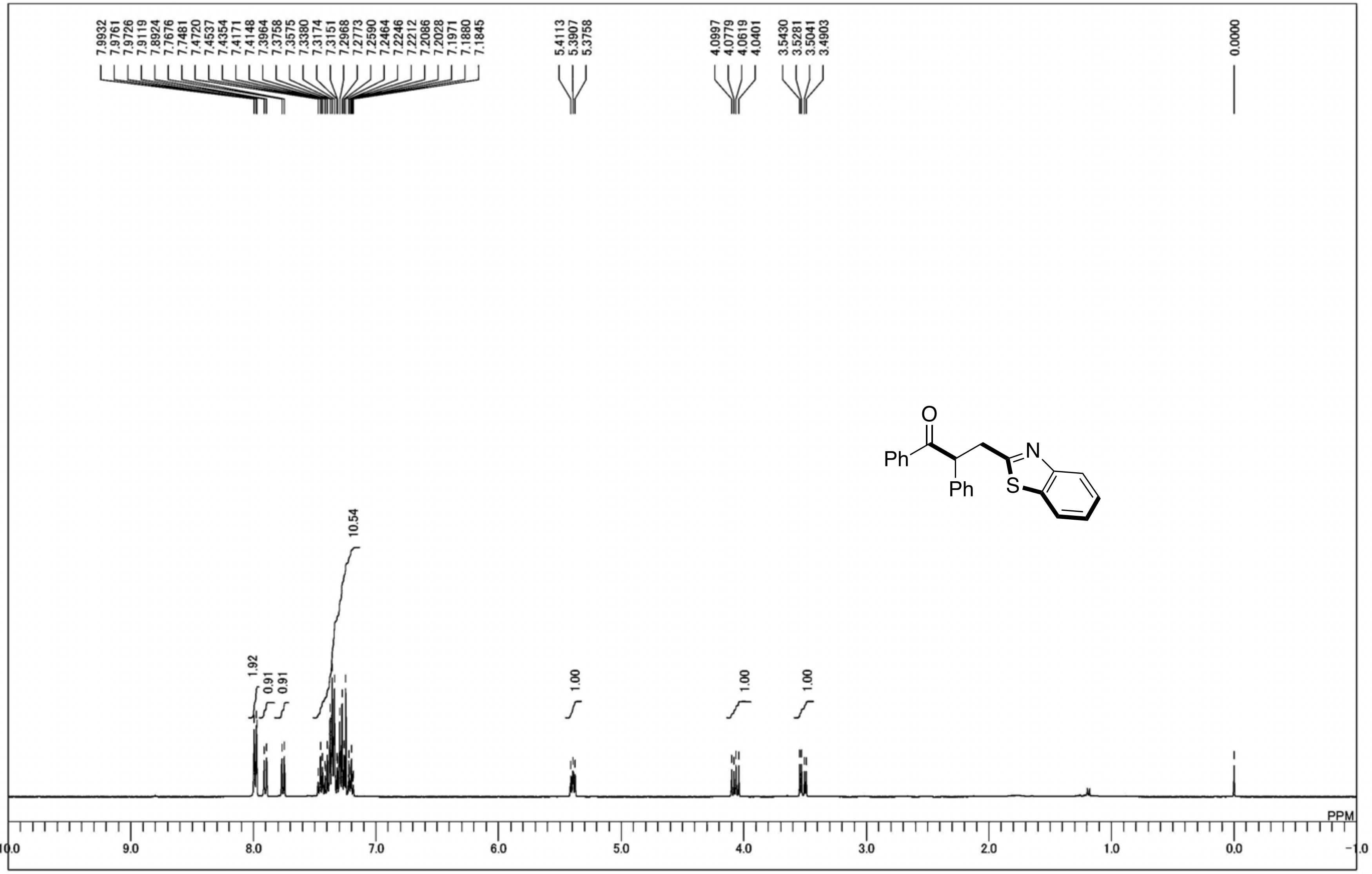

Supplementary Fig. 38 | ^{1}H NMR spectrum of **6aal**

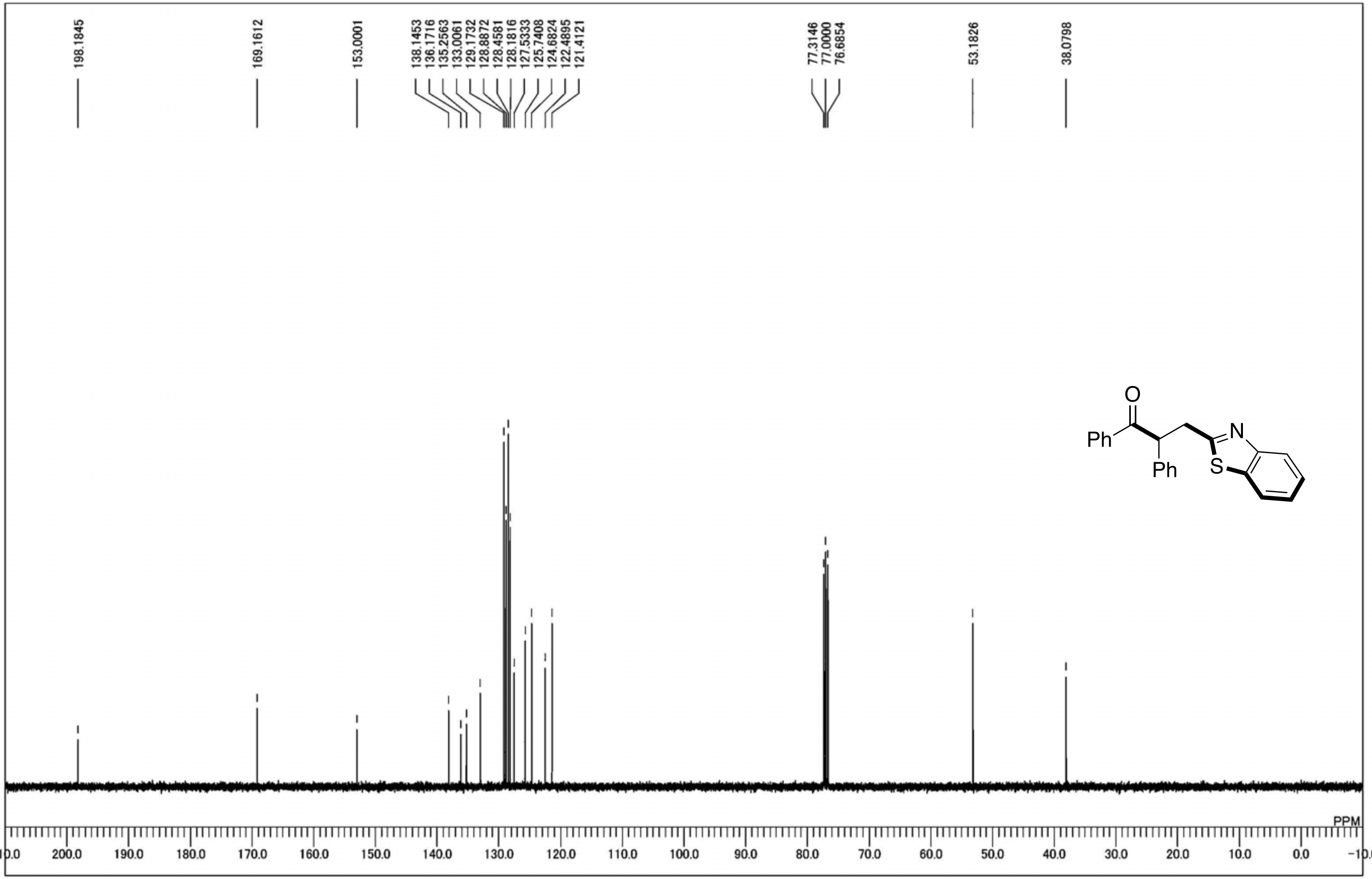

Supplementary Fig. 39 | ^{13}C NMR spectrum of **6aal**

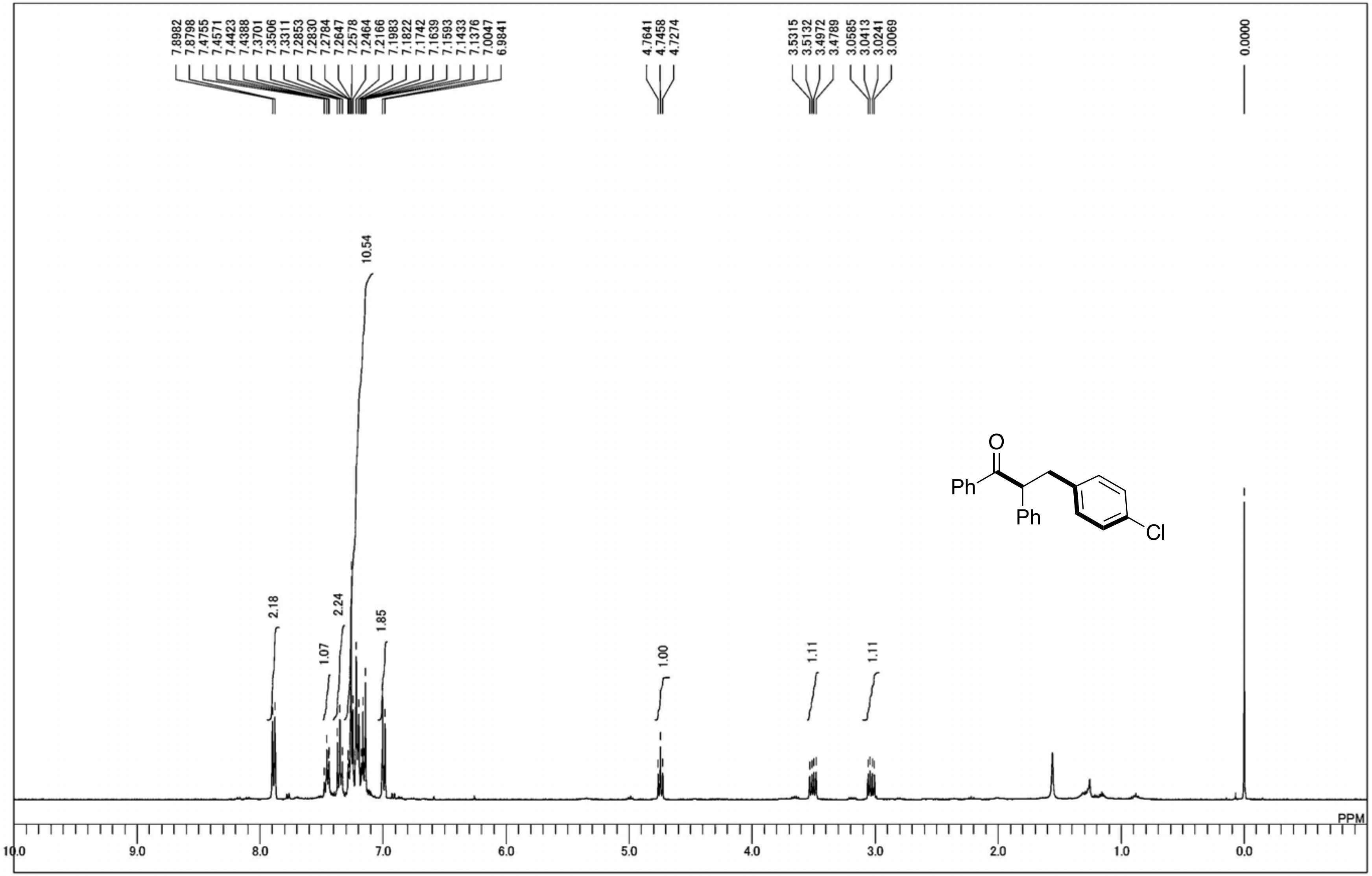

Supplementary Fig. 40 | ^{1}H NMR spectrum of **6aam**

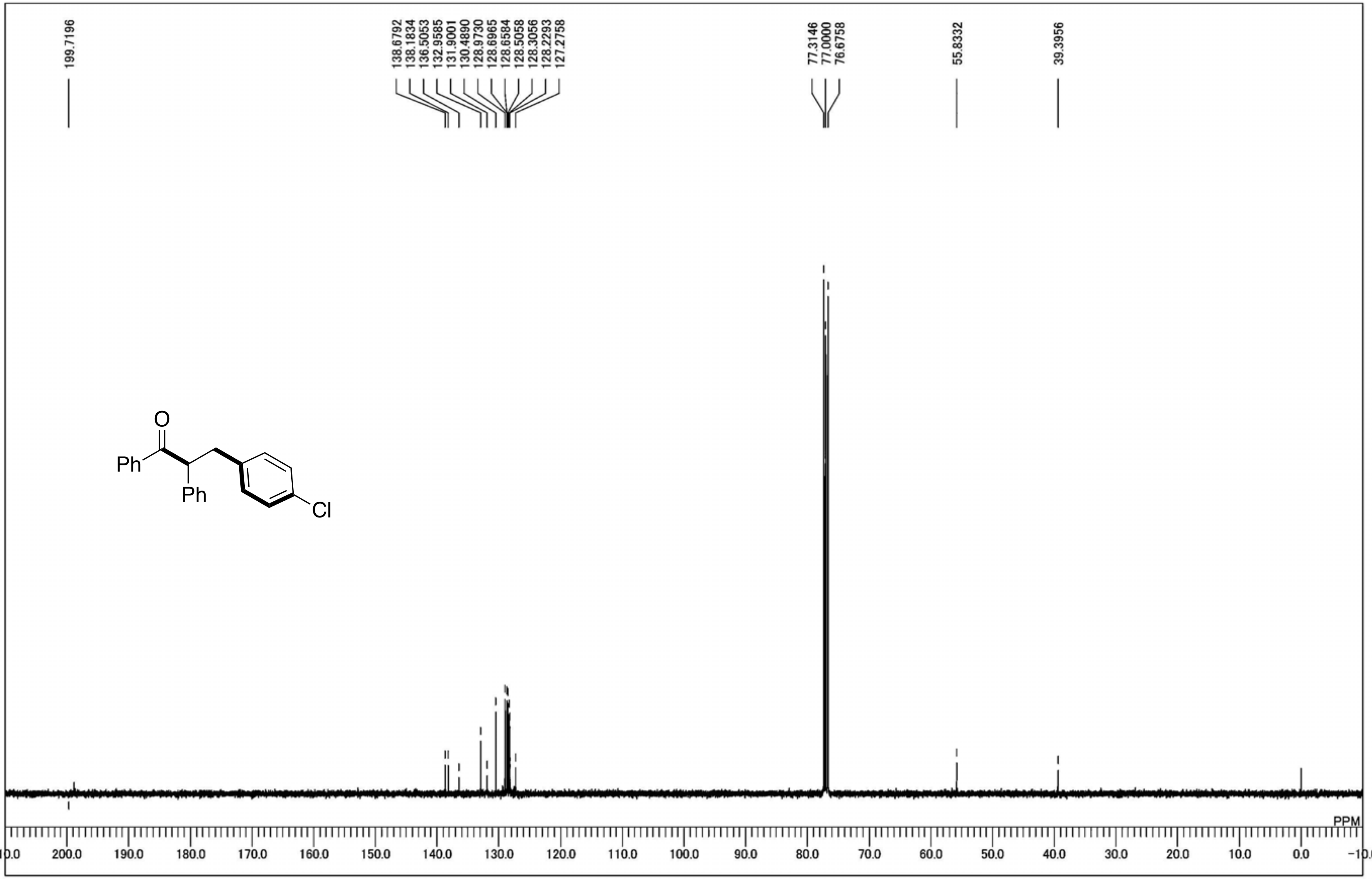

Supplementary Fig. 41 | ^{13}C NMR spectrum of **6aam**

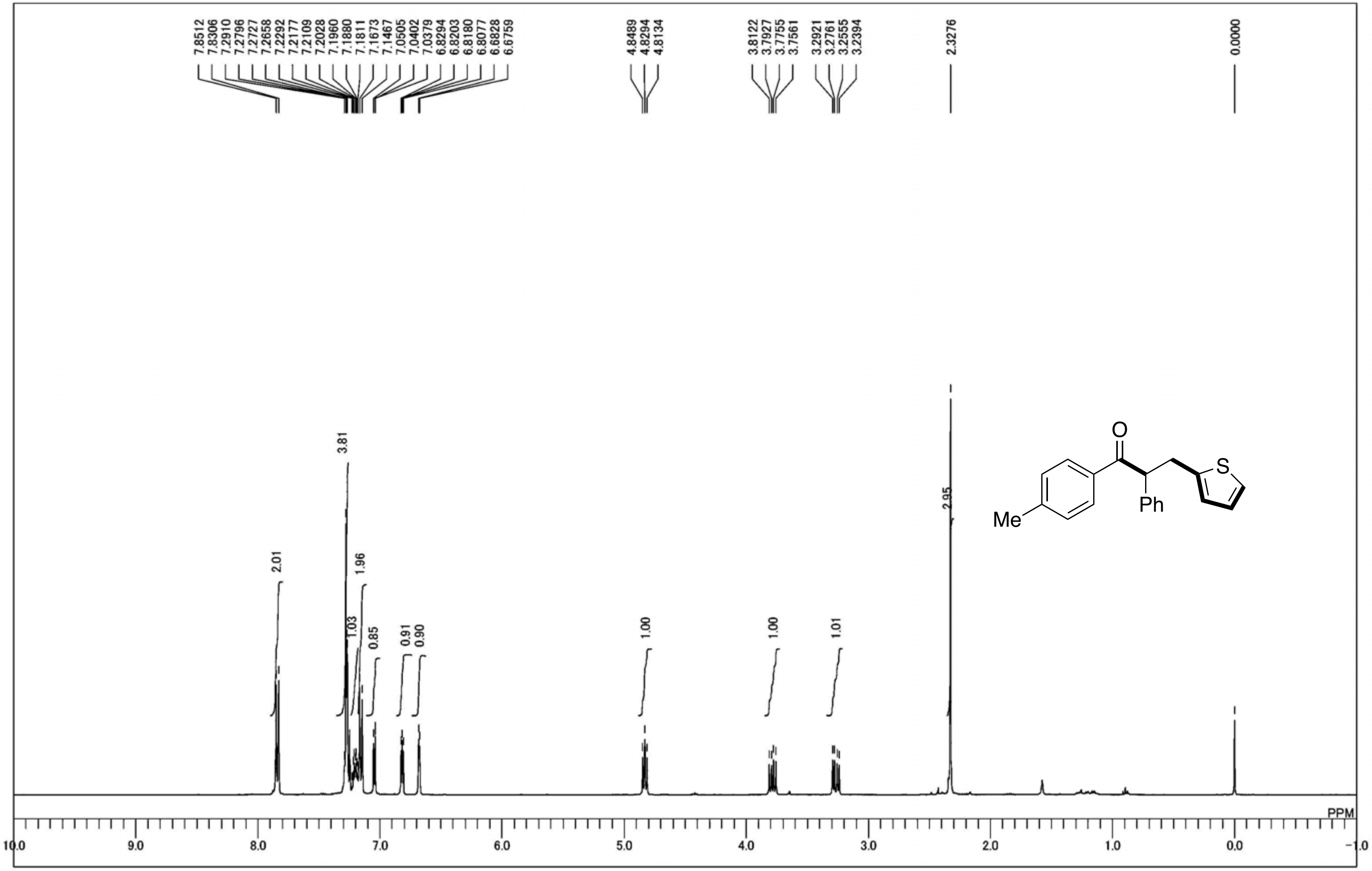

Supplementary Fig. 42 | ^{1}H NMR spectrum of **6bag**

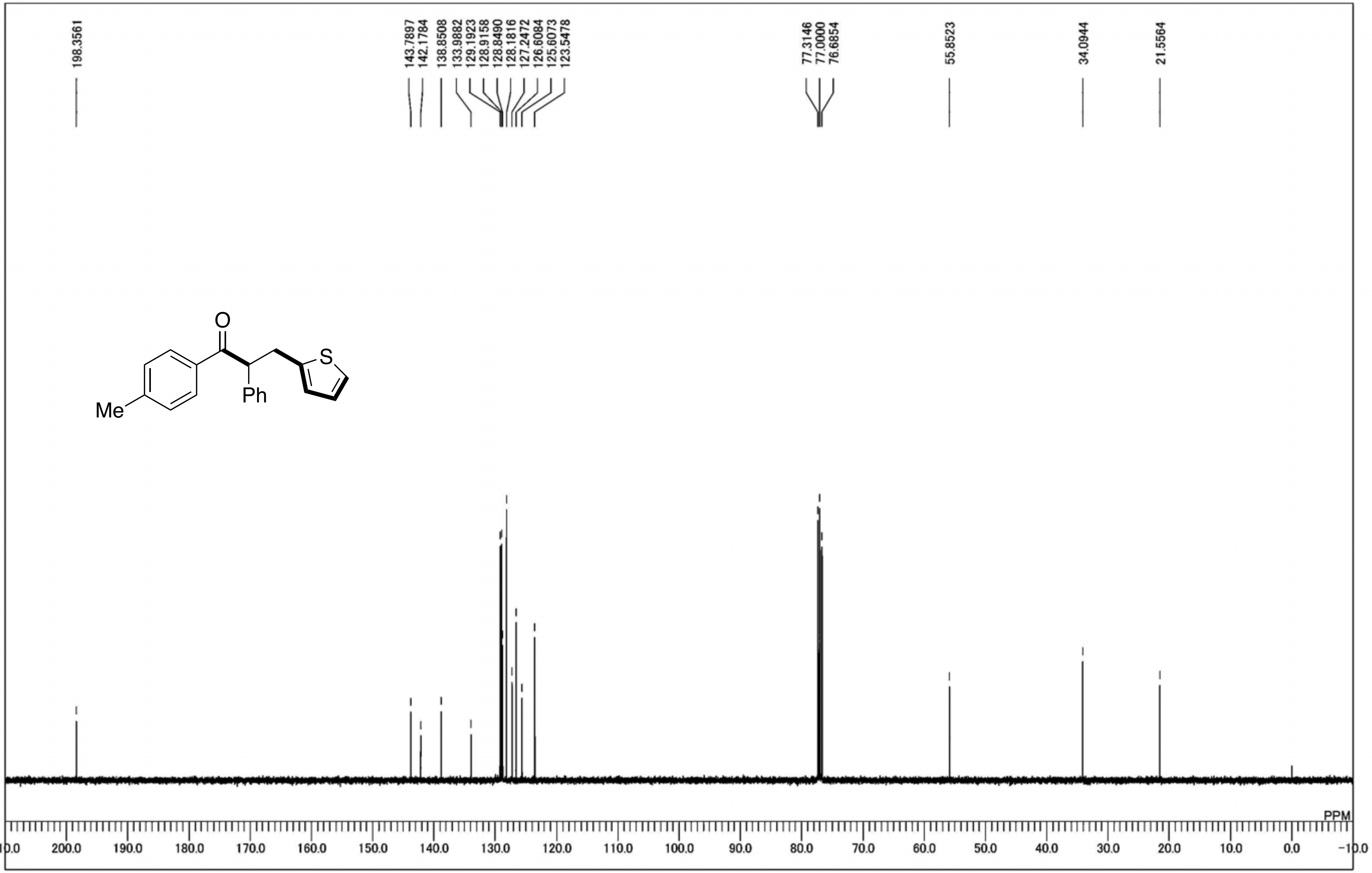

Supplementary Fig. 43 | ^{13}C NMR spectrum of **6bag**

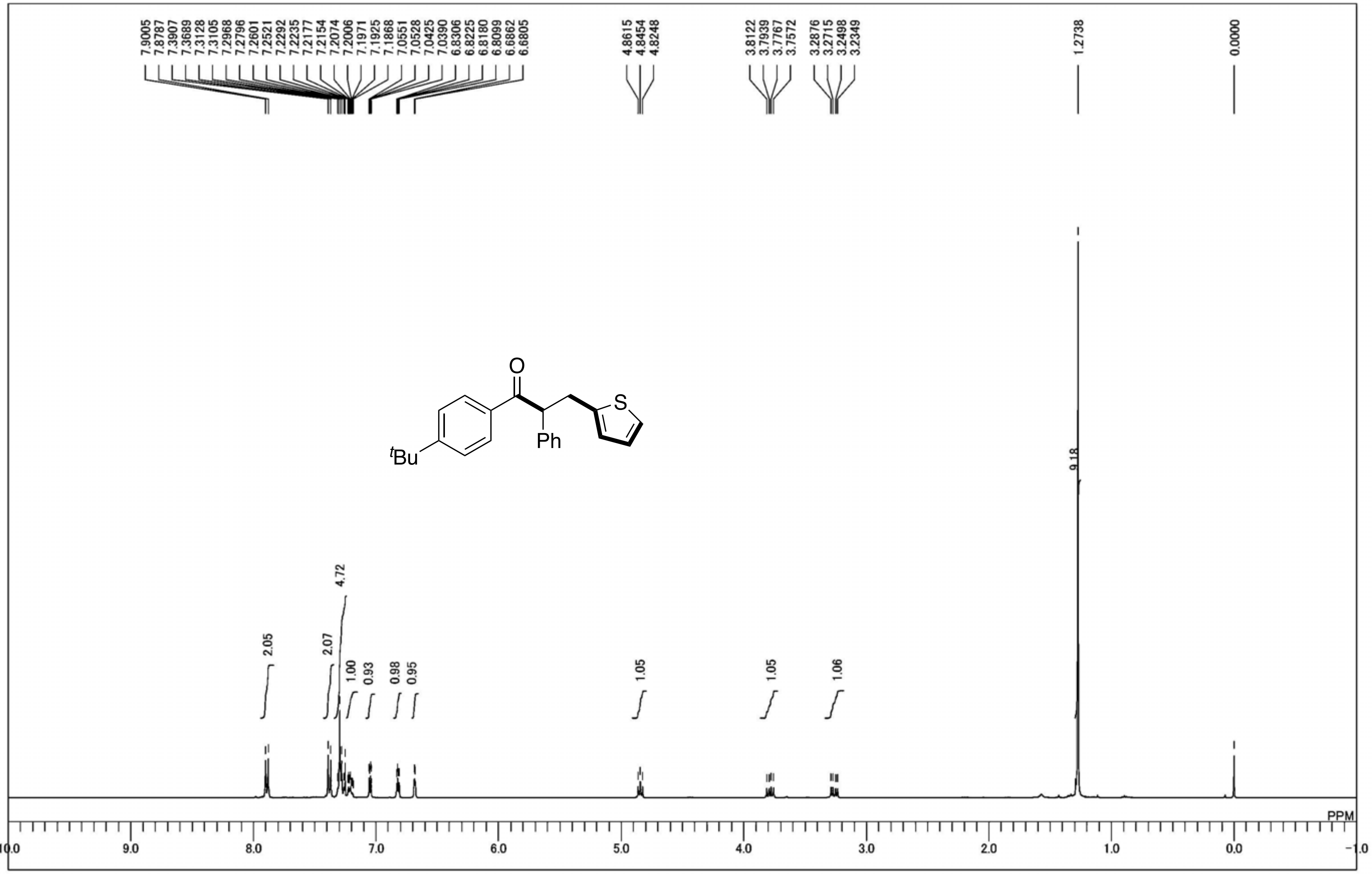

Supplementary Fig. 44 | ^{1}H NMR spectrum of **6cag**

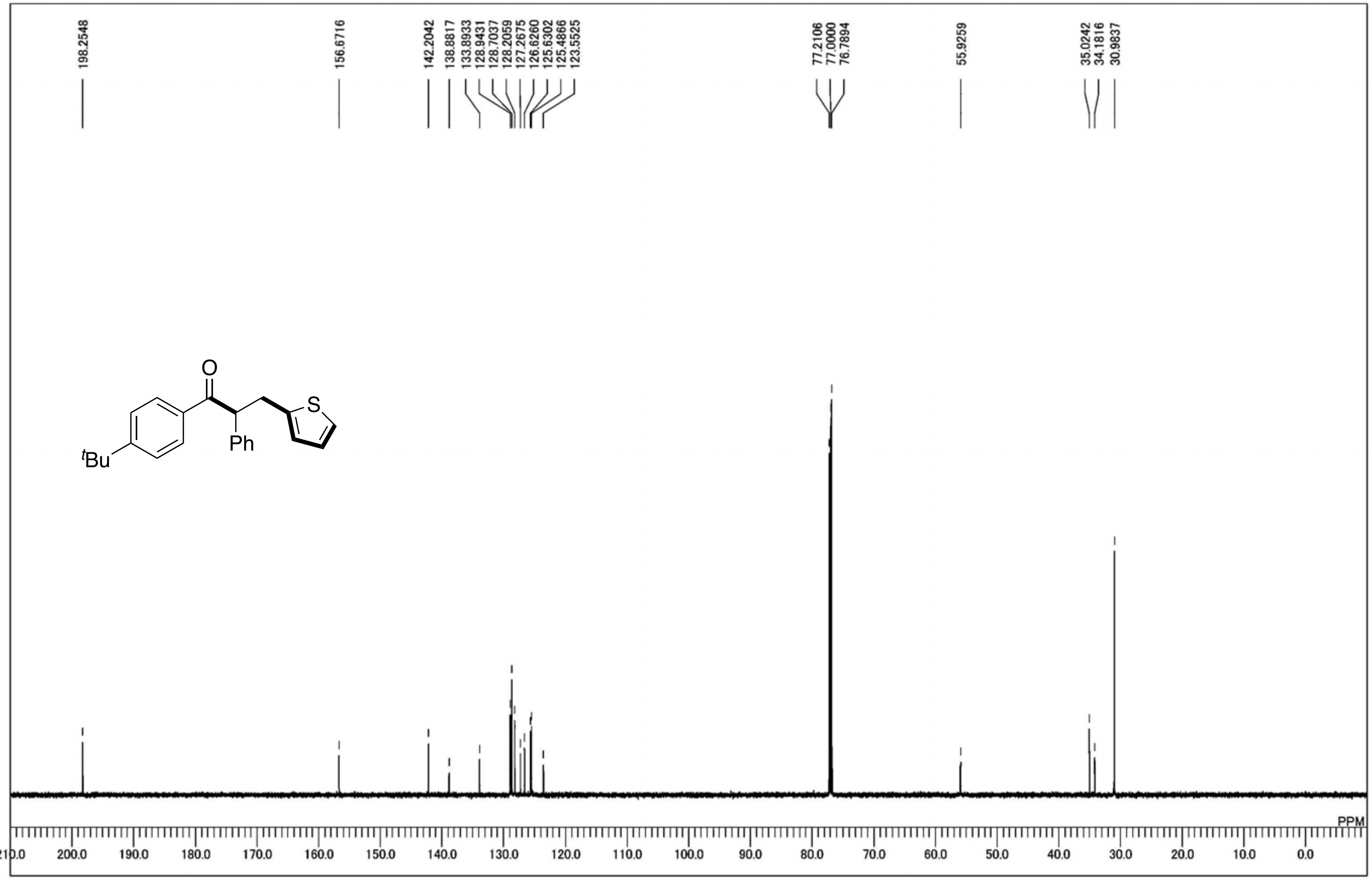

Supplementary Fig. 45 | ^{13}C NMR spectrum of **6cag**

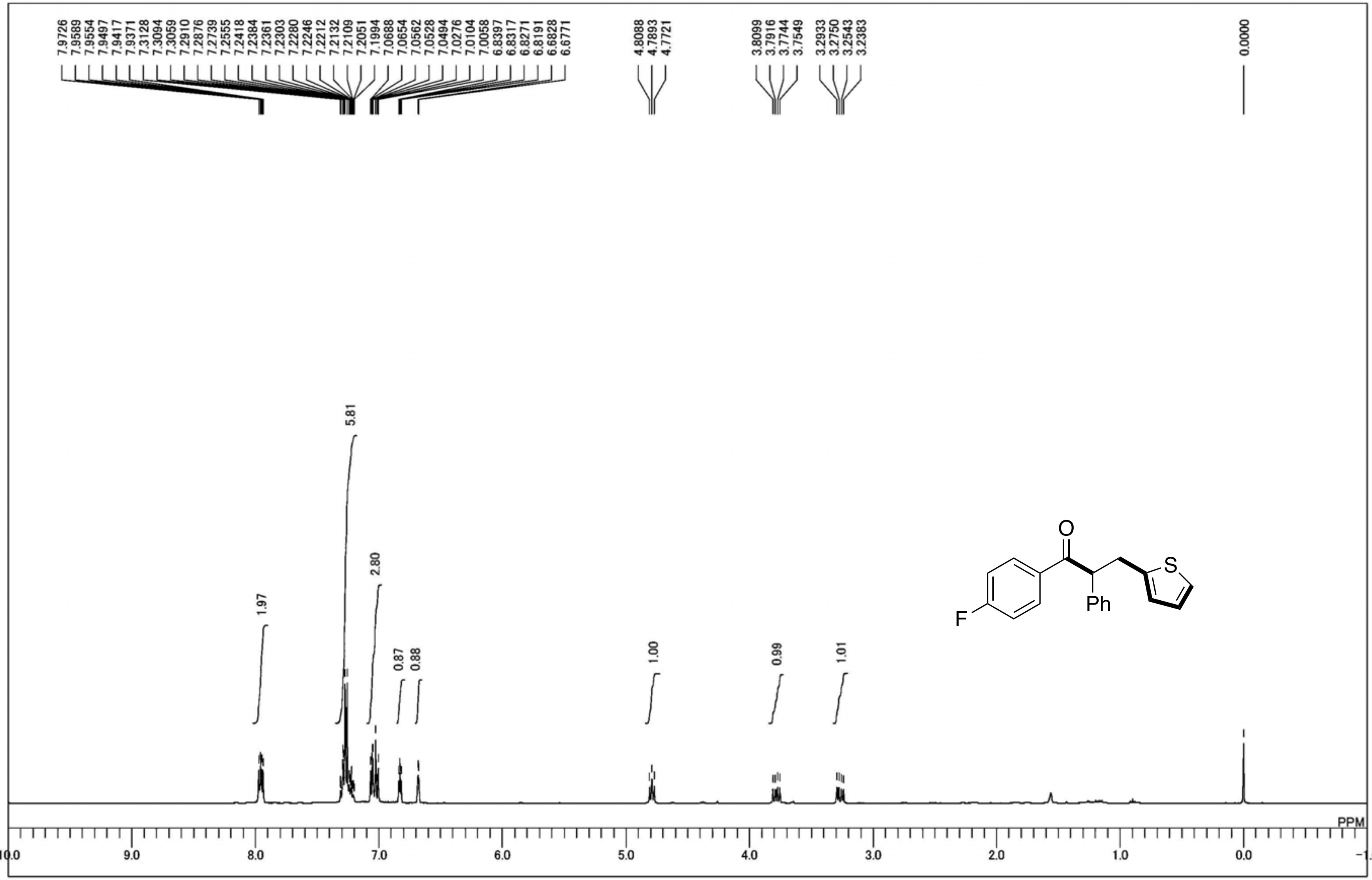

Supplementary Fig. 46 | ^{1}H NMR spectrum of **6dag**

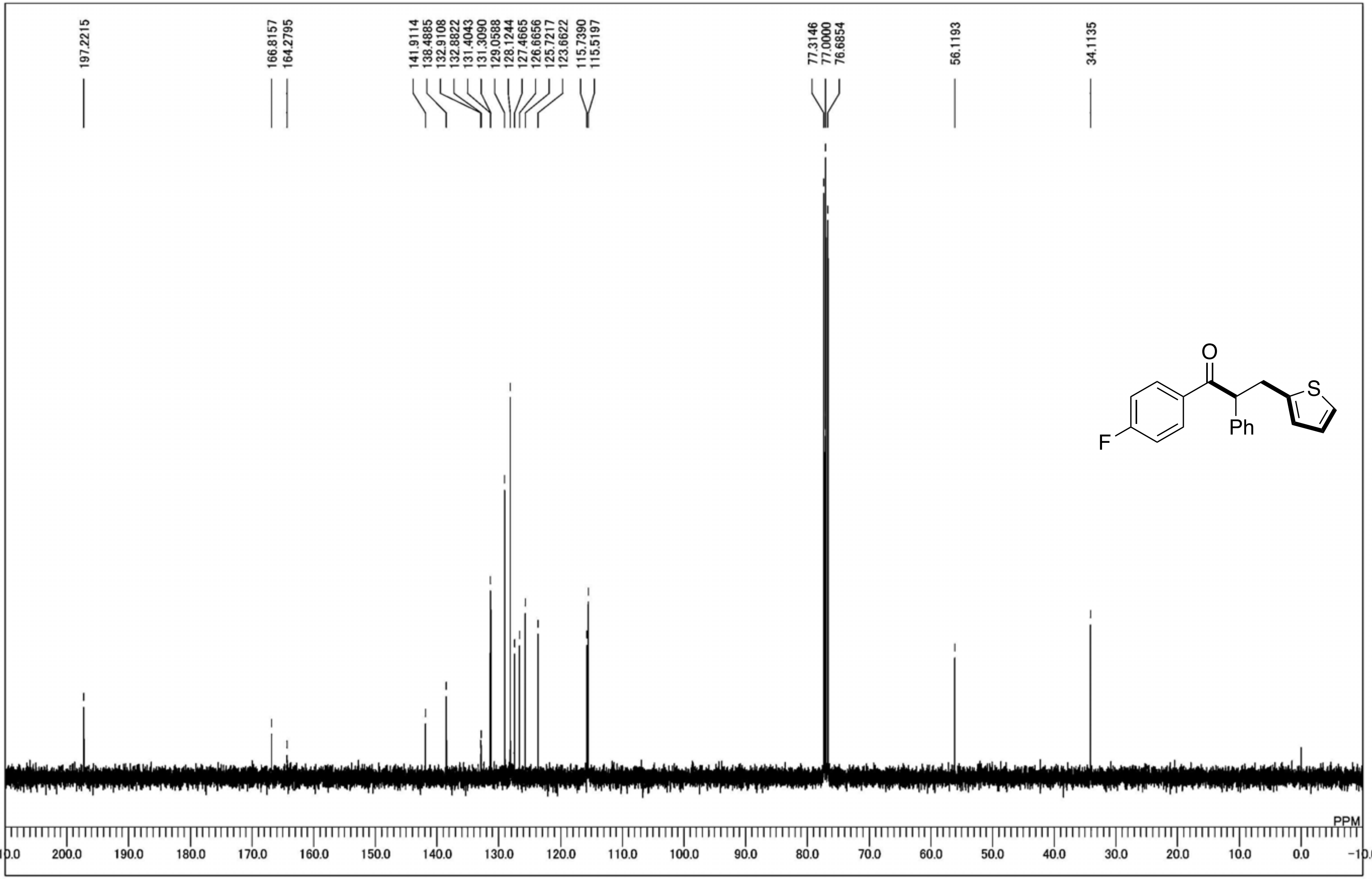

Supplementary Fig. 47 | ^{13}C NMR spectrum of **6dag**

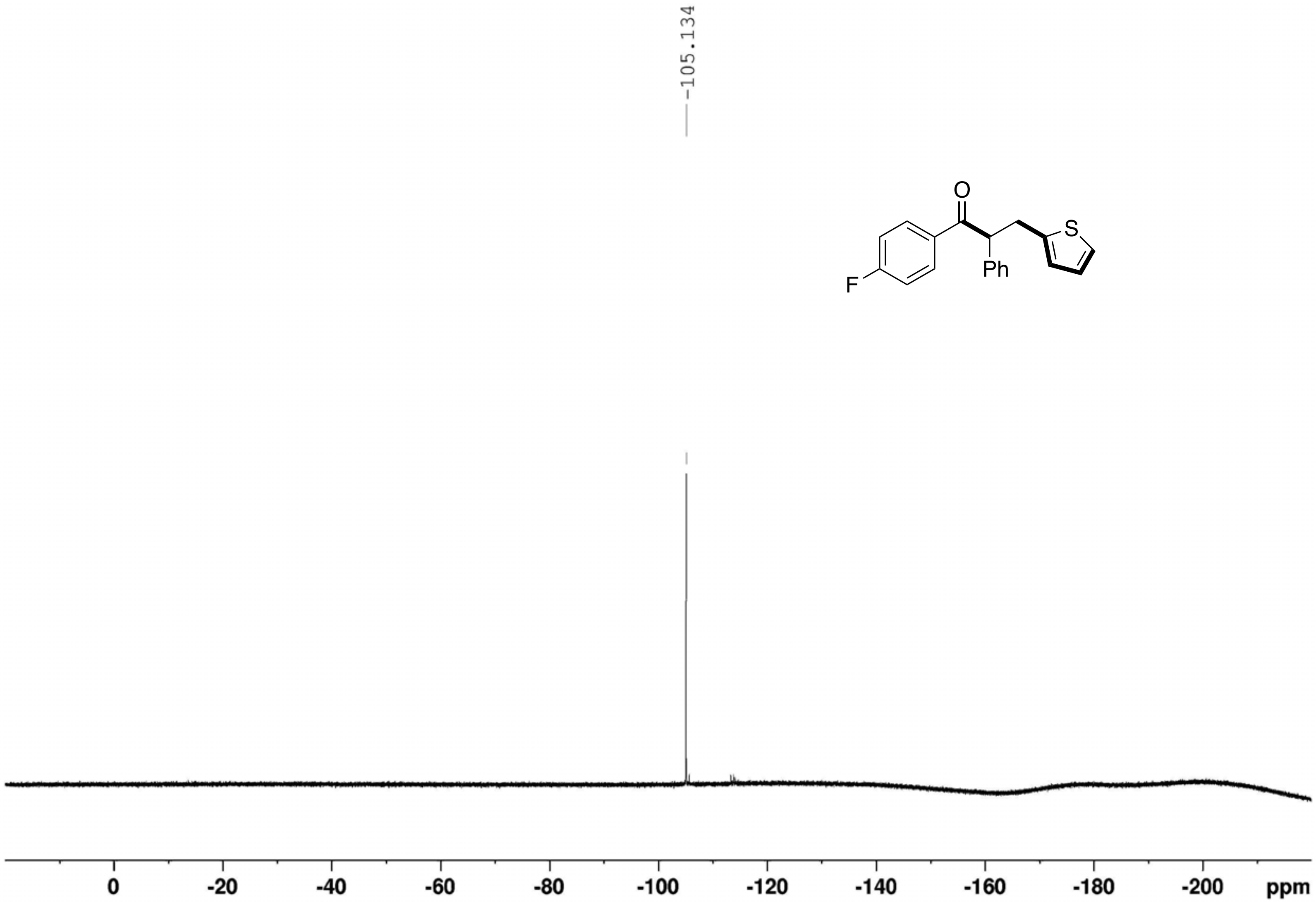

Supplementary Fig. 48 | ^{19}F NMR spectrum of **6dag**

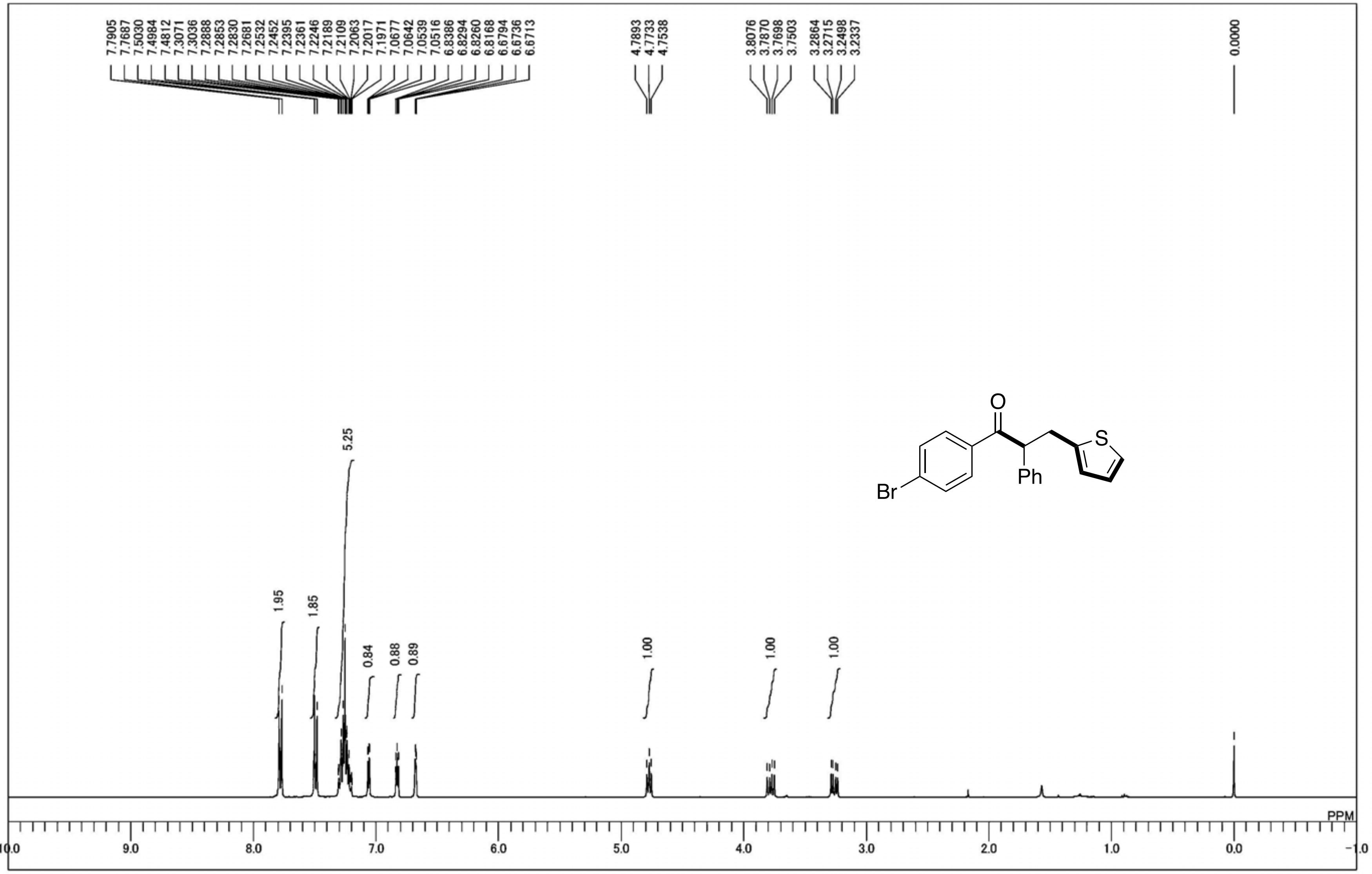

Supplementary Fig. 49 | ^{1}H NMR spectrum of **6eag**

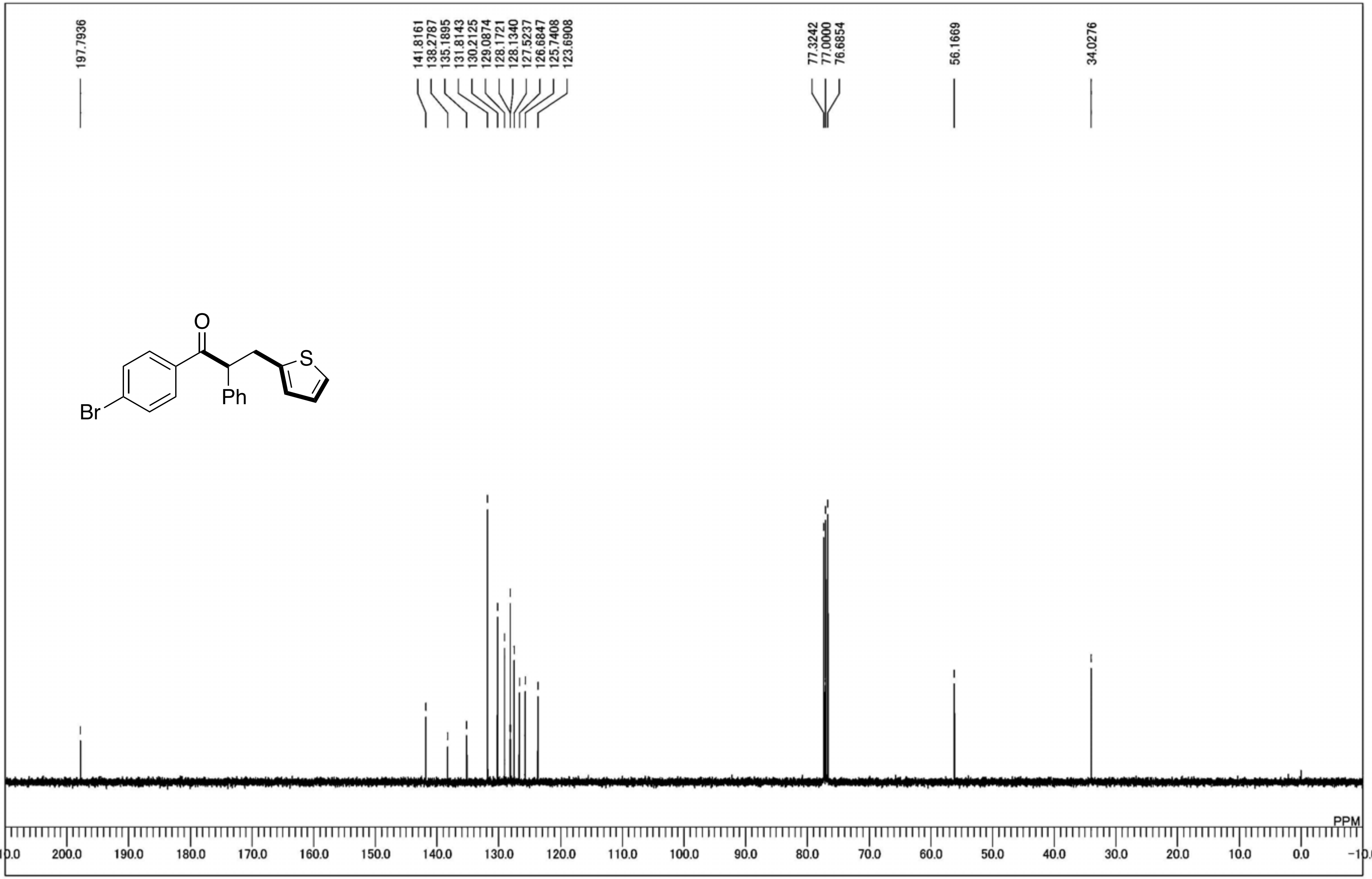

Supplementary Fig. 50 | ^{13}C NMR spectrum of **6eag**

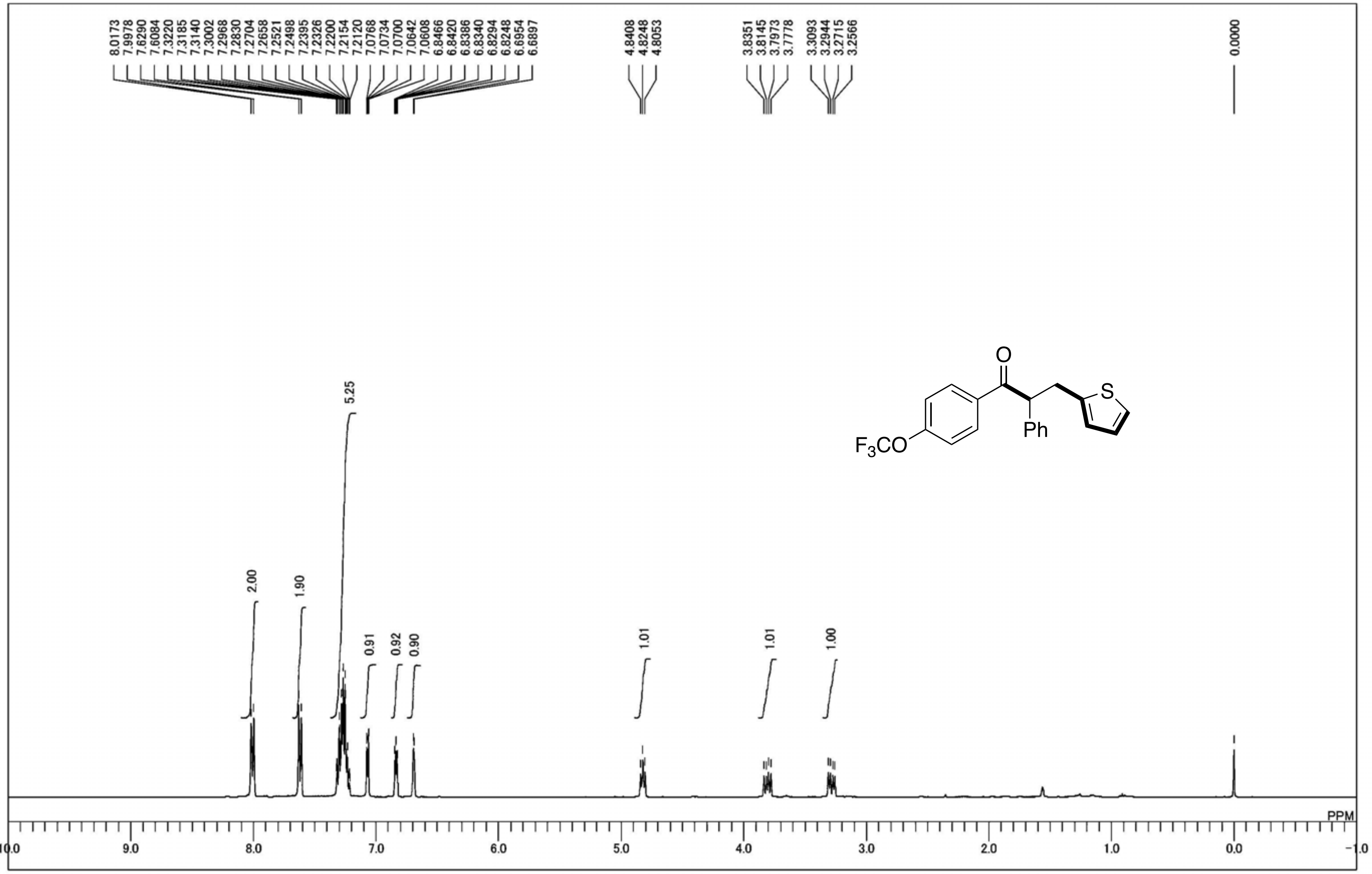

Supplementary Fig. 51 | ^{1}H NMR spectrum of **6fag**

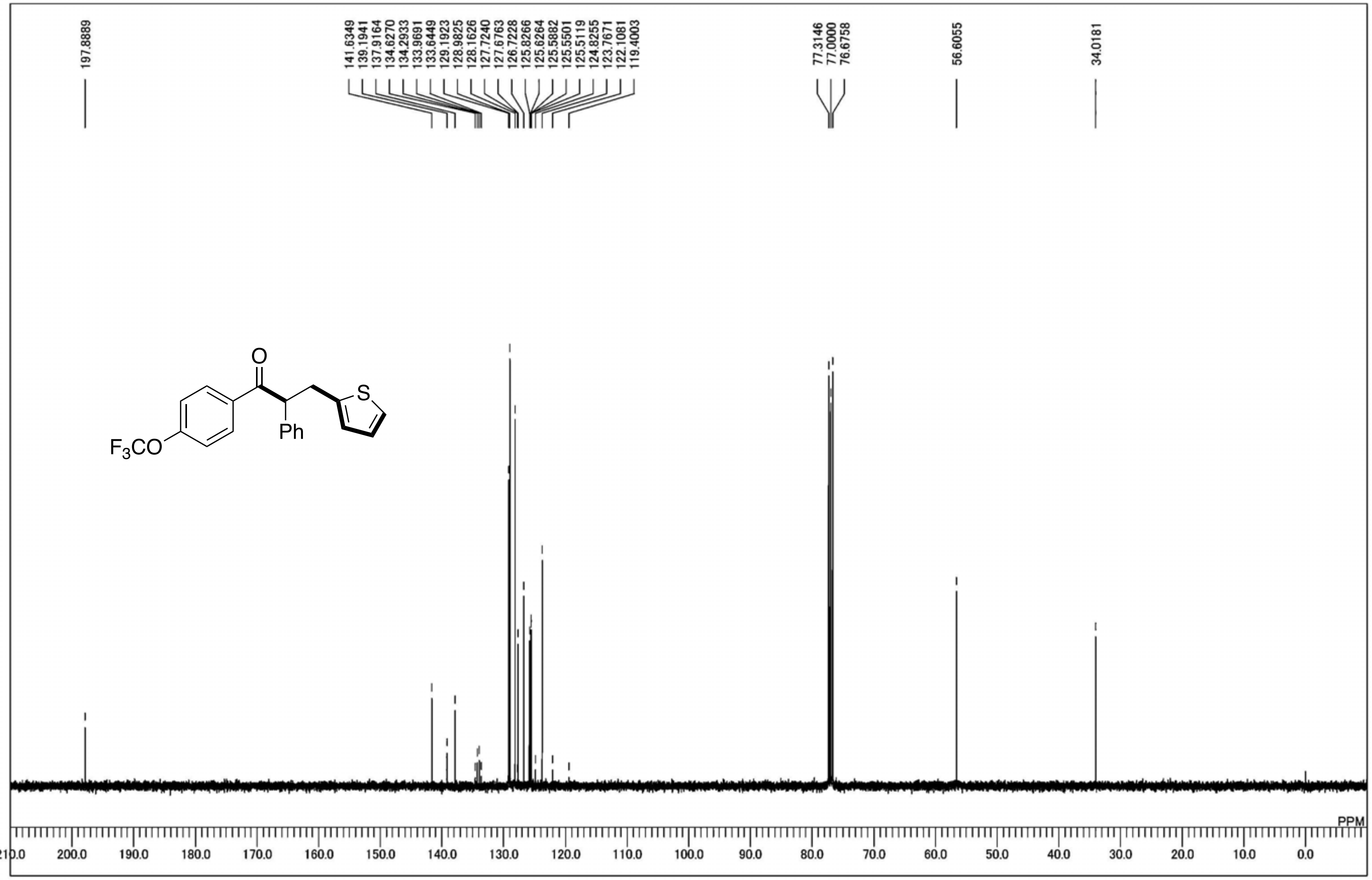

Supplementary Fig. 52 | ^{13}C NMR spectrum of **6fag**

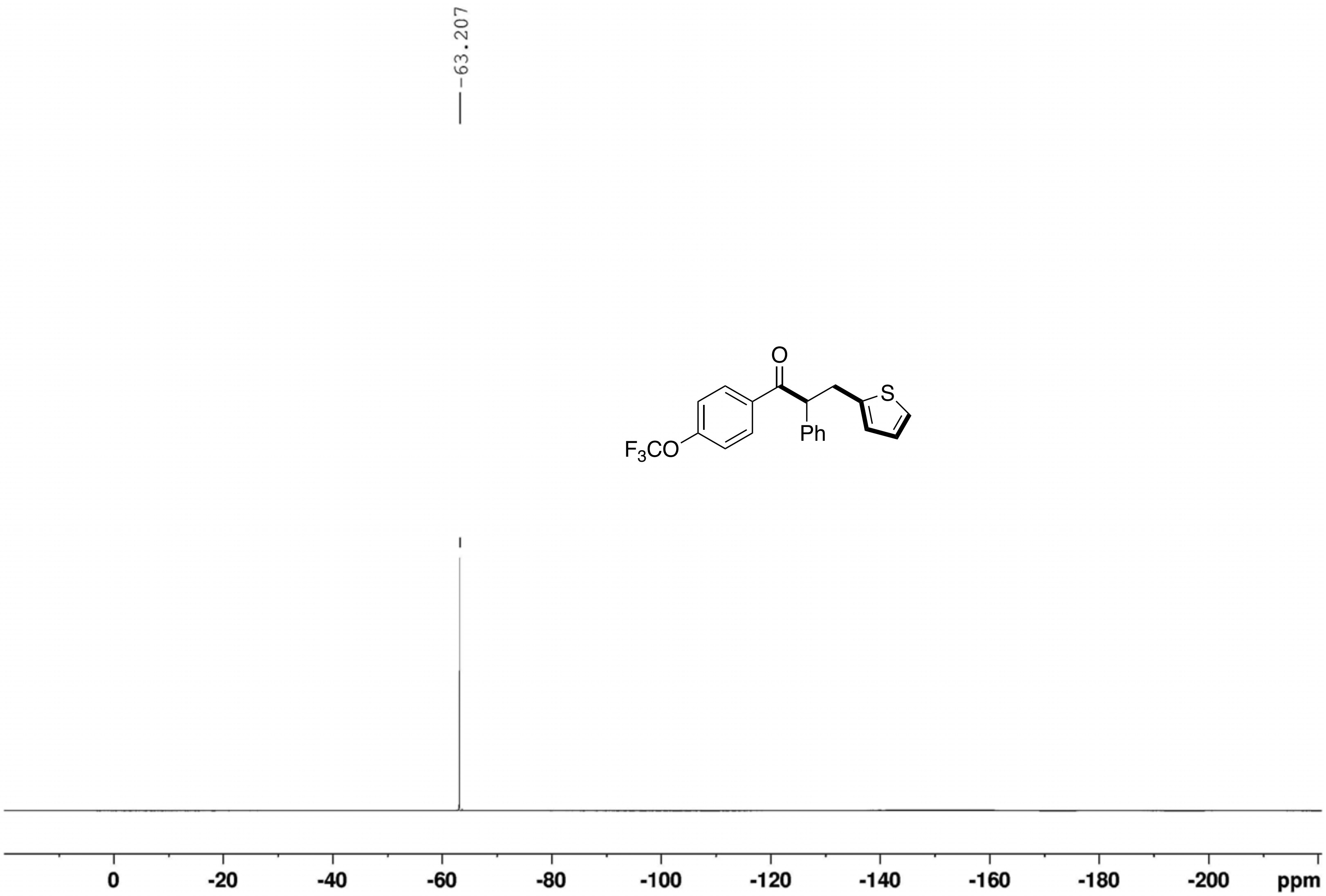

Supplementary Fig. 53 | ^{19}F NMR spectrum of **6fag**

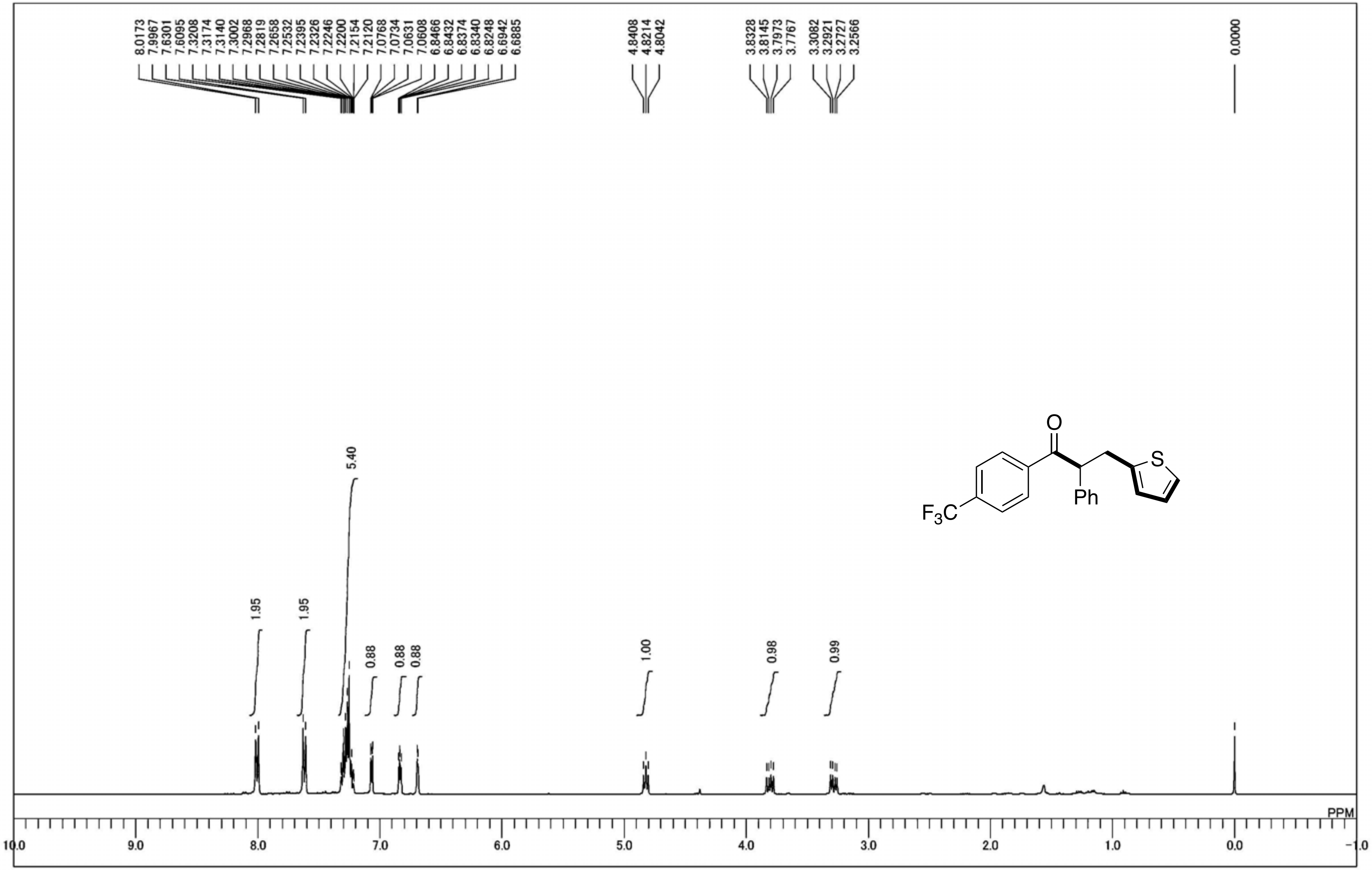

Supplementary Fig. 54 | ^{1}H NMR spectrum of **6gag**

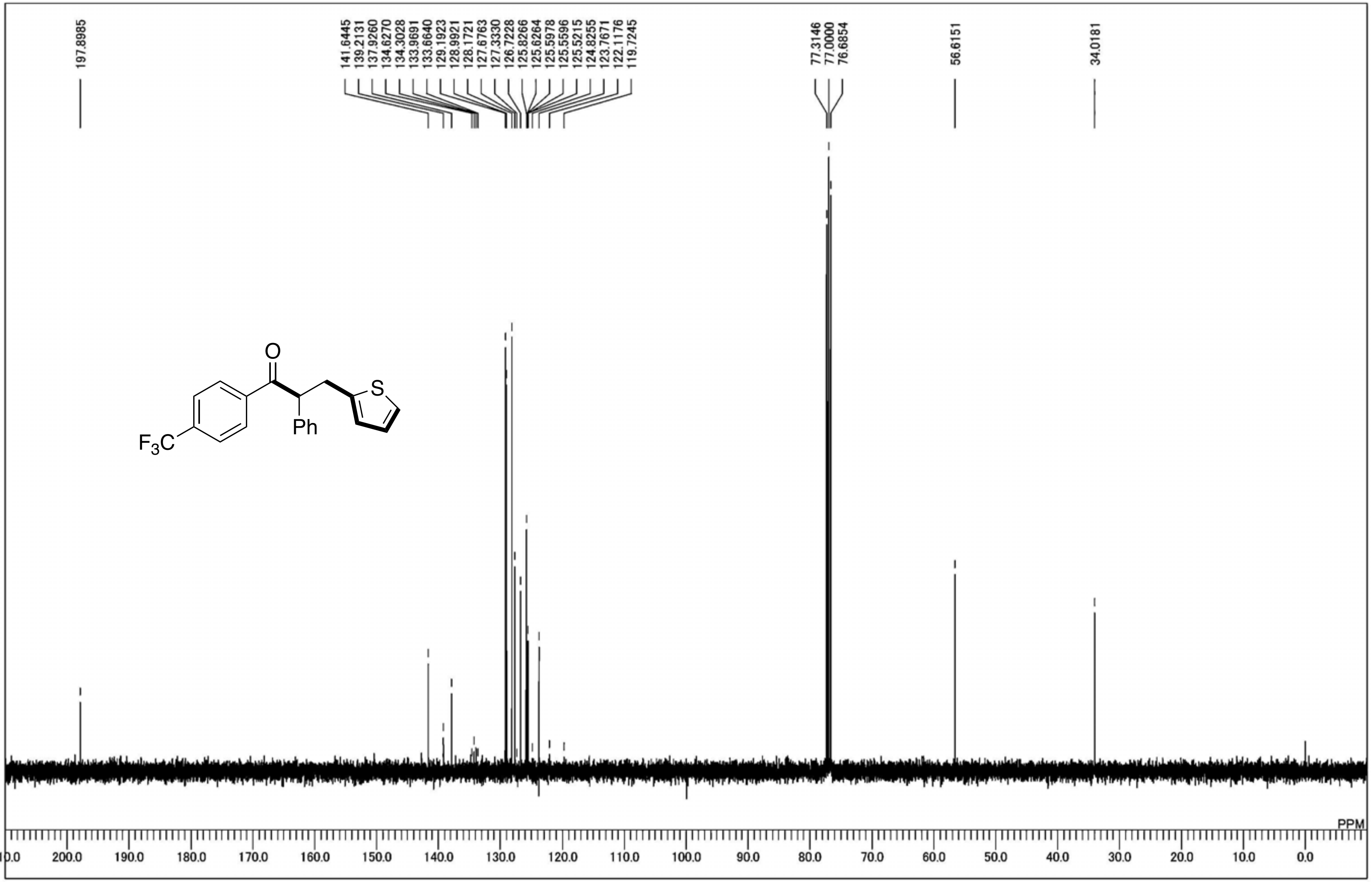

Supplementary Fig. 55 | ^{13}C NMR spectrum of **6gag**

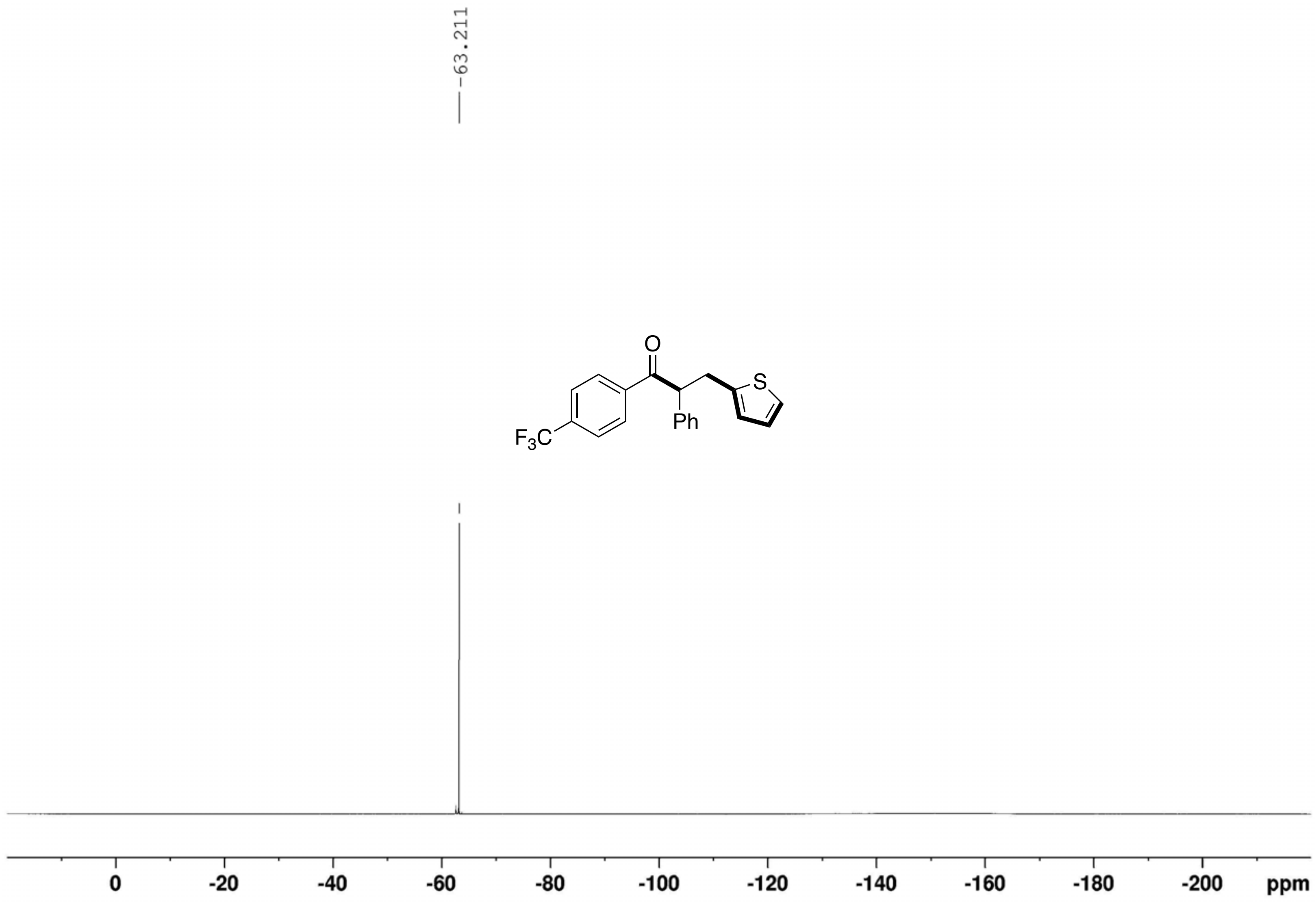

Supplementary Fig. 56 | ^{19}F NMR spectrum of **6gag**

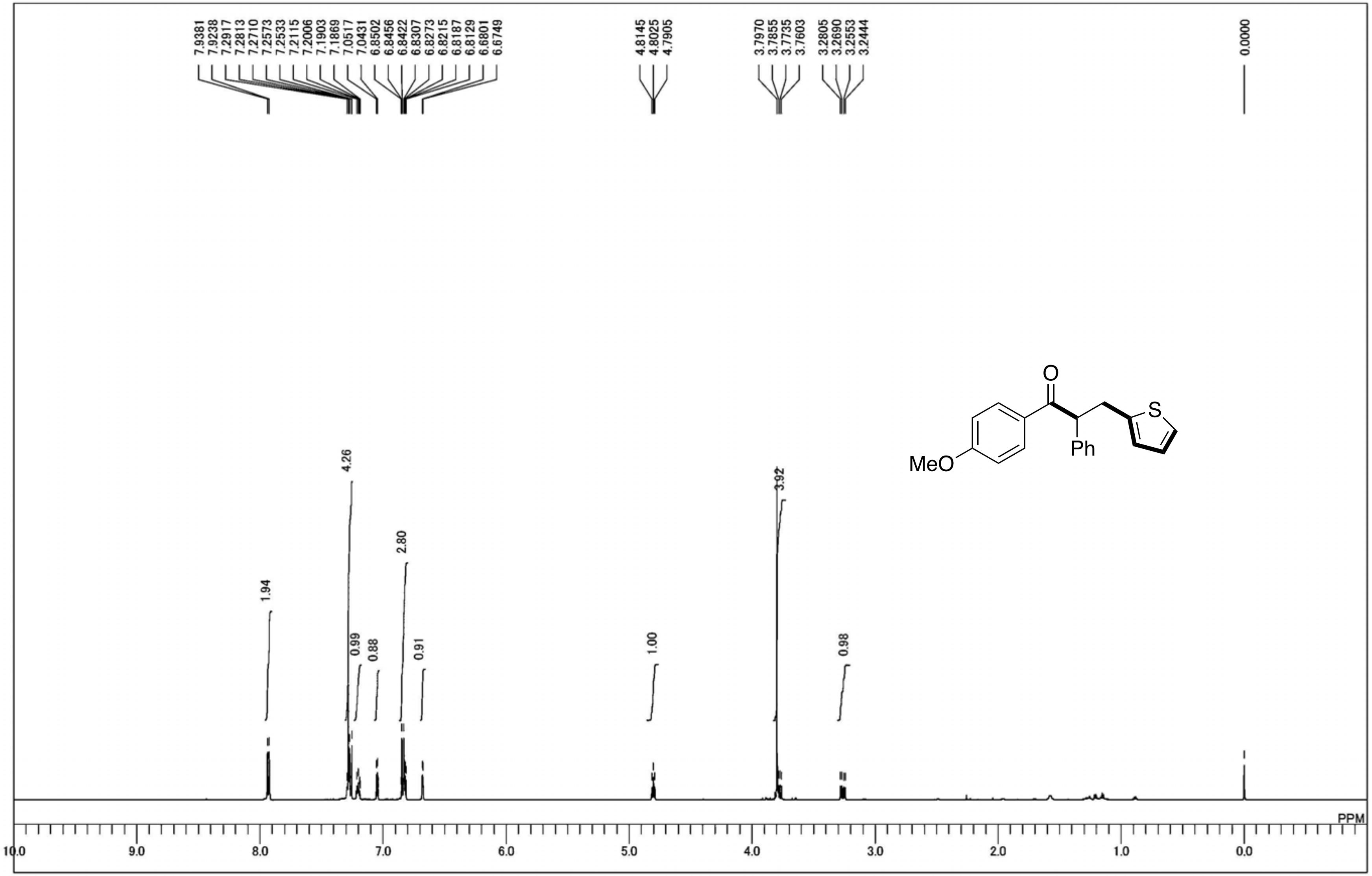

Supplementary Fig. 57 | ^{1}H NMR spectrum of **6hag**

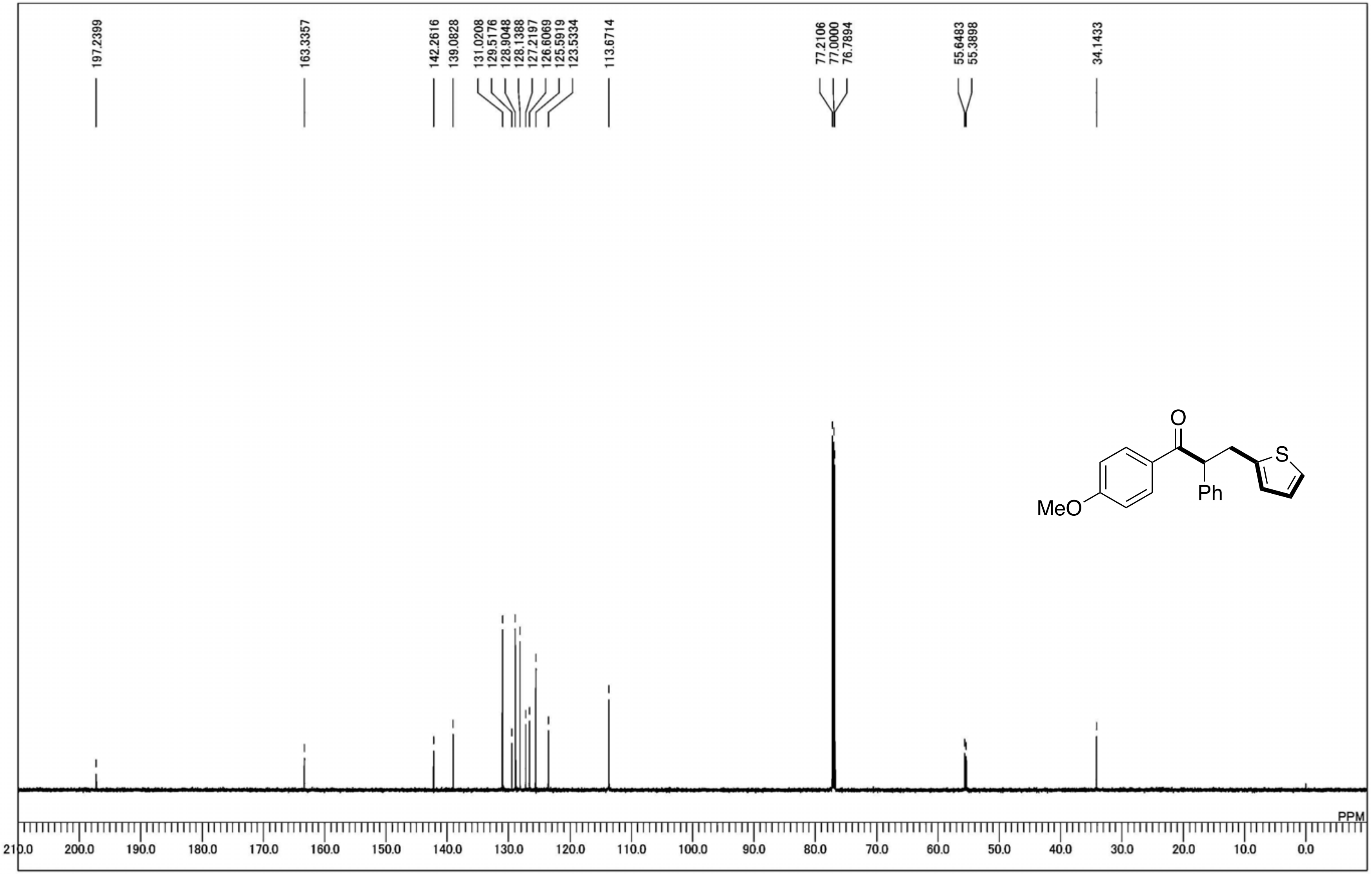

Supplementary Fig. 58 | ^{13}C NMR spectrum of **6hag**

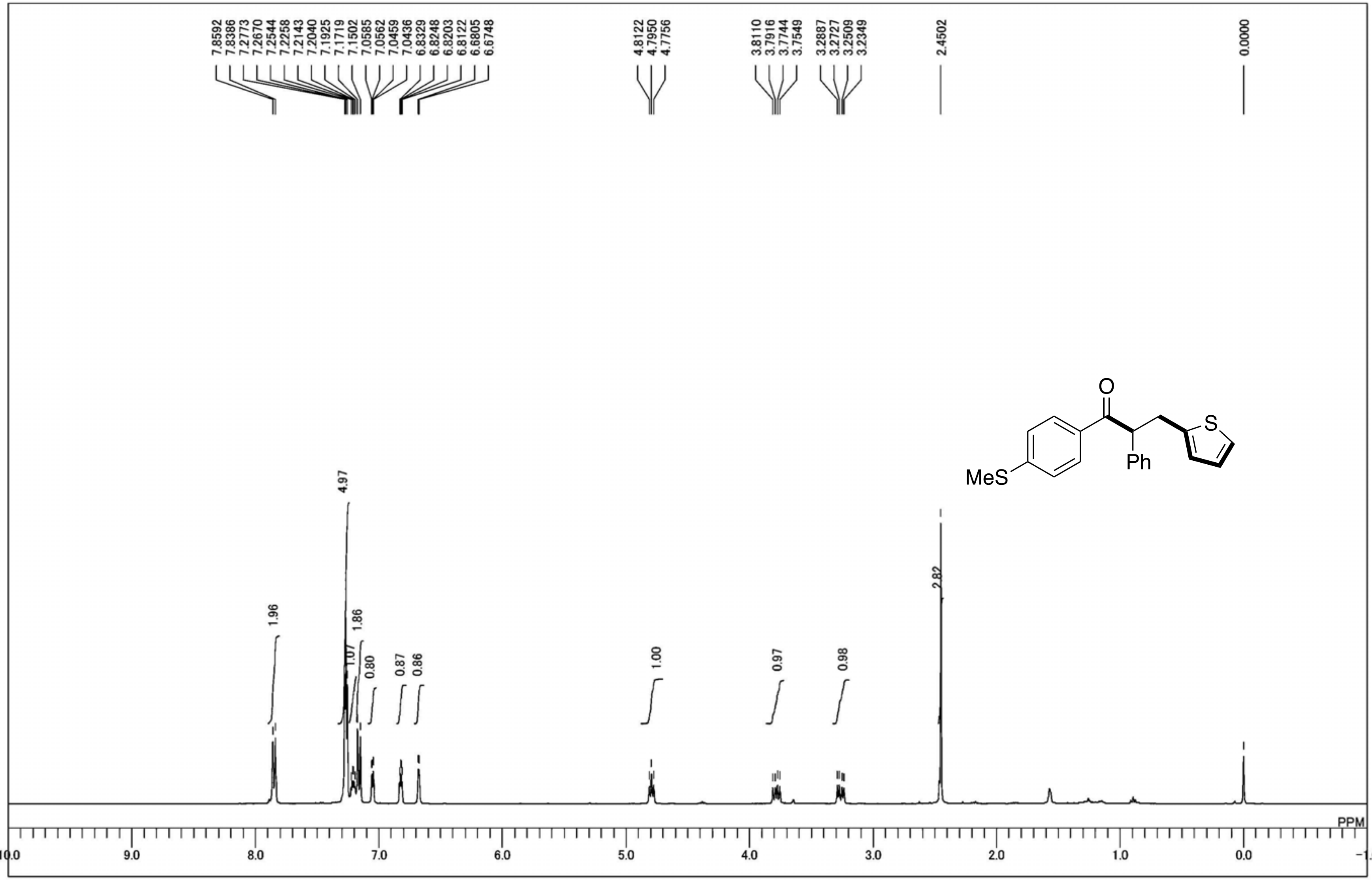

Supplementary Fig. 59 | ^{1}H NMR spectrum of **6iag**

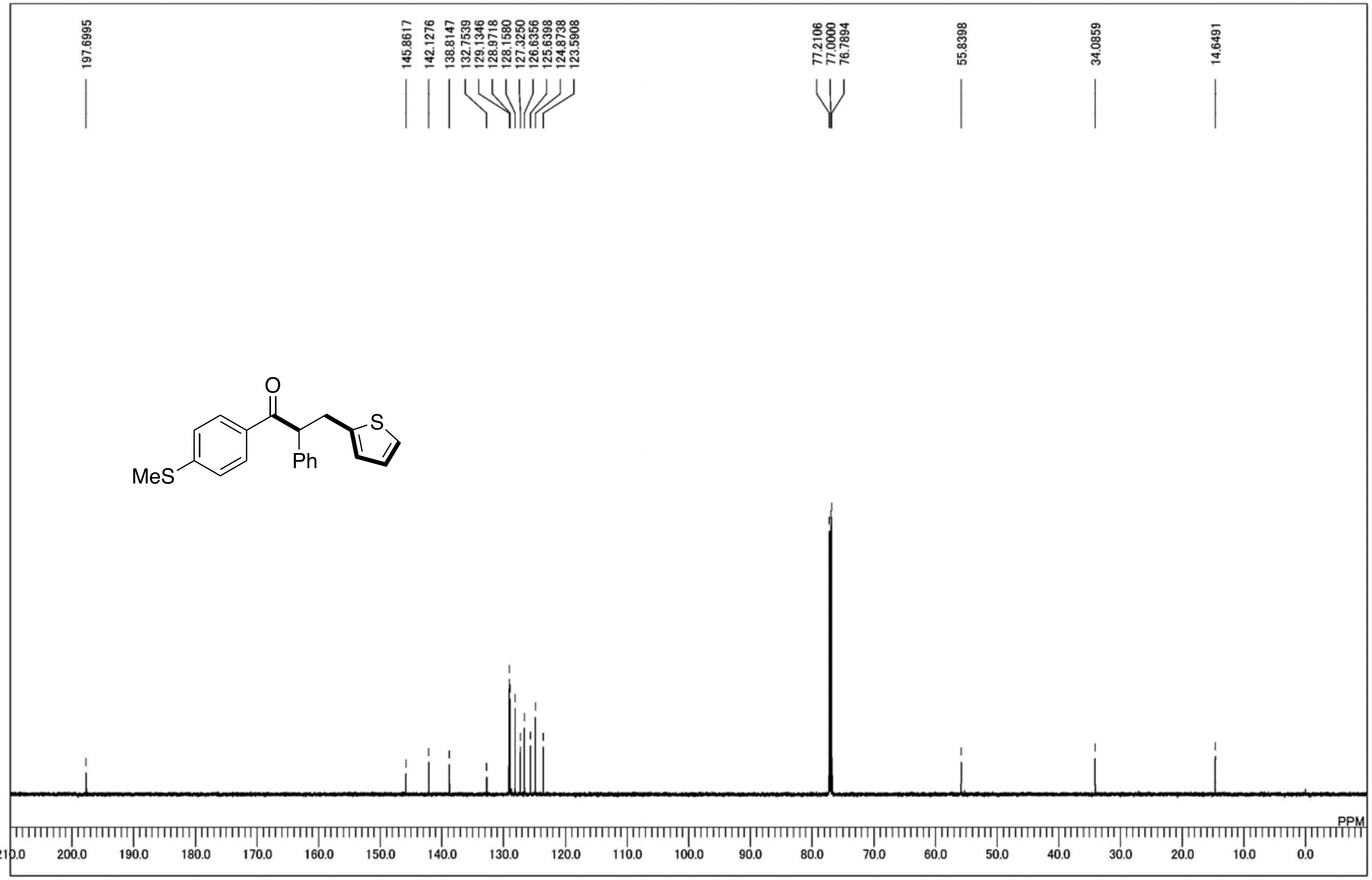

Supplementary Fig. 60 | ^{13}C NMR spectrum of **6iag**

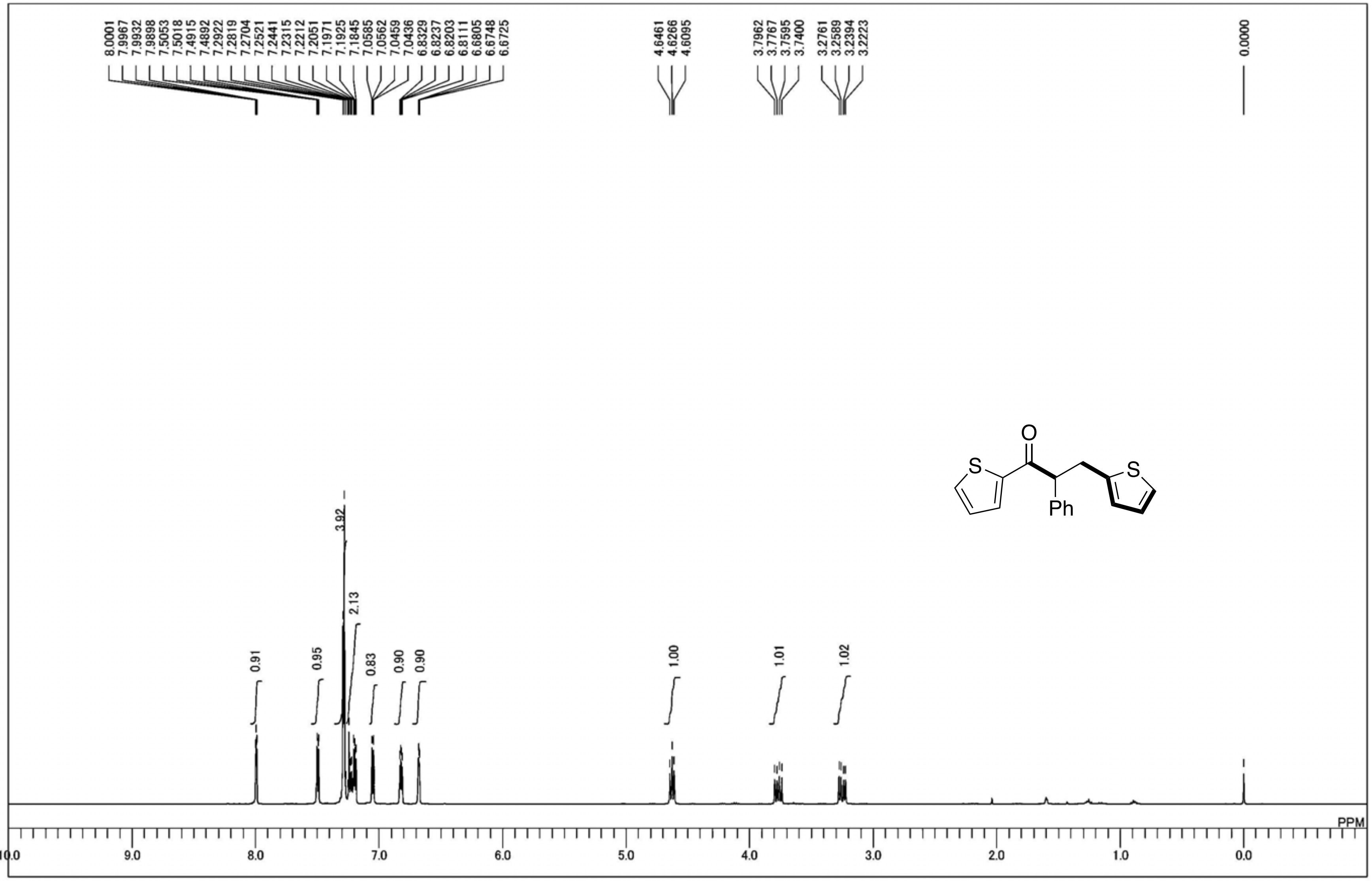

Supplementary Fig. 61 | ^{1}H NMR spectrum of **6jag**

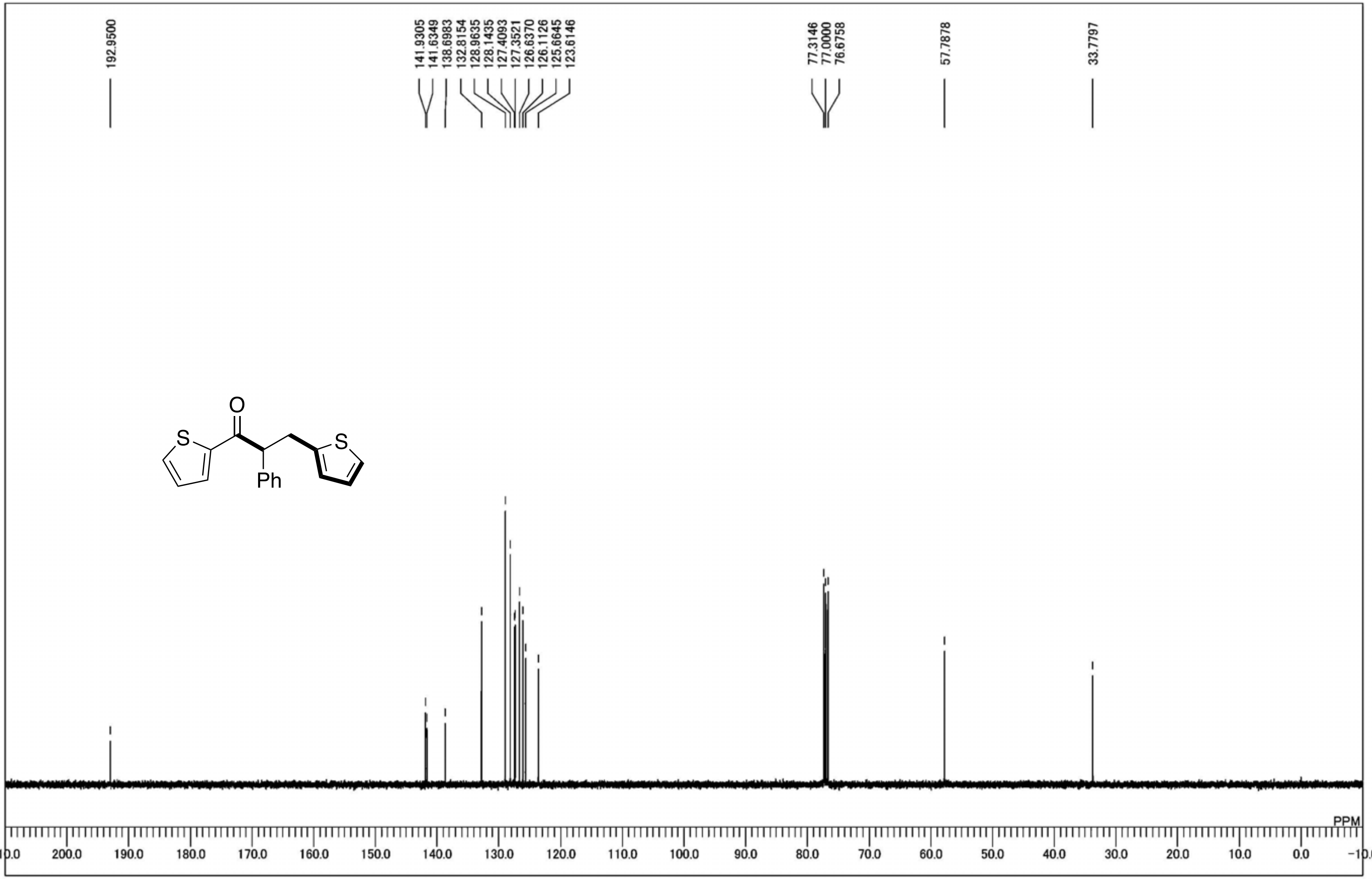

Supplementary Fig. 62 | ^{13}C NMR spectrum of **6jag**

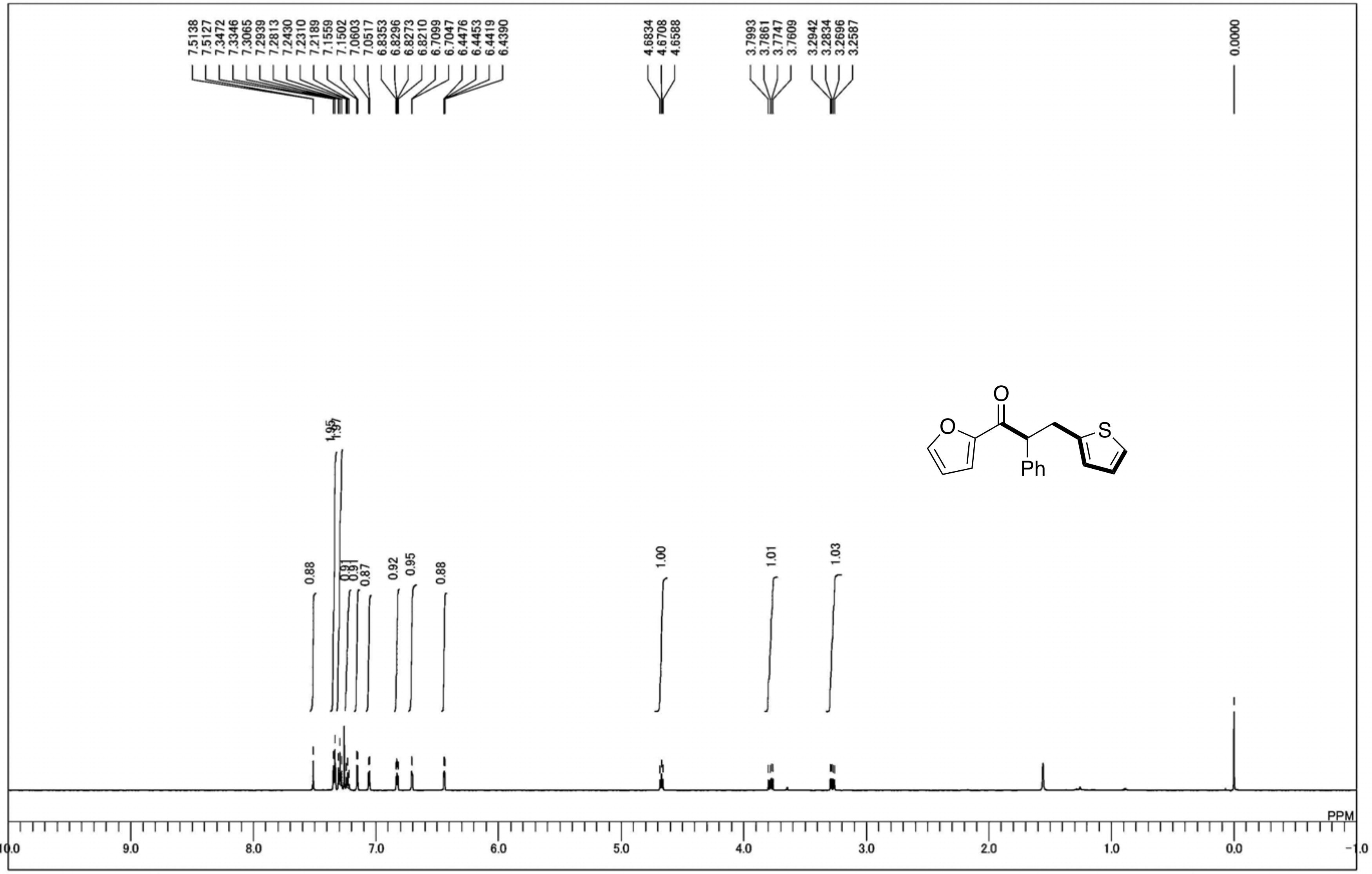

Supplementary Fig. 63 | ^{1}H NMR spectrum of **6kag**

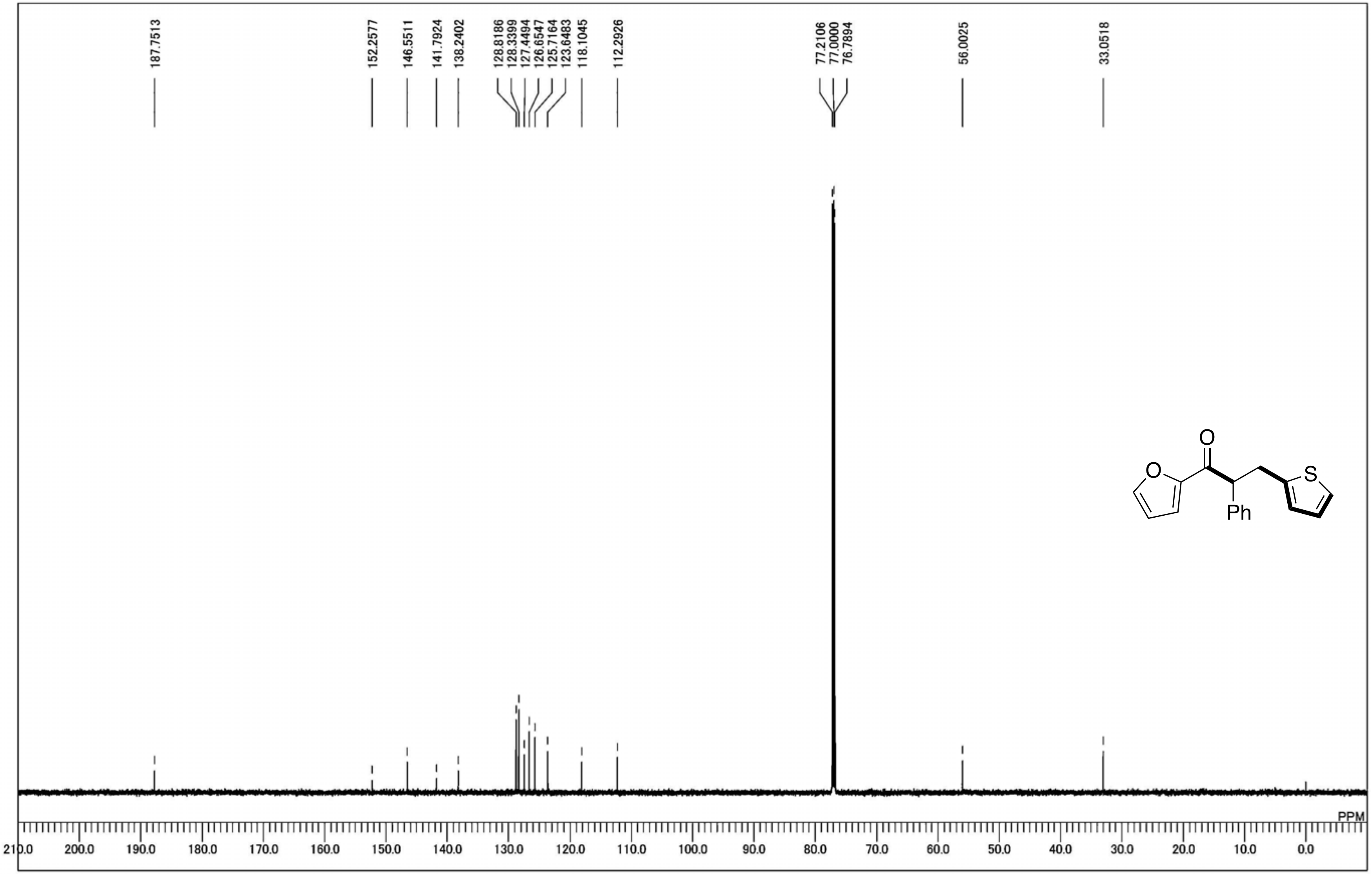

Supplementary Fig. 64 | ^{13}C NMR spectrum of **6kag**

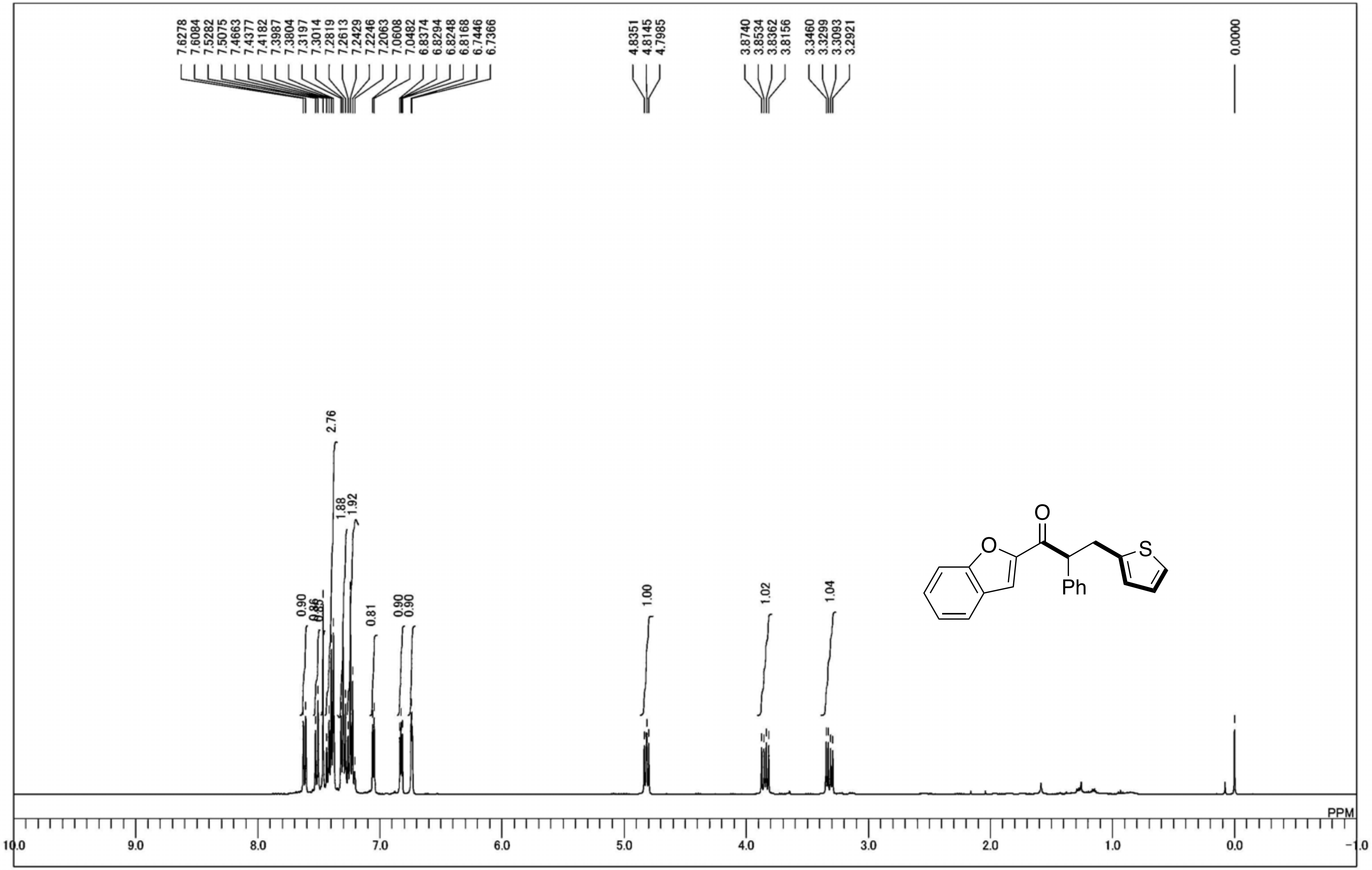

Supplementary Fig. 65 | ^{1}H NMR spectrum of **6lag**

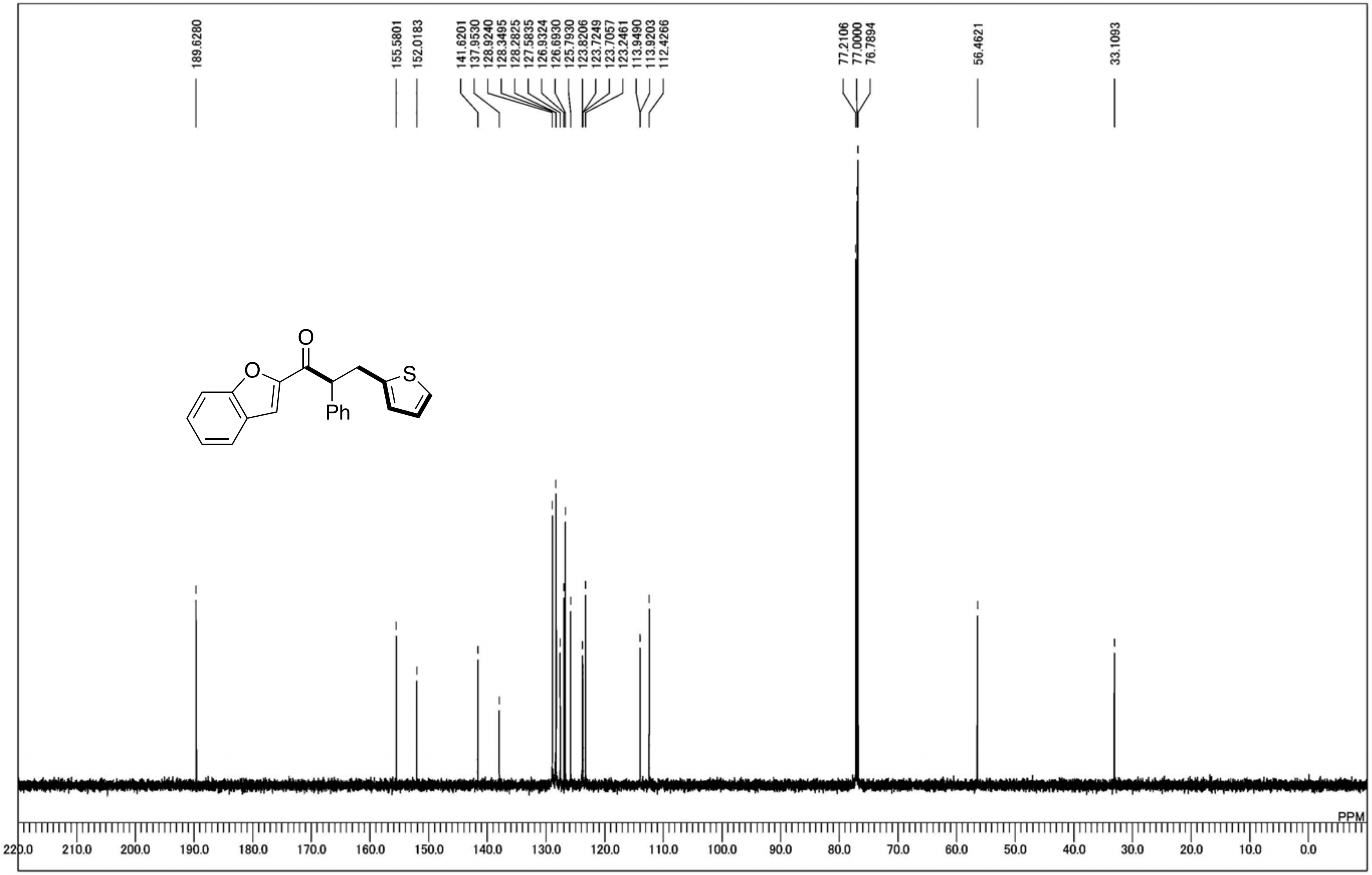

Supplementary Fig. 66 | ^{13}C NMR spectrum of **6lag**

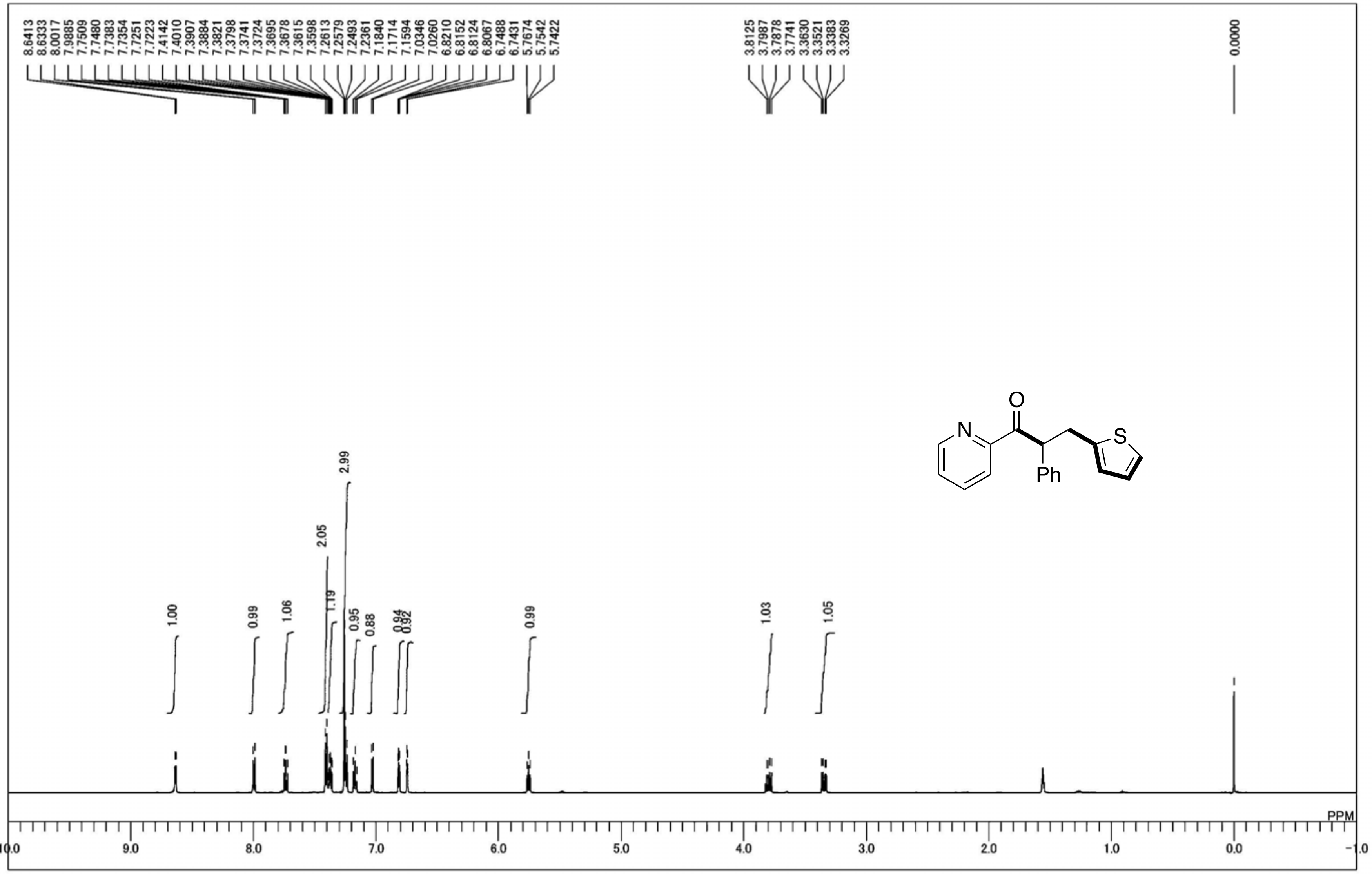

Supplementary Fig. 67 | ^{1}H NMR spectrum of **6mag**

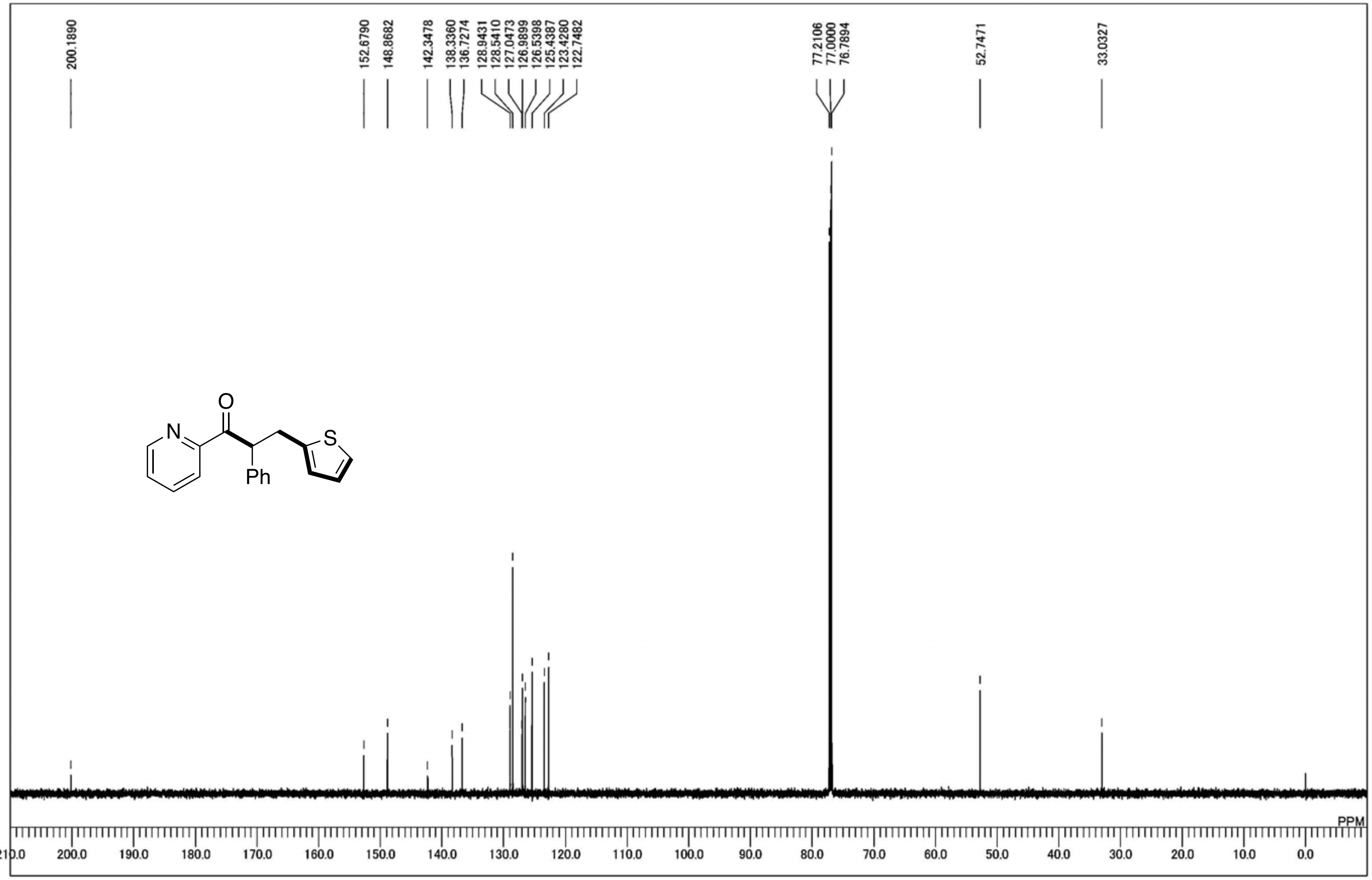

Supplementary Fig. 68 | ^{13}C NMR spectrum of **6mag**

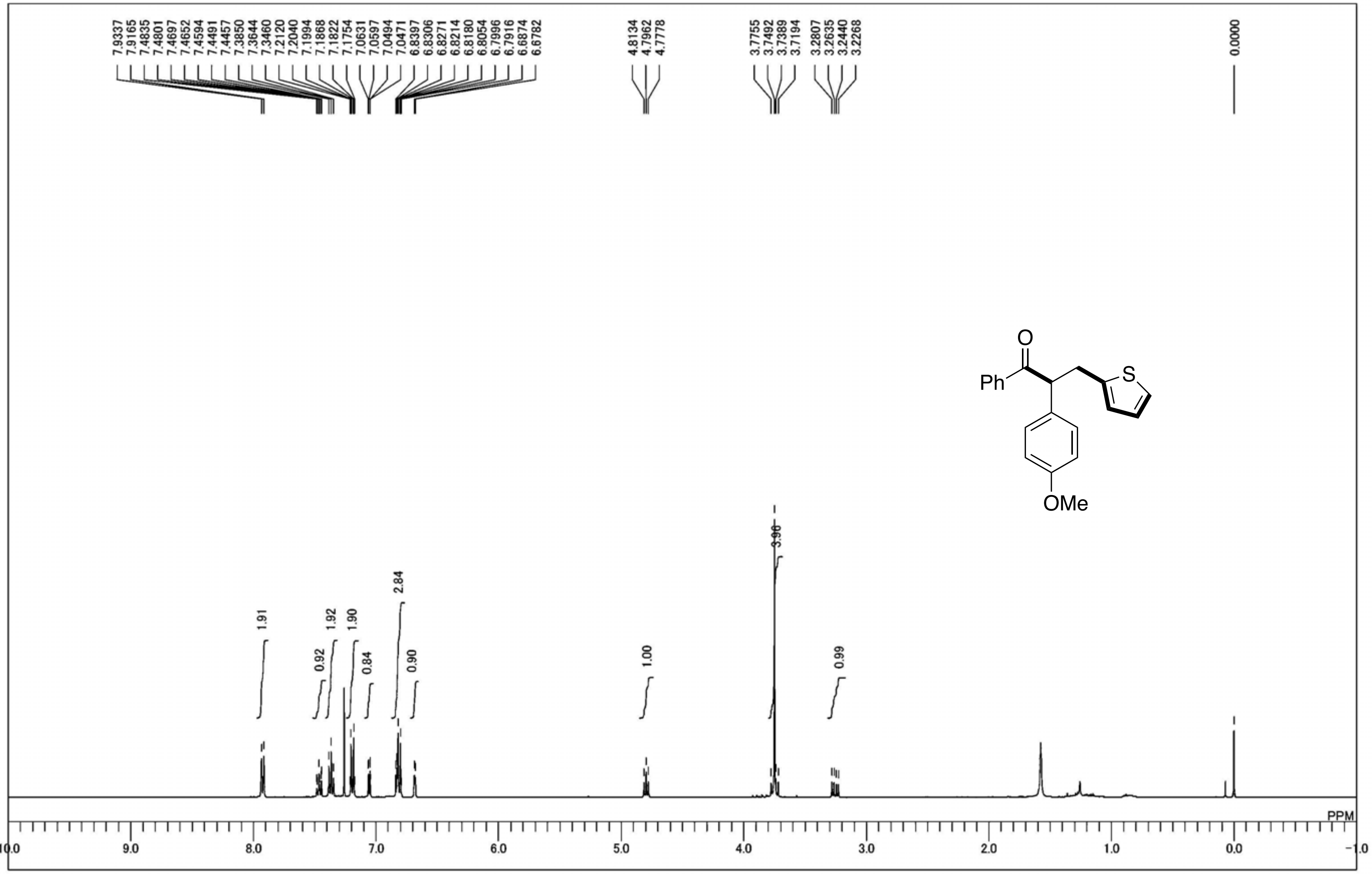

Supplementary Fig. 69 | ^{1}H NMR spectrum of **6abg**

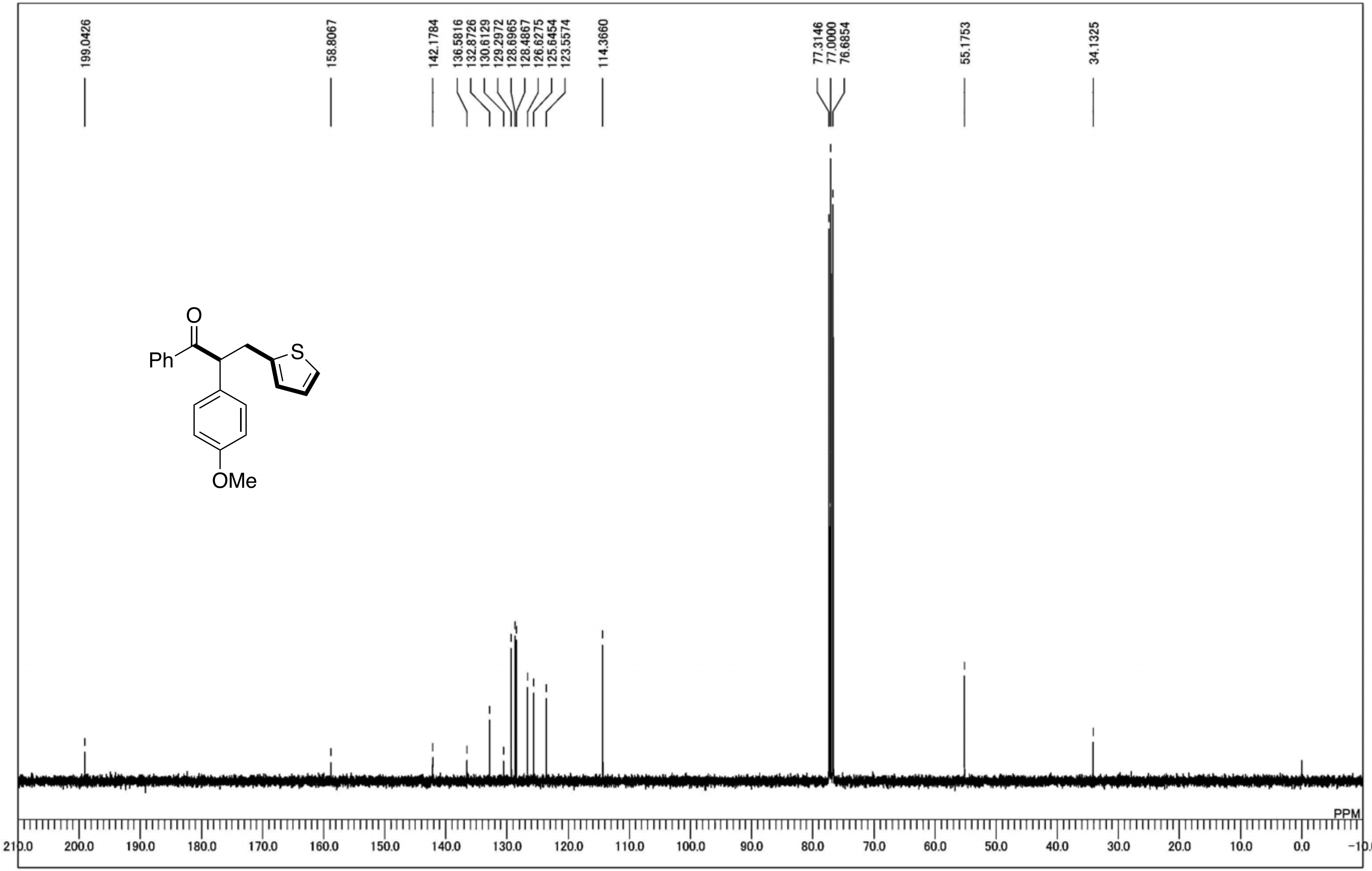

Supplementary Fig. 70 | ^{13}C NMR spectrum of **6abg**

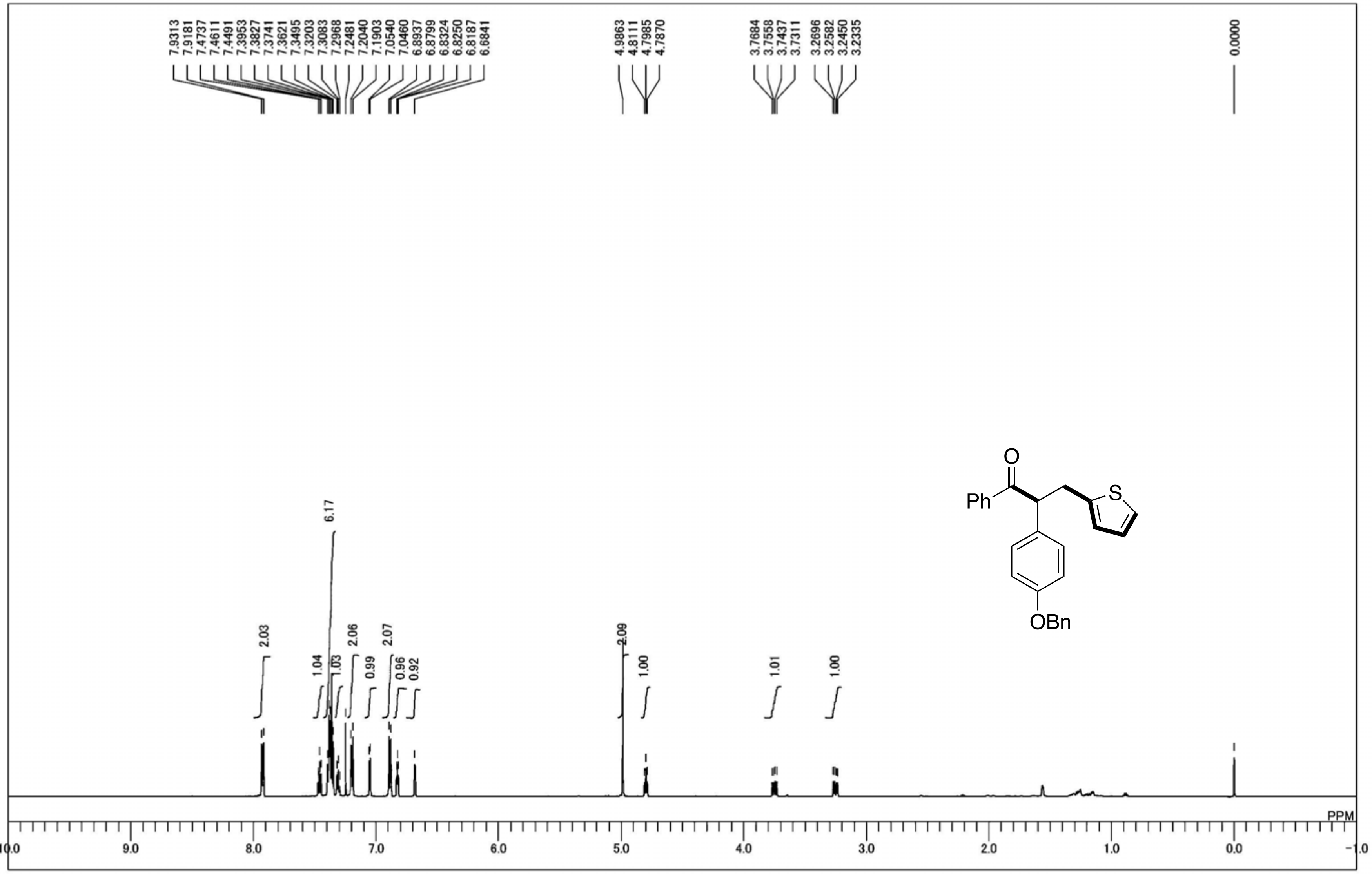

Supplementary Fig. 71 | ^{1}H NMR spectrum of **6acg**

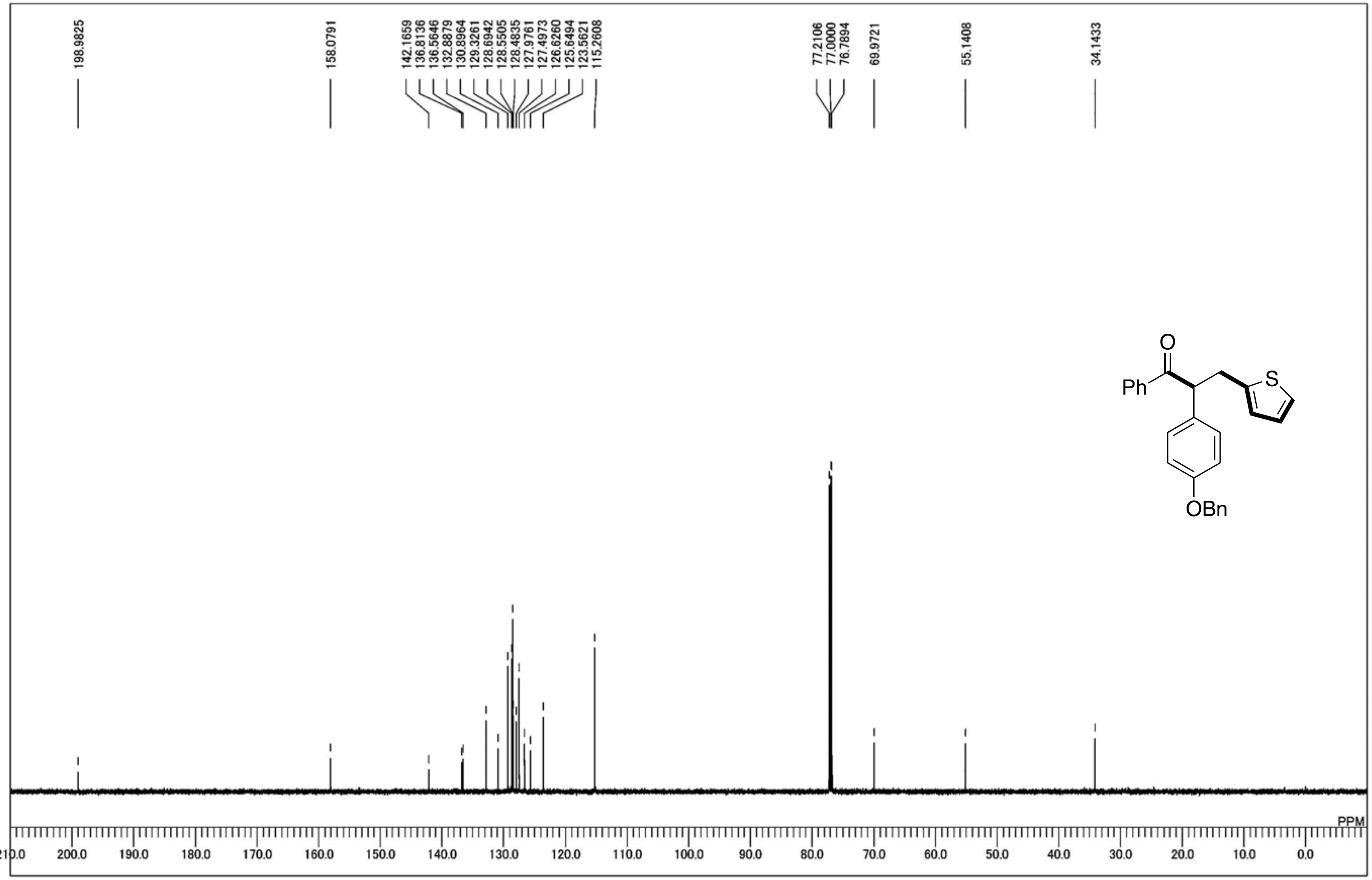

Supplementary Fig. 72 | ^{13}C NMR spectrum of **6acg**

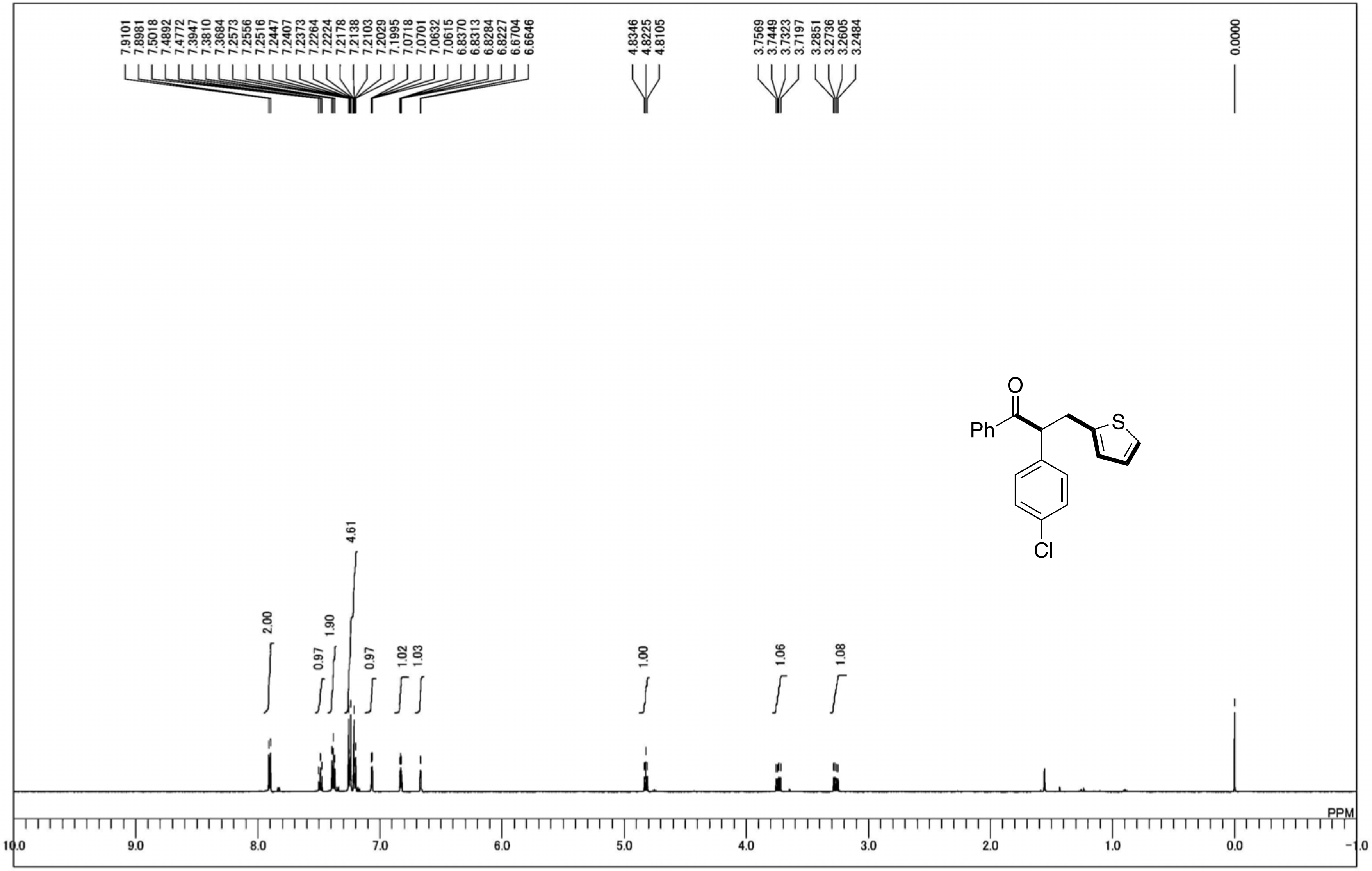

Supplementary Fig. 73 | ^{1}H NMR spectrum of **6adg**

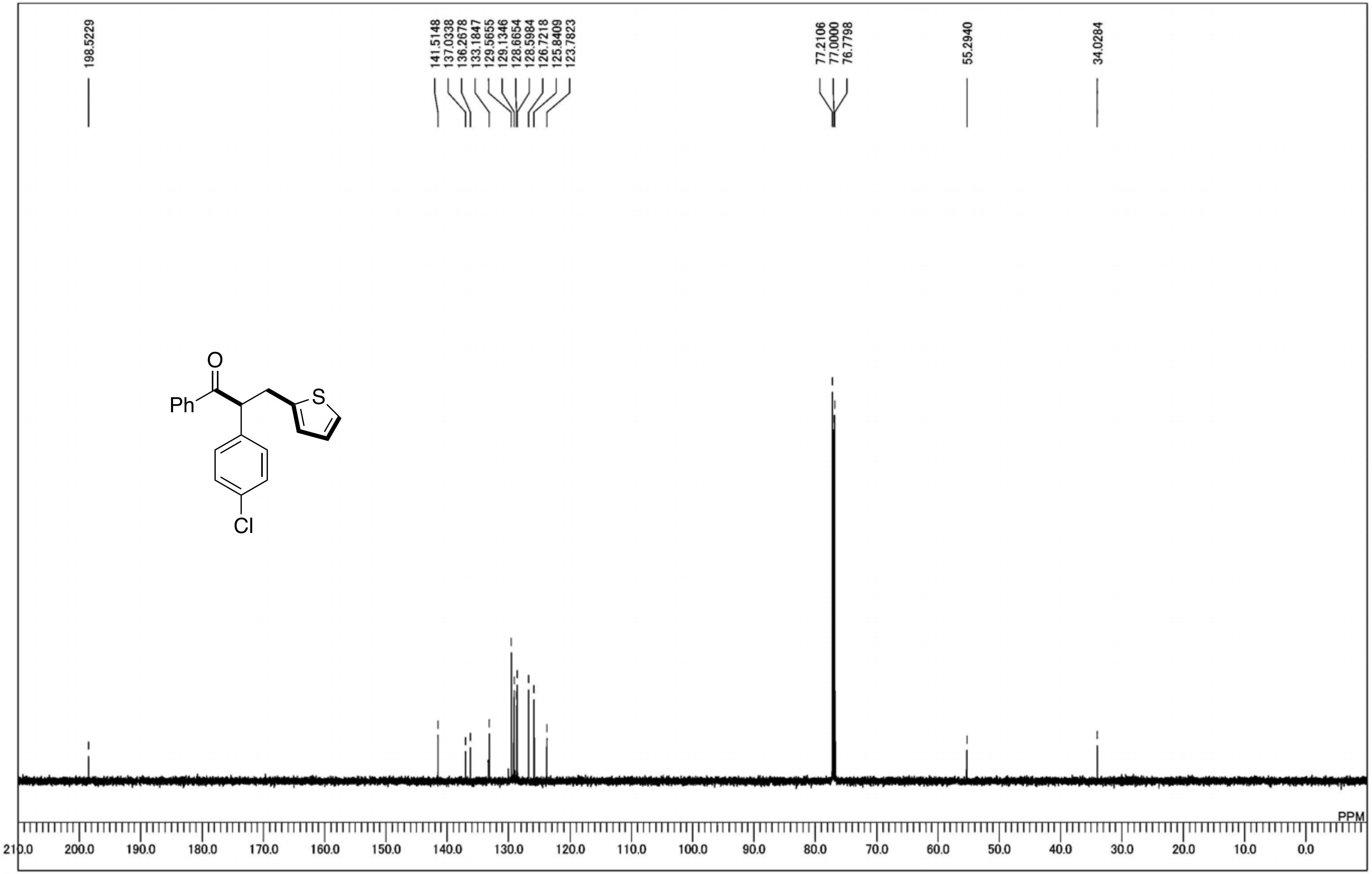

Supplementary Fig. 74 | ^{13}C NMR spectrum of **6adg**

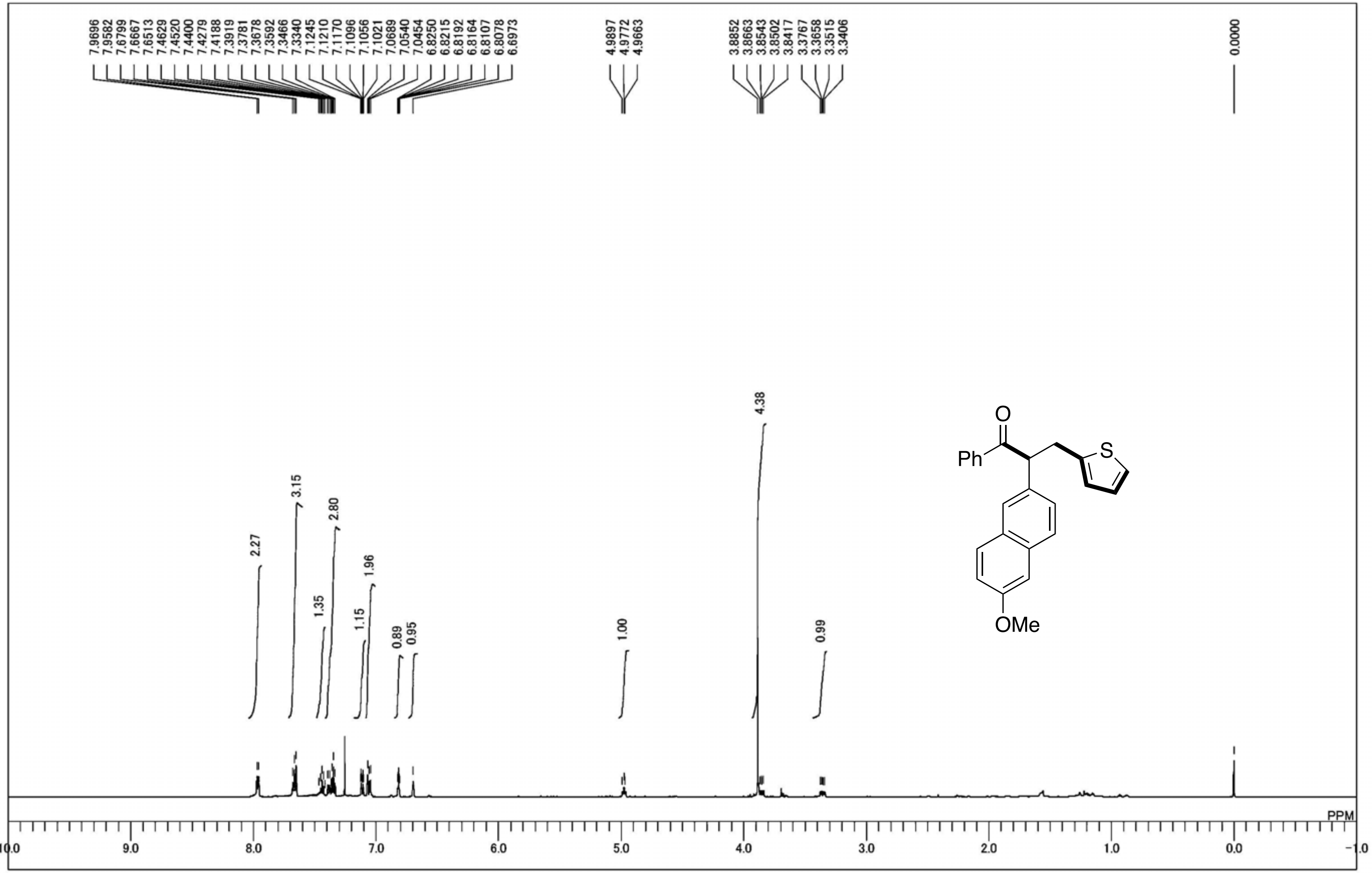

Supplementary Fig. 75 | ^{1}H NMR spectrum of **6aeg**

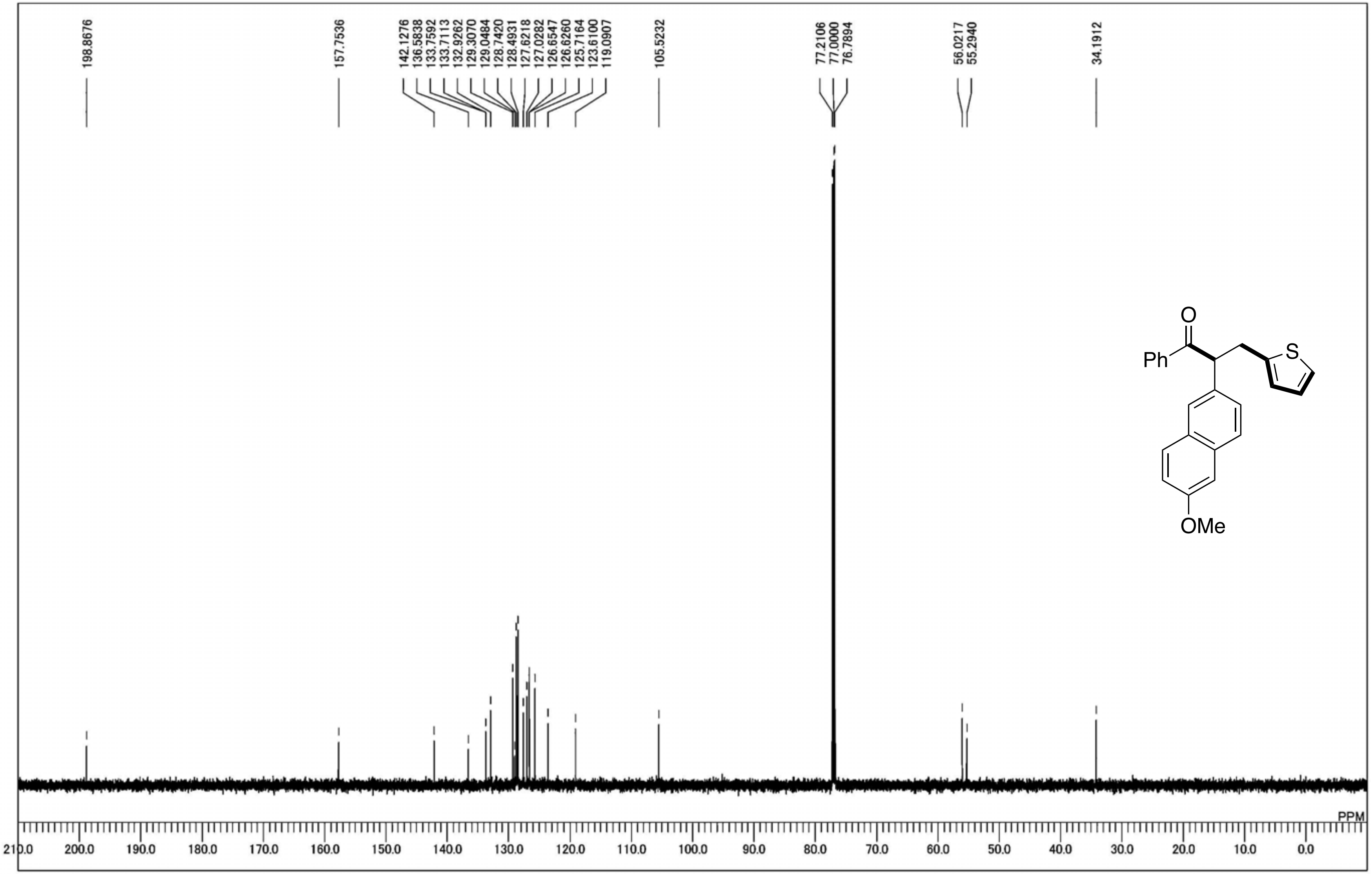

Supplementary Fig. 76 | ^{13}C NMR spectrum of **6aeg**

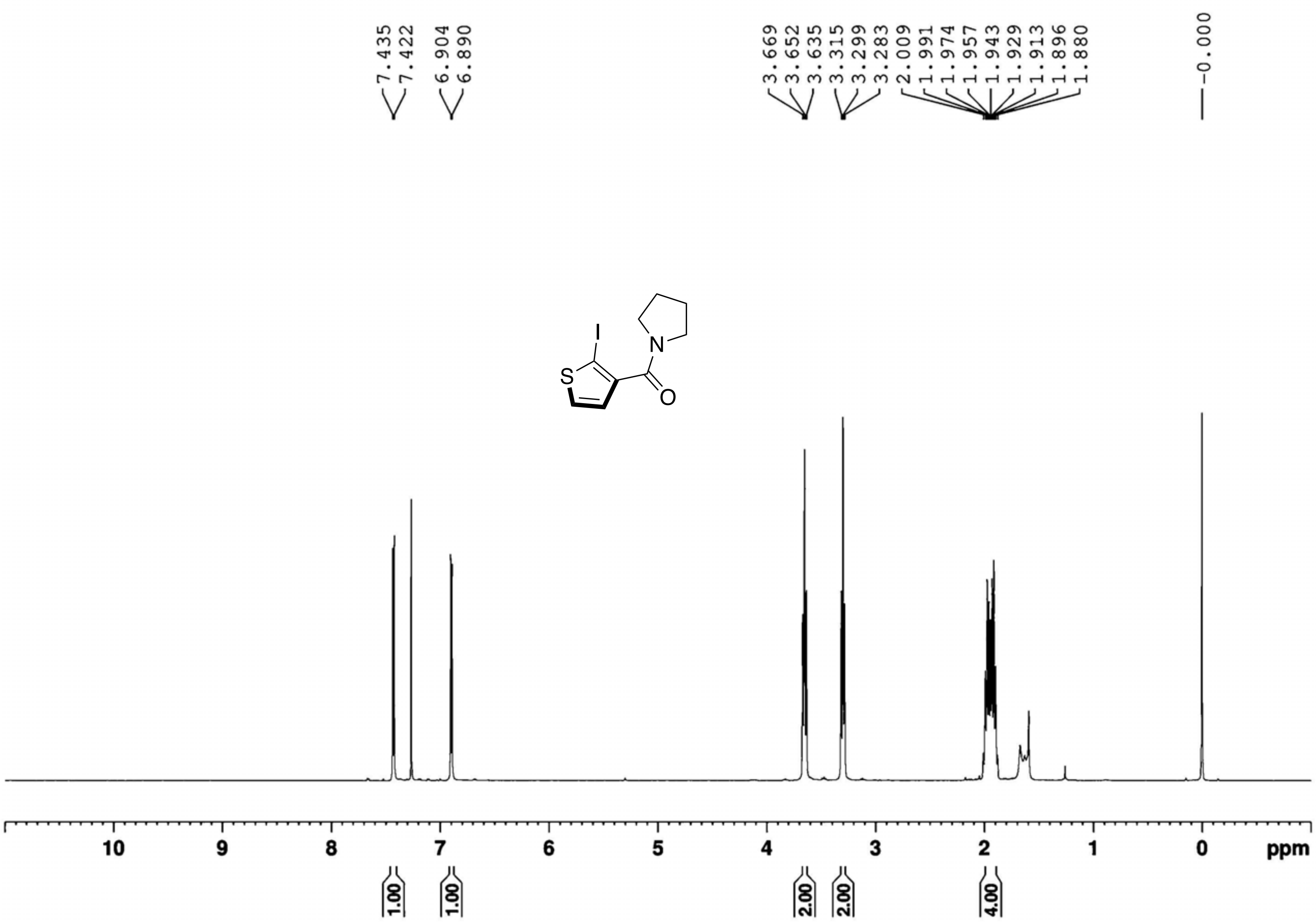

Supplementary Fig. 77 | ^{1}H NMR spectrum of **7a**

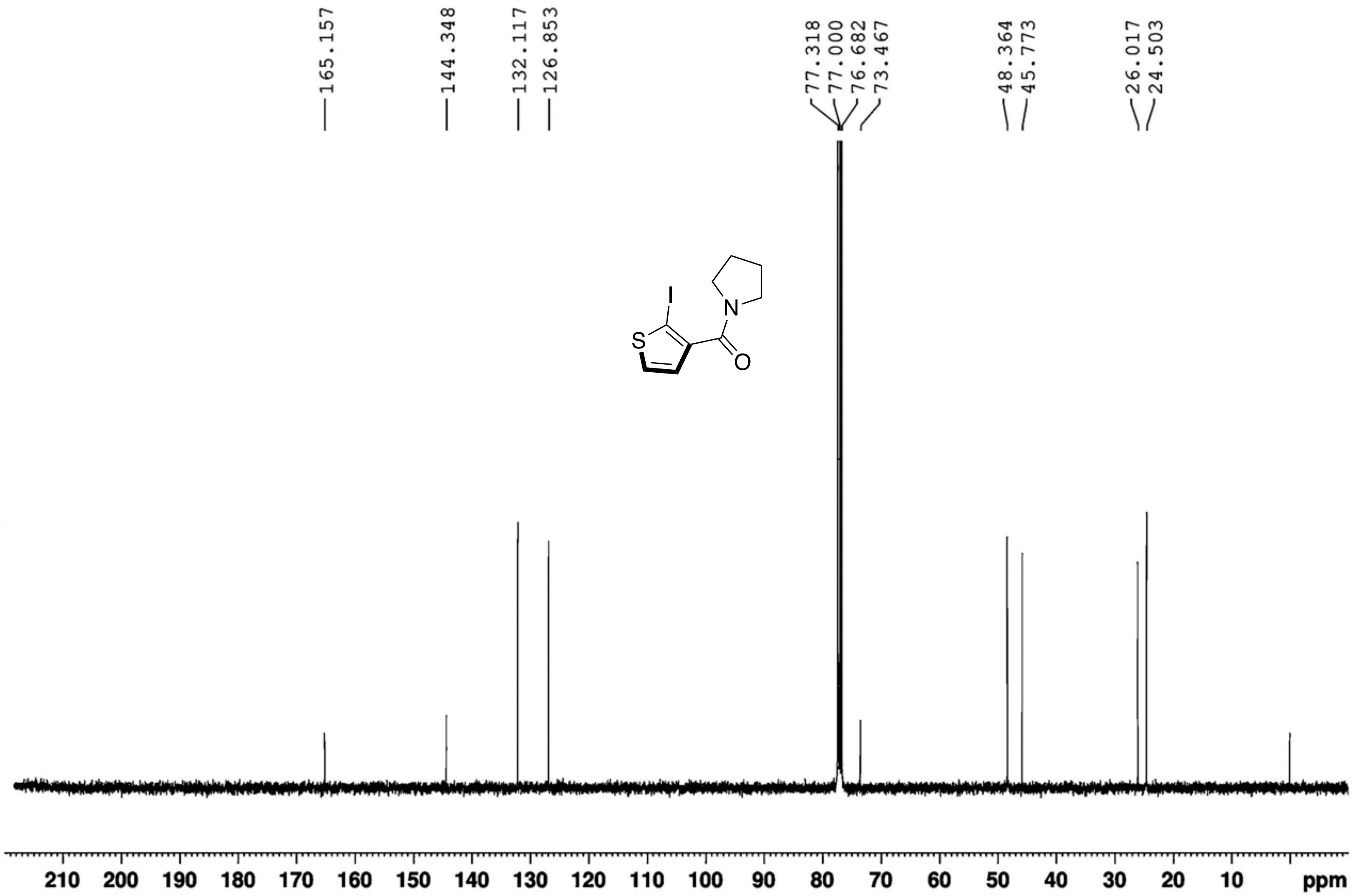

Supplementary Fig. 78 | ^{13}C NMR spectrum of **7a**

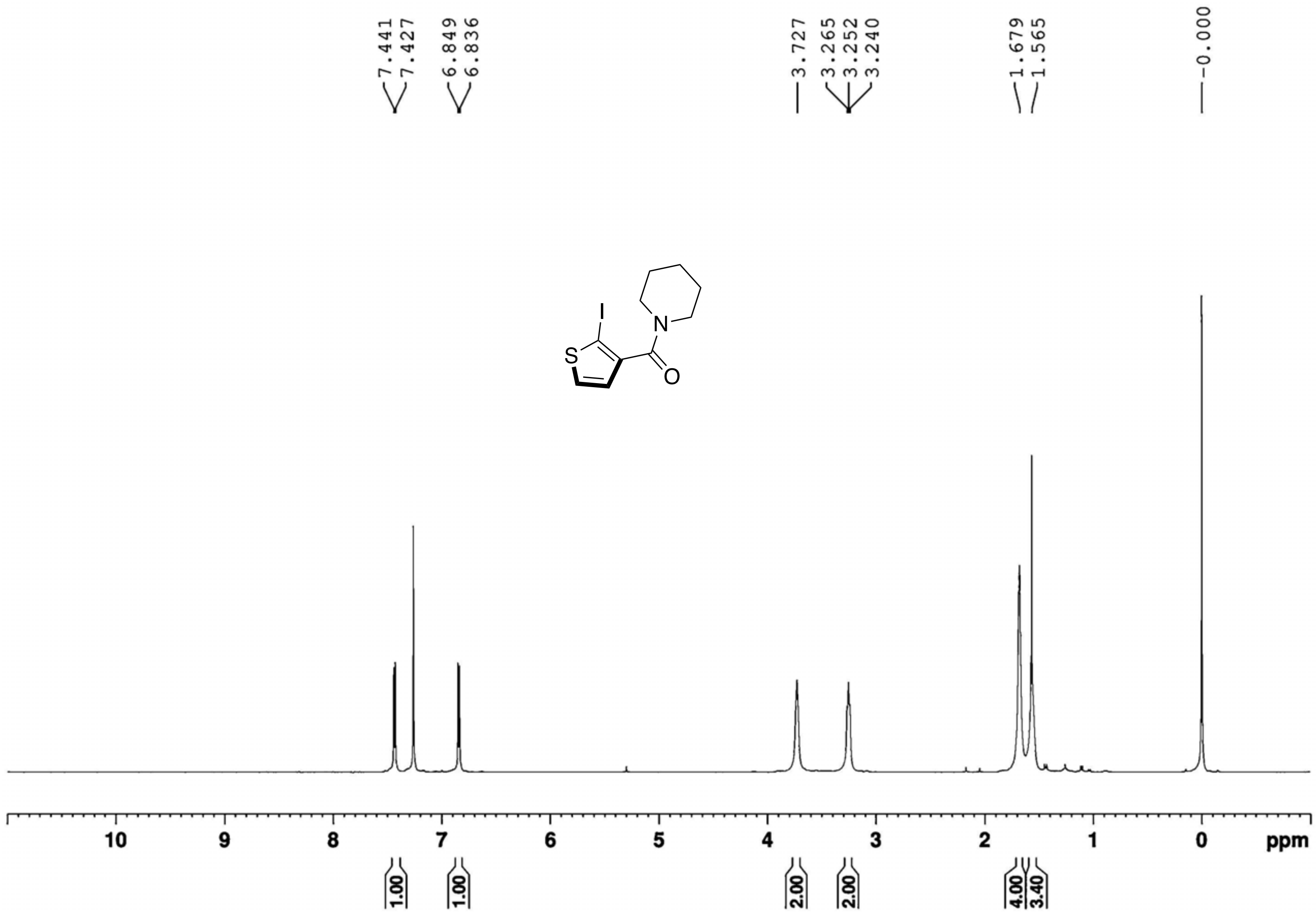

Supplementary Fig. 79 | ^{1}H NMR spectrum of **7b**

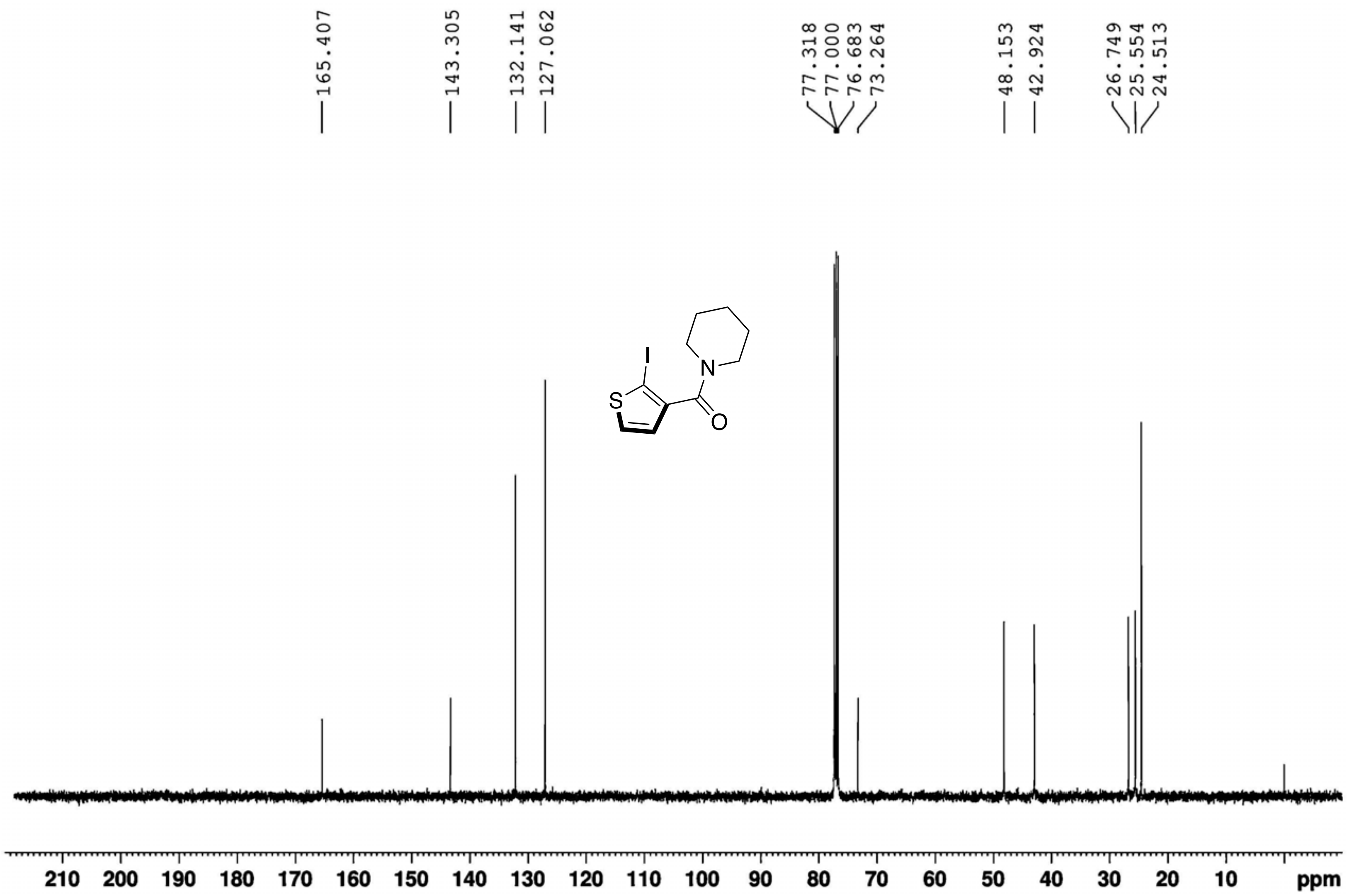

Supplementary Fig. 80 | ^{13}C NMR spectrum of **7b**

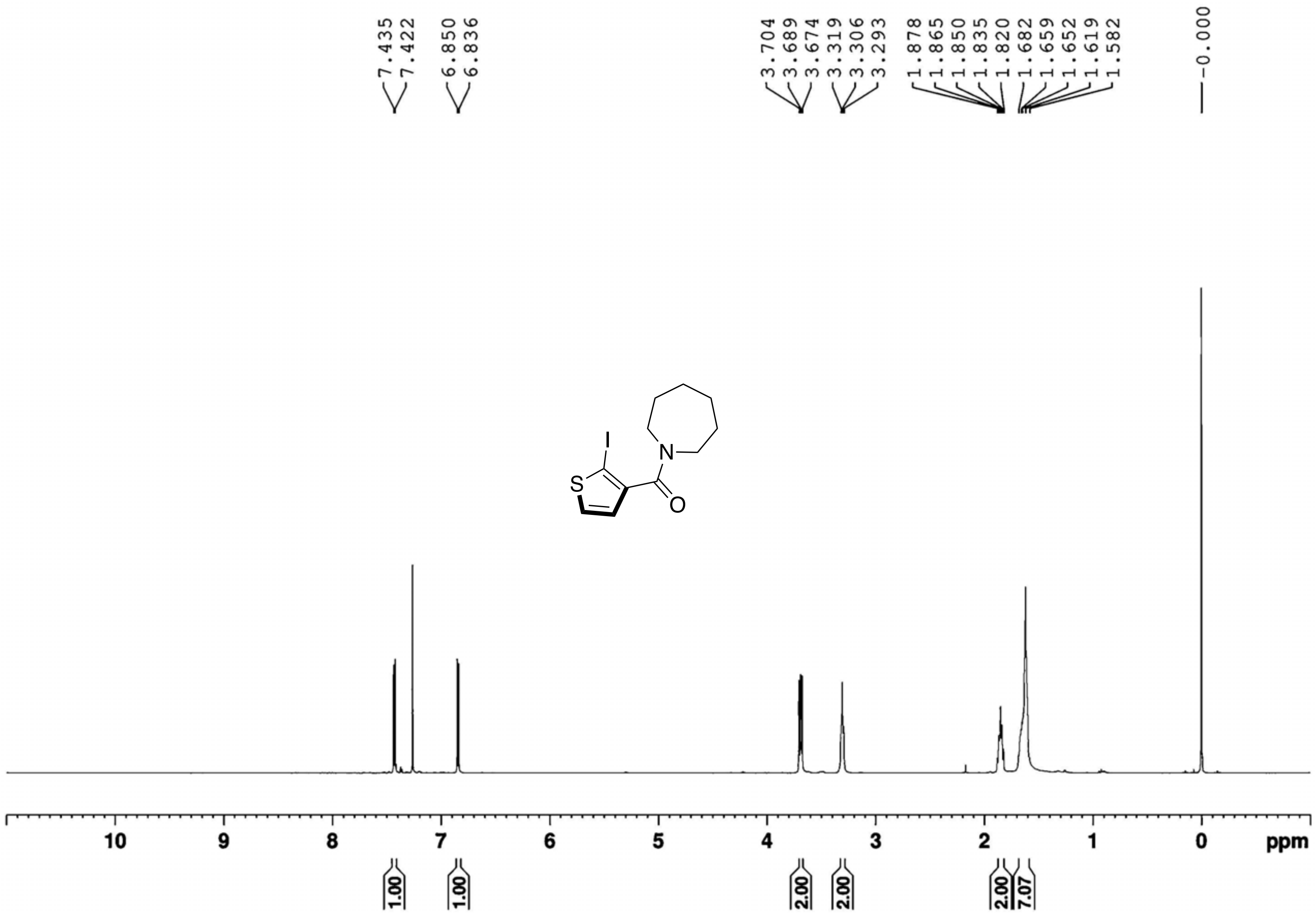

Supplementary Fig. 81 | ^{1}H NMR spectrum of **7c**

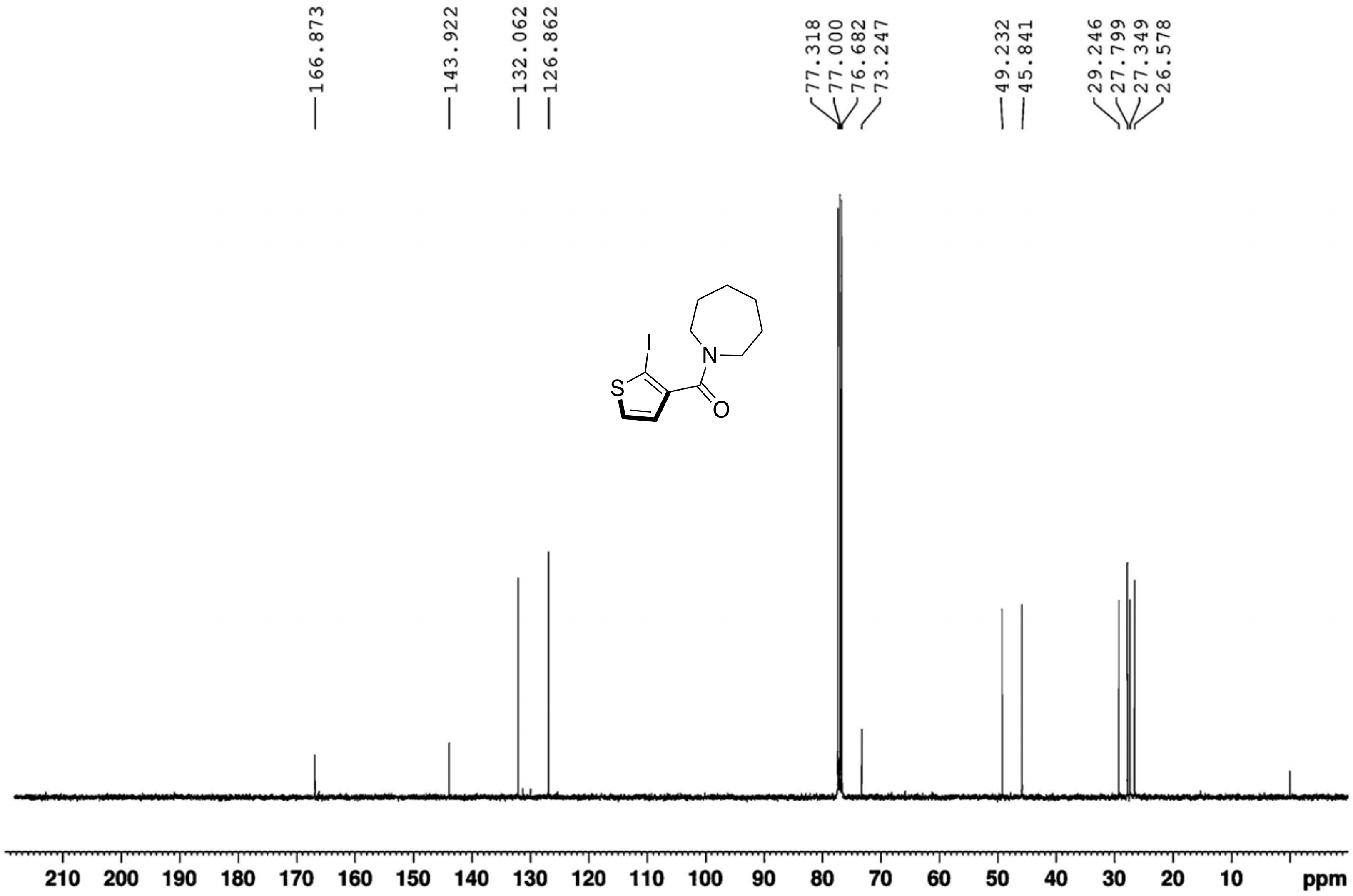

Supplementary Fig. 82 | ^{13}C NMR spectrum of **7c**

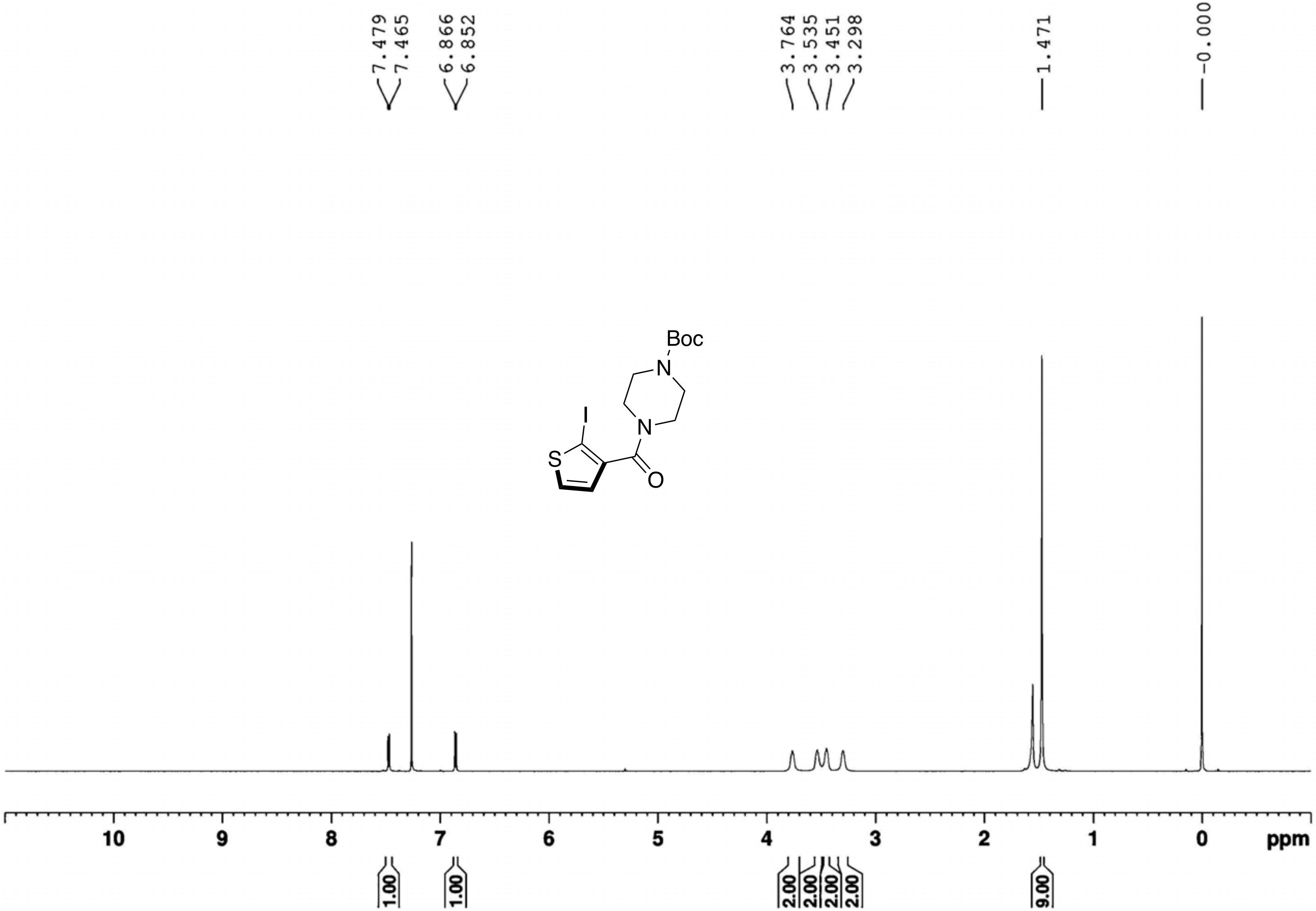

Supplementary Fig. 83 | ^{1}H NMR spectrum of **7d**

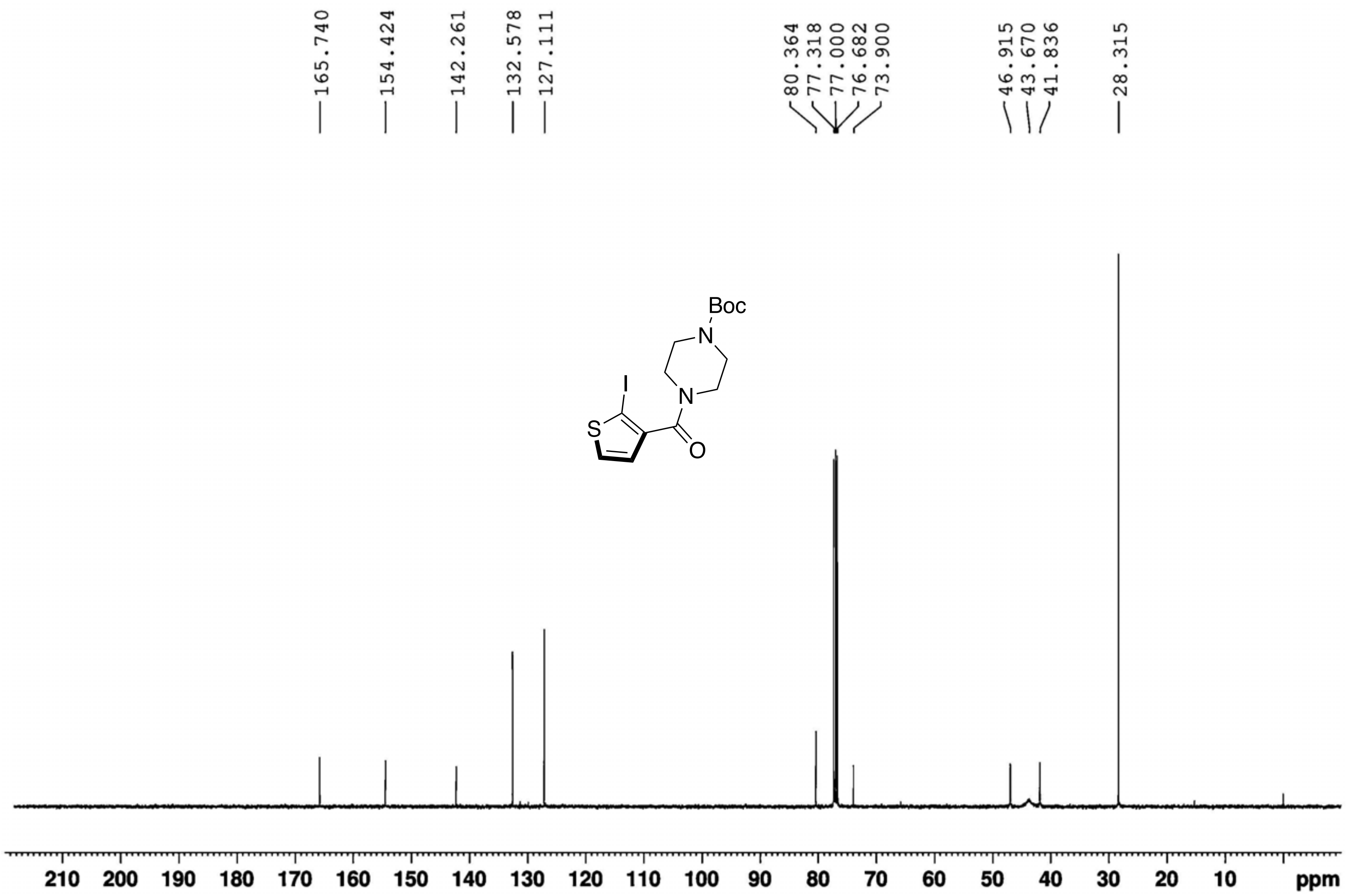

Supplementary Fig. 84 | ^{13}C NMR spectrum of **7d**

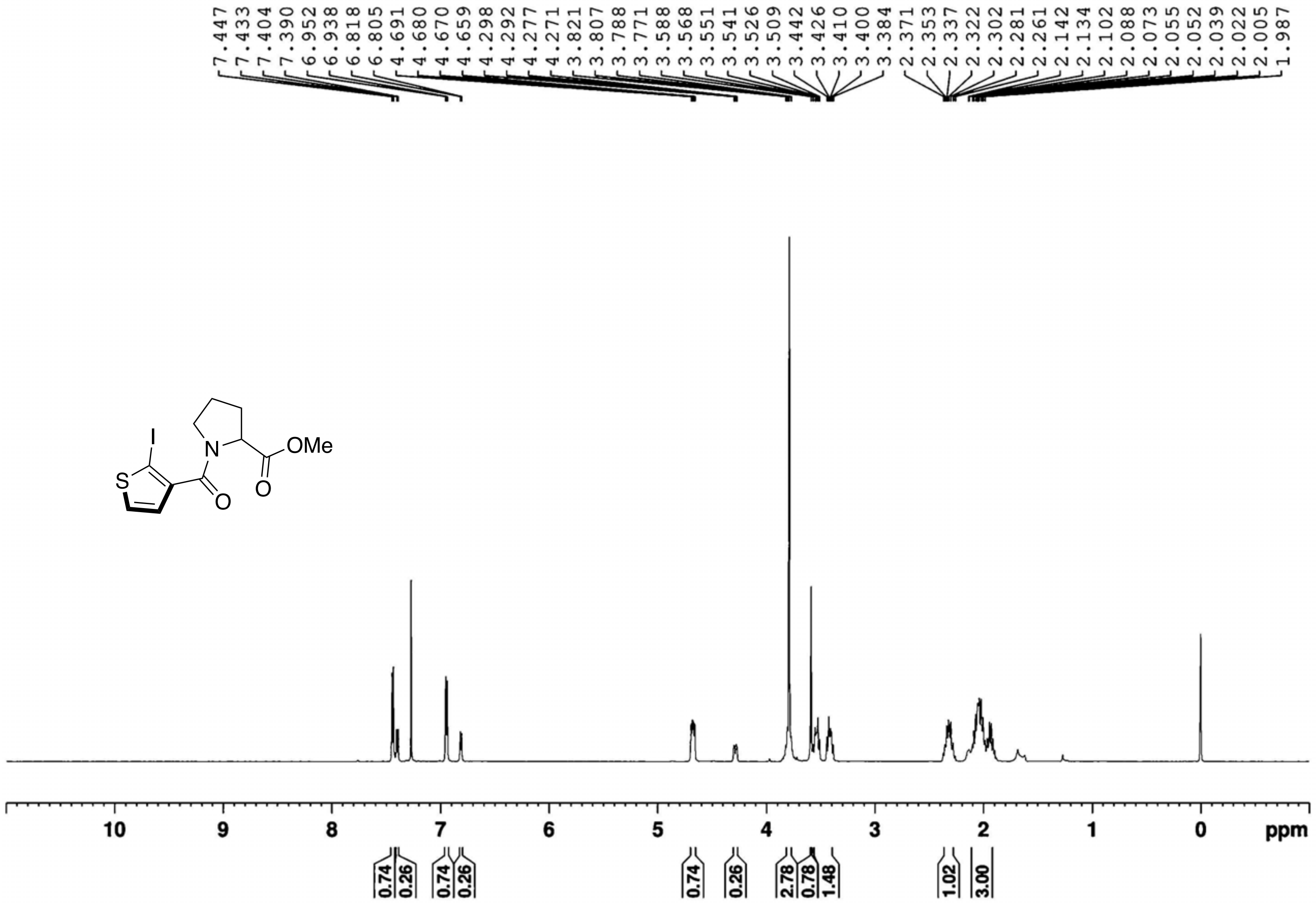

Supplementary Fig. 85 | ^{1}H NMR spectrum of **7e**

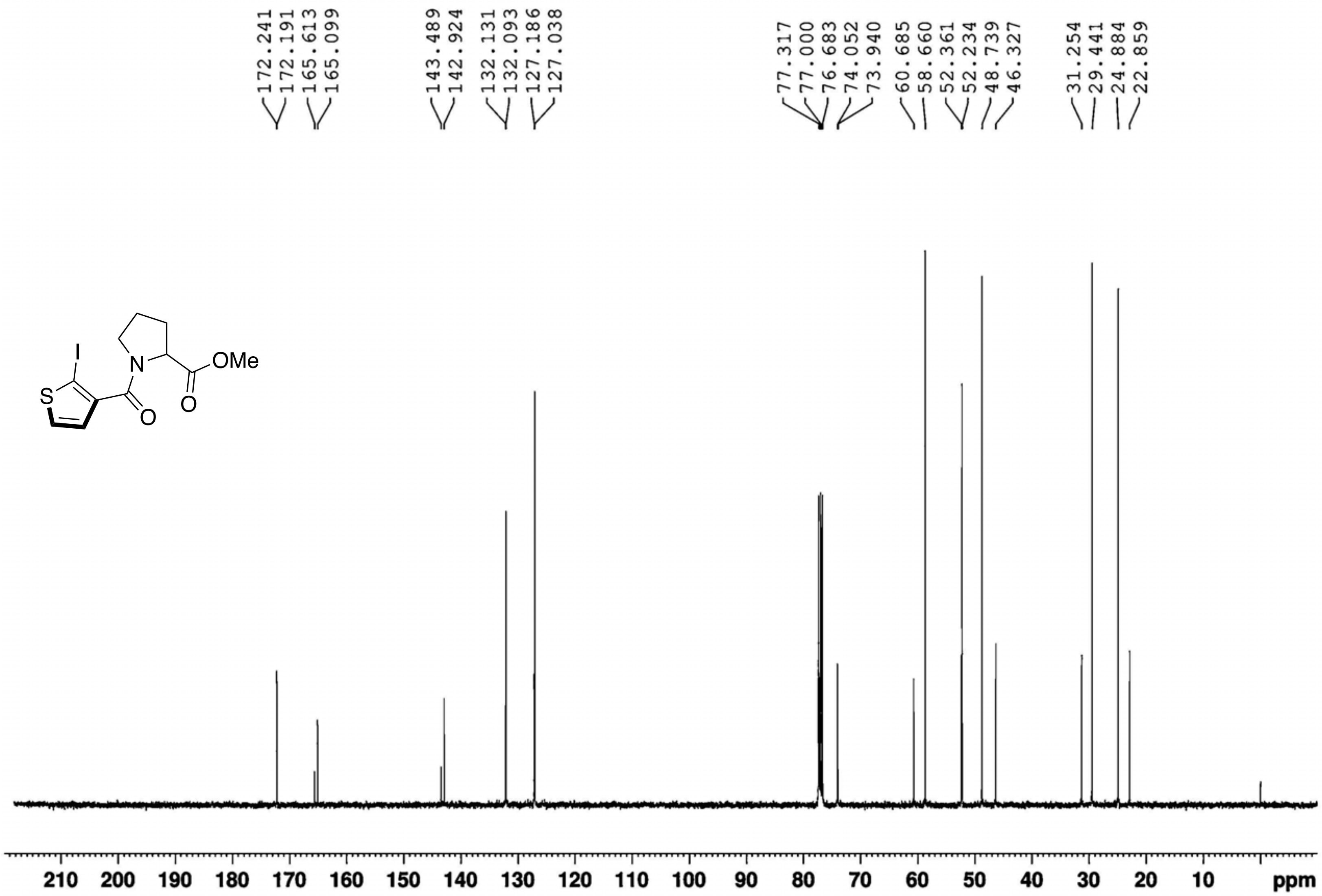

Supplementary Fig. 86 | ^{13}C NMR spectrum of **7e**

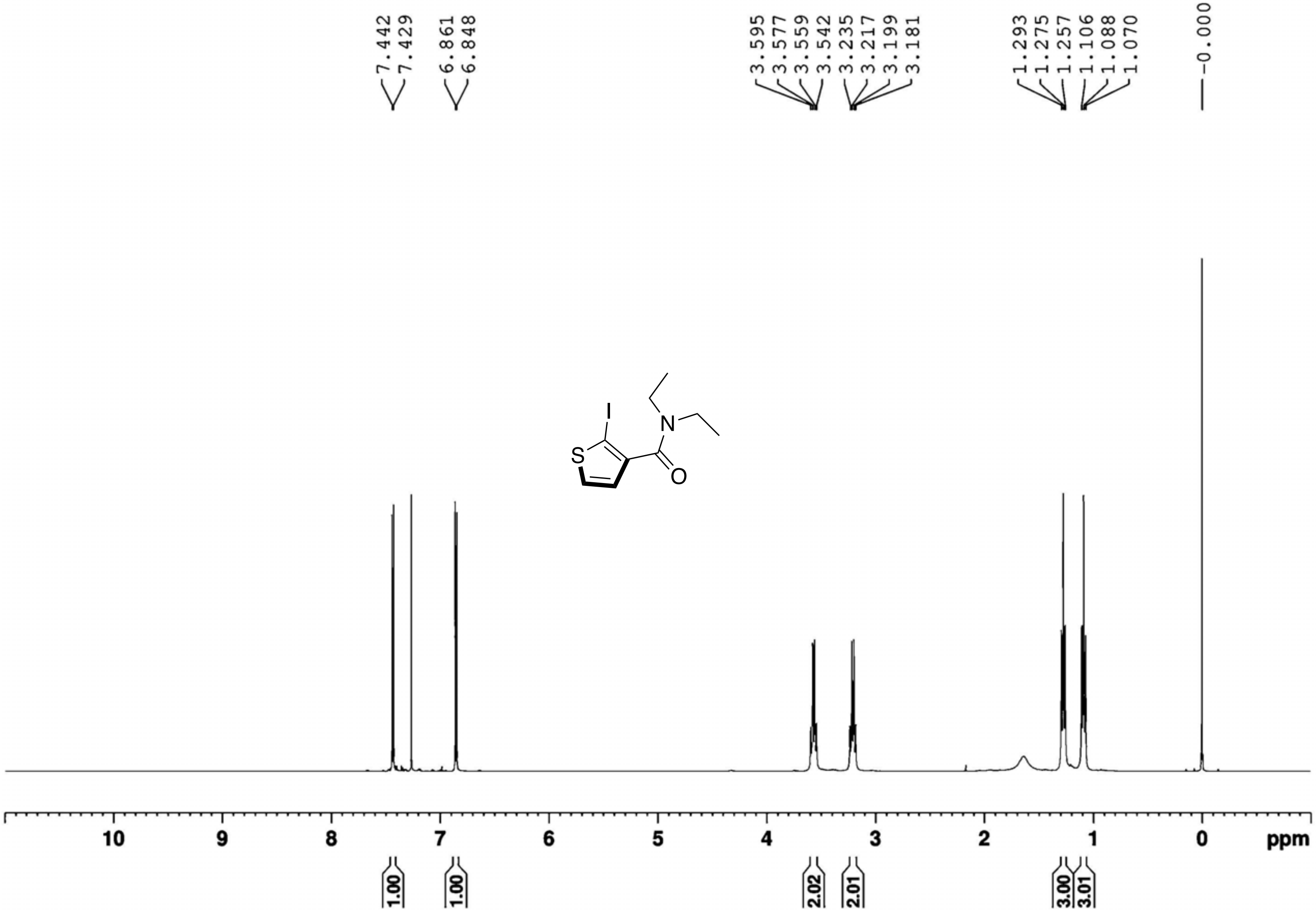

Supplementary Fig. 87 | ^{1}H NMR spectrum of **7f**

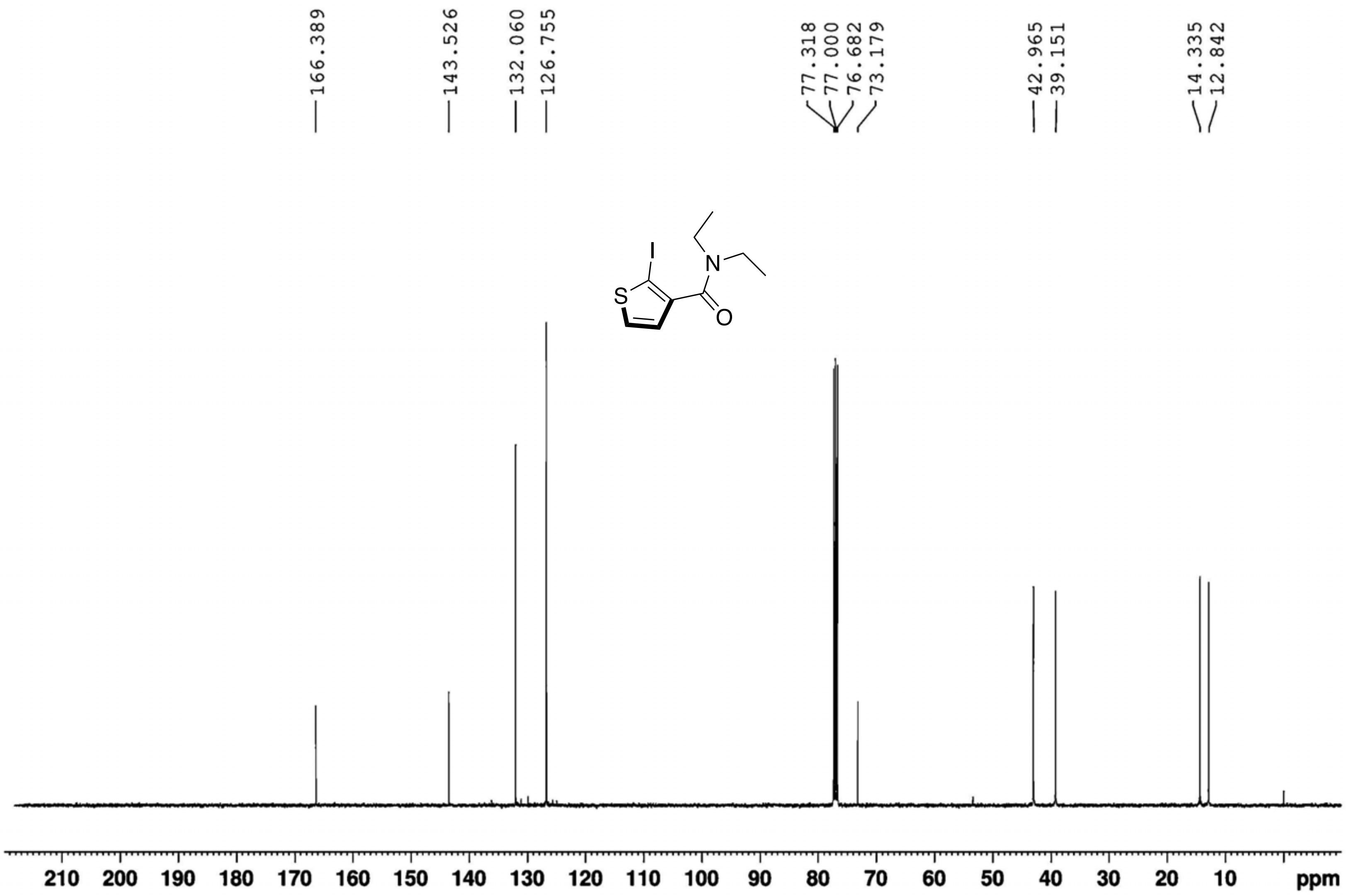

Supplementary Fig. 88 | ^{13}C NMR spectrum of **7f**

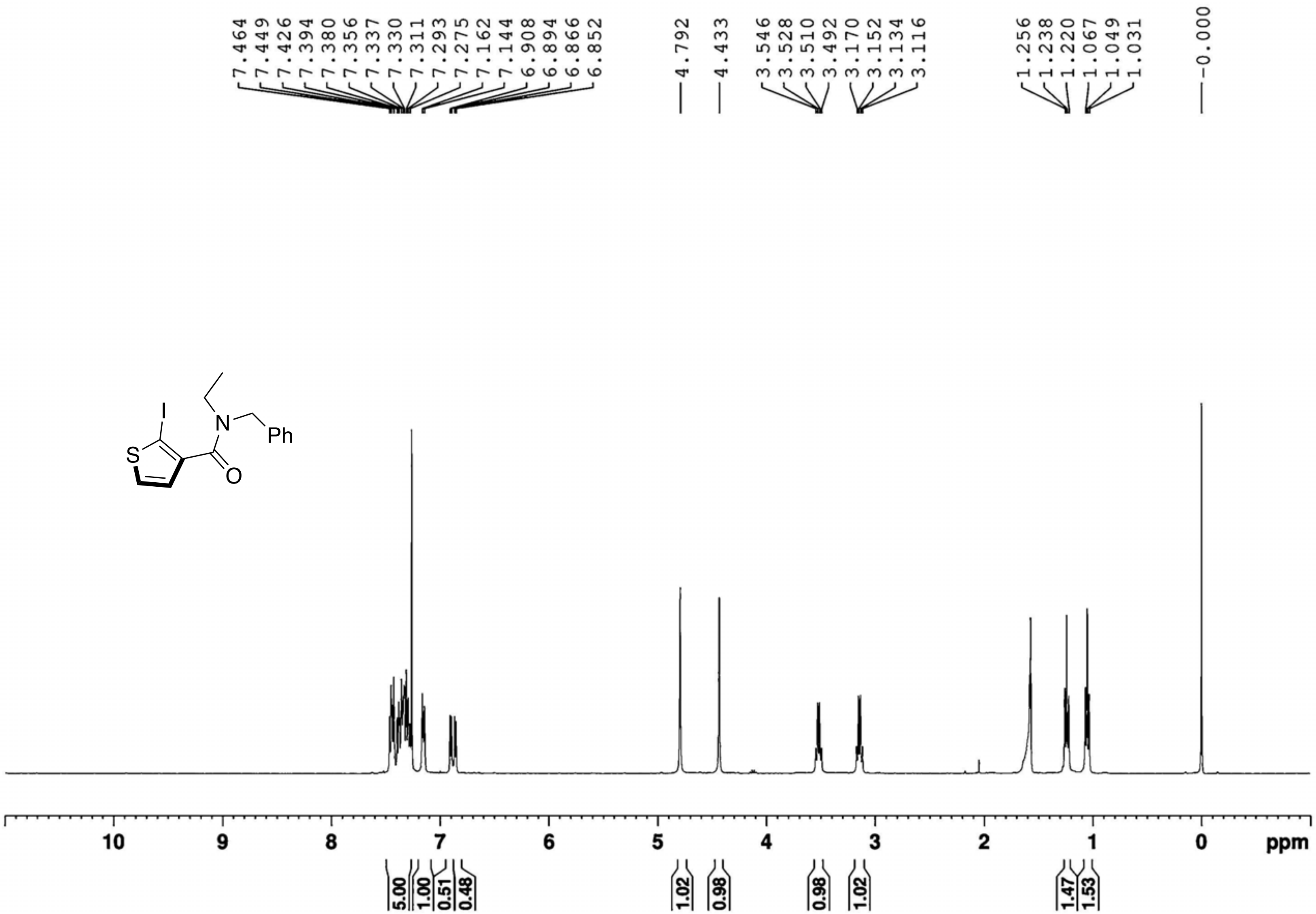

Supplementary Fig. 89 | ^{1}H NMR spectrum of **7g**

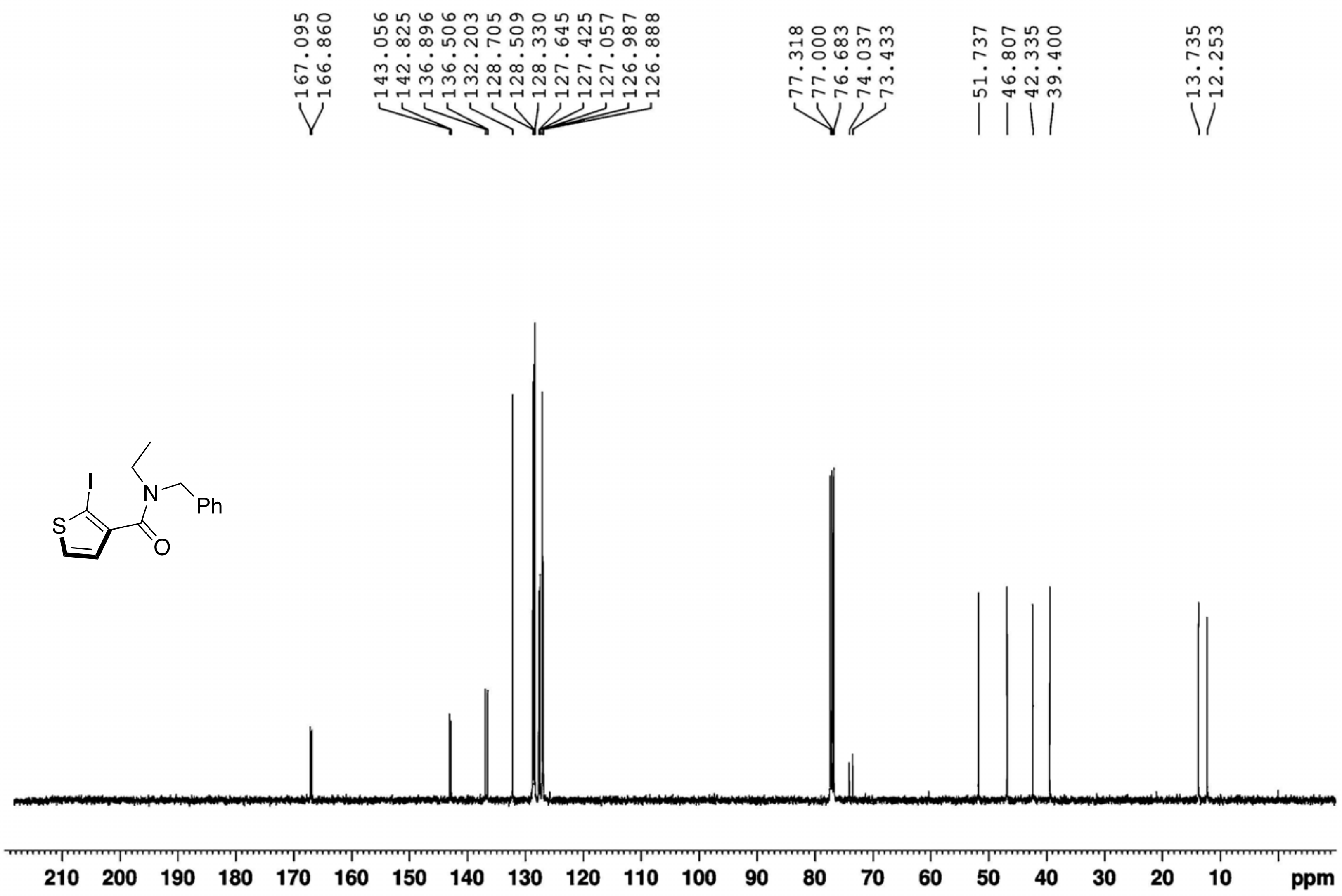

Supplementary Fig. 90 | ^{13}C NMR spectrum of **7g**

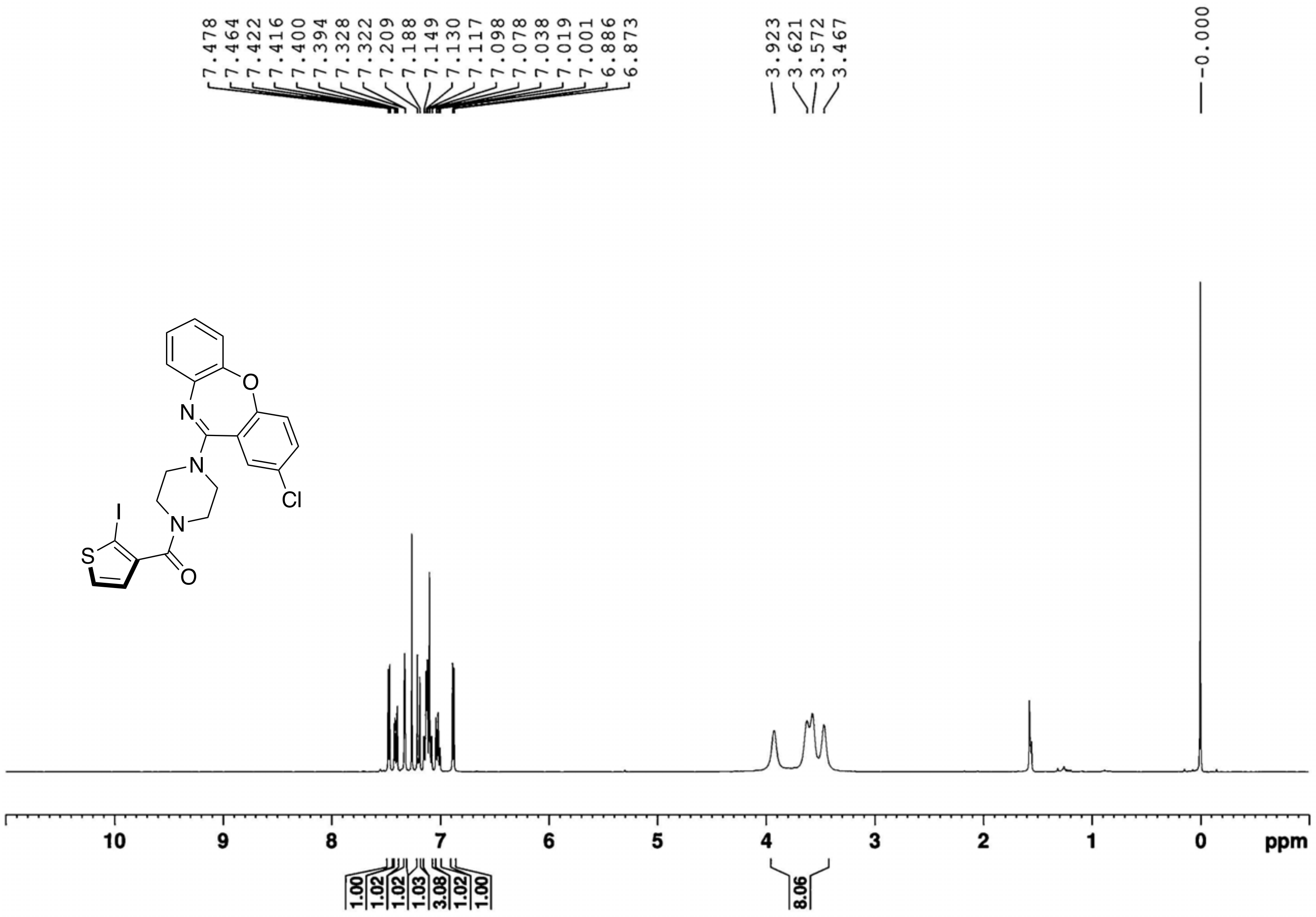

Supplementary Fig. 91 | ^{1}H NMR spectrum of **7h**

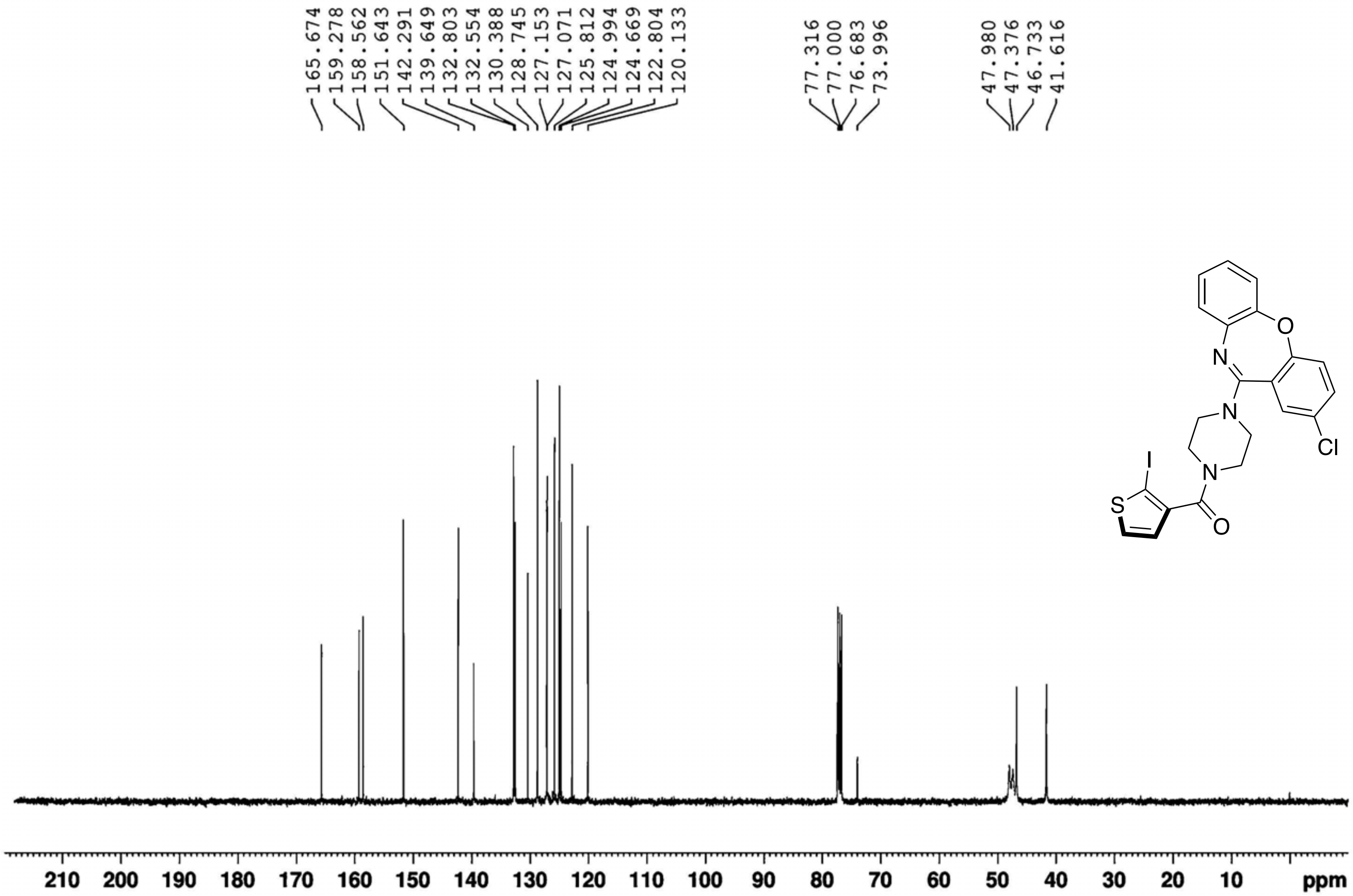

Supplementary Fig. 92 | ^{13}C NMR spectrum of **7h**

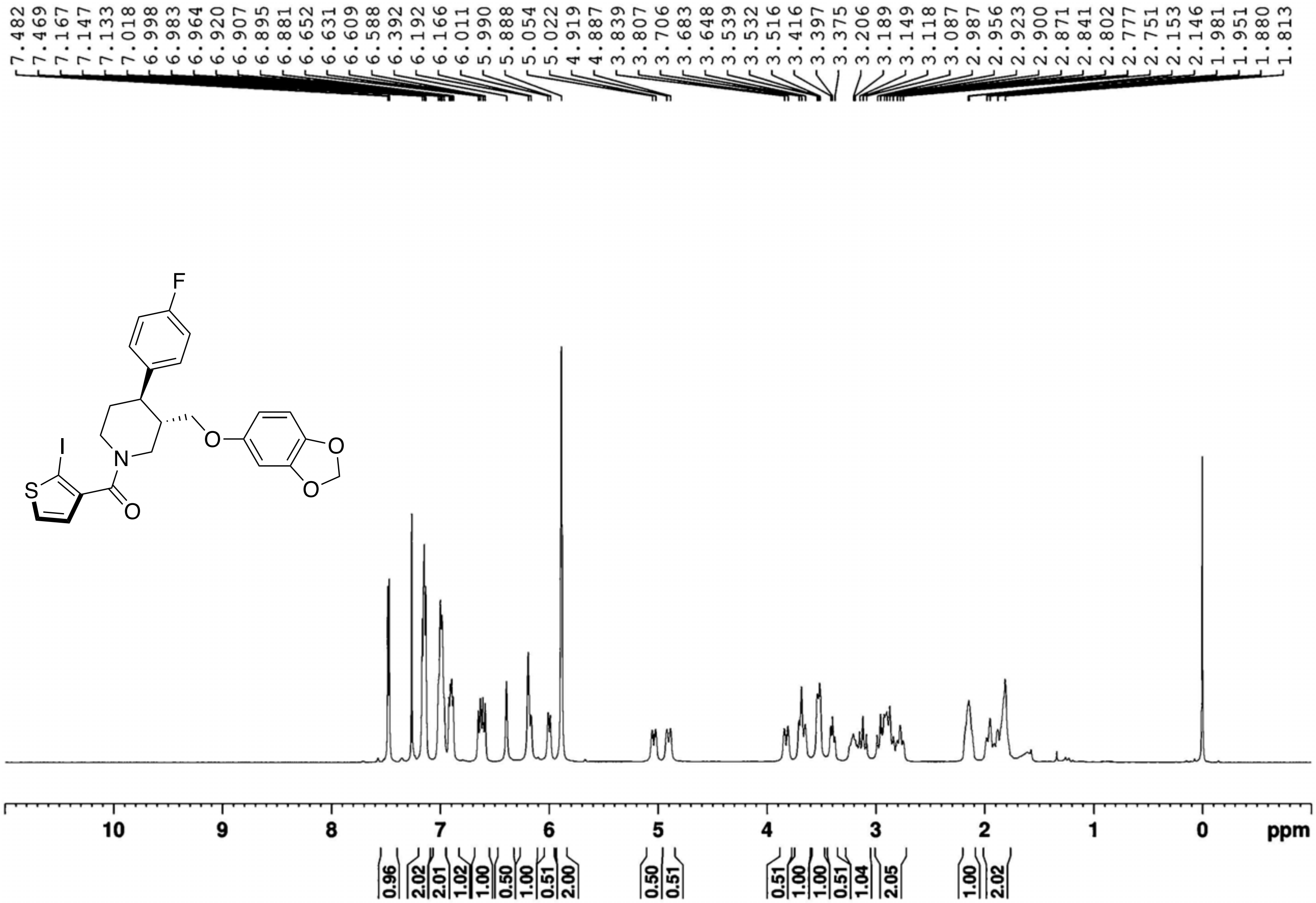

Supplementary Fig. 93 | ^{1}H NMR spectrum of **7i**

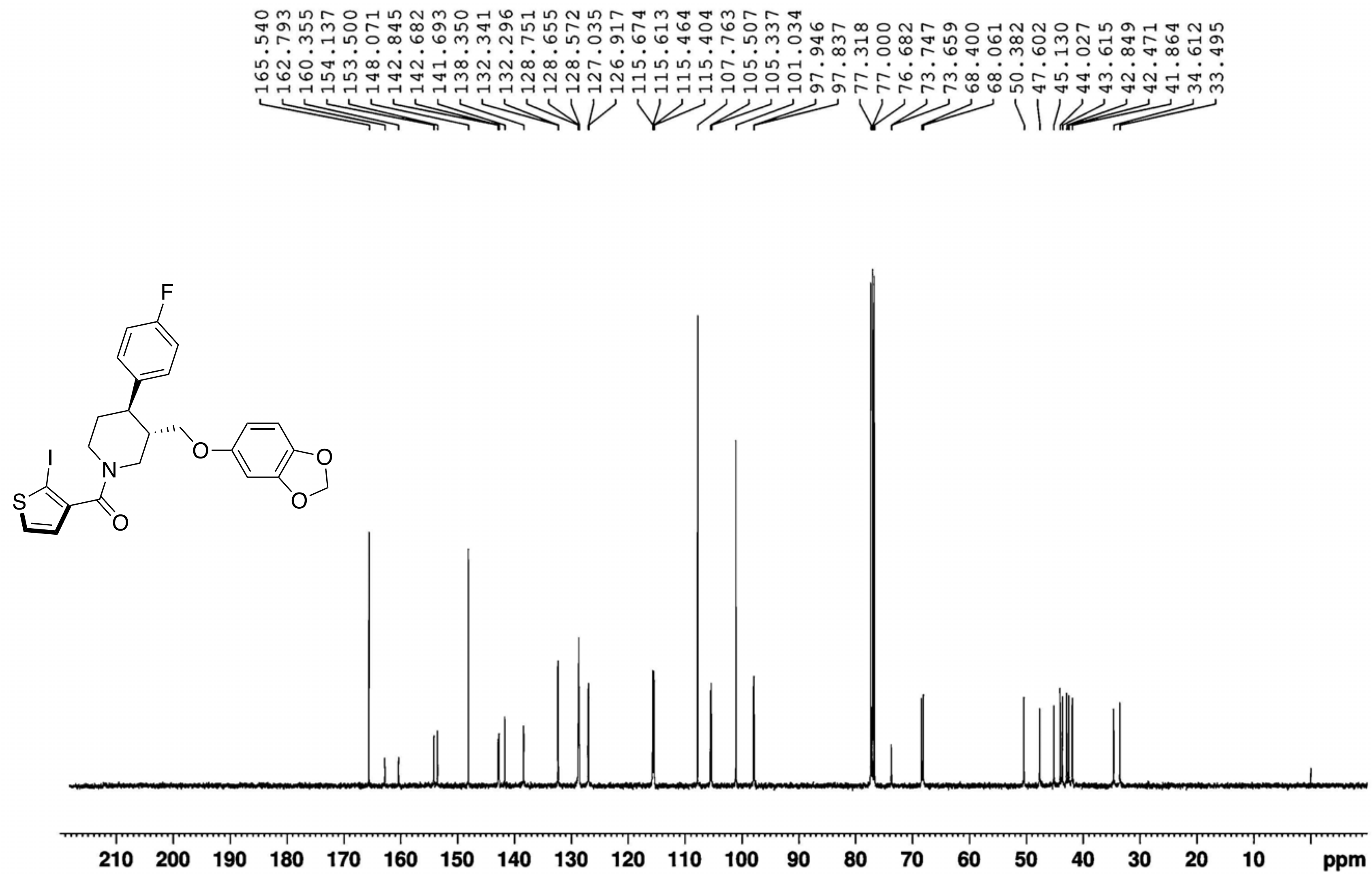

Supplementary Fig. 94 | ^{13}C NMR spectrum of **7i**

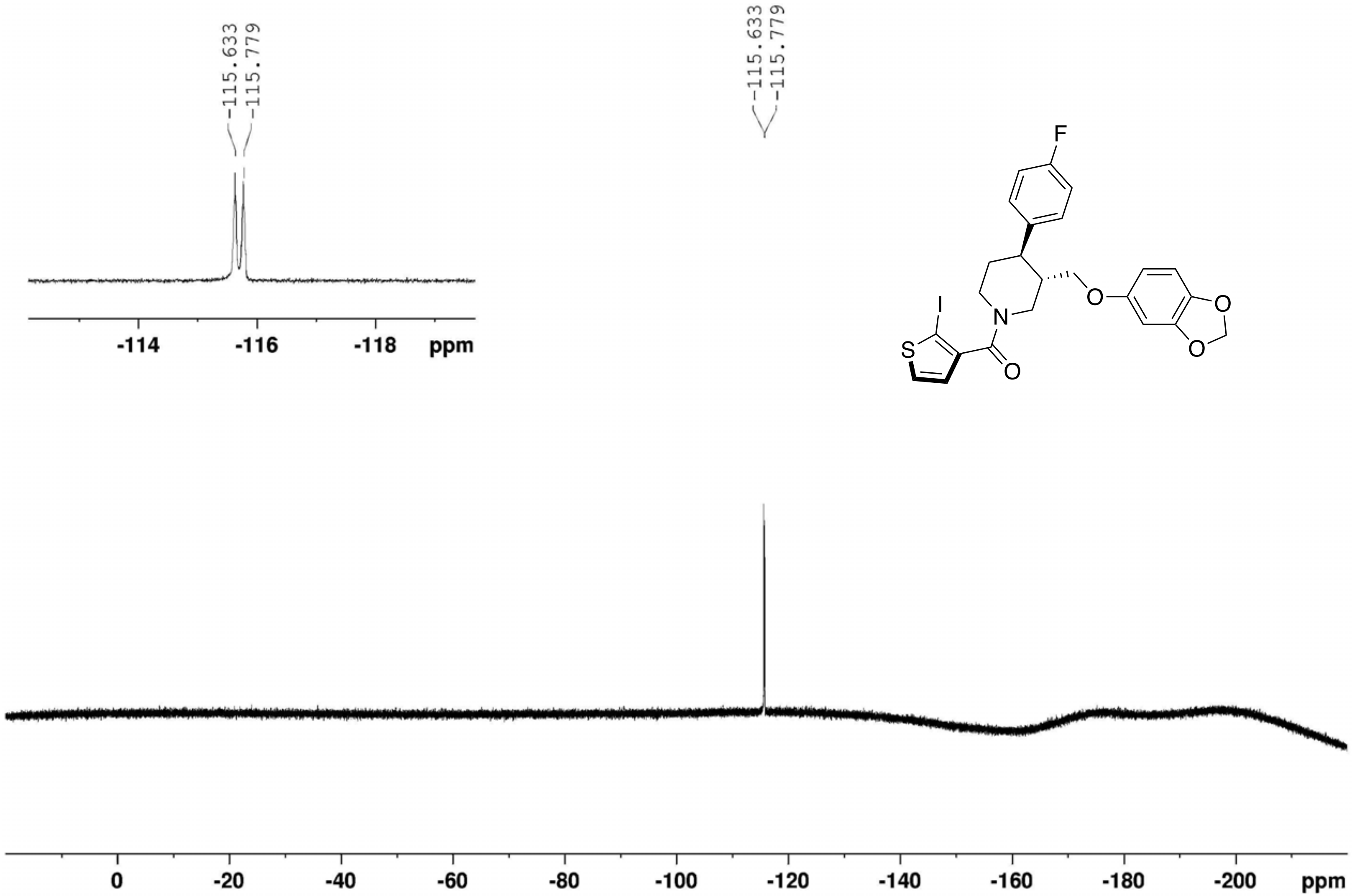

Supplementary Fig. 95 | ^{19}F NMR spectrum of **7i**

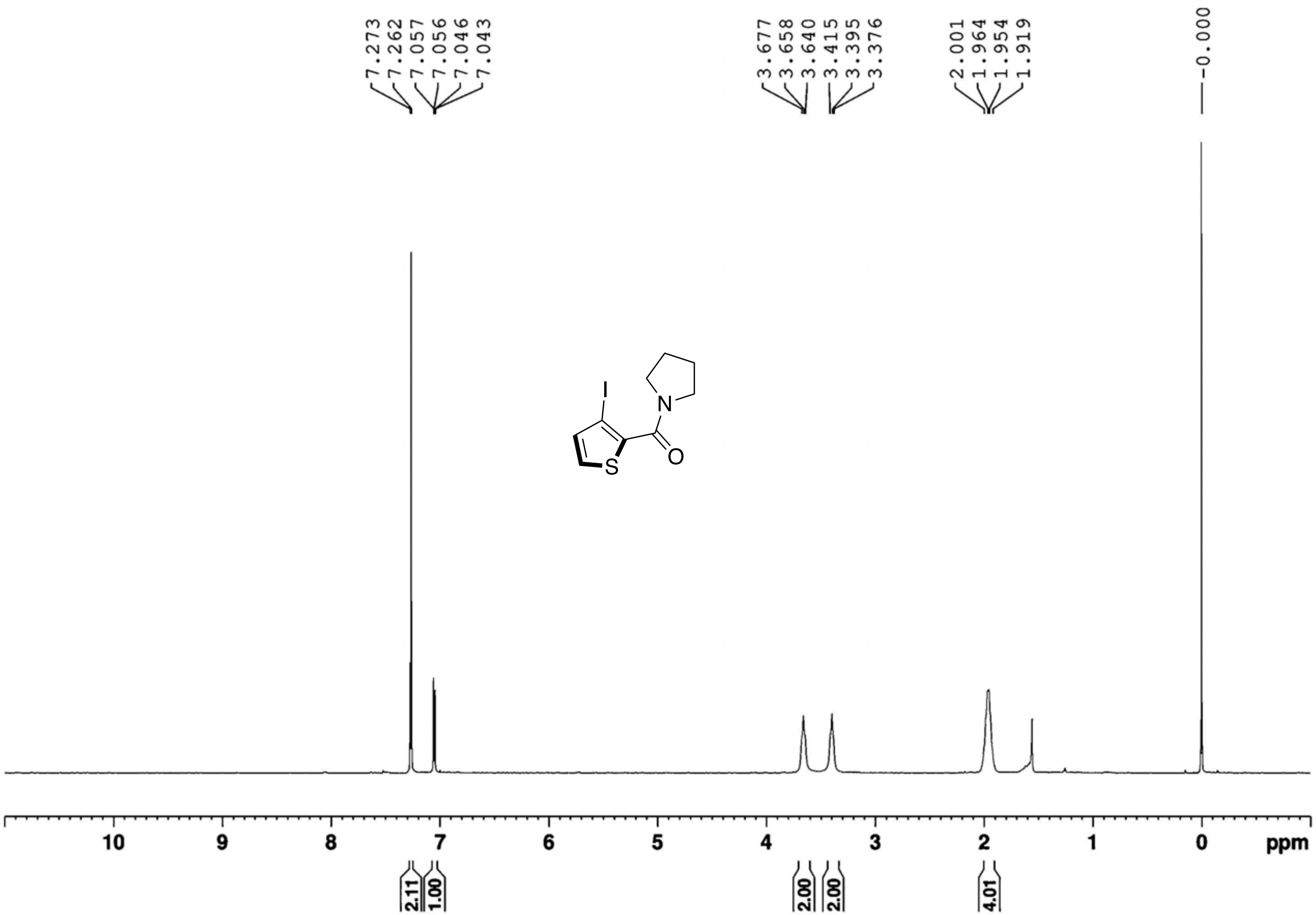

Supplementary Fig. 96 | ^{1}H NMR spectrum of **7a-A**

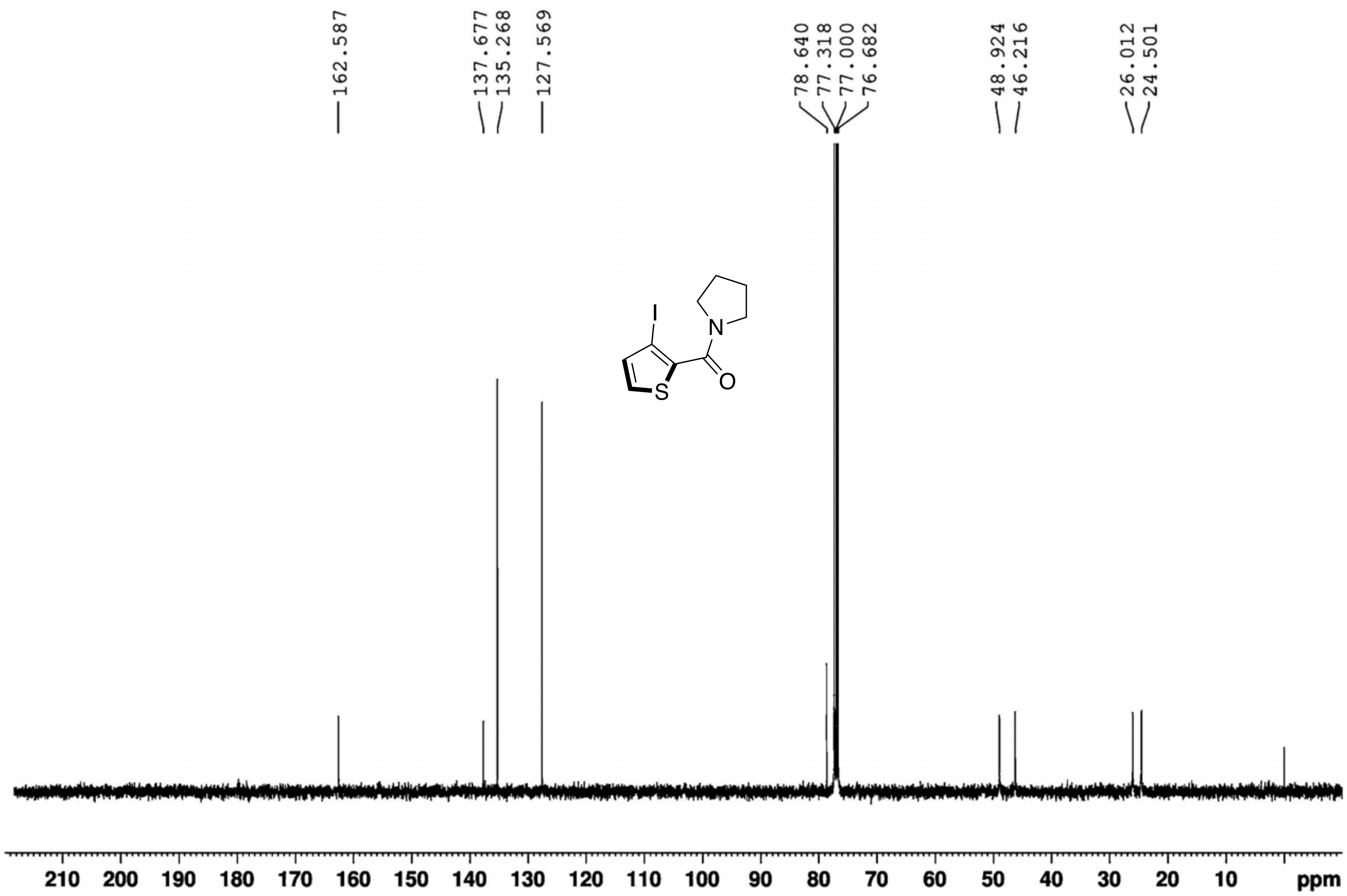

Supplementary Fig. 97 | ^{13}C NMR spectrum of **7a-A**

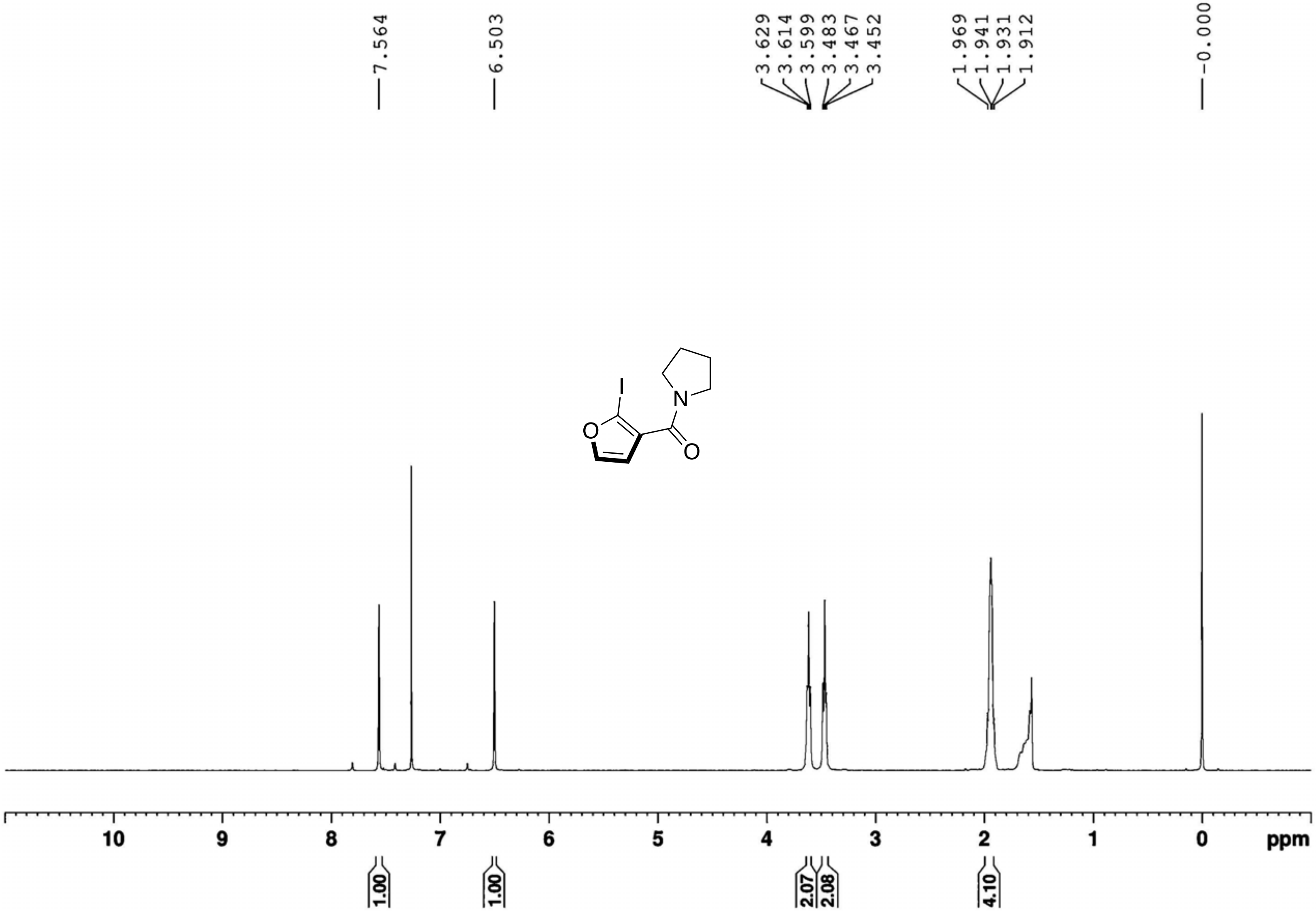

Supplementary Fig. 98 | ^{1}H NMR spectrum of **7a-B**

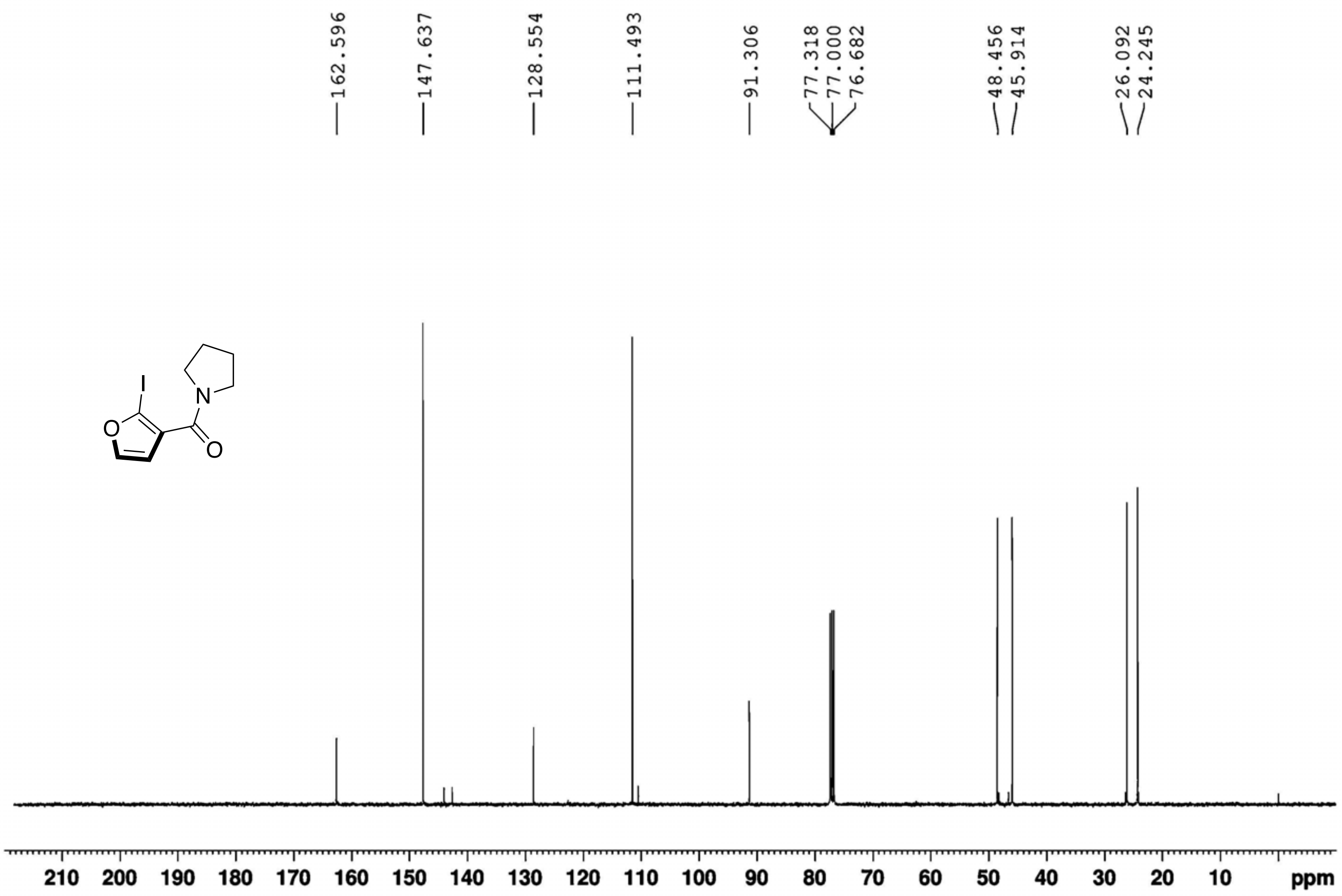

Supplementary Fig. 99 | ^{13}C NMR spectrum of **7a-B**

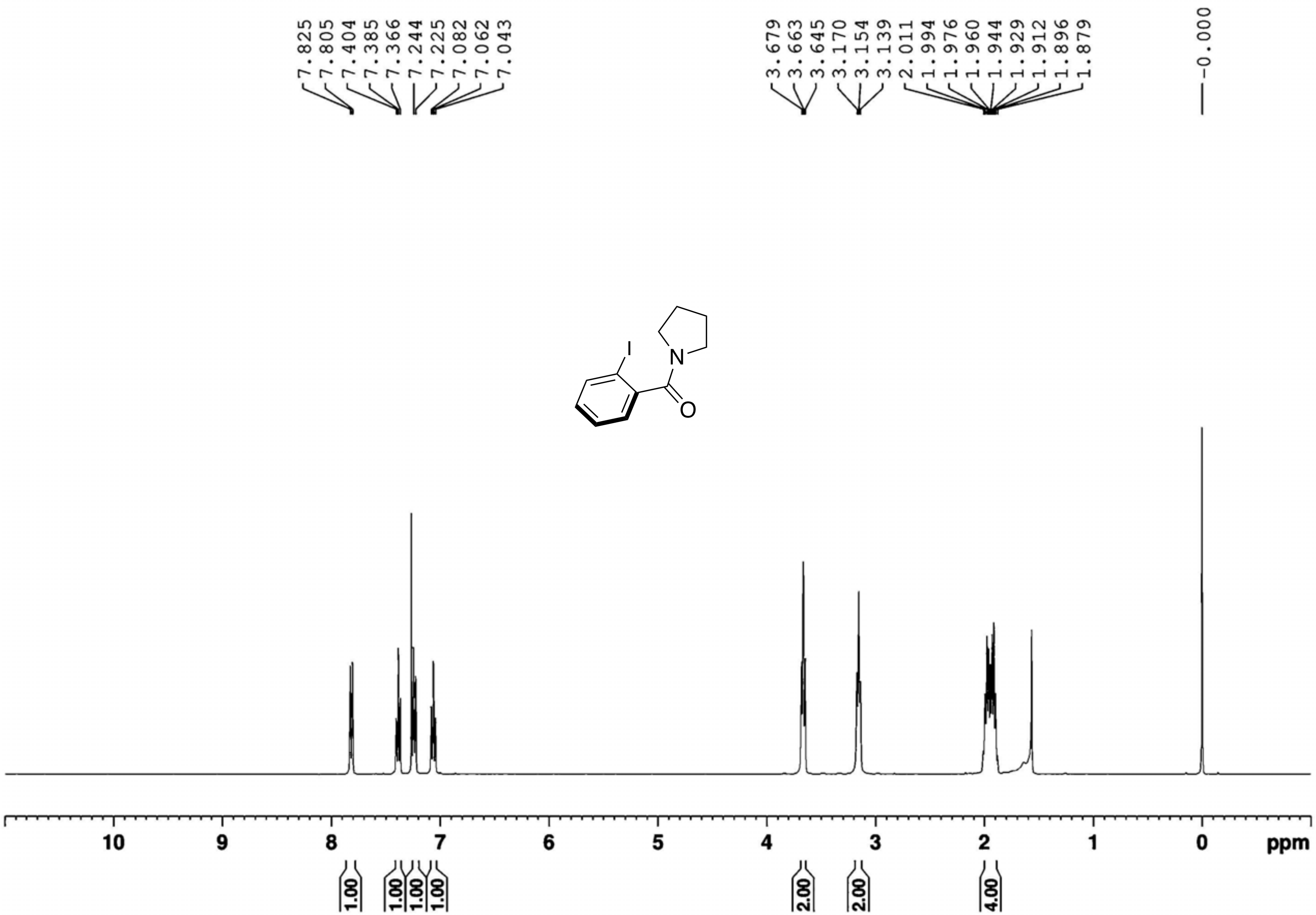

Supplementary Fig. 100 | ^{1}H NMR spectrum of **7a-C**

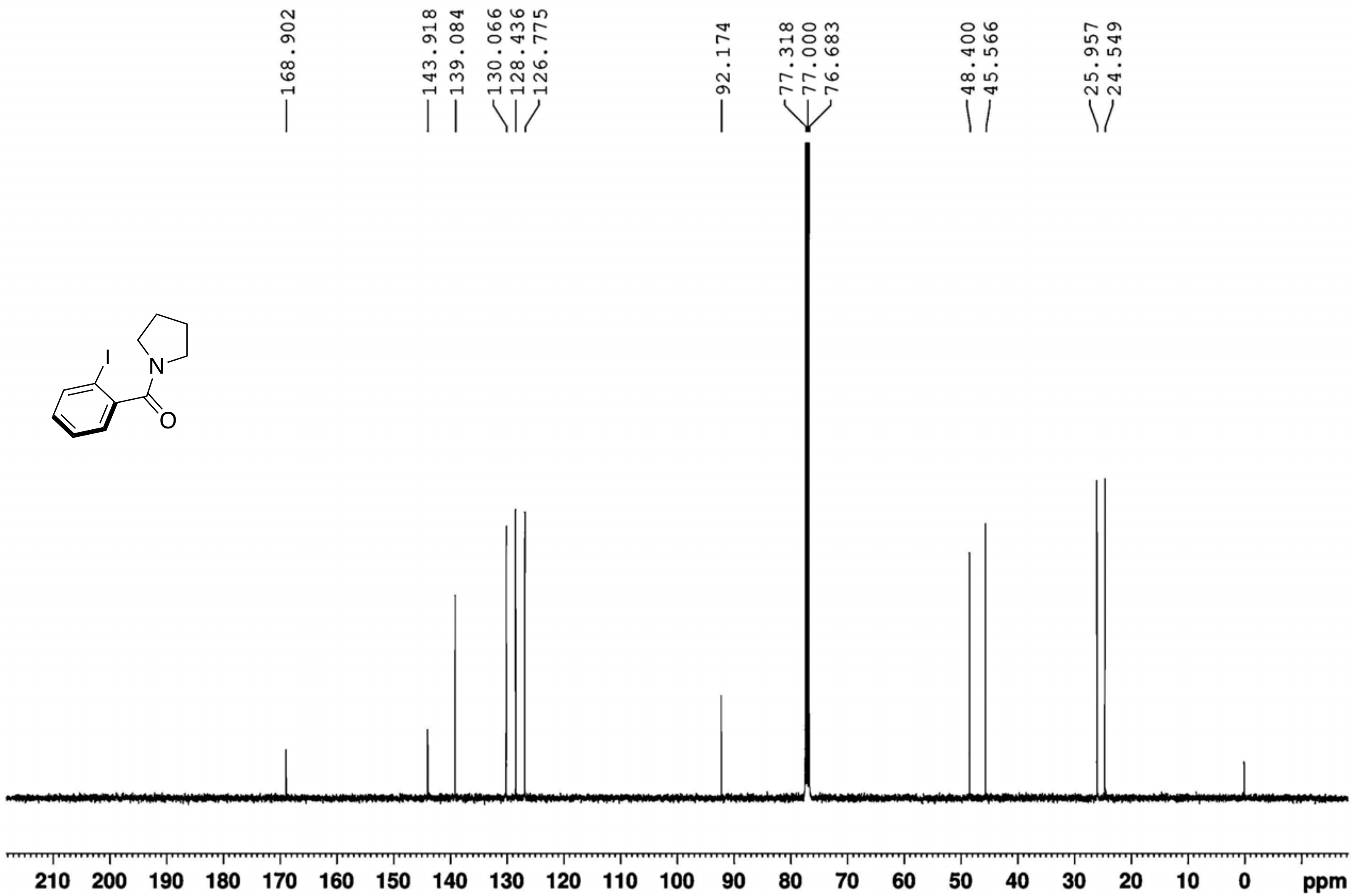

Supplementary Fig. 101 | ^{13}C NMR spectrum of **7a-C**

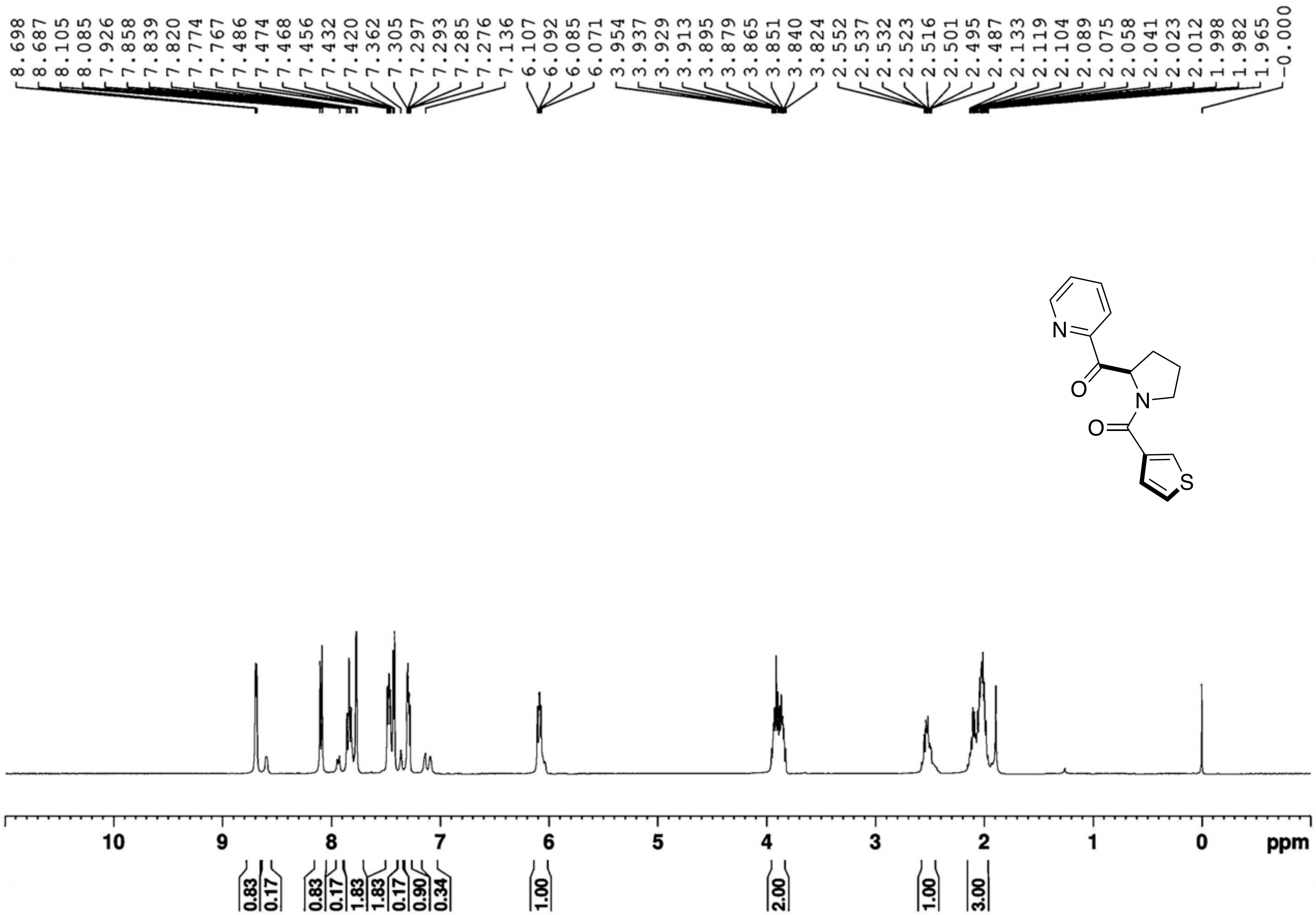

Supplementary Fig. 102 | ^{1}H NMR spectrum of **8ma**

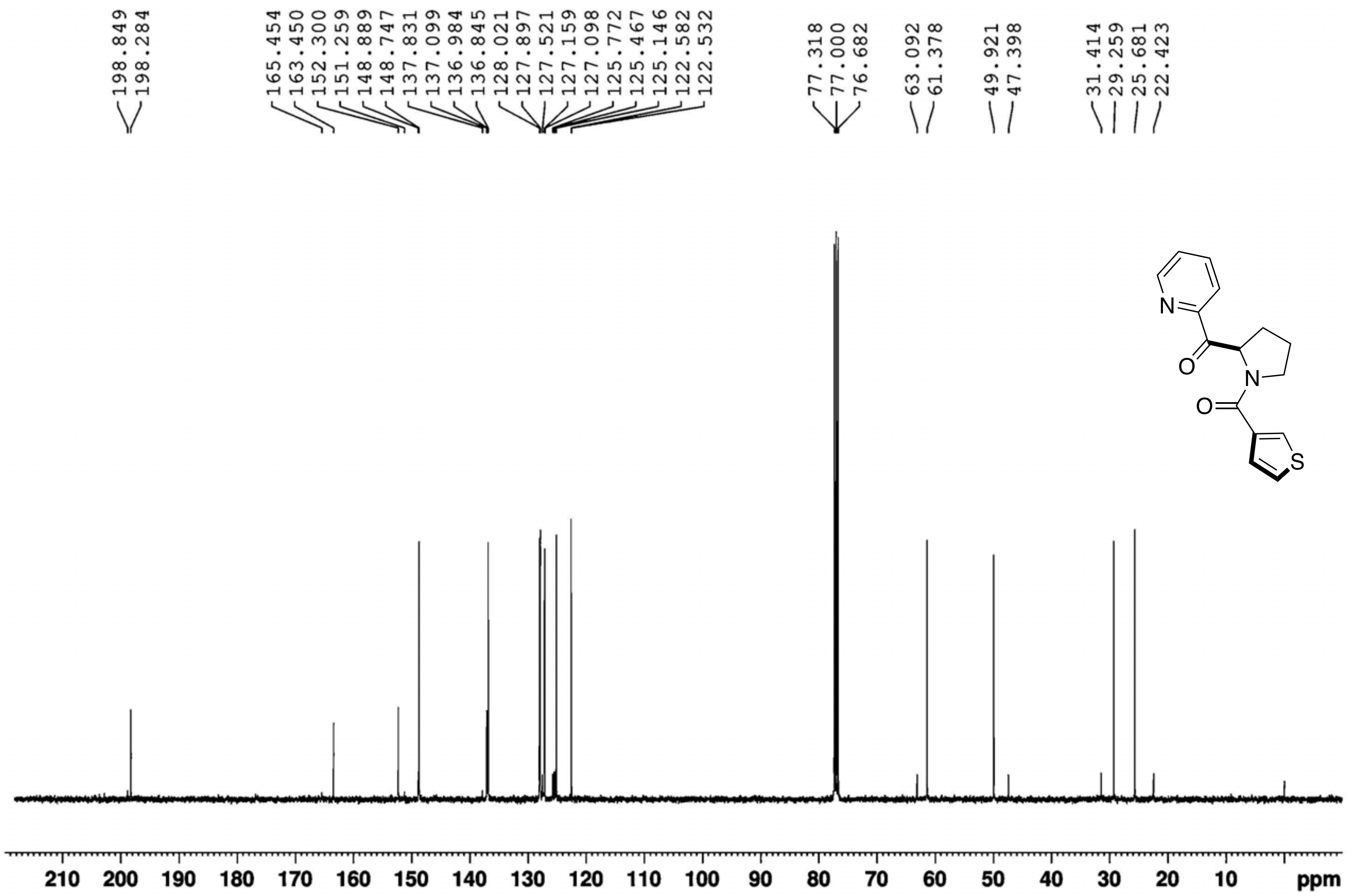

Supplementary Fig. 103 | ^{13}C NMR spectrum of **8ma**

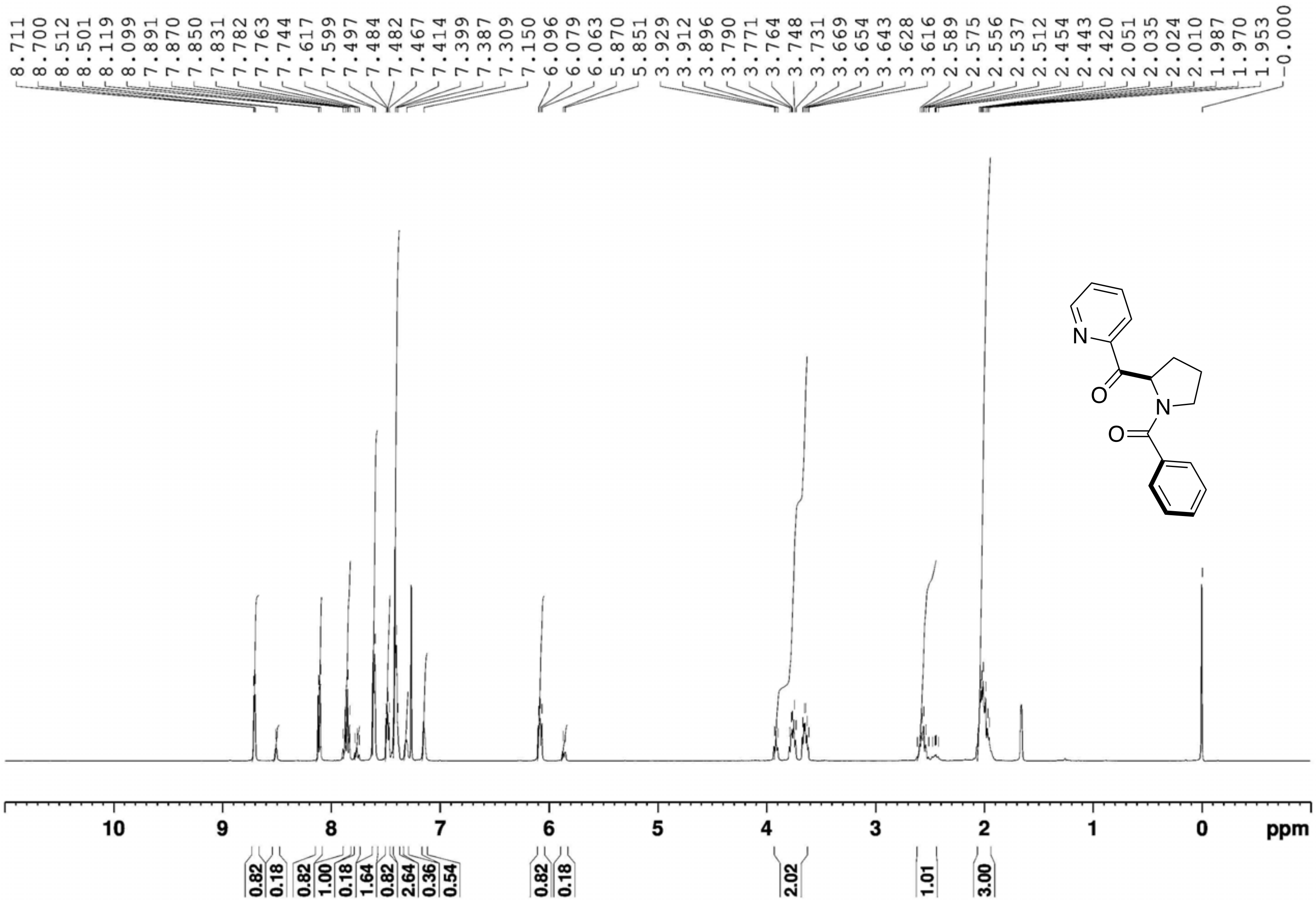

Supplementary Fig. 104 | ^{1}H NMR spectrum of **8ma-C**

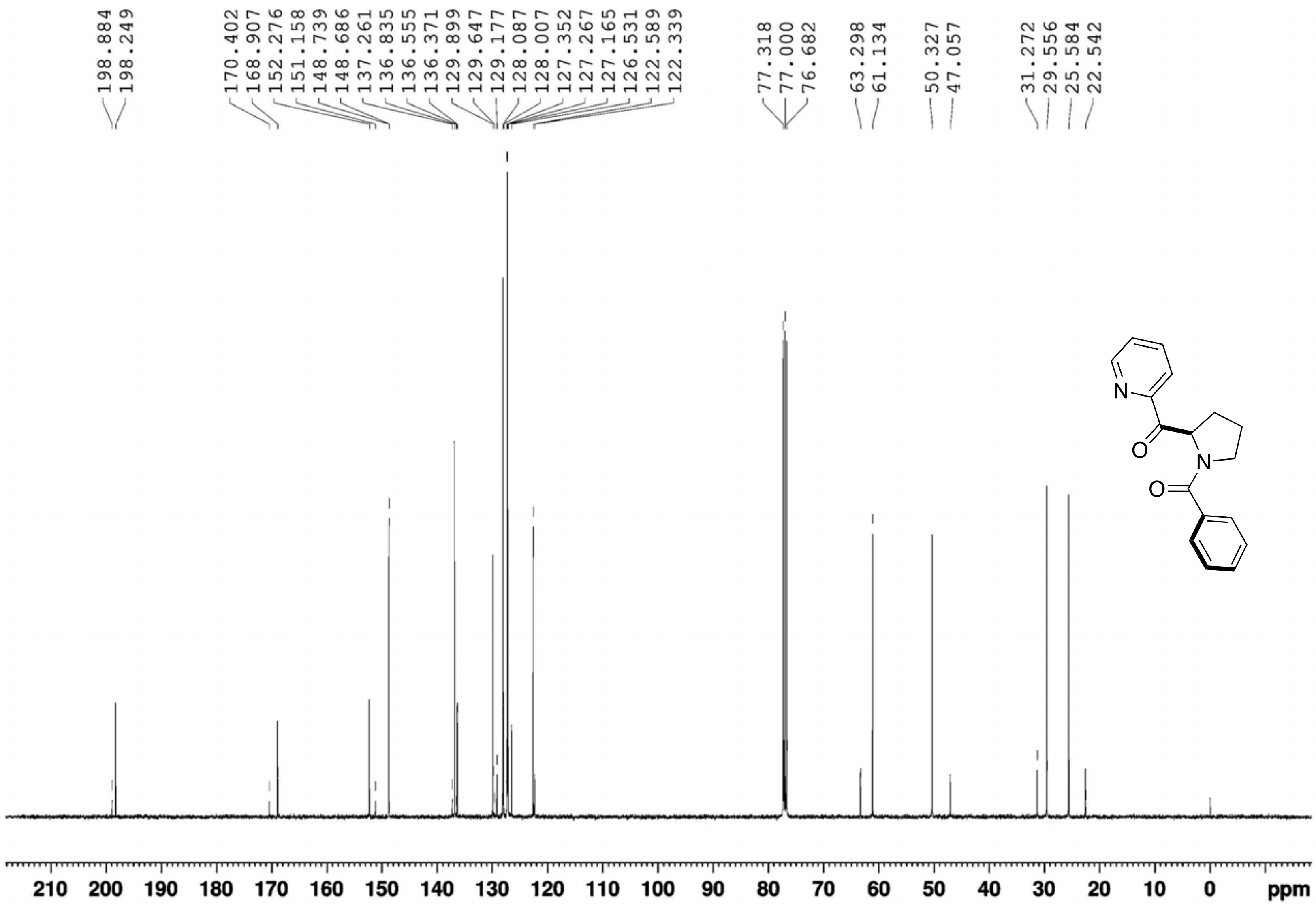

Supplementary Fig. 105 | ^{13}C NMR spectrum of **8ma-C**

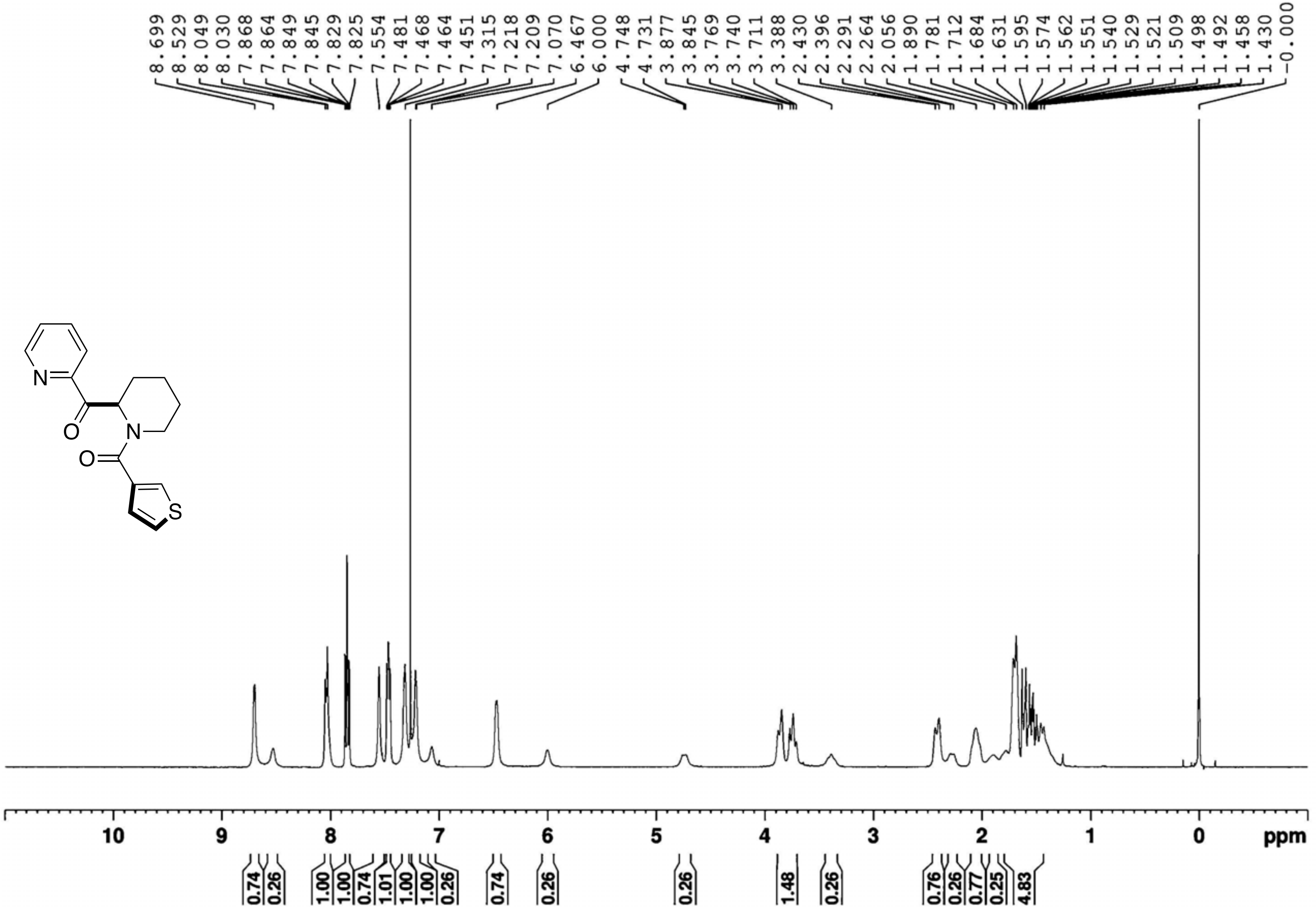

Supplementary Fig. 106 | ^{1}H NMR spectrum of **8mb**

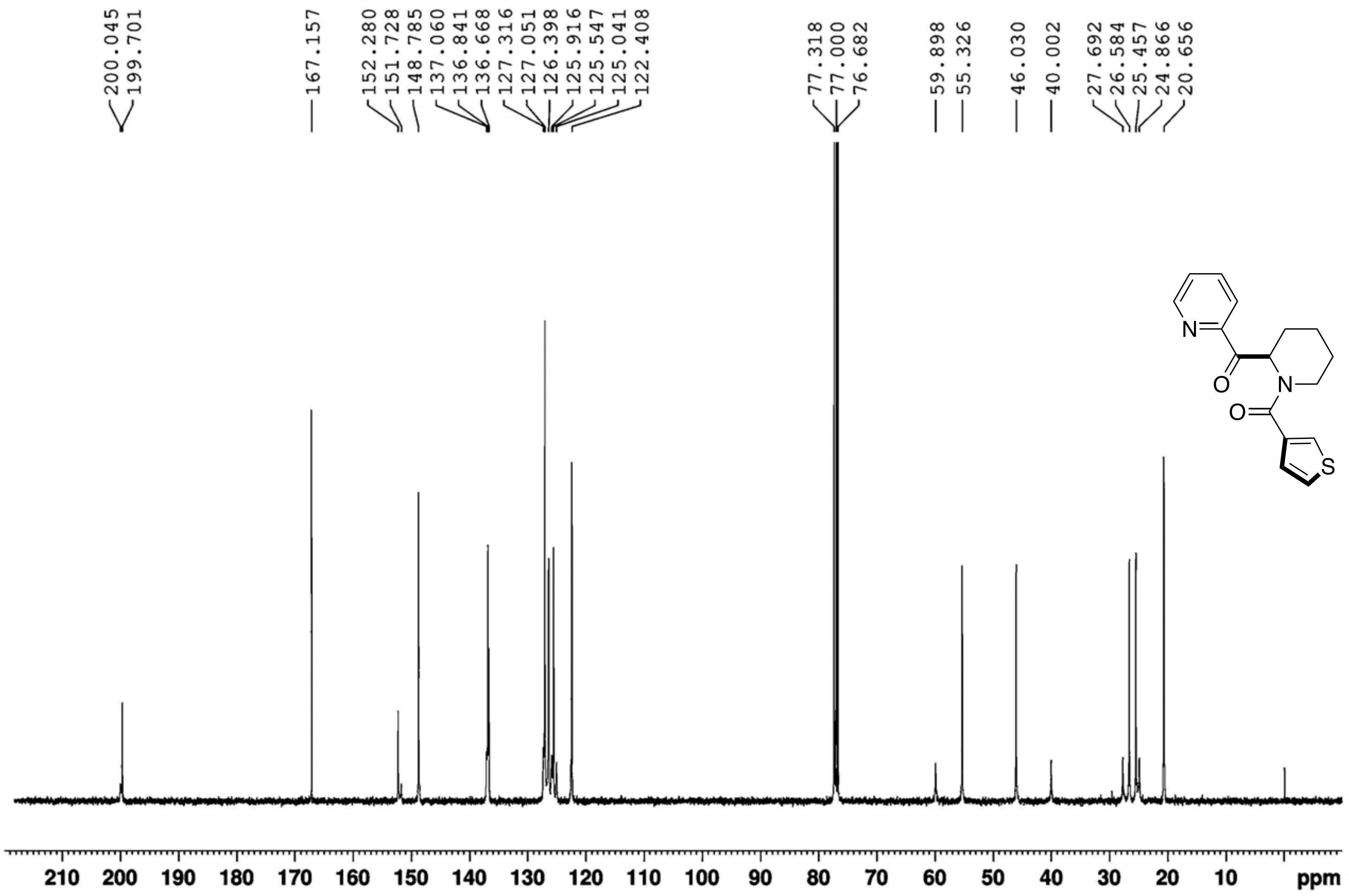

Supplementary Fig. 107 | ^{13}C NMR spectrum of **8mb**

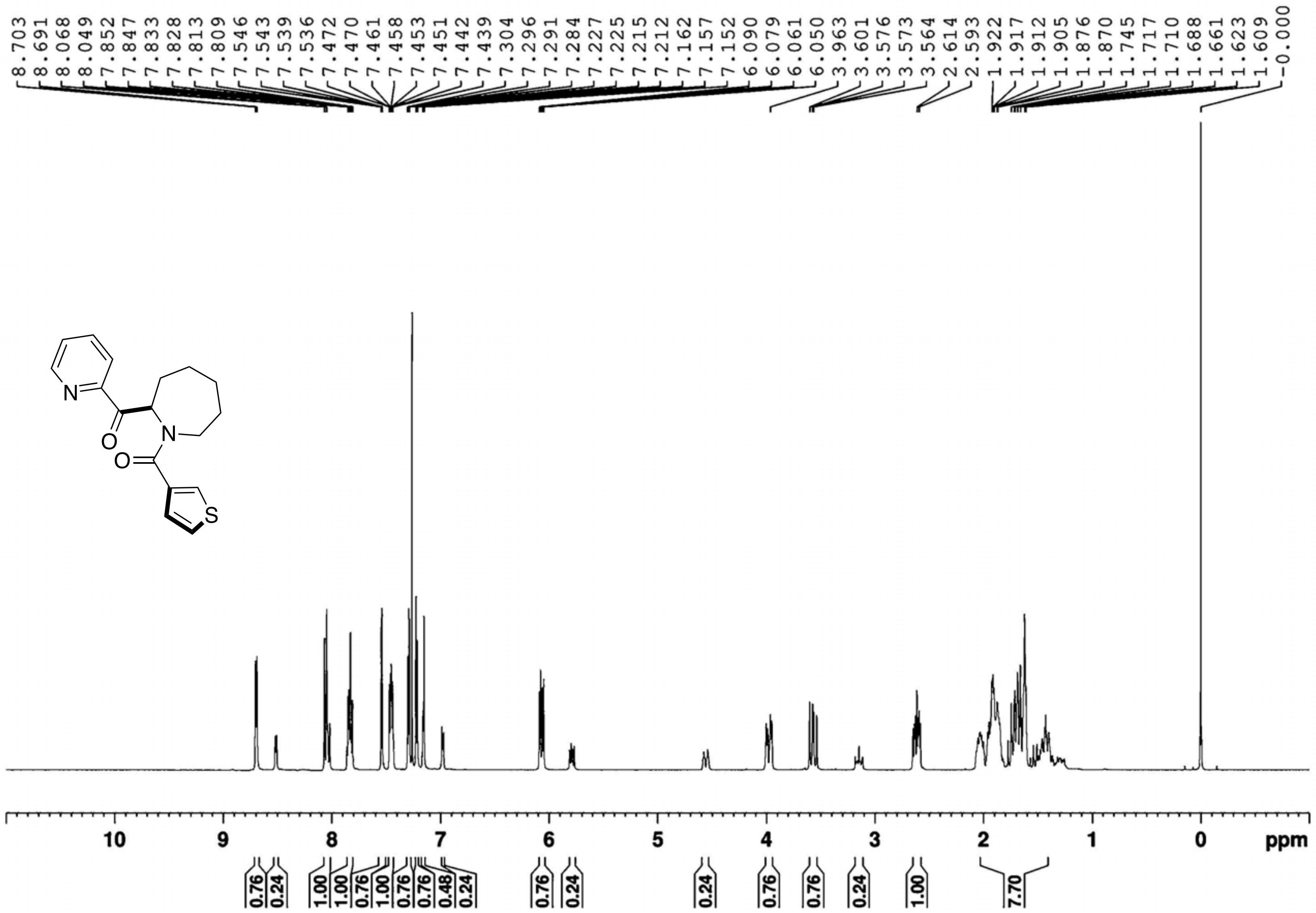

Supplementary Fig. 108 | ^{1}H NMR spectrum of **8mc**

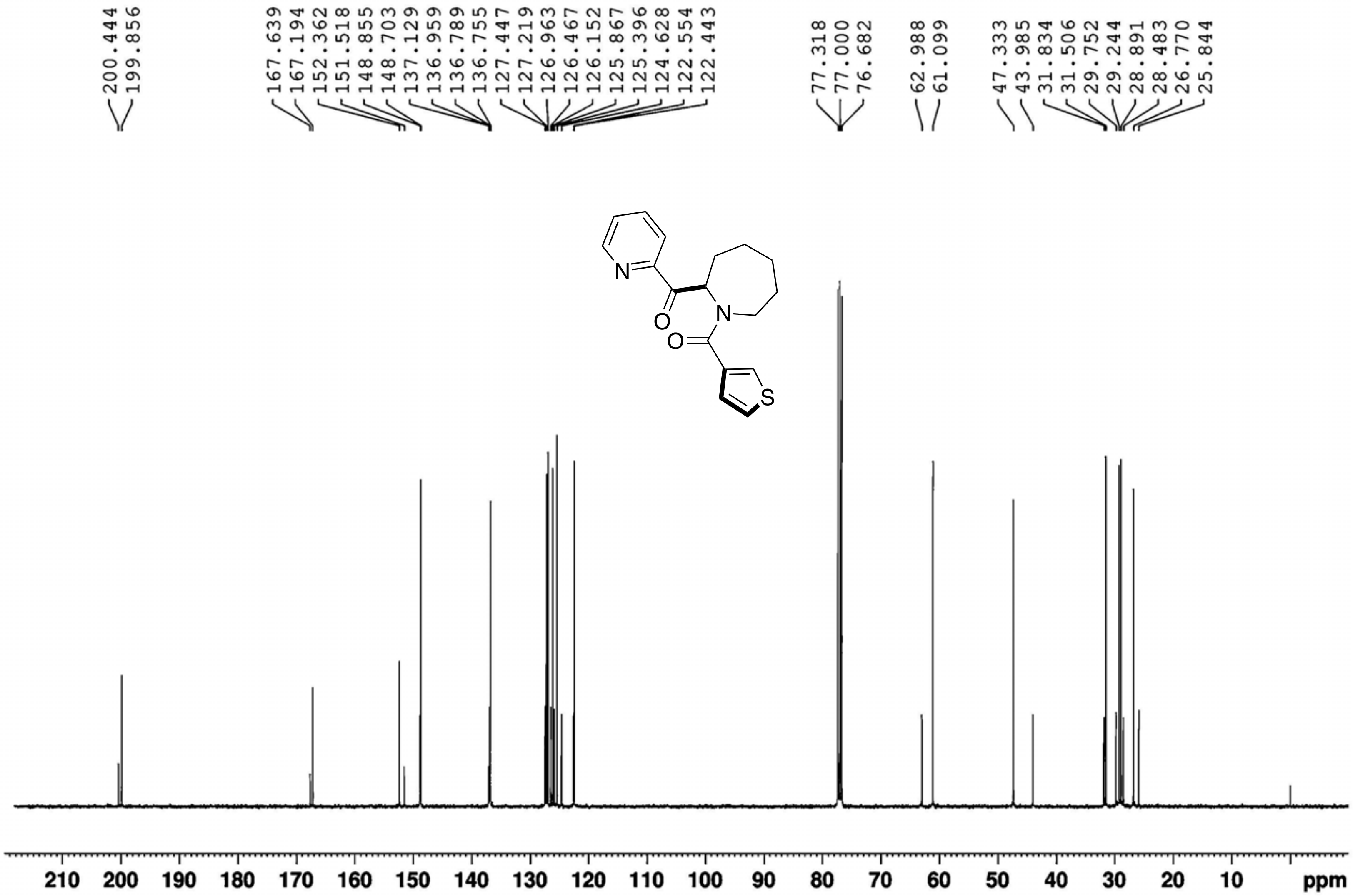

Supplementary Fig. 109 | ^{13}C NMR spectrum of **8mc**

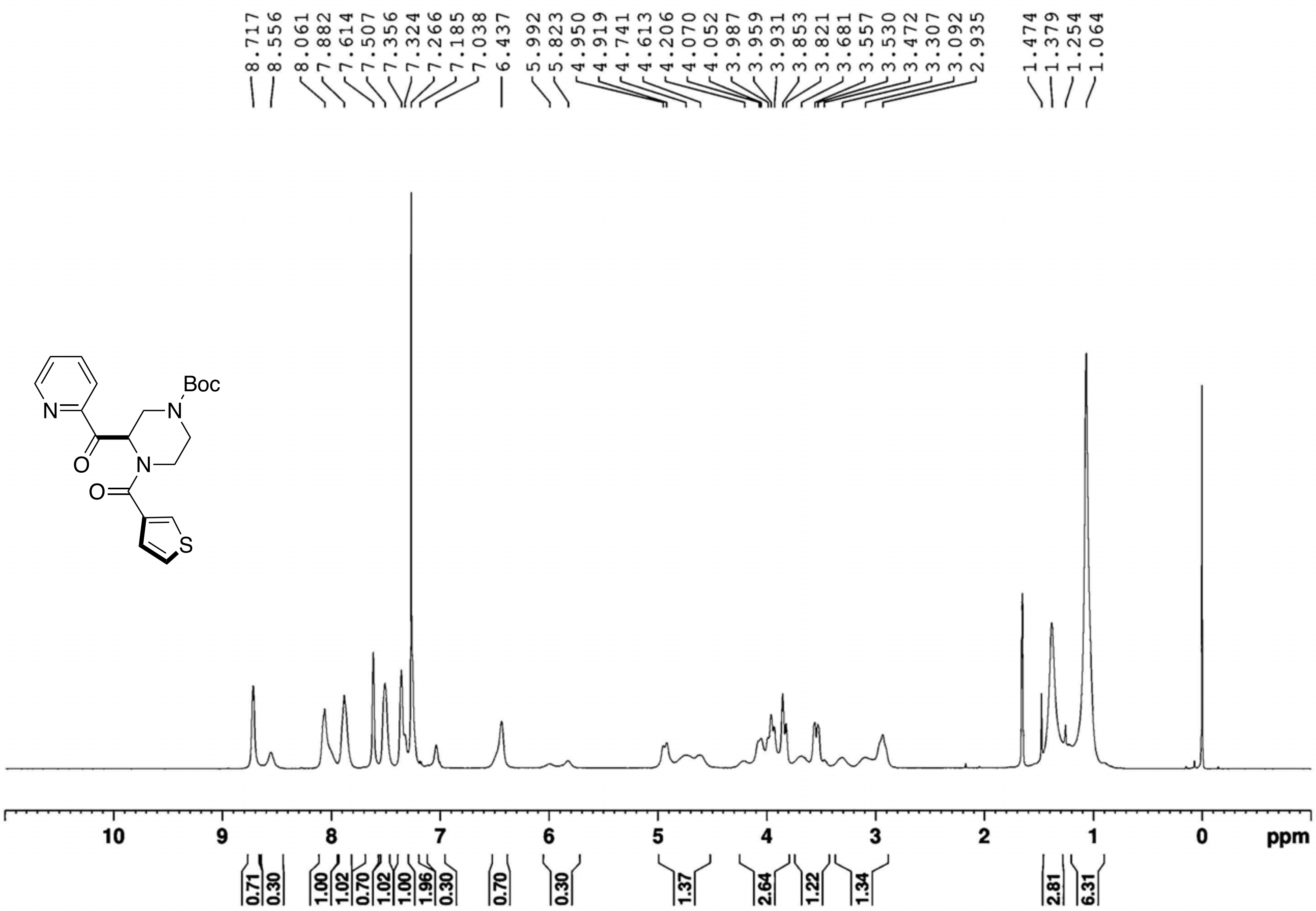

Supplementary Fig. 110 | ^{1}H NMR spectrum of **8md**

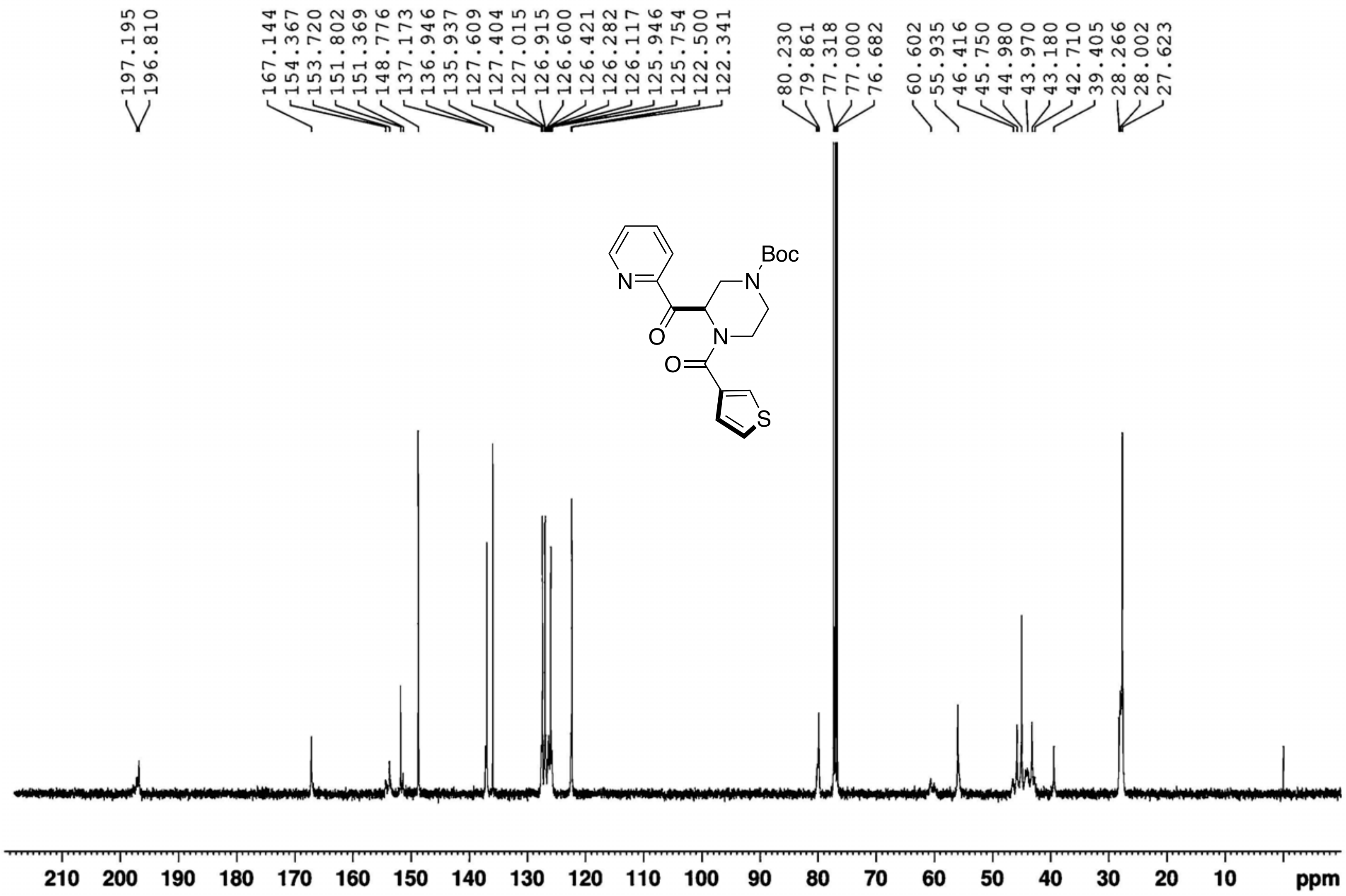

Supplementary Fig. 111 | ^{13}C NMR spectrum of **8md**

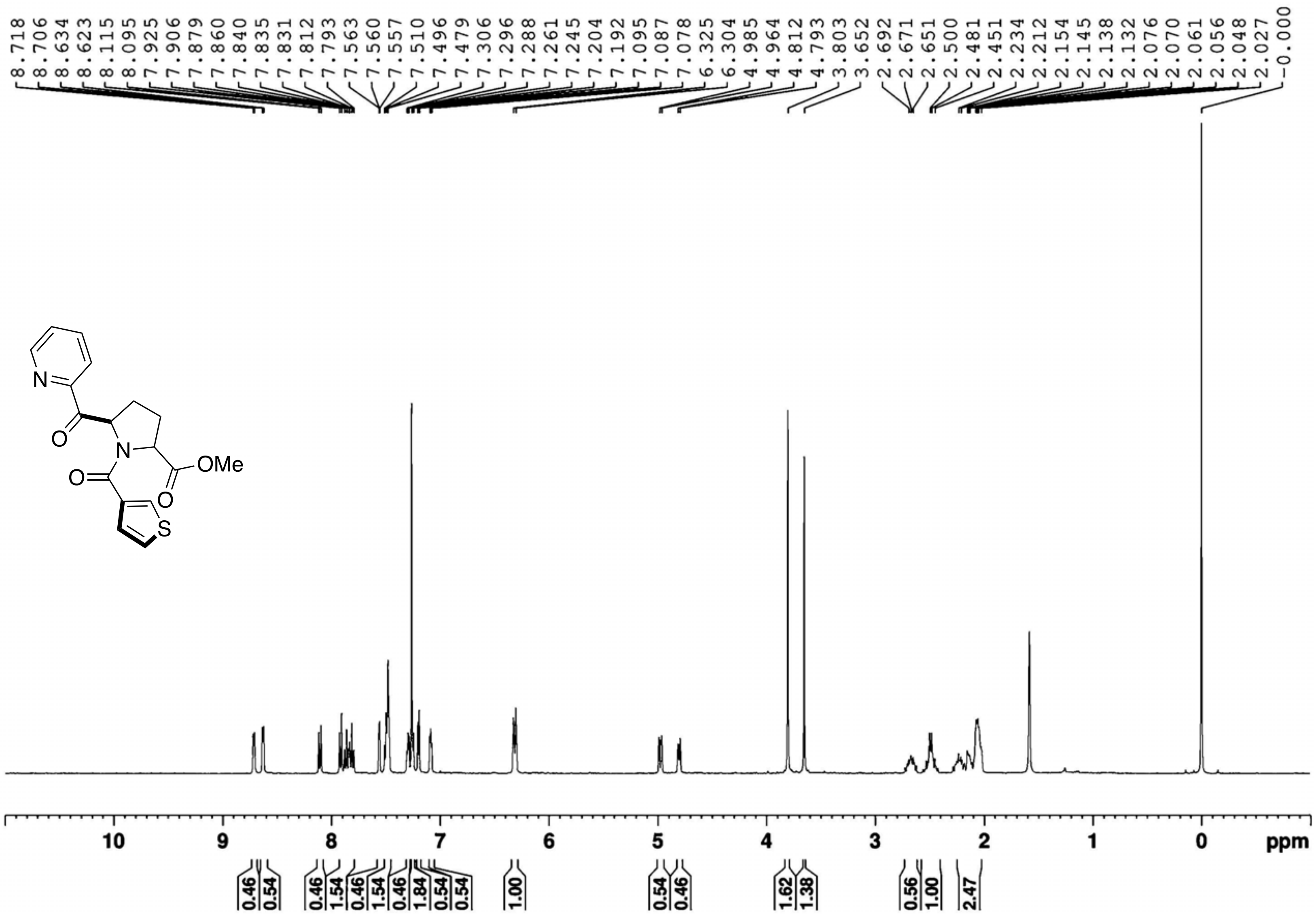

Supplementary Fig. 112 | ^{1}H NMR spectrum of **8me**

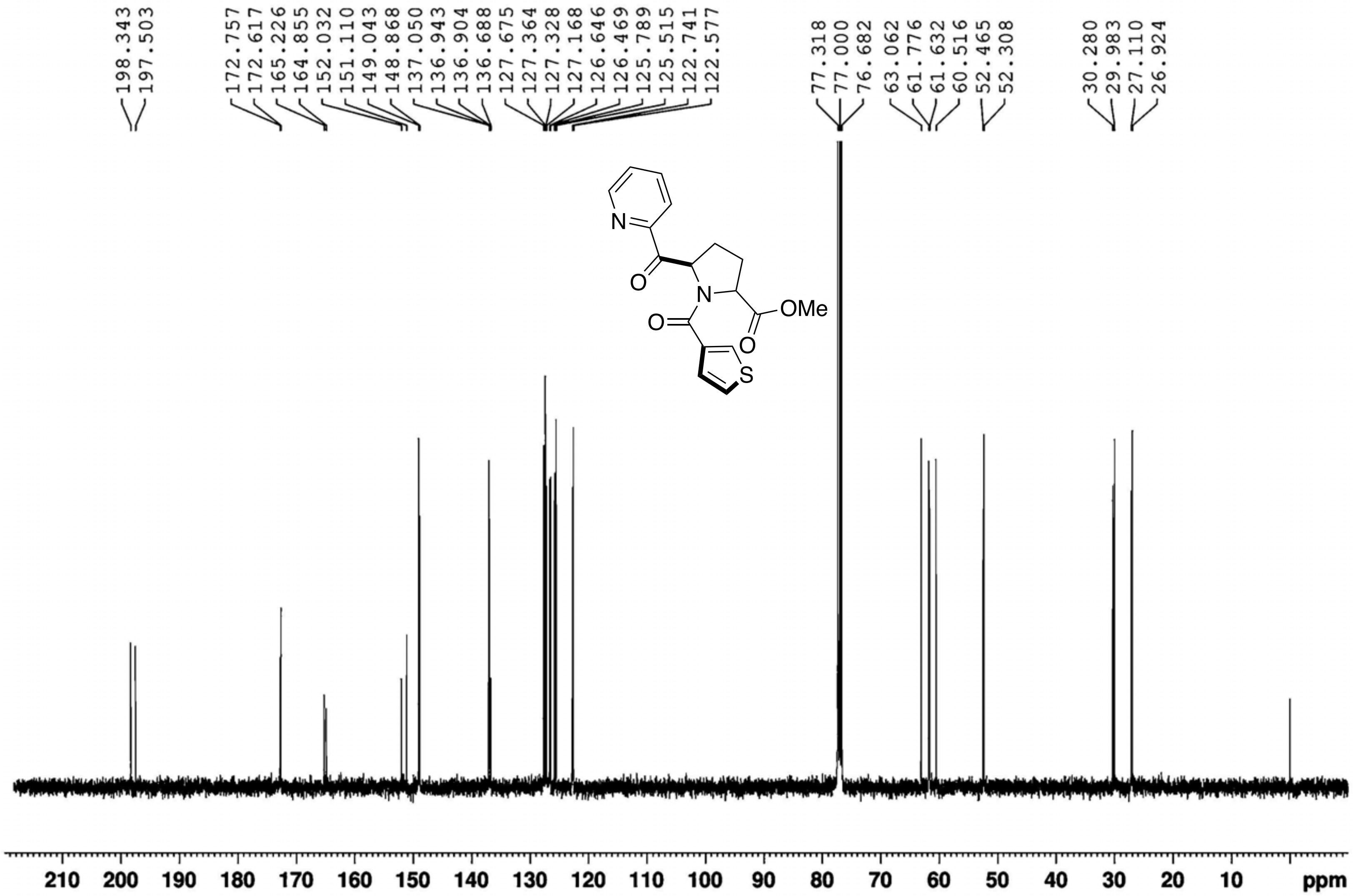

Supplementary Fig. 113 | ^{13}C NMR spectrum of **8me**

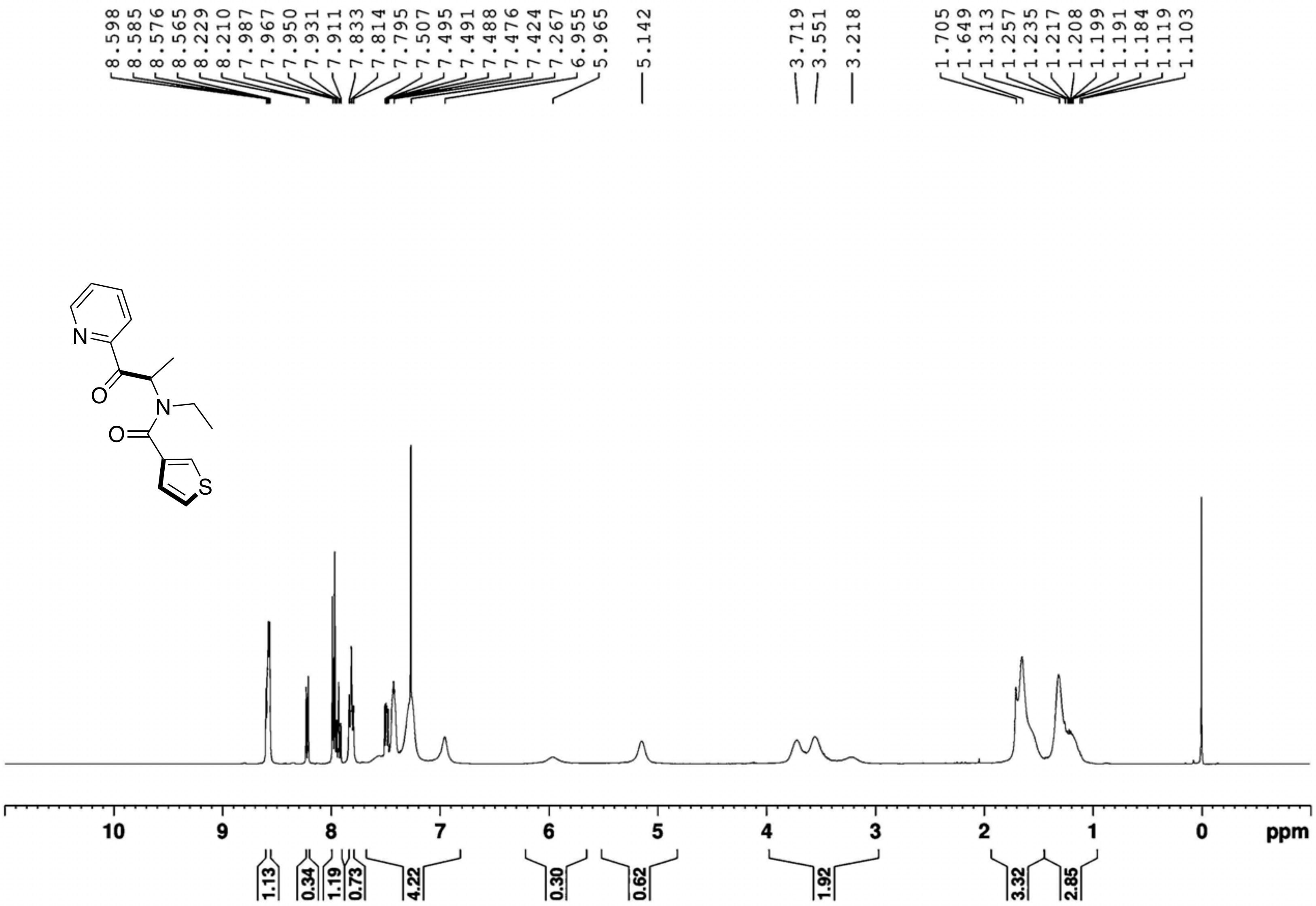

Supplementary Fig. 114 | ^{1}H NMR spectrum of **8mf**

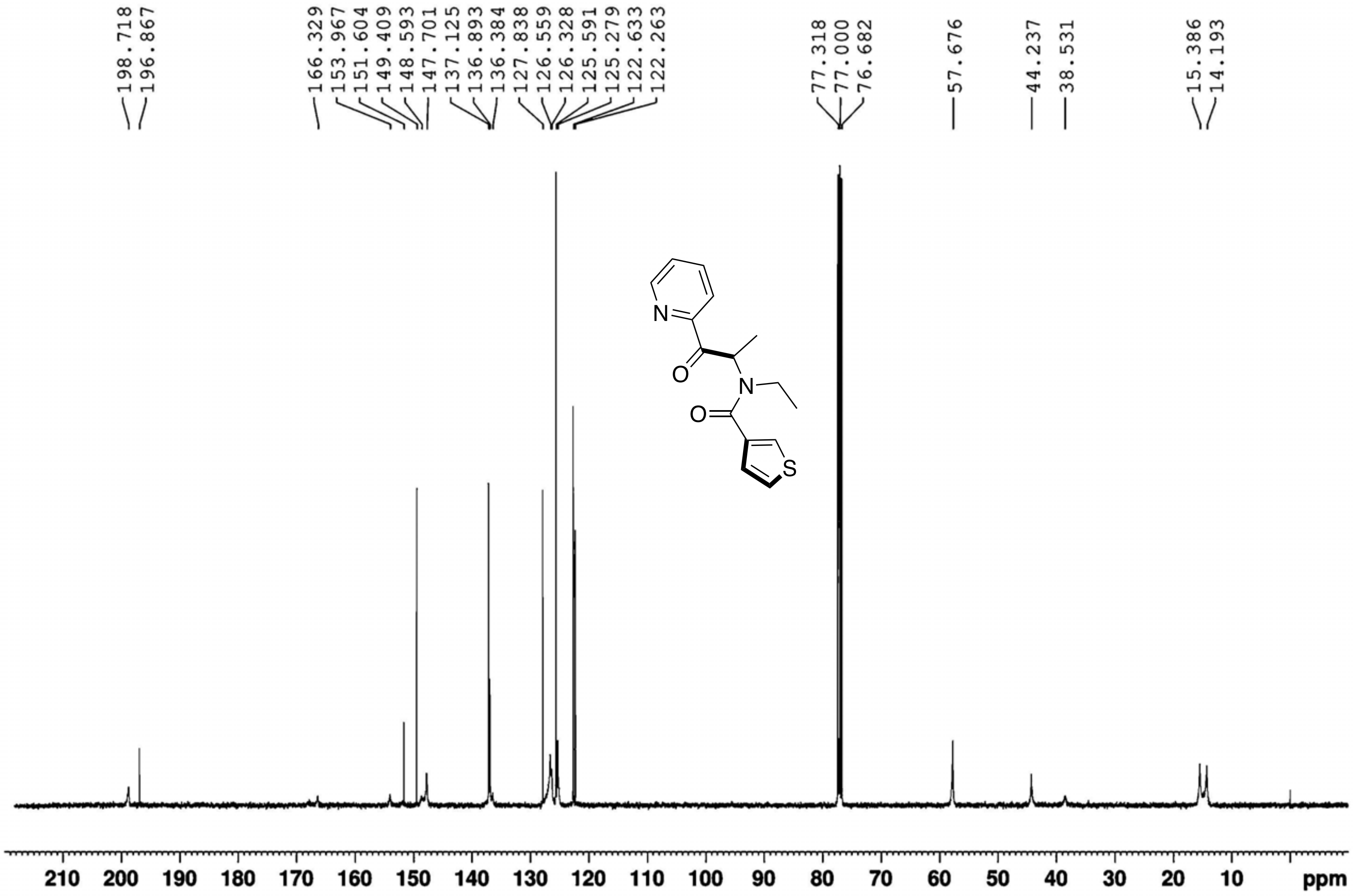

Supplementary Fig. 115 | ^{13}C NMR spectrum of **8mf**

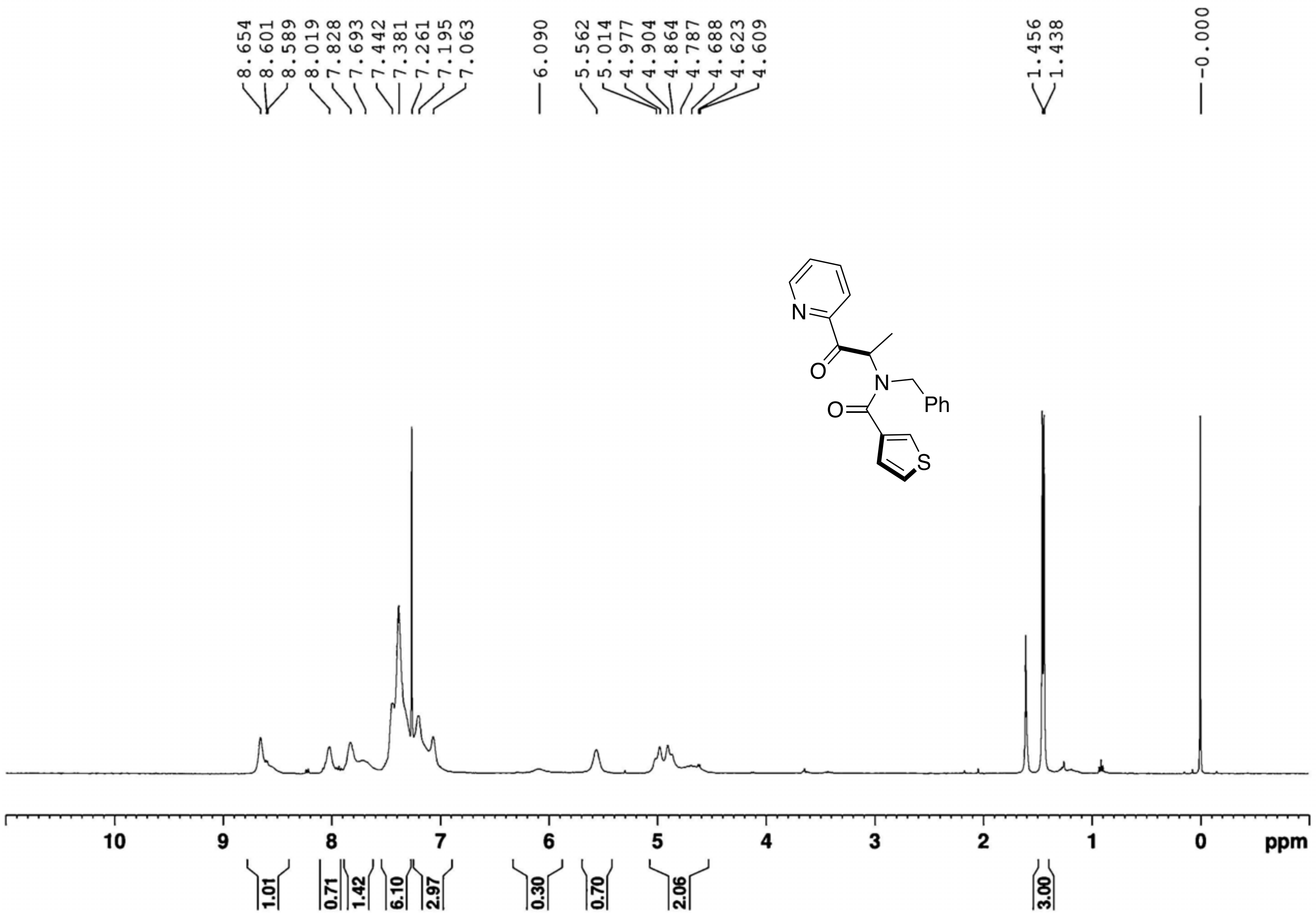

Supplementary Fig. 116 | ^{1}H NMR spectrum of **8mg-major**

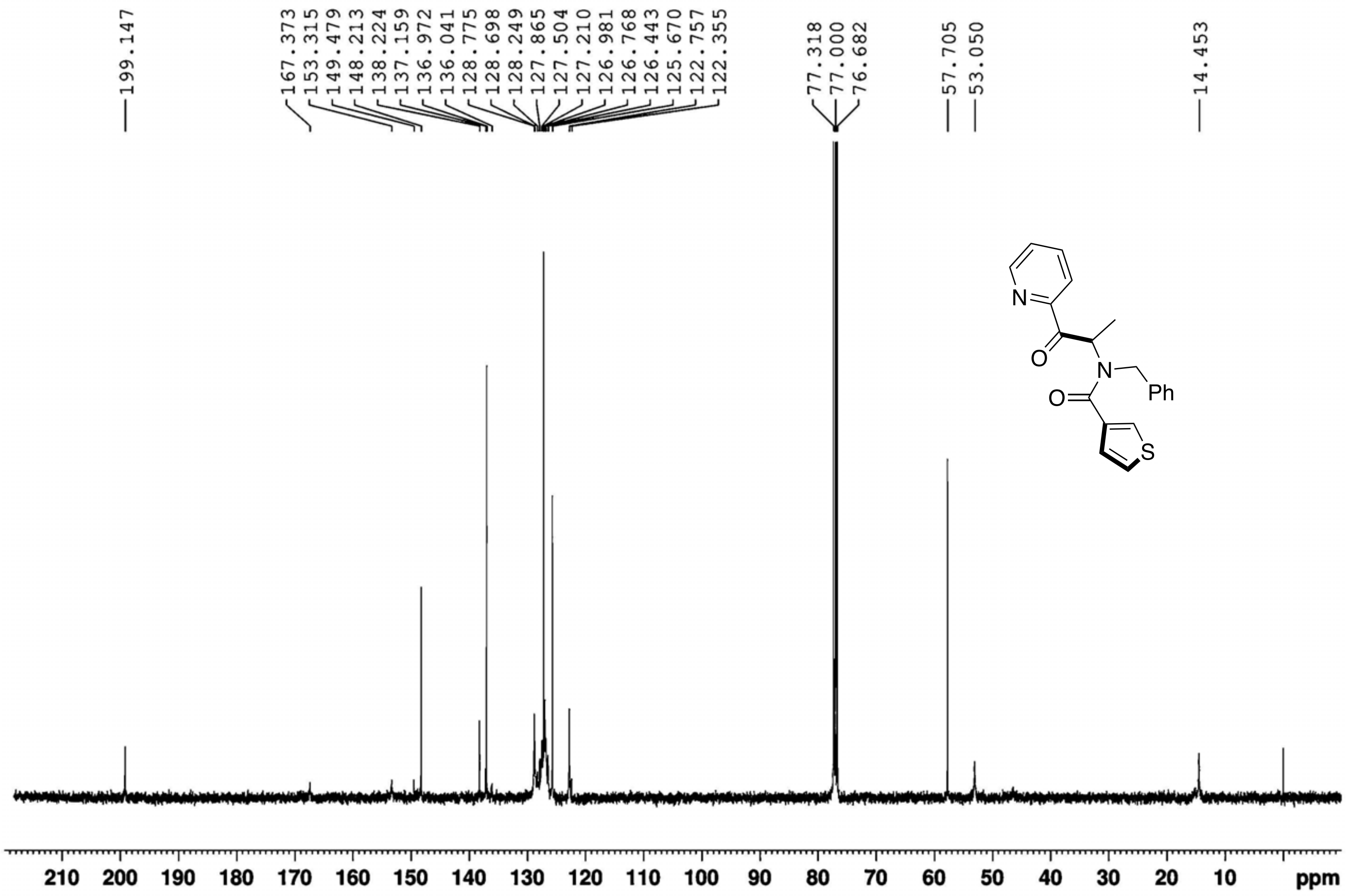

Supplementary Fig. 117 | ^{13}C NMR spectrum of **8mg-major**

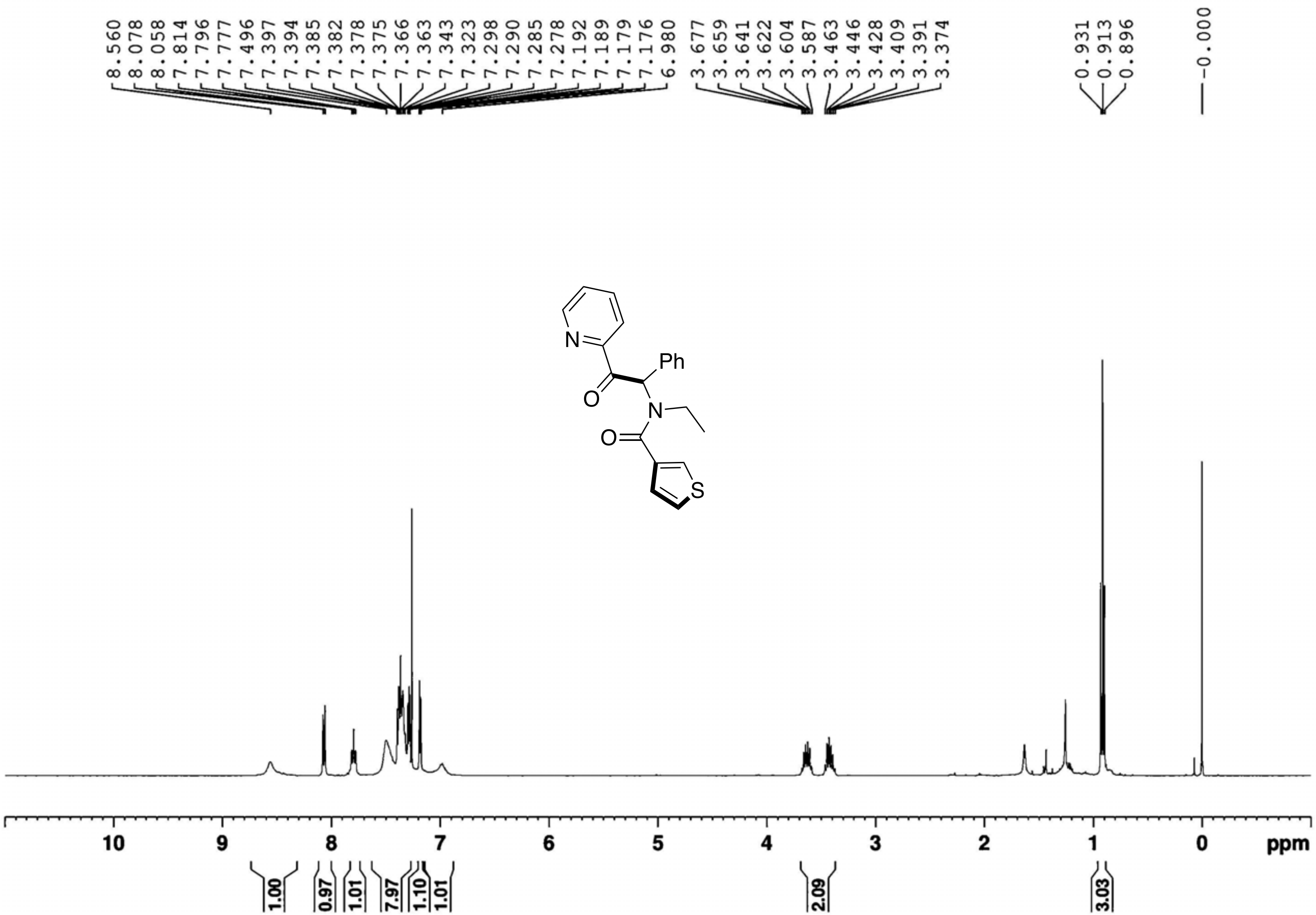

Supplementary Fig. 118 | ^{1}H NMR spectrum of **8mg-minor**

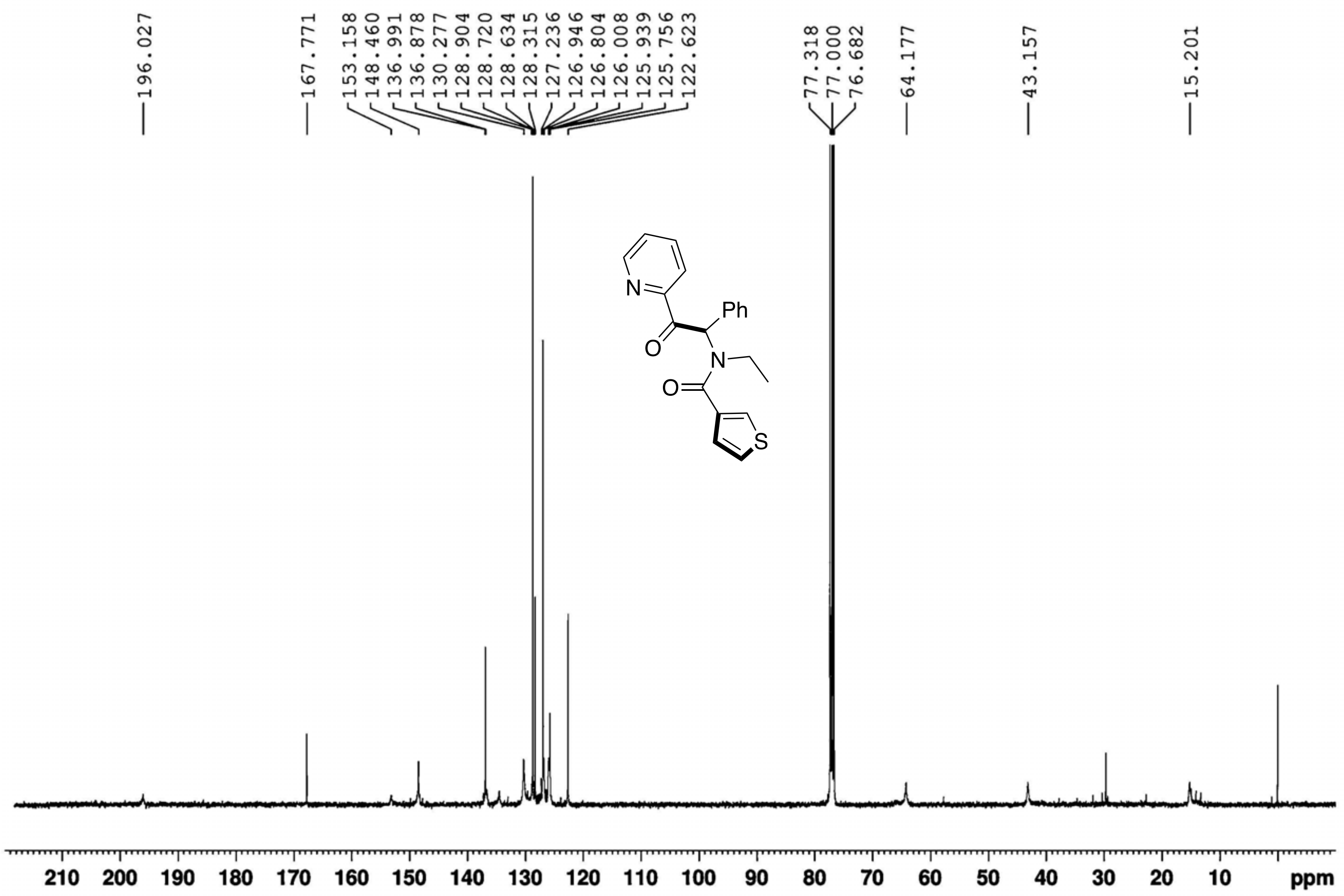

Supplementary Fig. 119 | ^{13}C NMR spectrum of **8mg-minor**

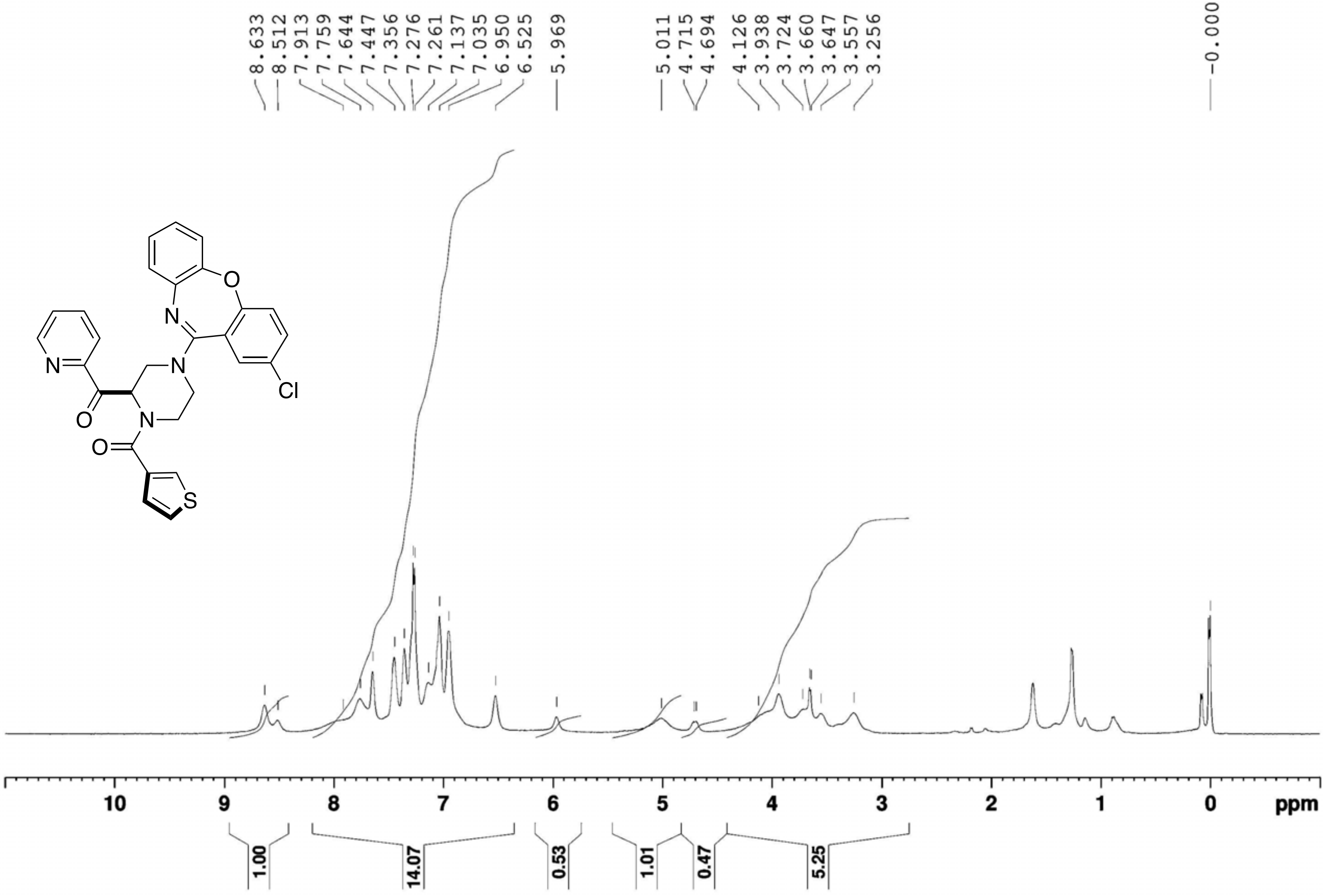

Supplementary Fig. 120 | ^{1}H NMR spectrum of **8mh**

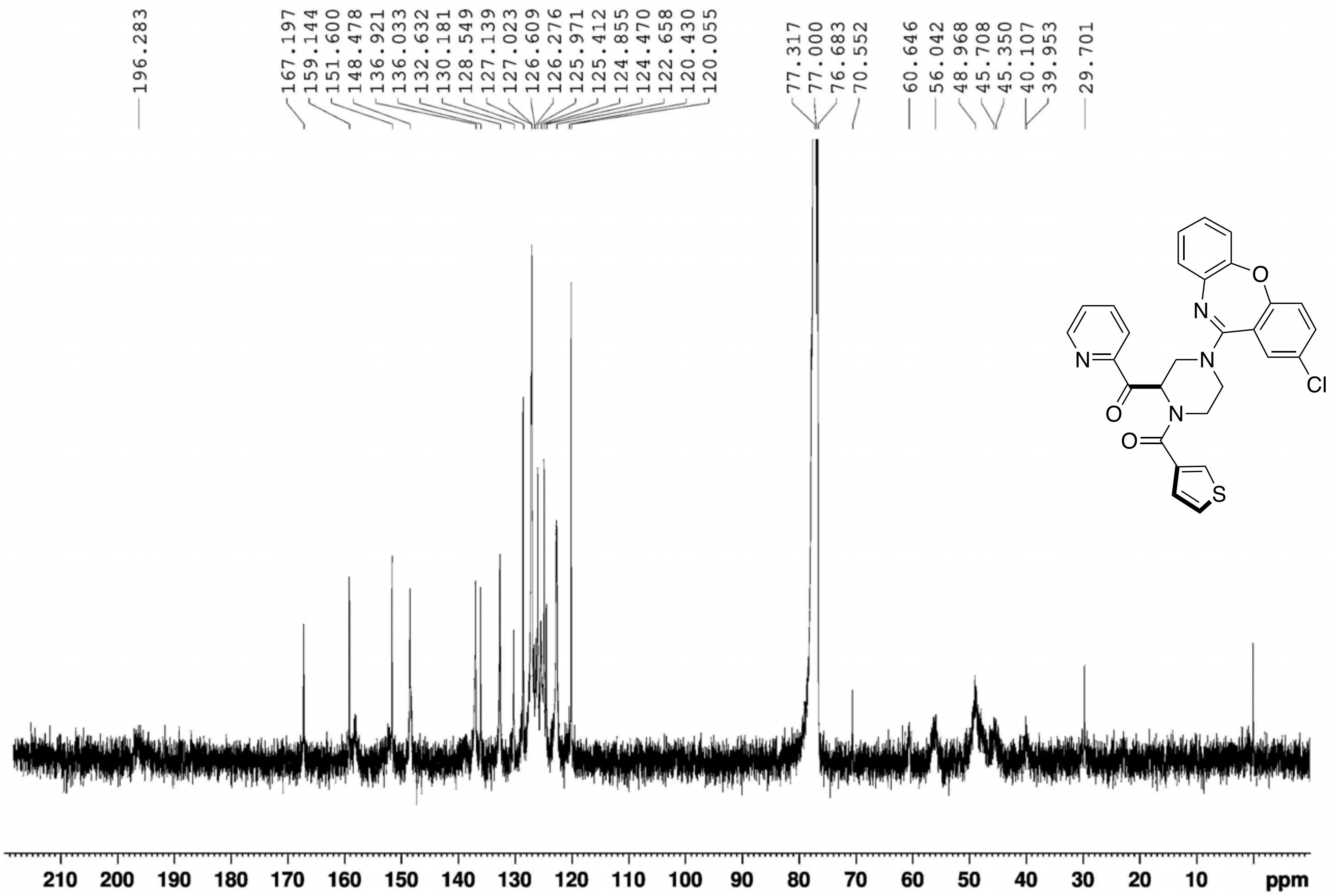

Supplementary Fig. 121 | ^{13}C NMR spectrum of **8mh**

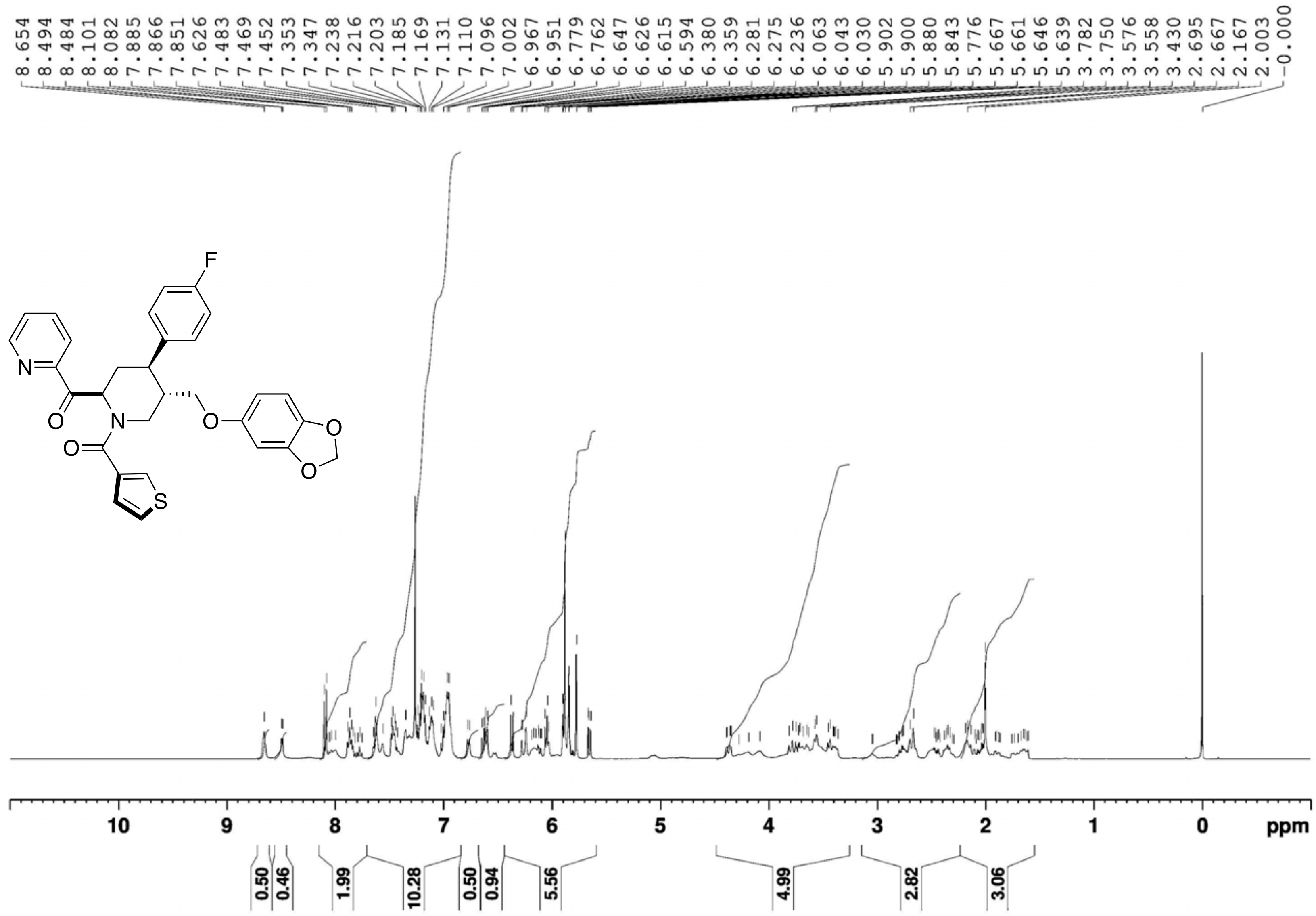

Supplementary Fig. 122 | ^{1}H NMR spectrum of **8mi**

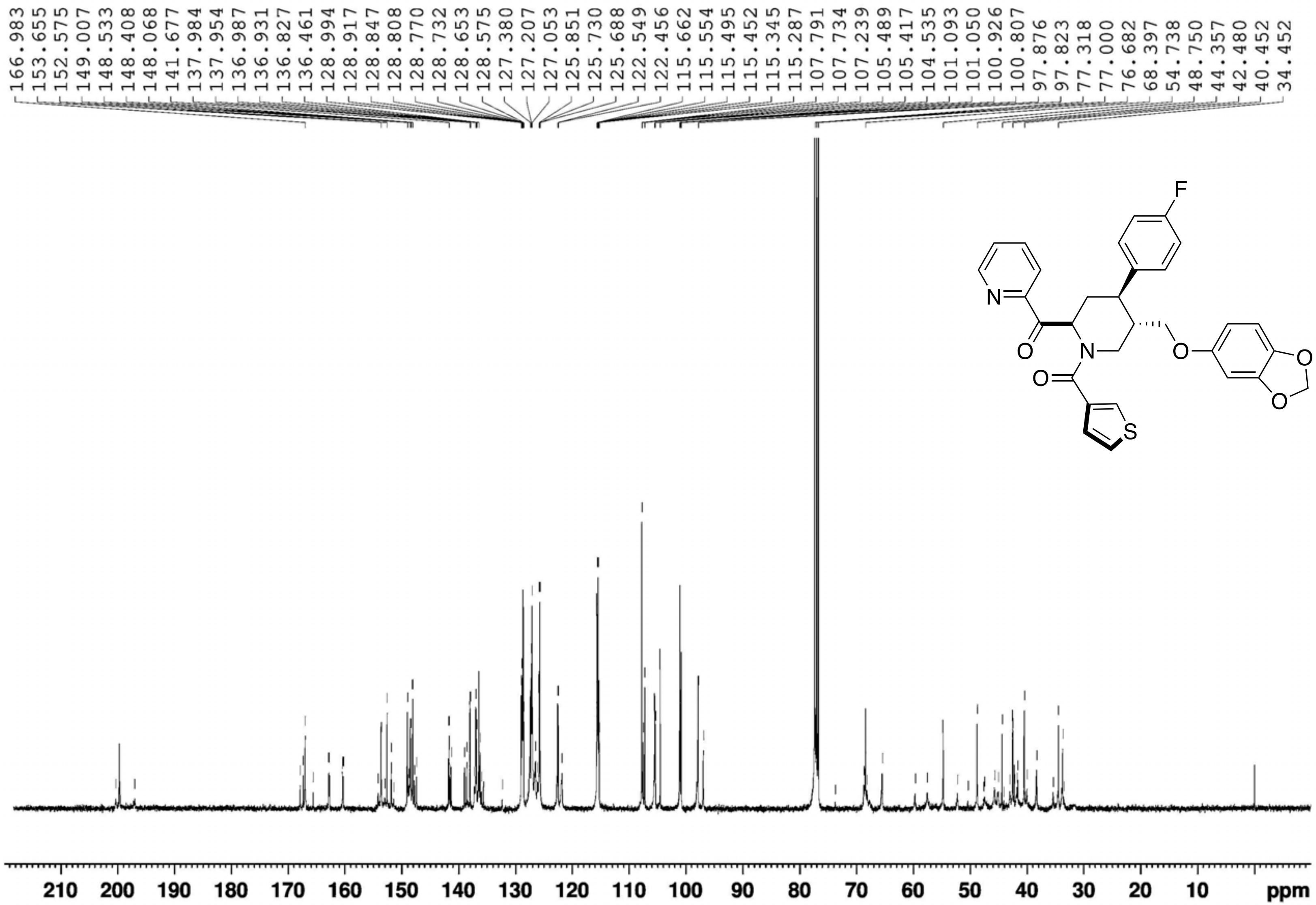

Supplementary Fig. 123 | ^{13}C NMR spectrum of **8mi** (whole)

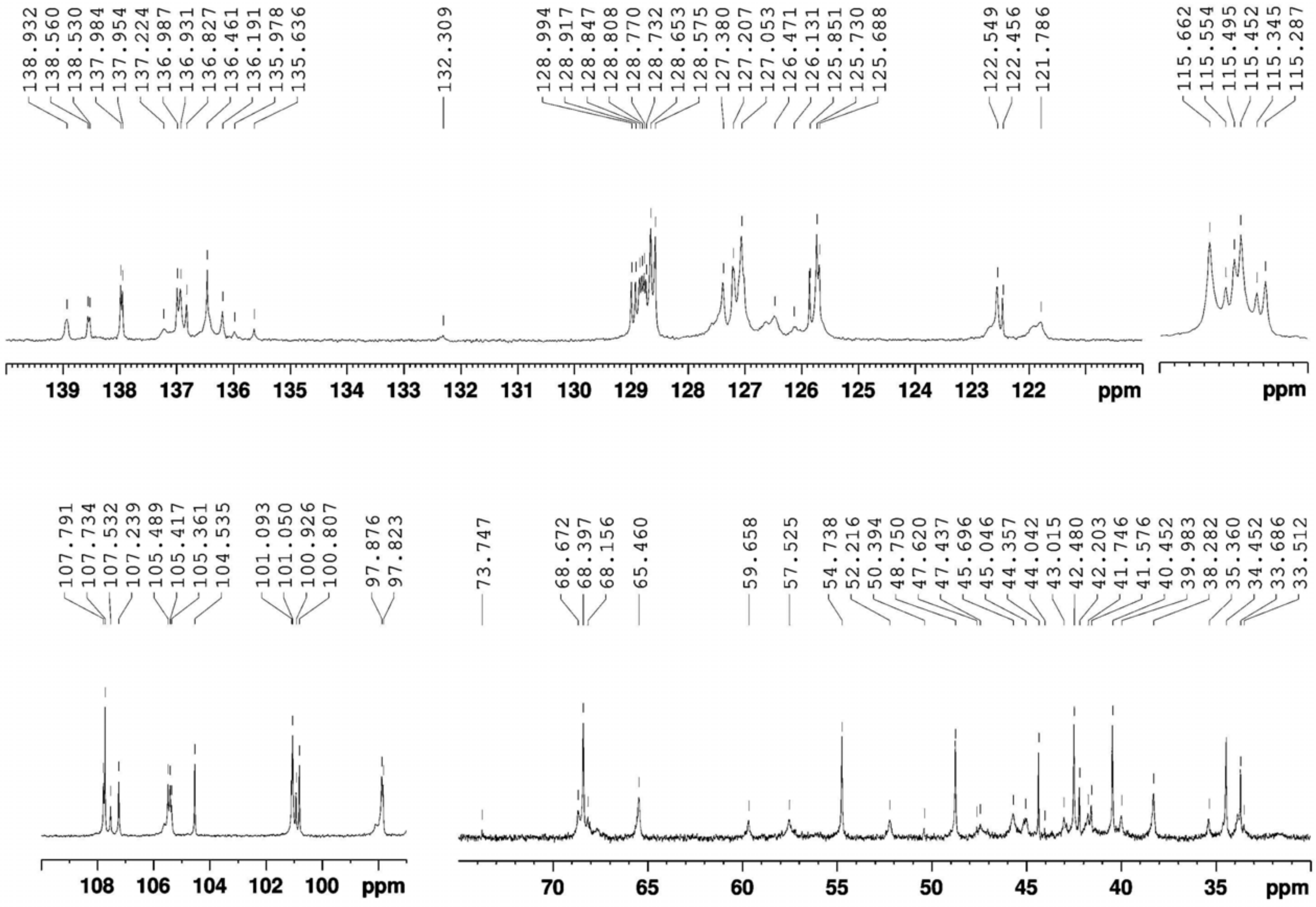

Supplementary Fig. 124 | ^{13}C NMR spectrum of **8mi**

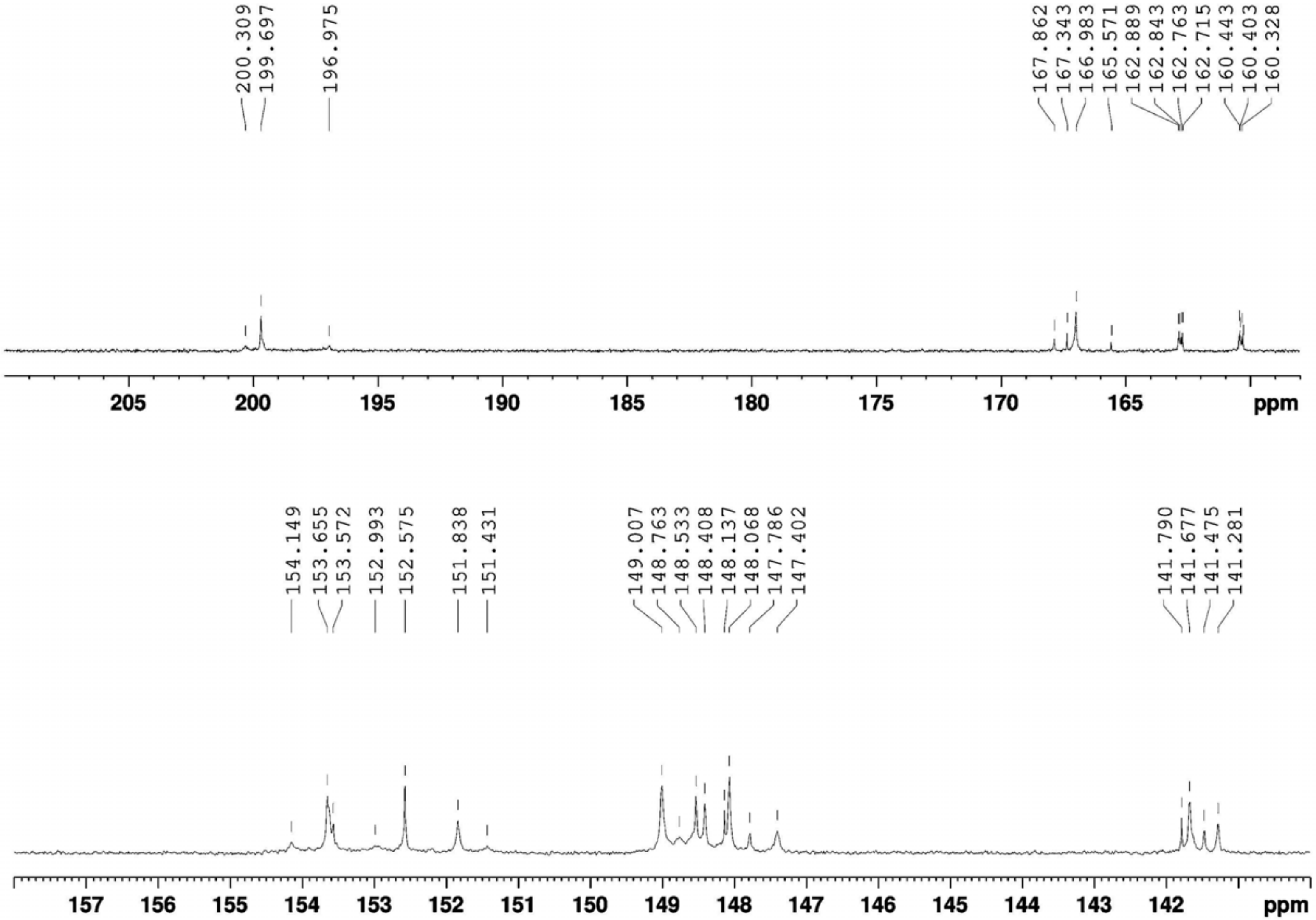

Supplementary Fig. 125 | ^{13}C NMR spectrum of **8mi**

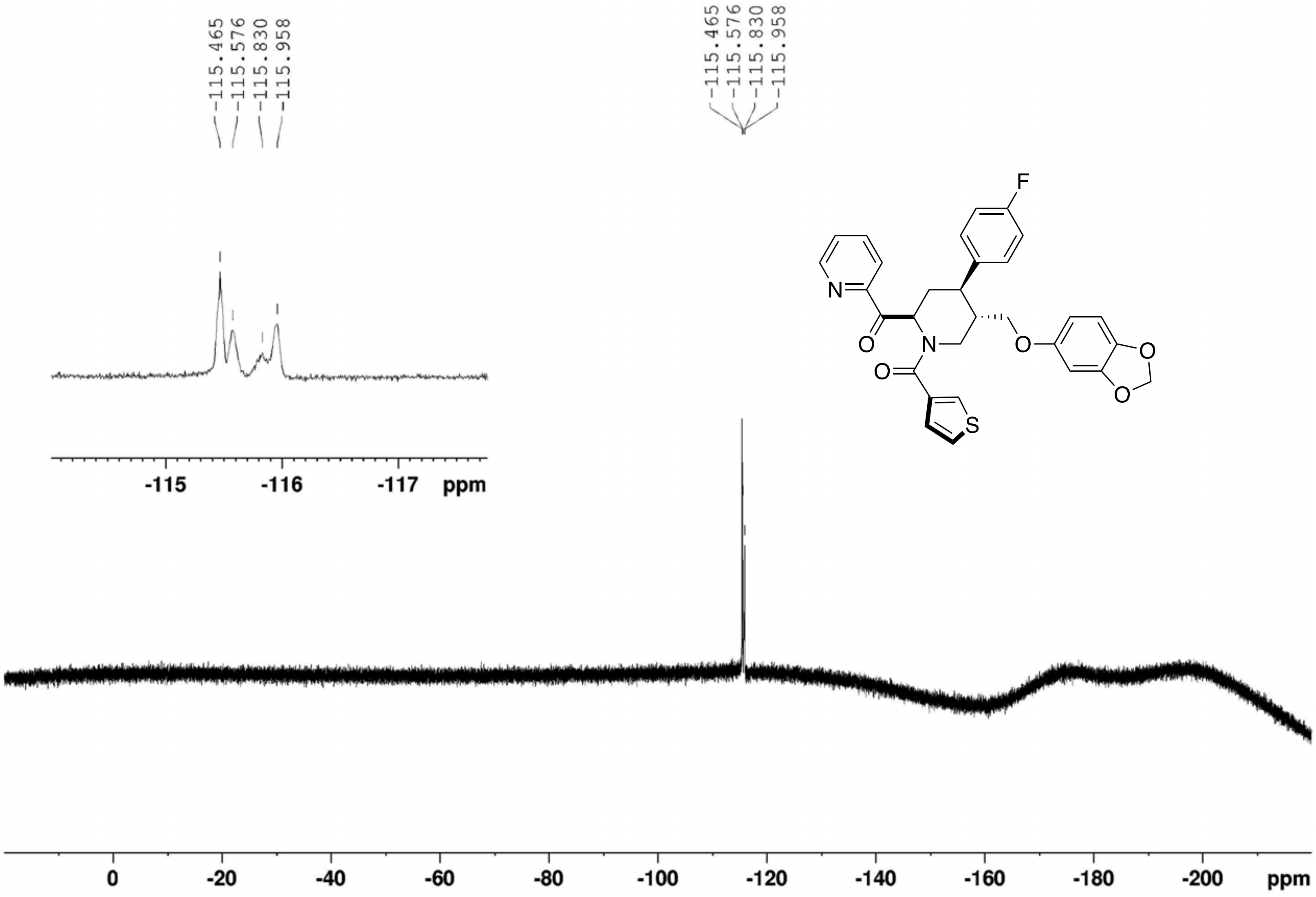

Supplementary Fig. 126 | ^{19}F NMR spectrum of **8mi**

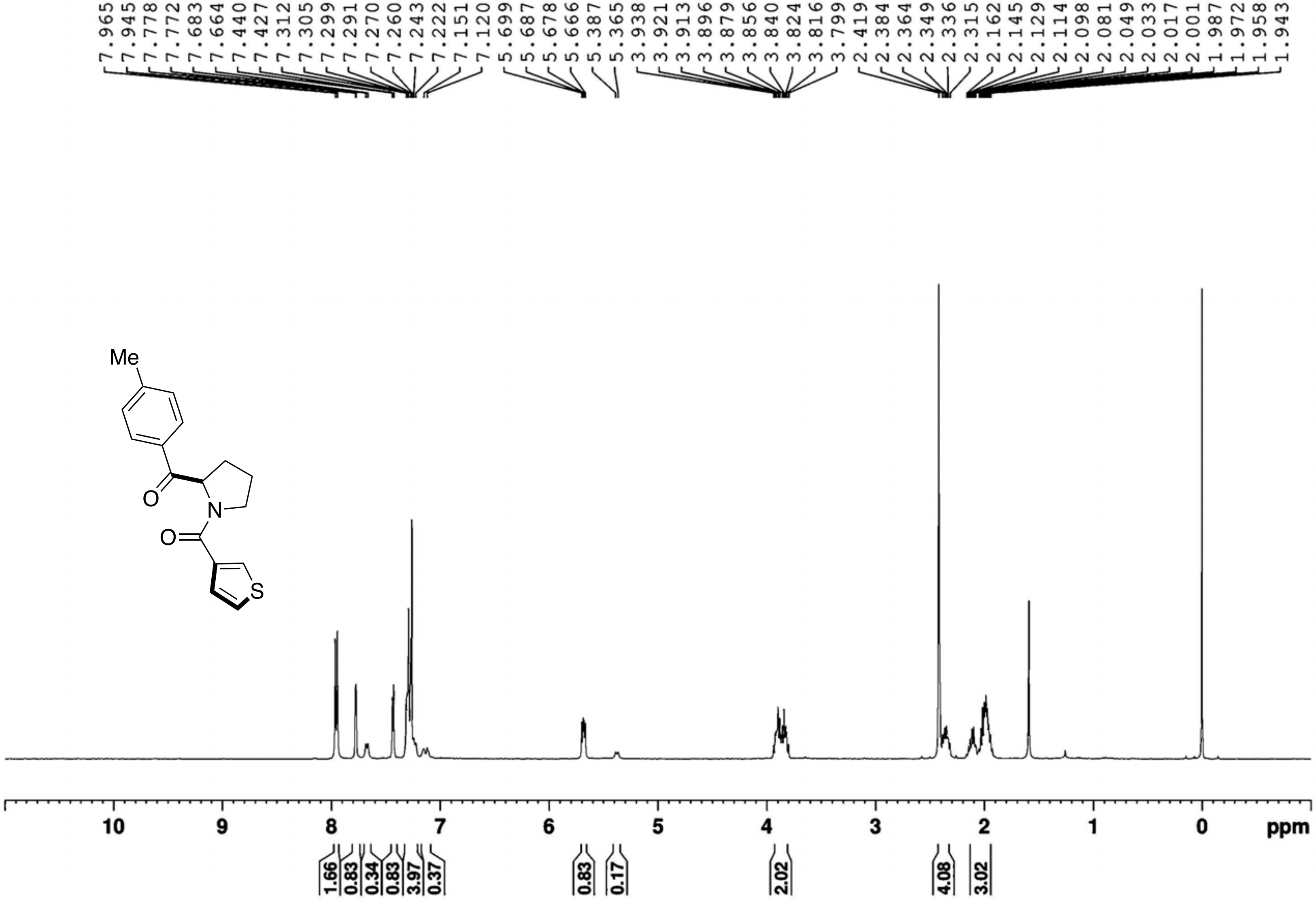

Supplementary Fig. 127 | ^{1}H NMR spectrum of **8ba**

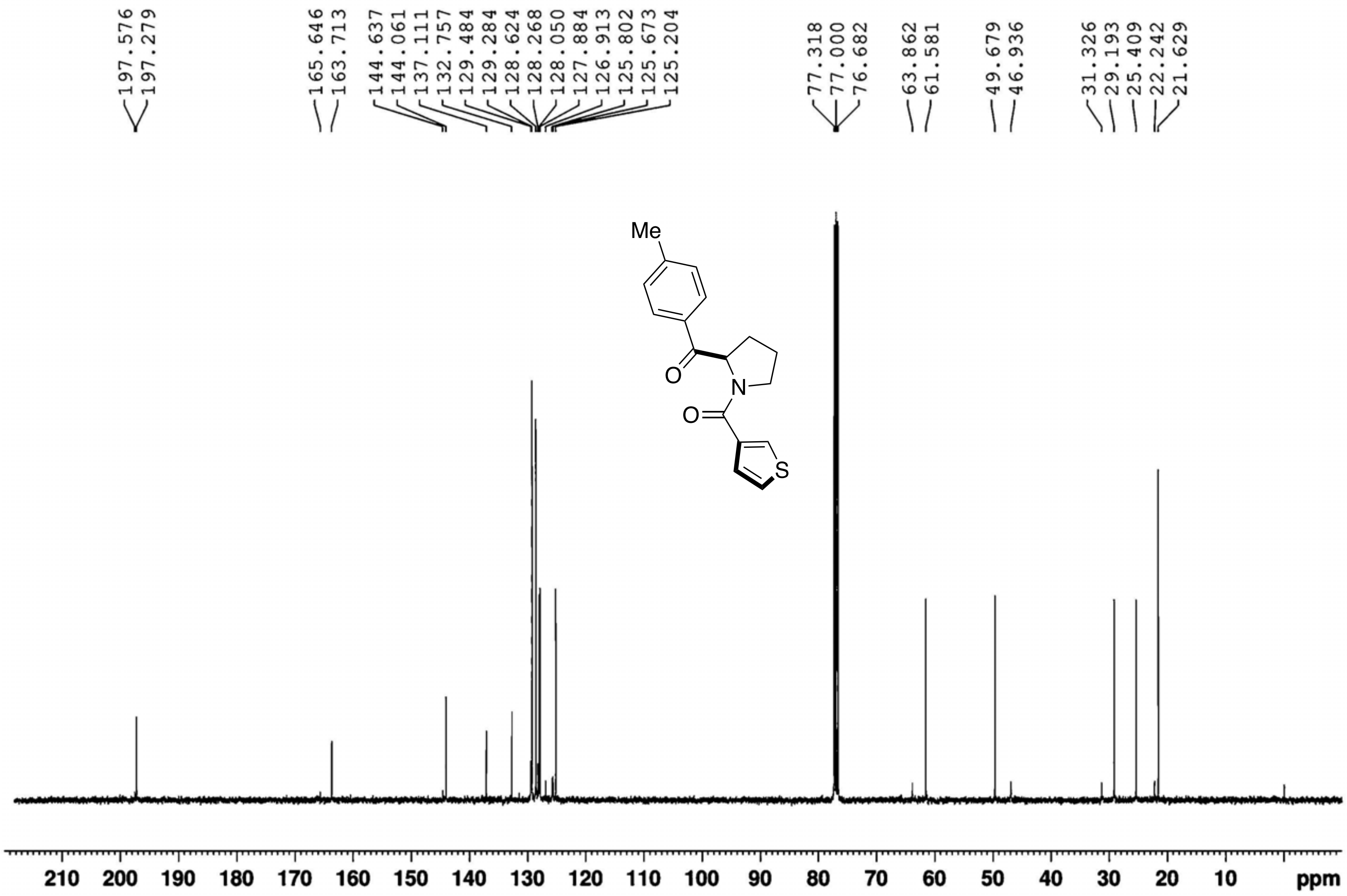

Supplementary Fig. 128 | ^{13}C NMR spectrum of **8ba**

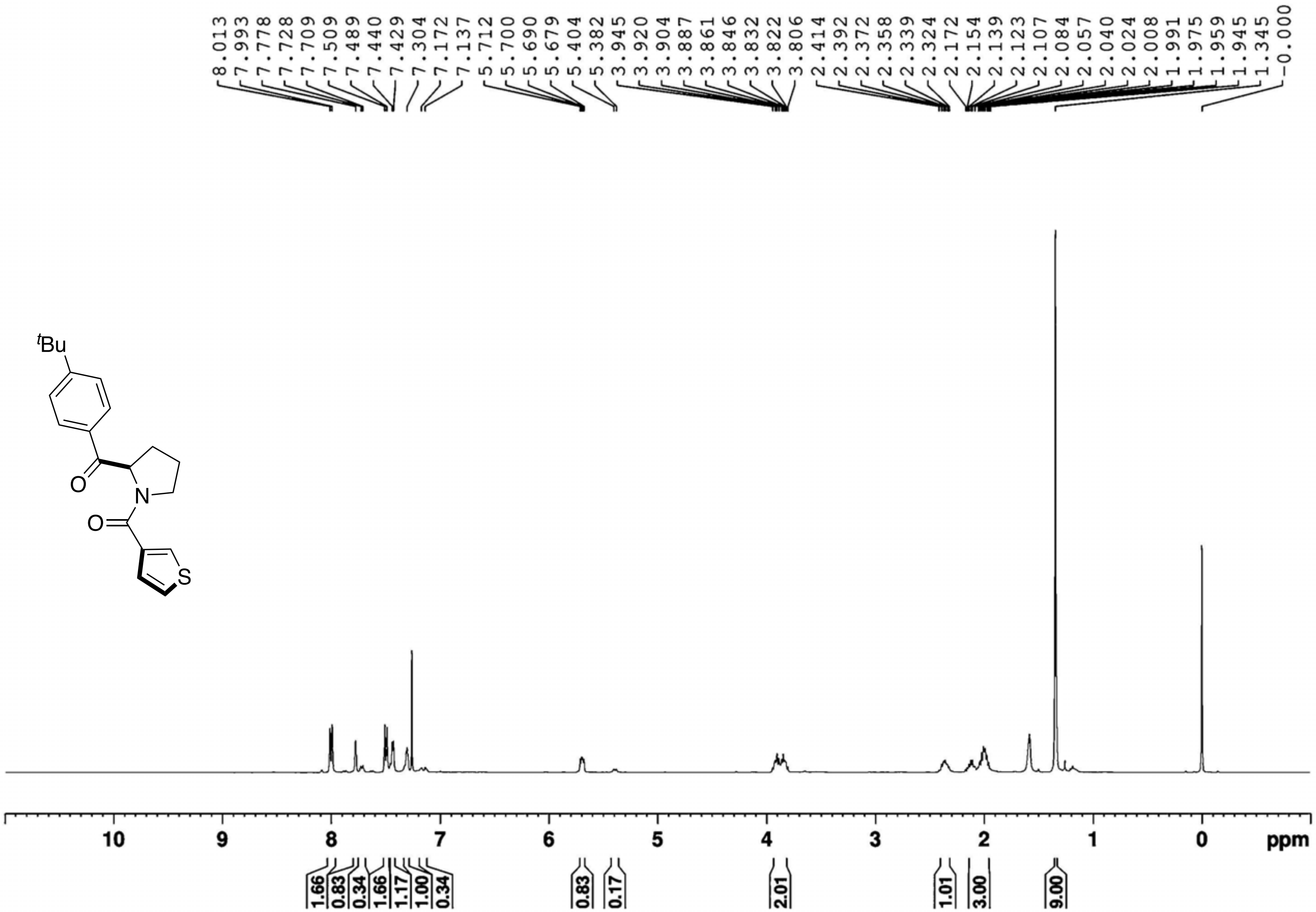

Supplementary Fig. 129 | ^{1}H NMR spectrum of **8ca**

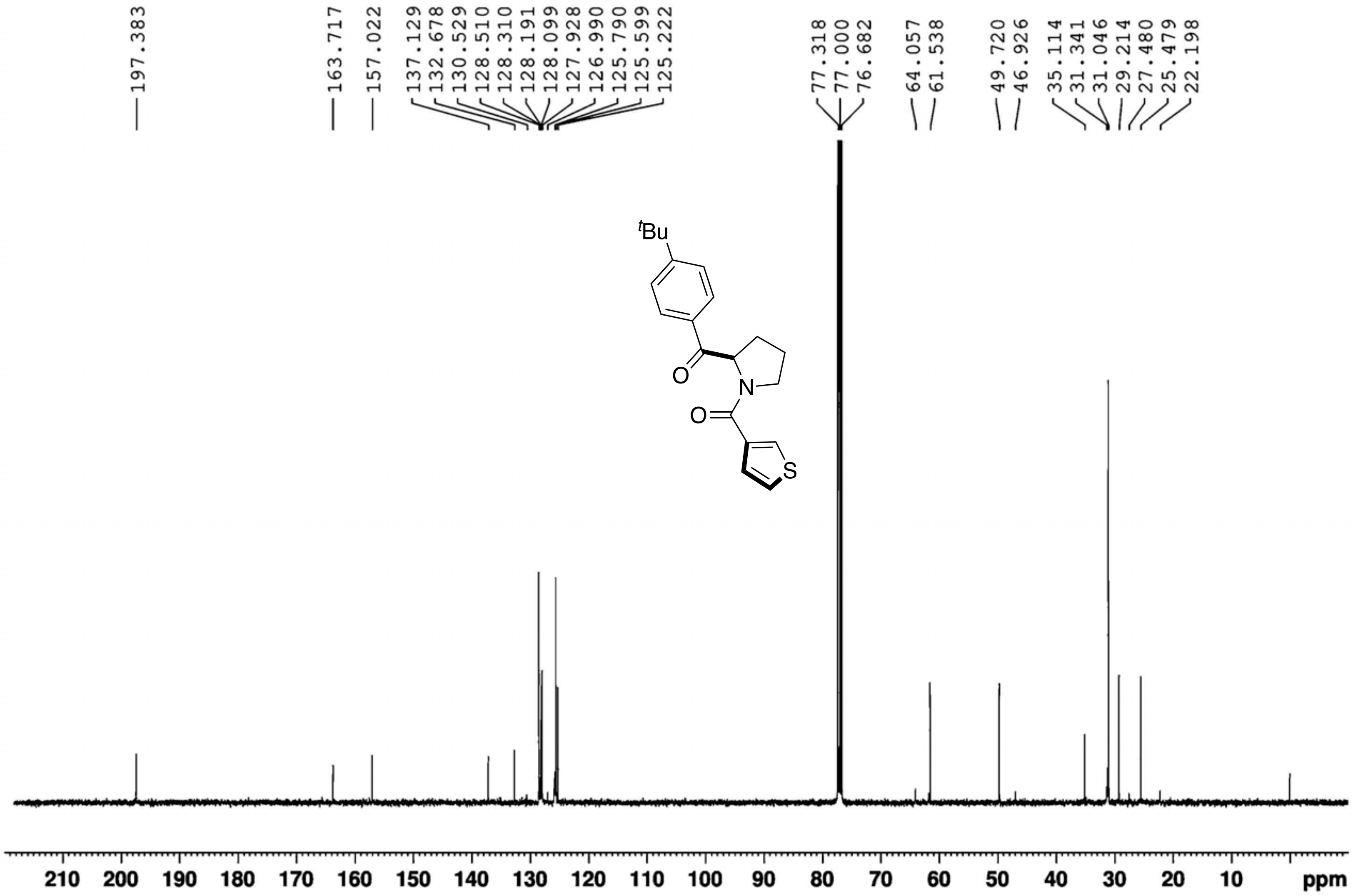

Supplementary Fig. 130 | ^{13}C NMR spectrum of **8ca**

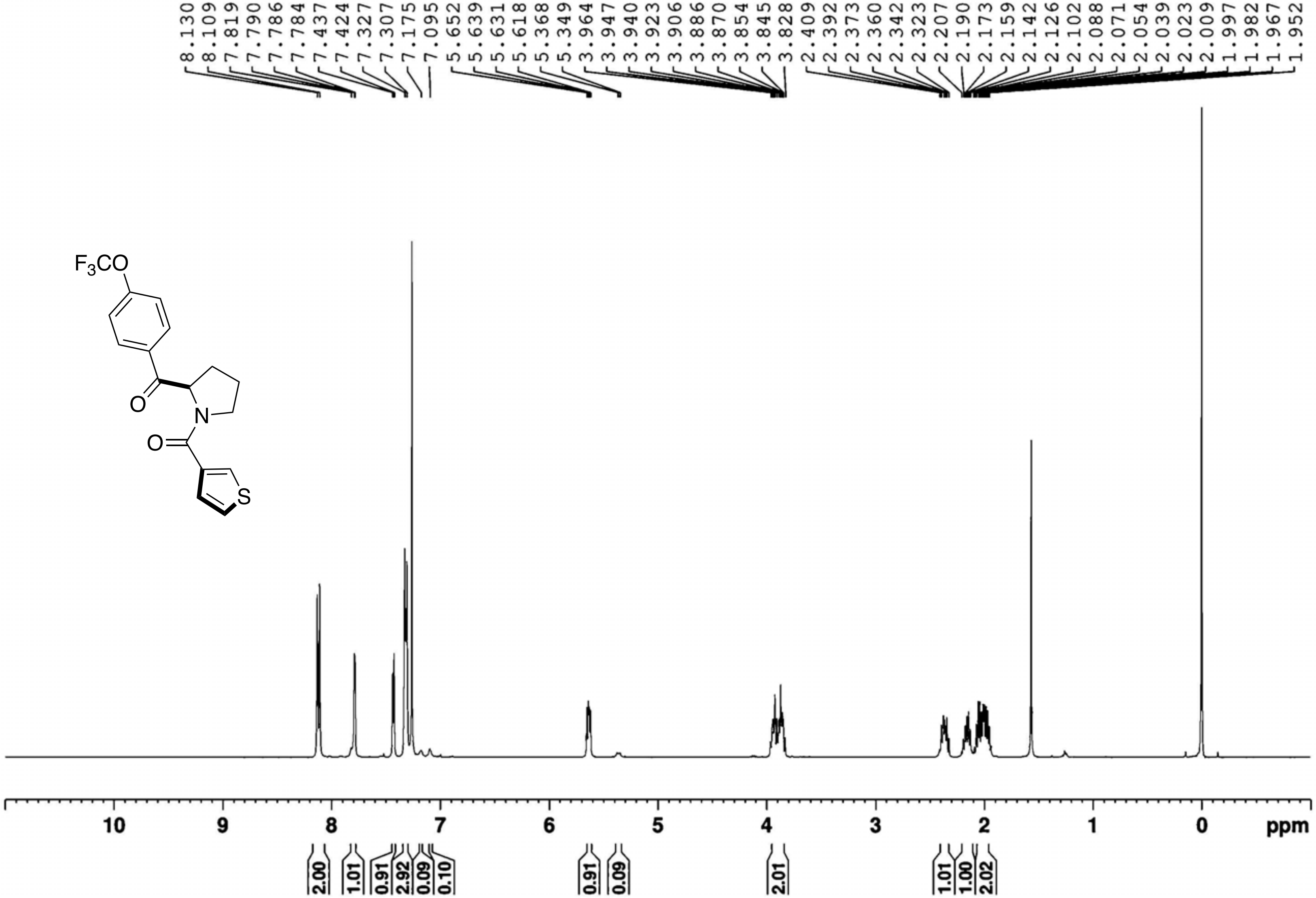

Supplementary Fig. 131 | ^{1}H NMR spectrum of **8fa**

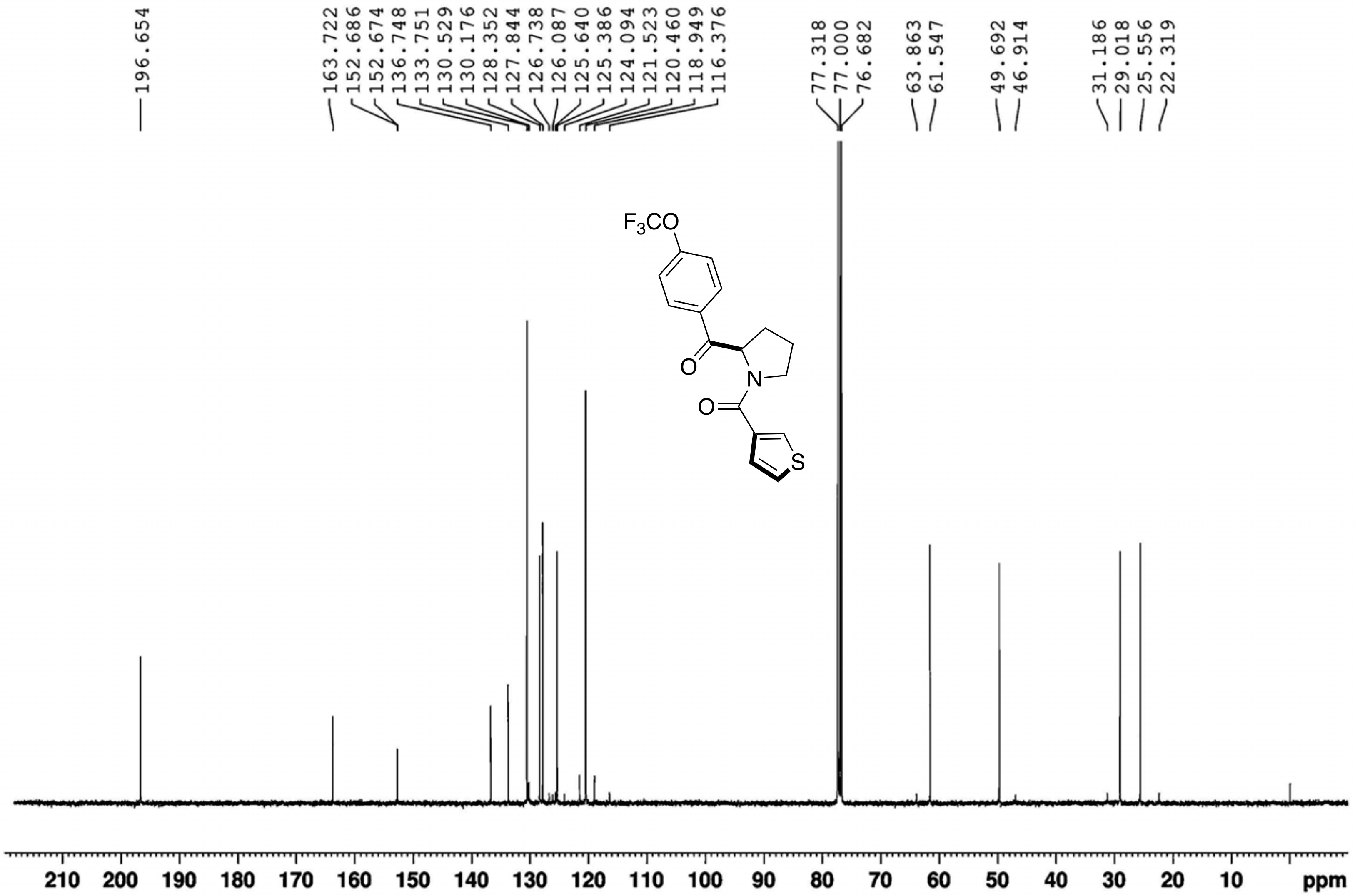

Supplementary Fig. 132 | ^{13}C NMR spectrum of **8fa**

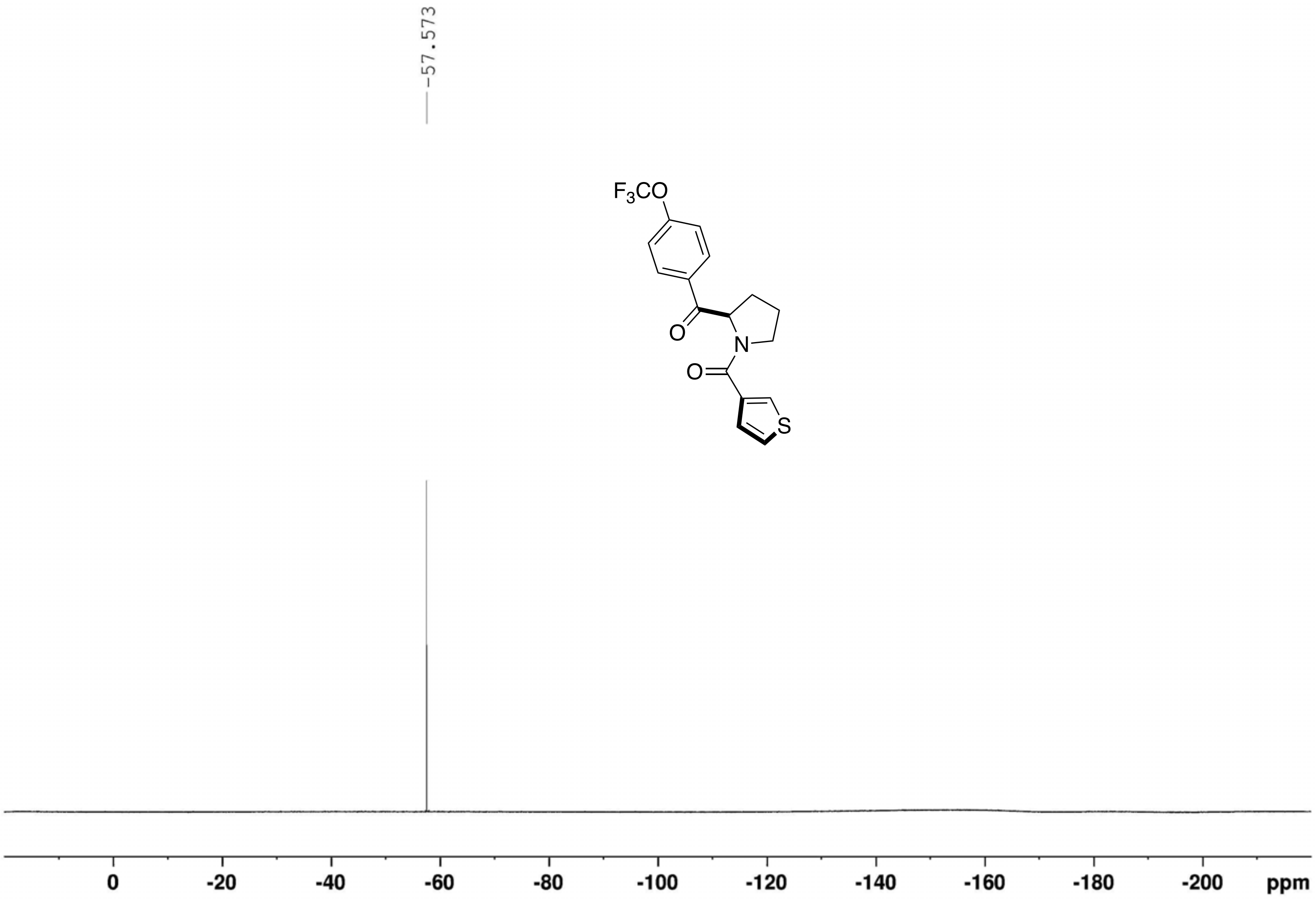

Supplementary Fig. 133 | ^{19}F NMR spectrum of **8fa**

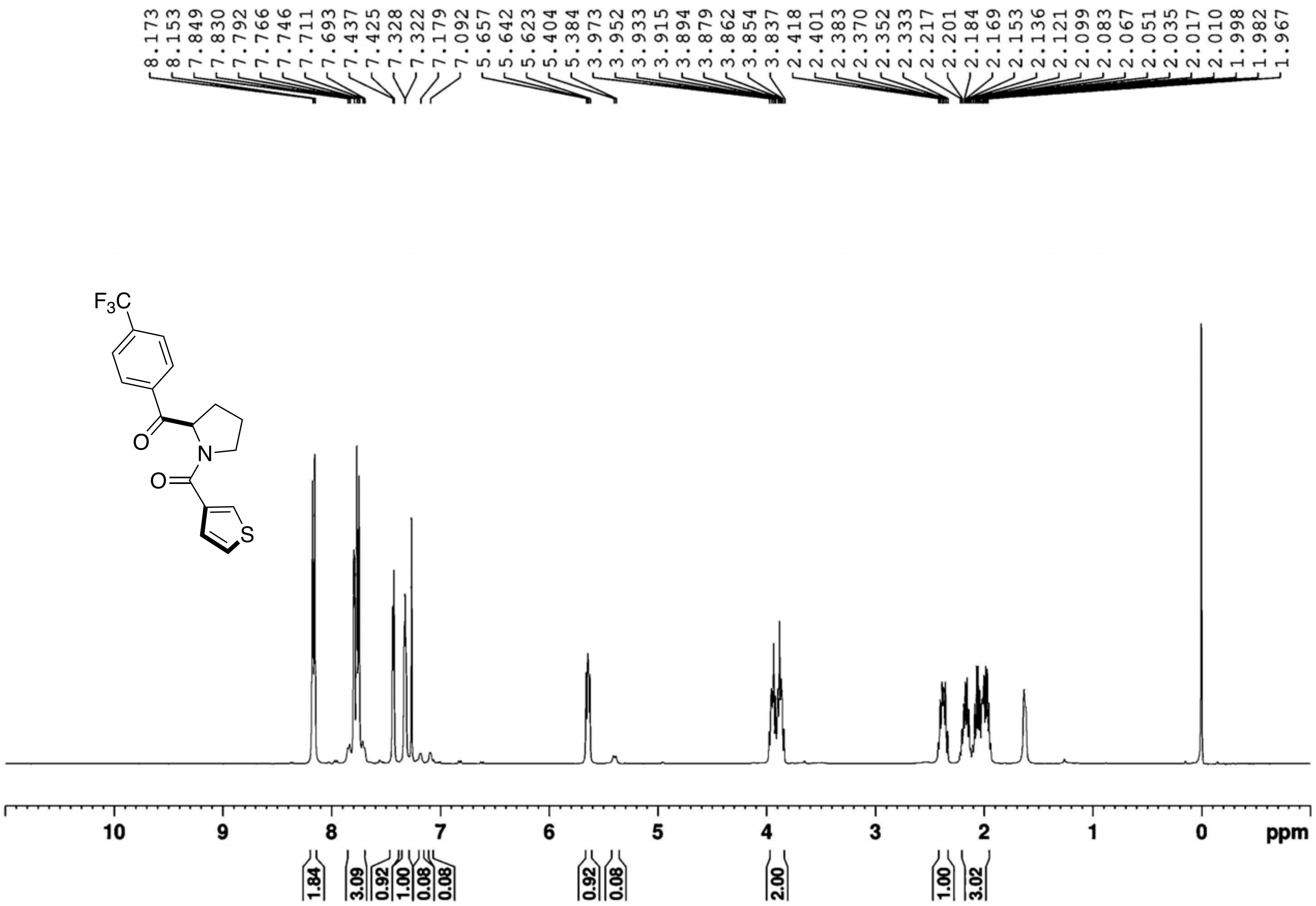

Supplementary Fig. 134 | ^{1}H NMR spectrum of **8ga**

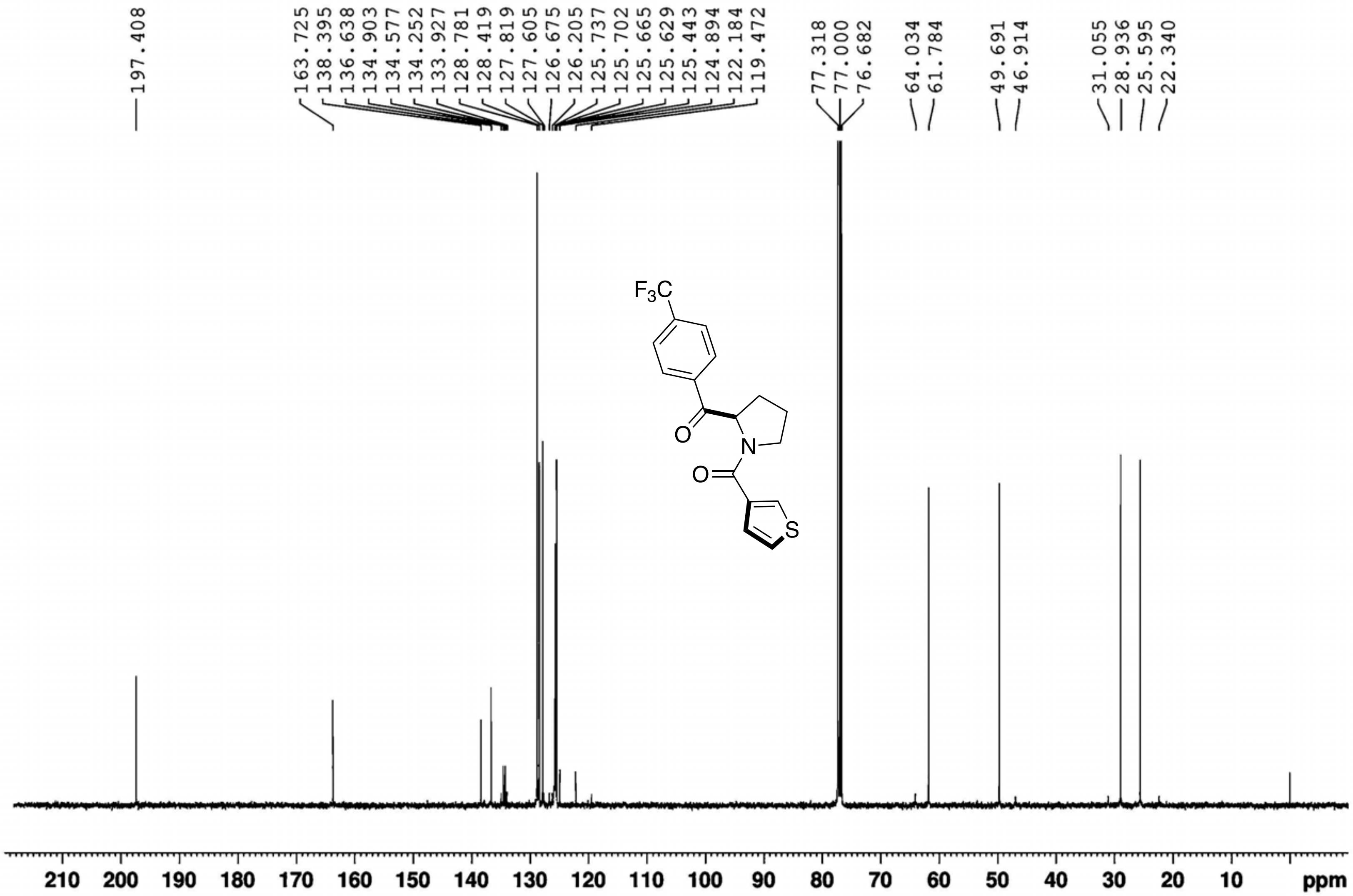

Supplementary Fig. 135 | ^{13}C NMR spectrum of **8ga**

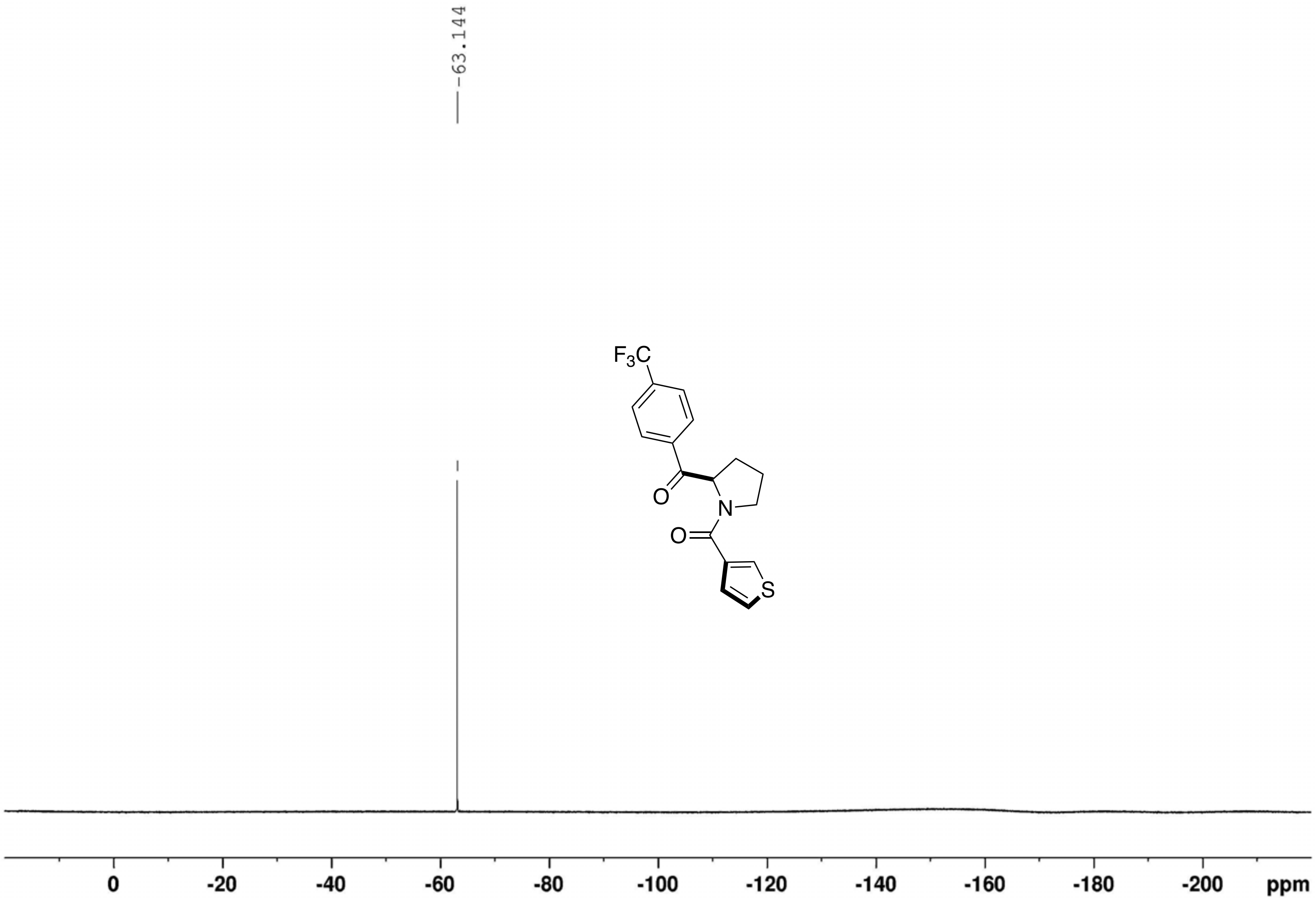

Supplementary Fig. 136 | ^{19}F NMR spectrum of **8ga**

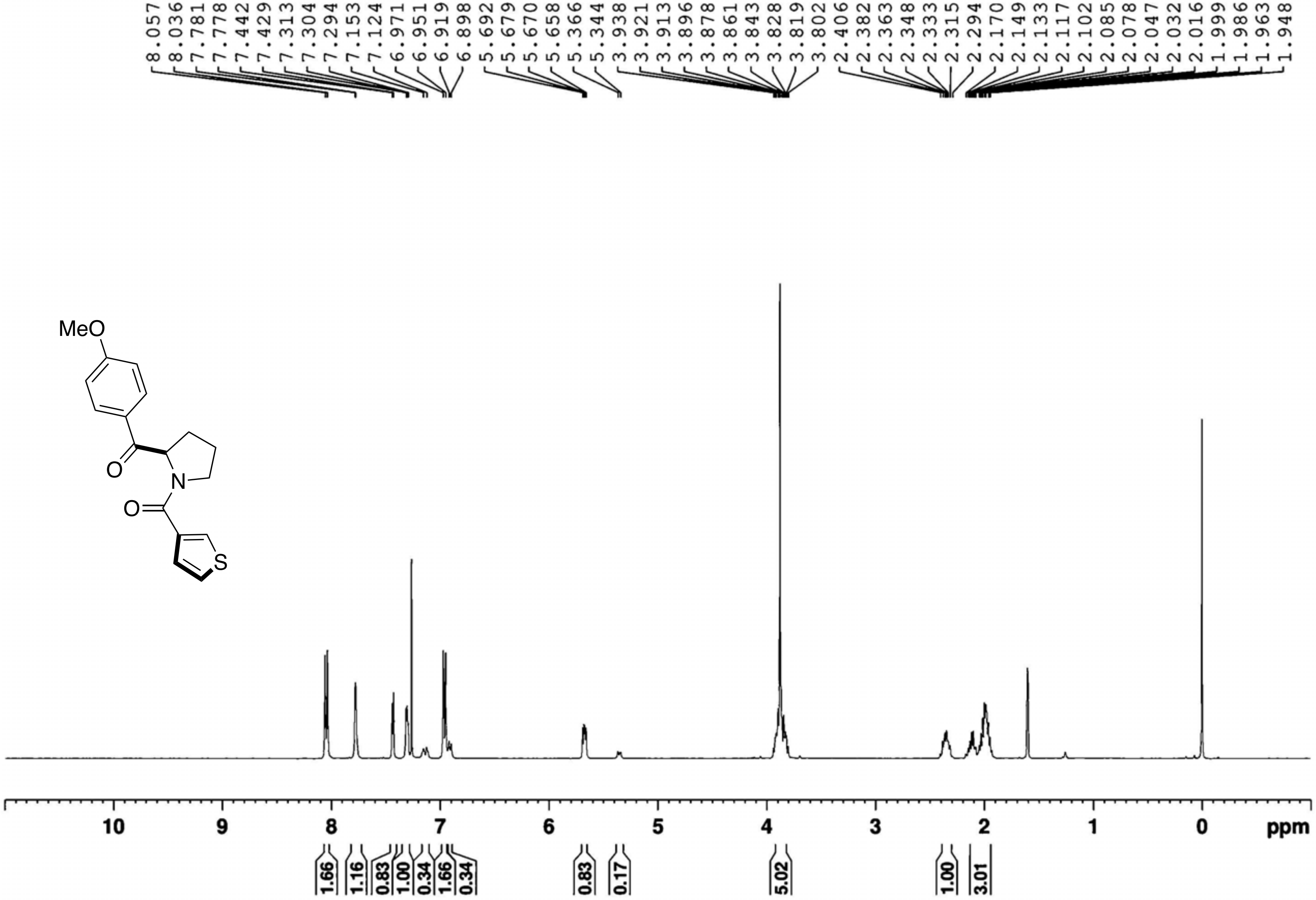

Supplementary Fig. 137 | ^{1}H NMR spectrum of **8ha**

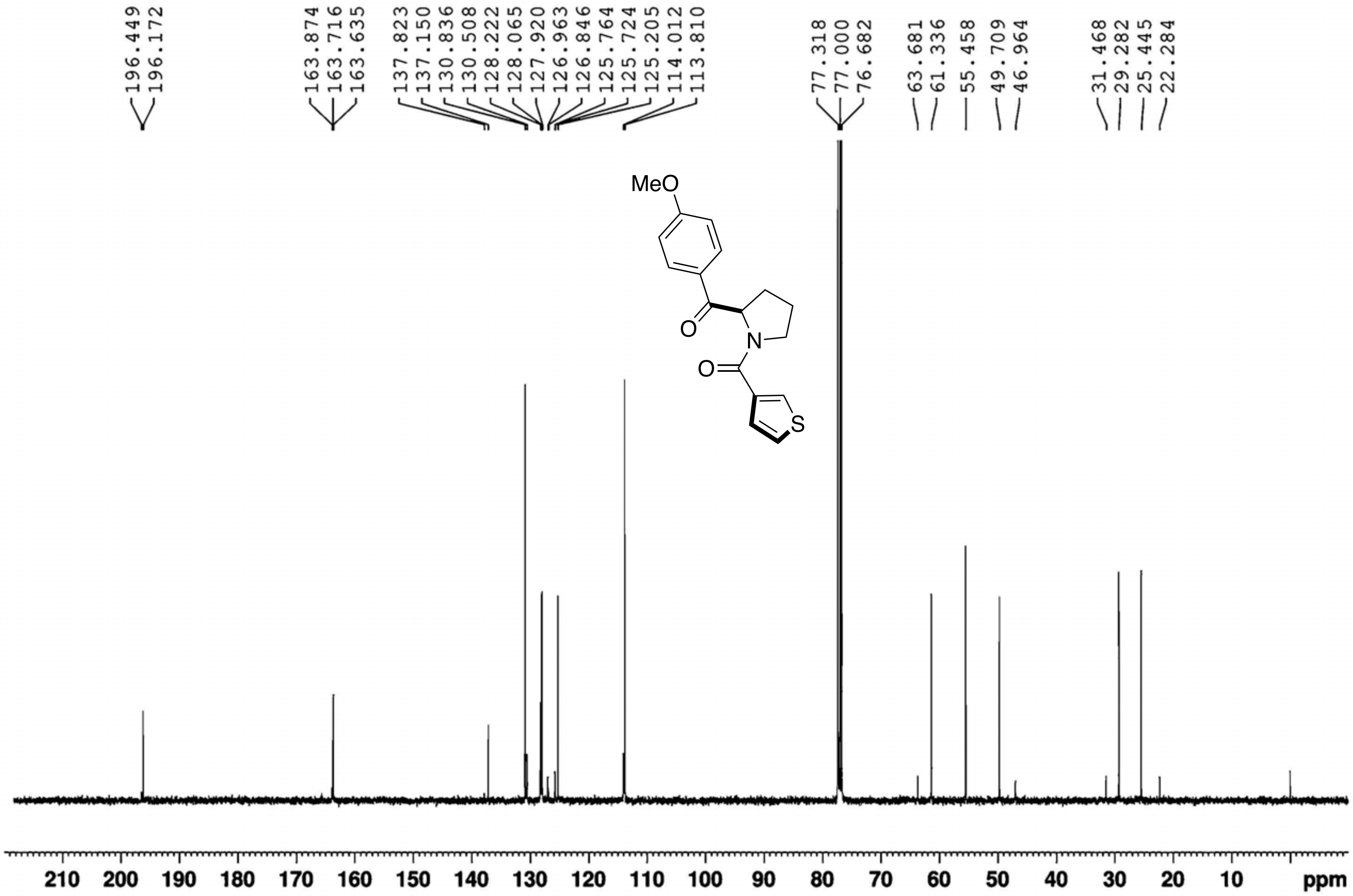

Supplementary Fig. 138 | ^{13}C NMR spectrum of **8ha**

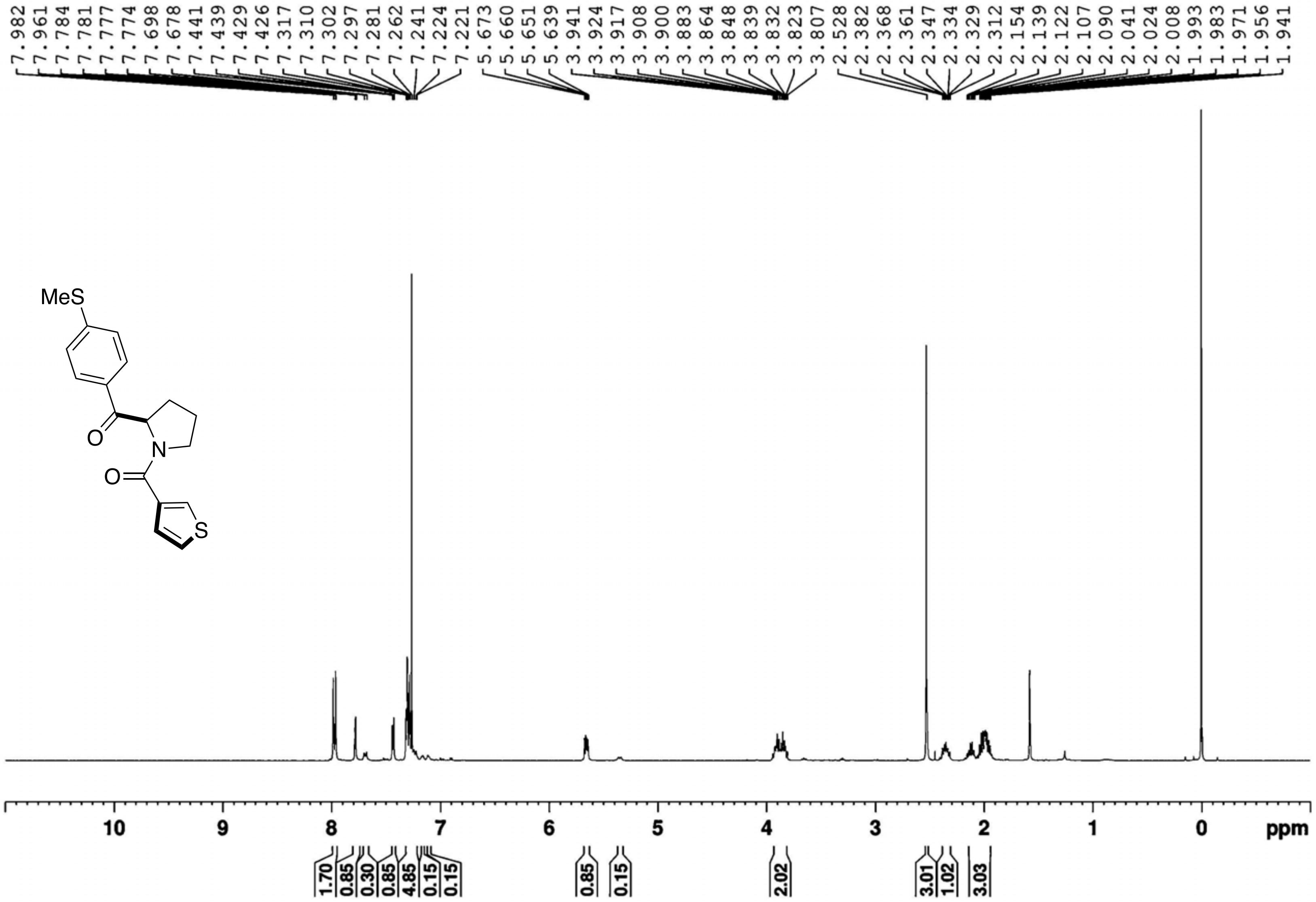

Supplementary Fig. 139 | ^{1}H NMR spectrum of **8ia**

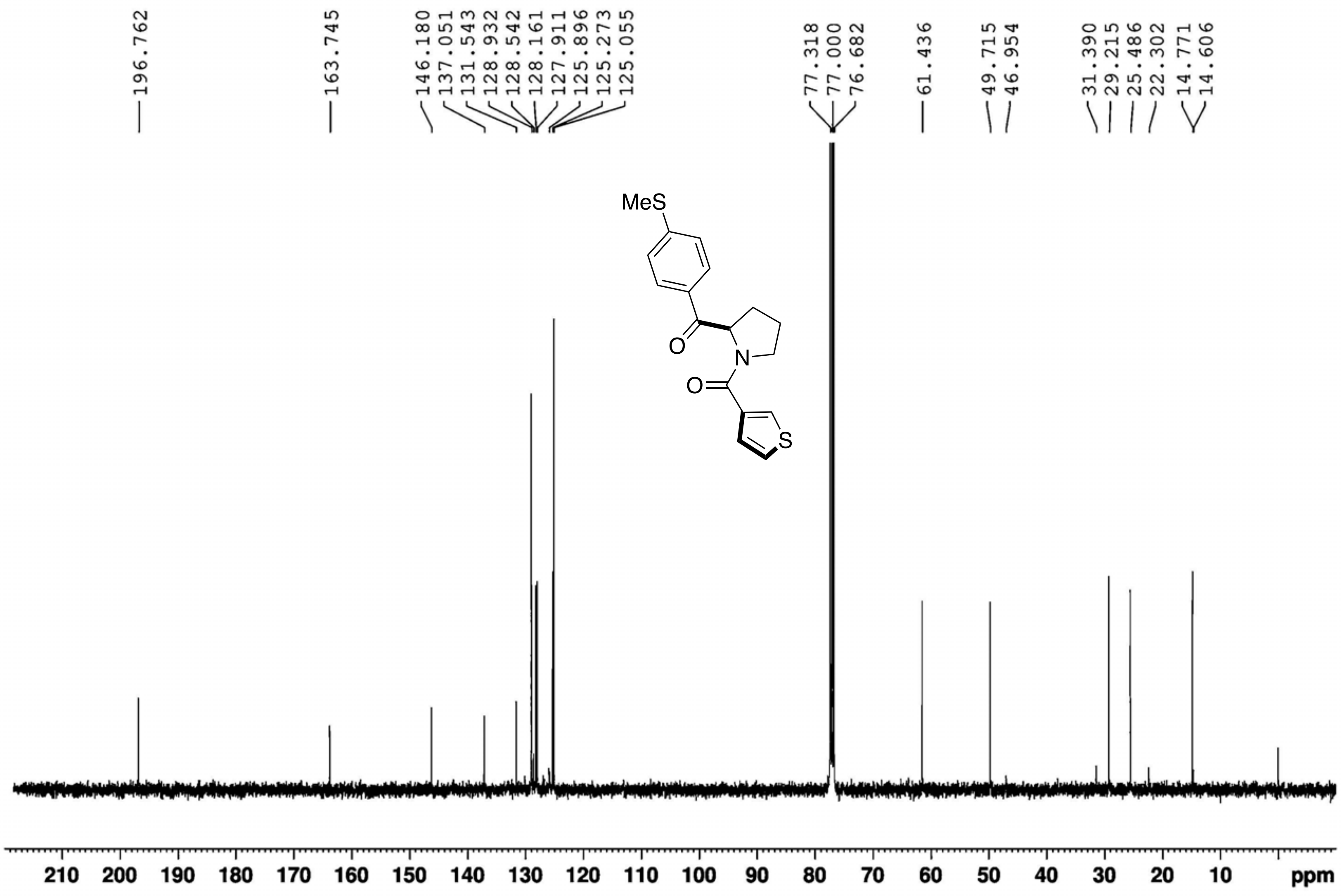

Supplementary Fig. 140 | ^{13}C NMR spectrum of **8ia**

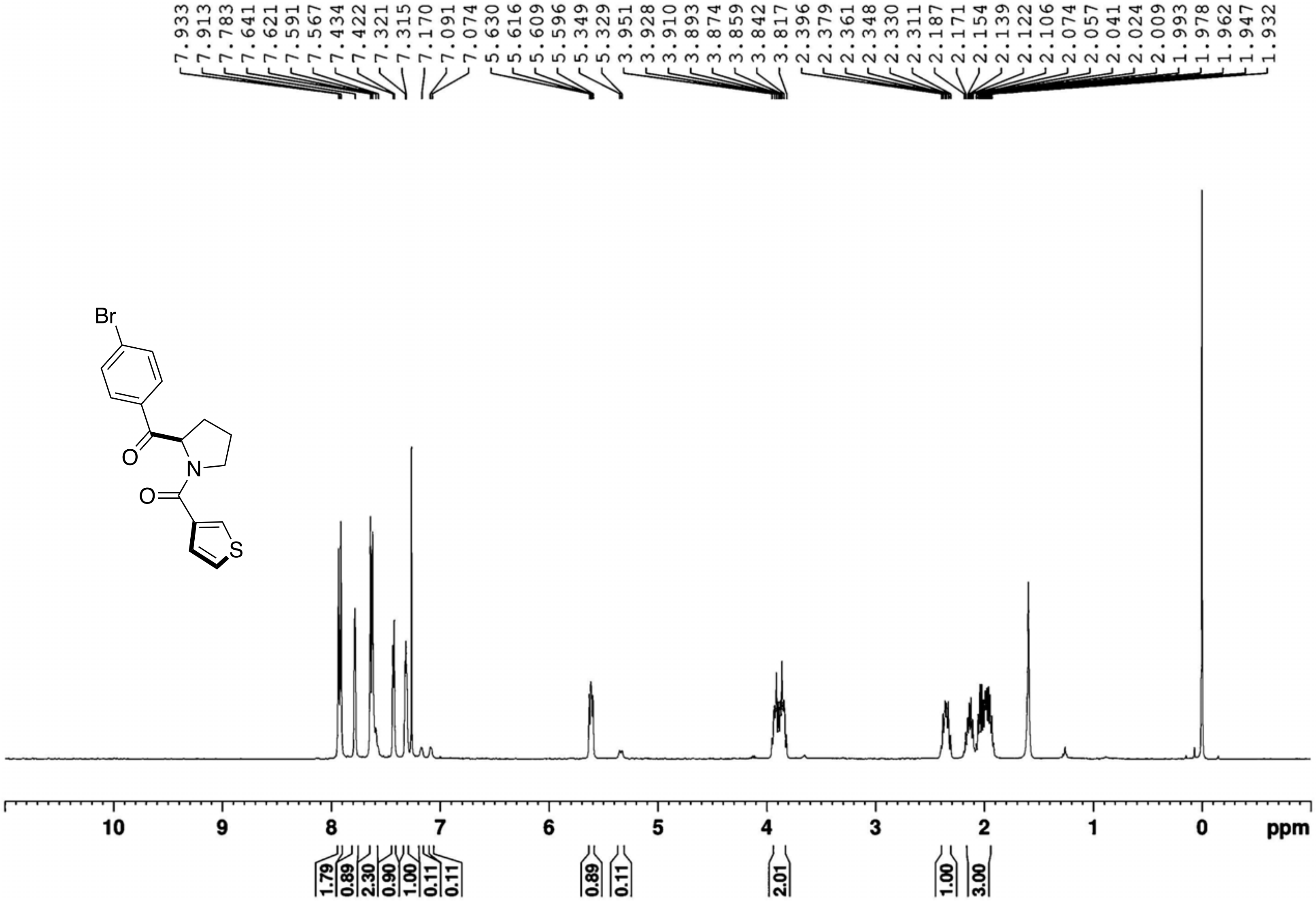

Supplementary Fig. 141 | ^{1}H NMR spectrum of **8ea**

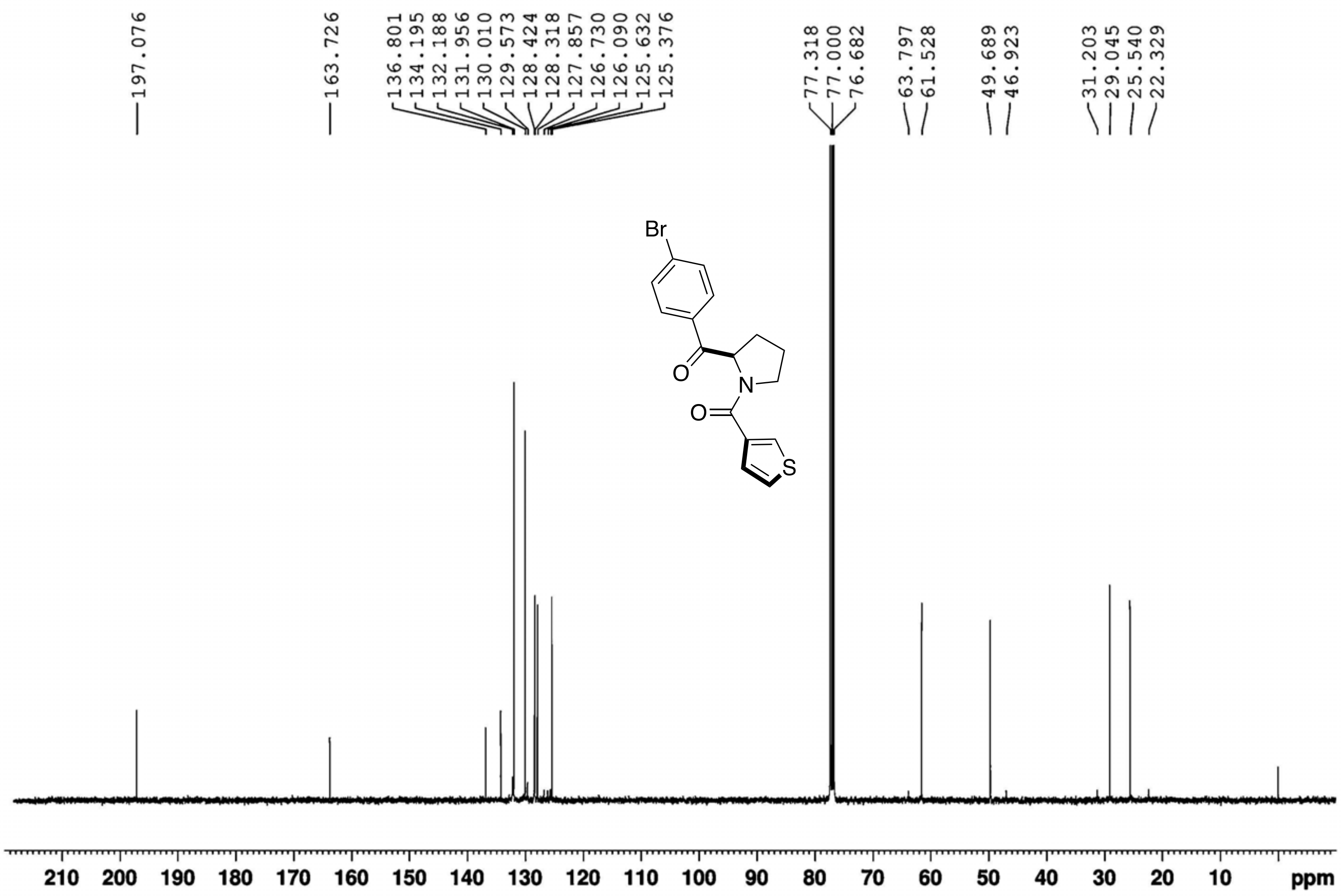

Supplementary Fig. 142 | ^{13}C NMR spectrum of **8ea**

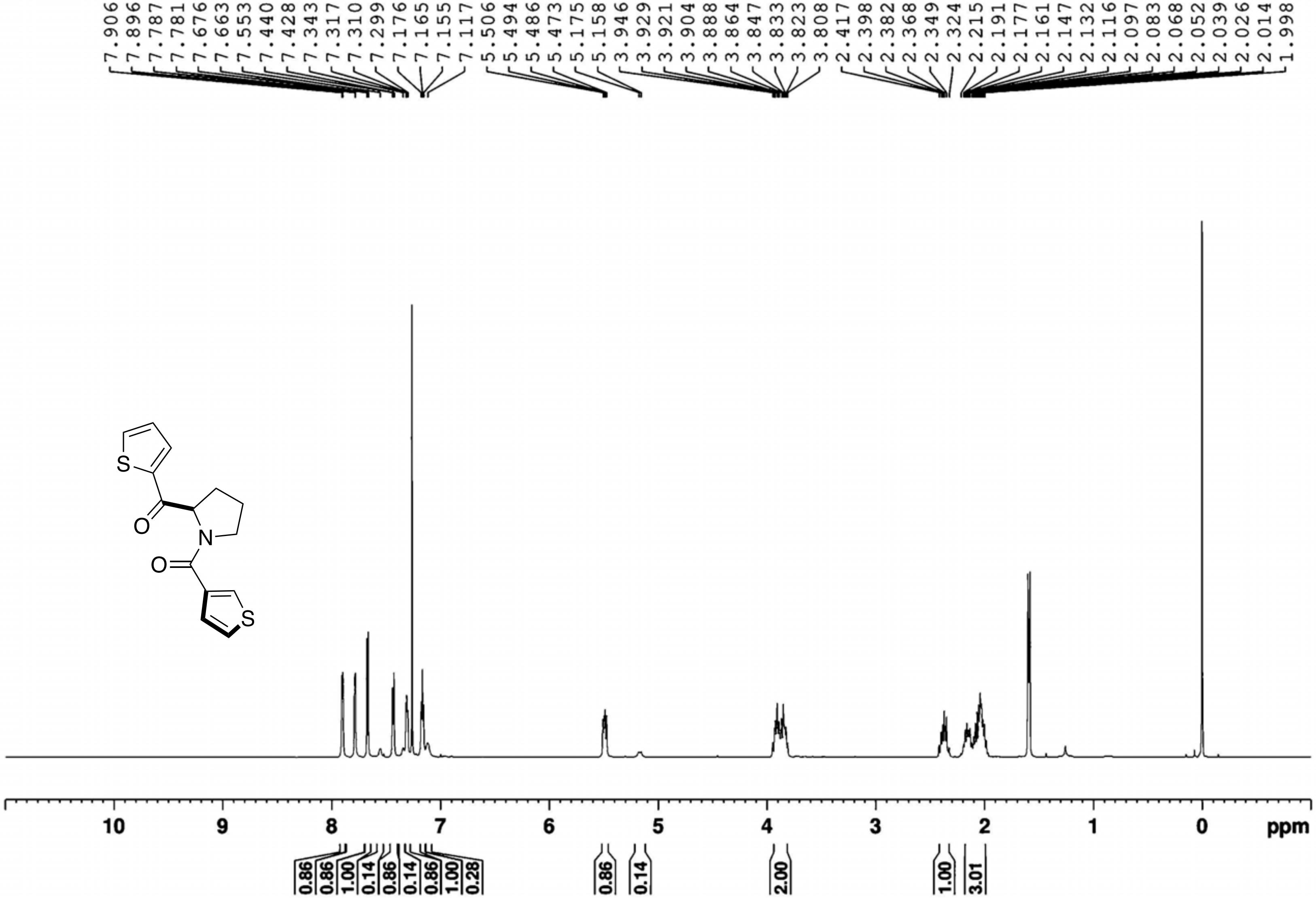

Supplementary Fig. 143 | ^{1}H NMR spectrum of **8ja**

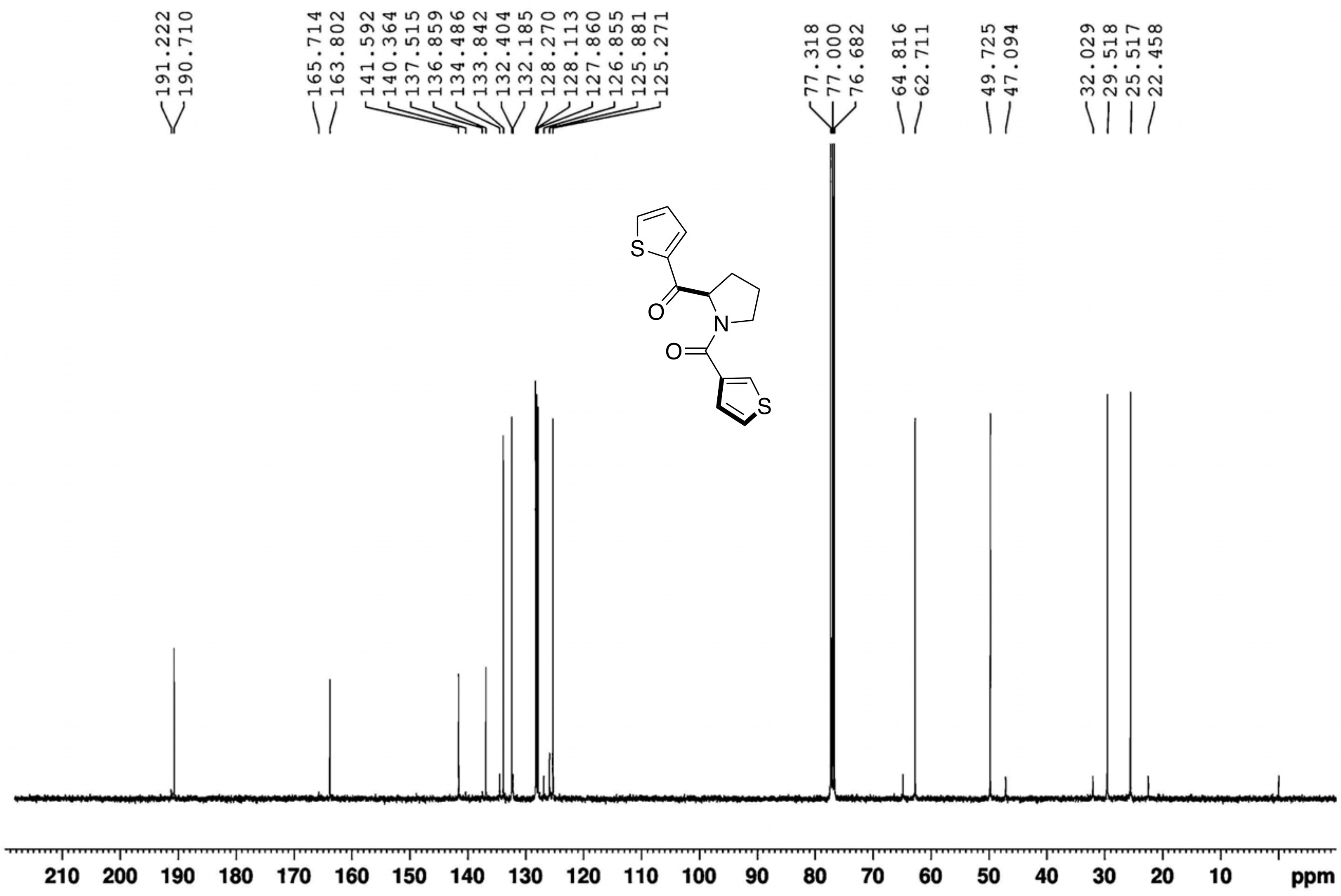

Supplementary Fig. 144 | ^{13}C NMR spectrum of **8ja**

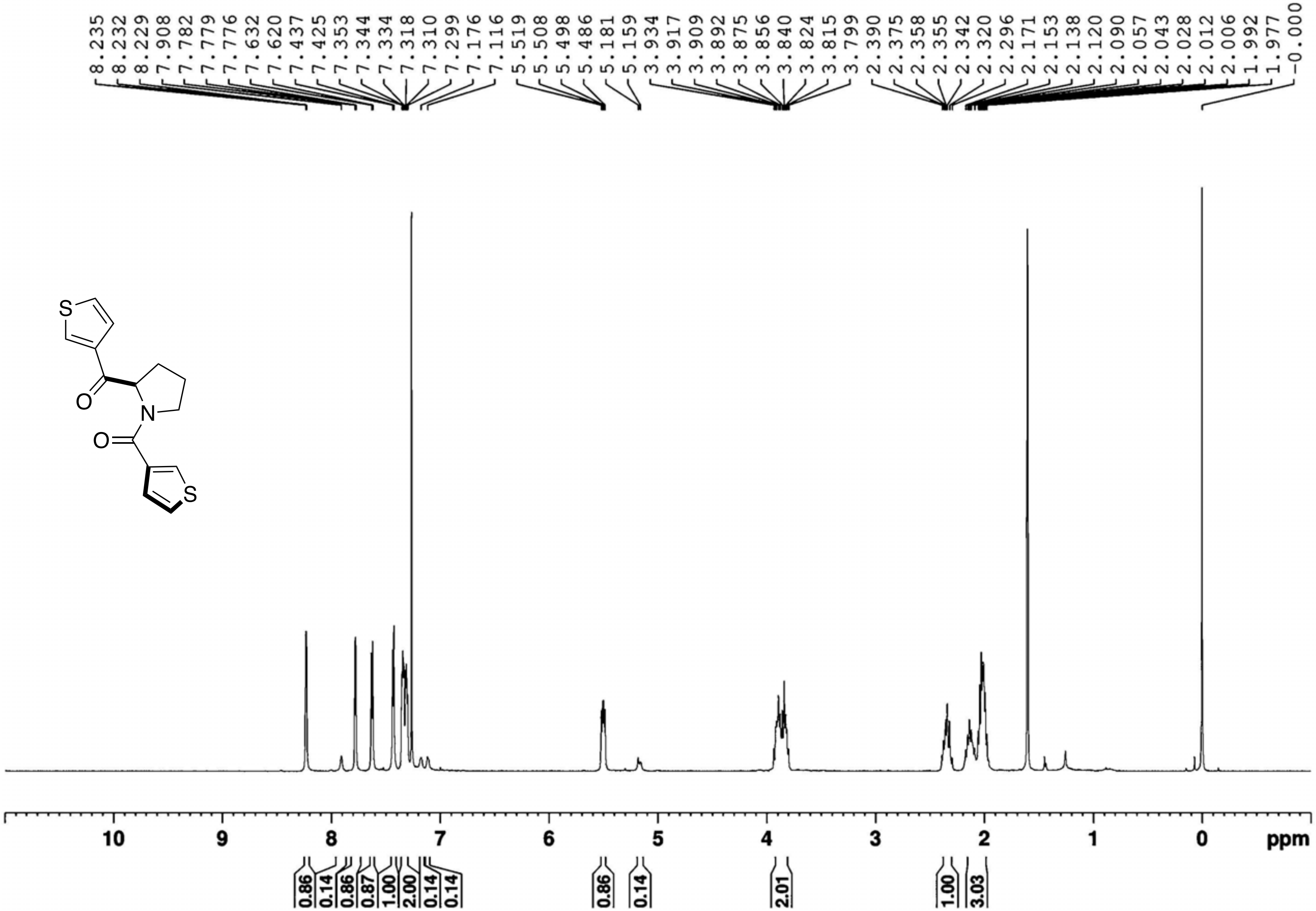

Supplementary Fig. 145 | ^{1}H NMR spectrum of **8na**

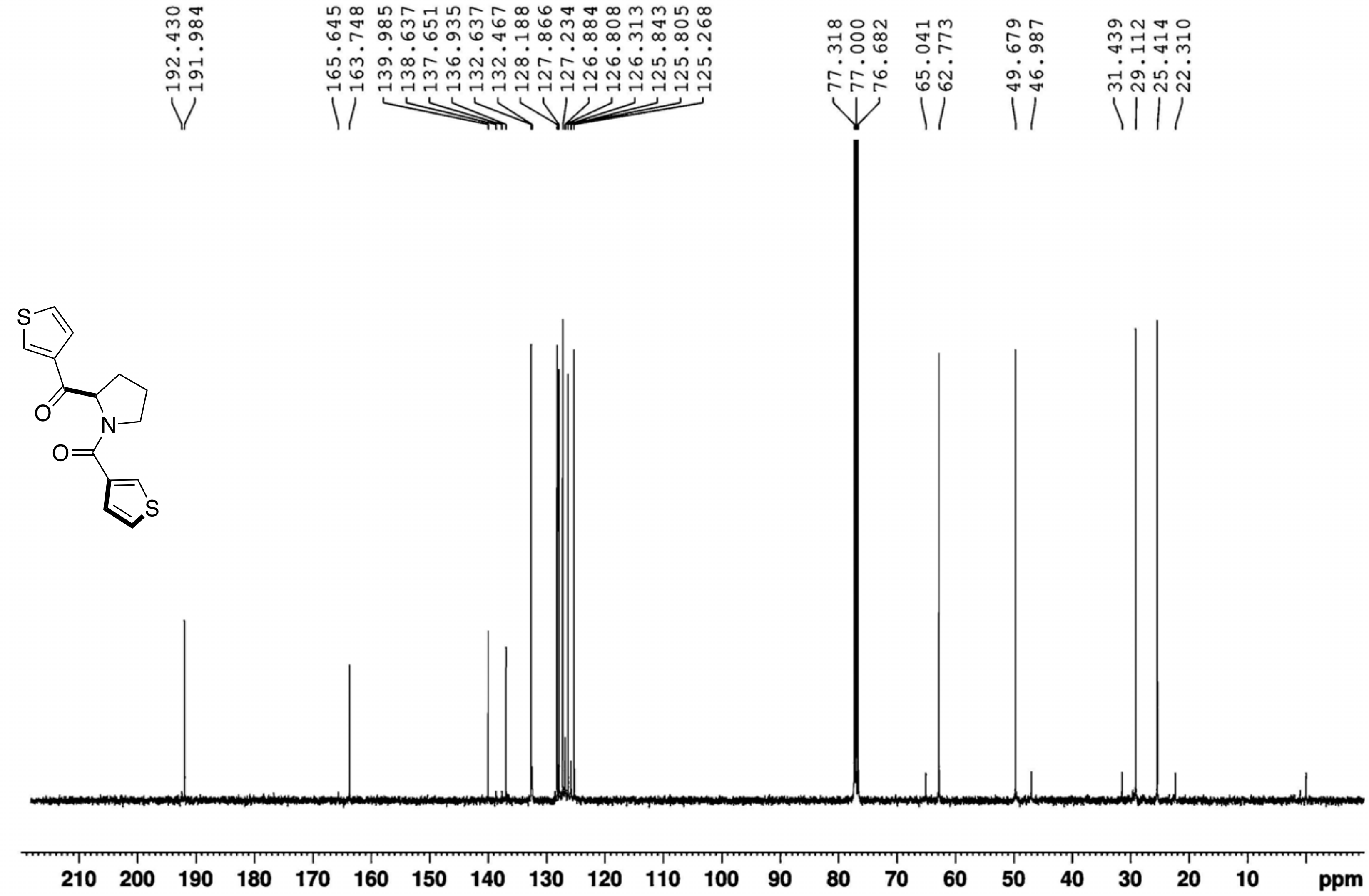

Supplementary Fig. 146 | ^{13}C NMR spectrum of **8na**

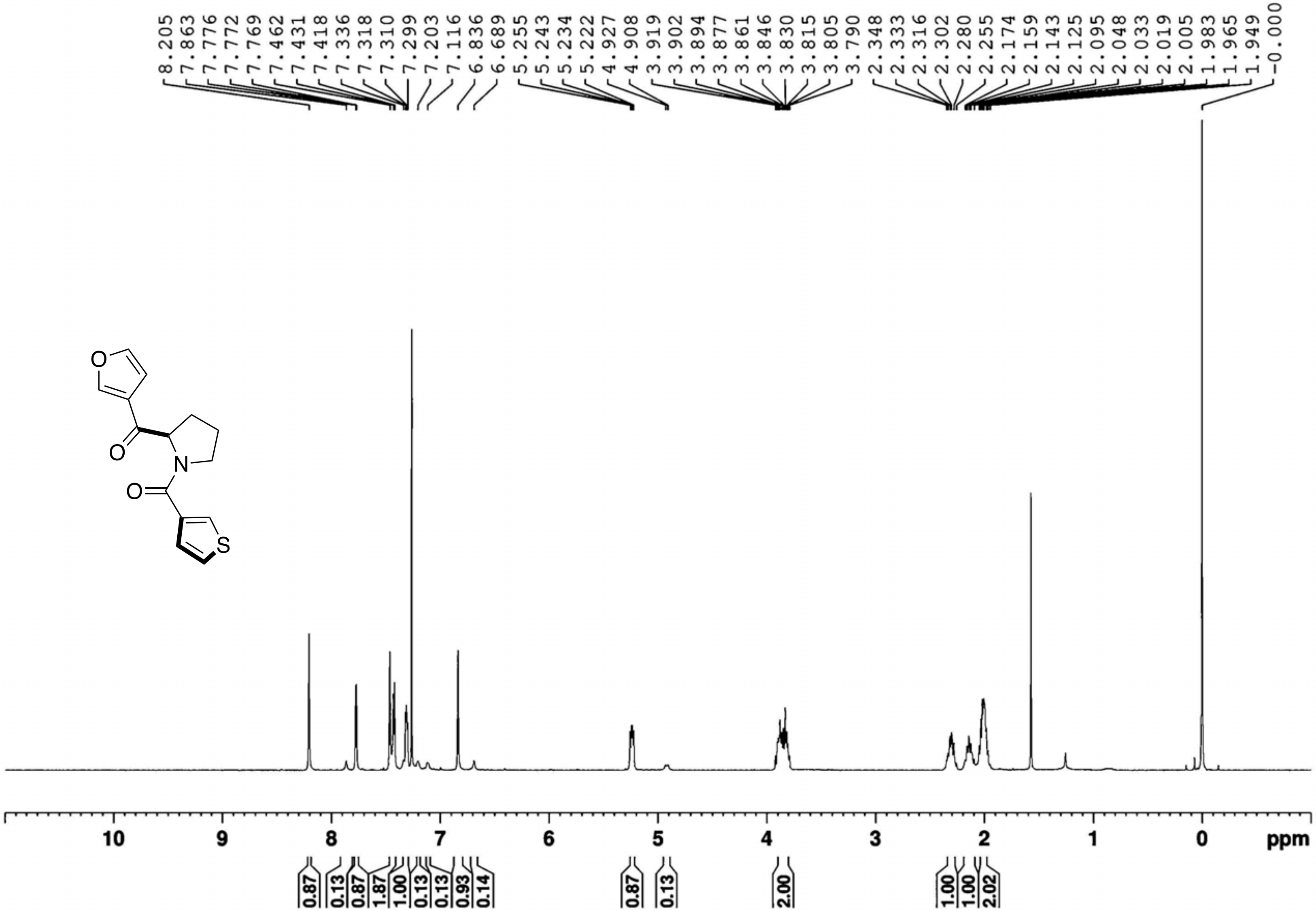

Supplementary Fig. 147 | ^{1}H NMR spectrum of **8oa**

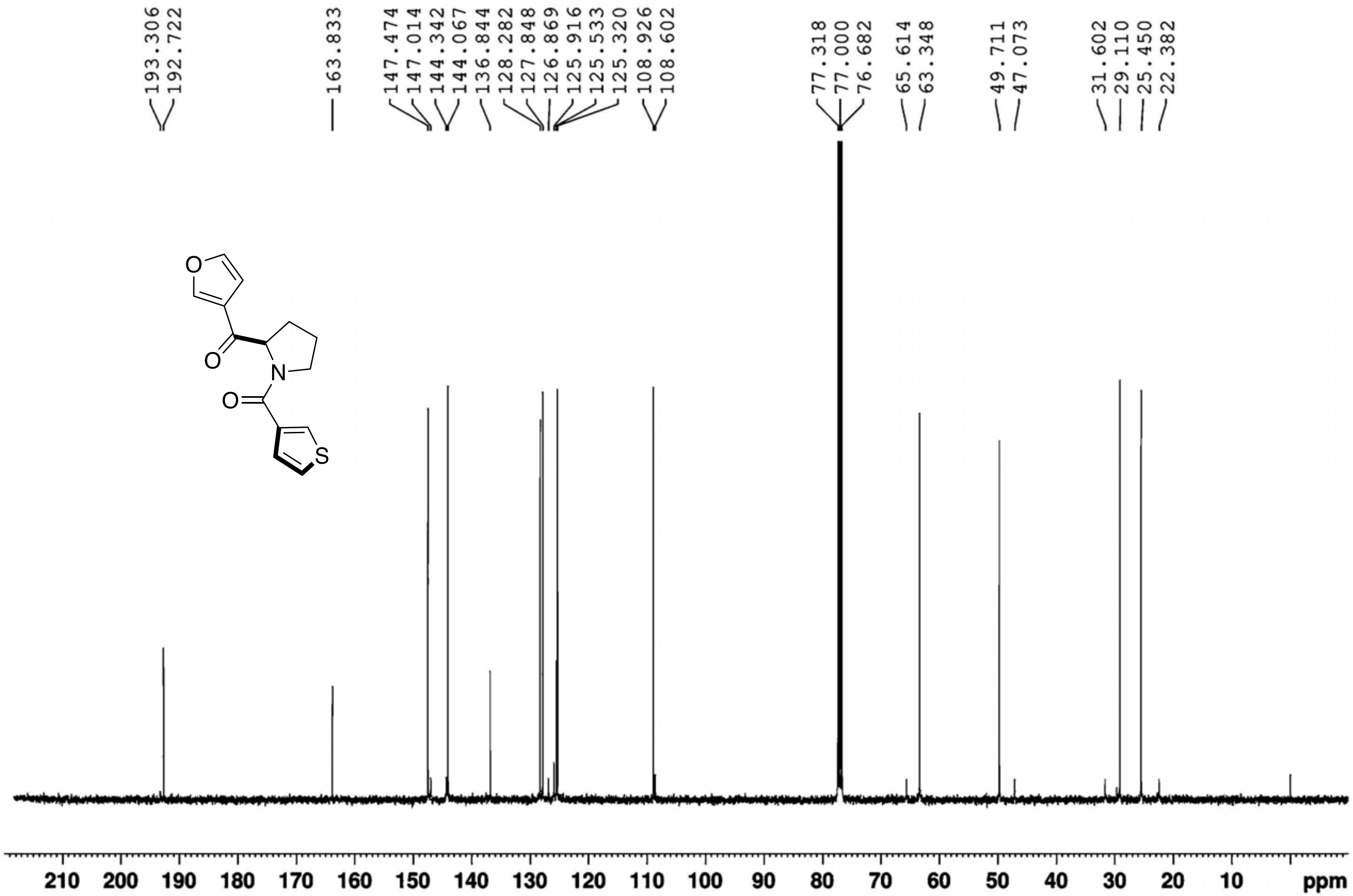

Supplementary Fig. 148 | ^{13}C NMR spectrum of **8oa**

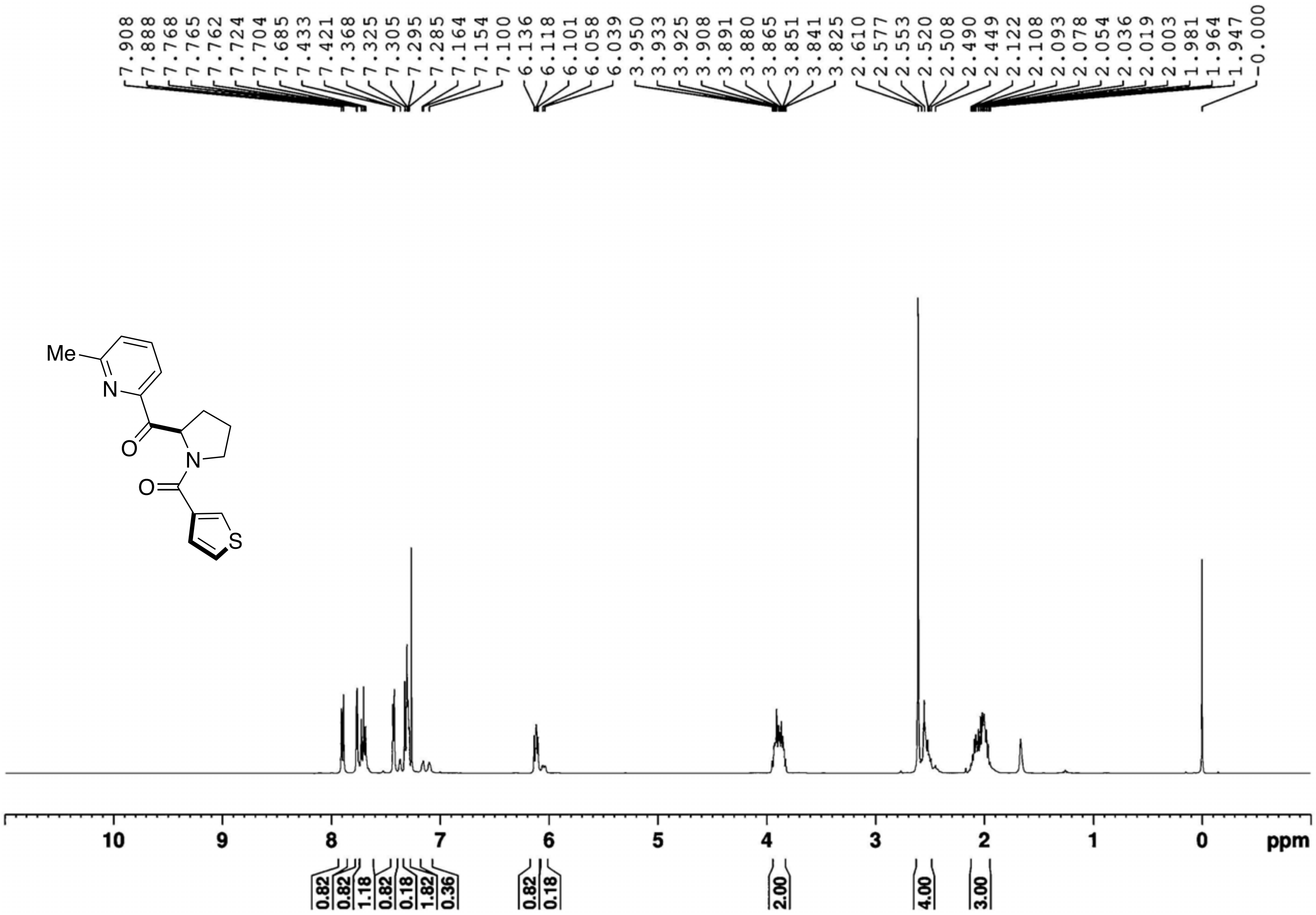

Supplementary Fig. 149 | ¹H NMR spectrum of **8pa**

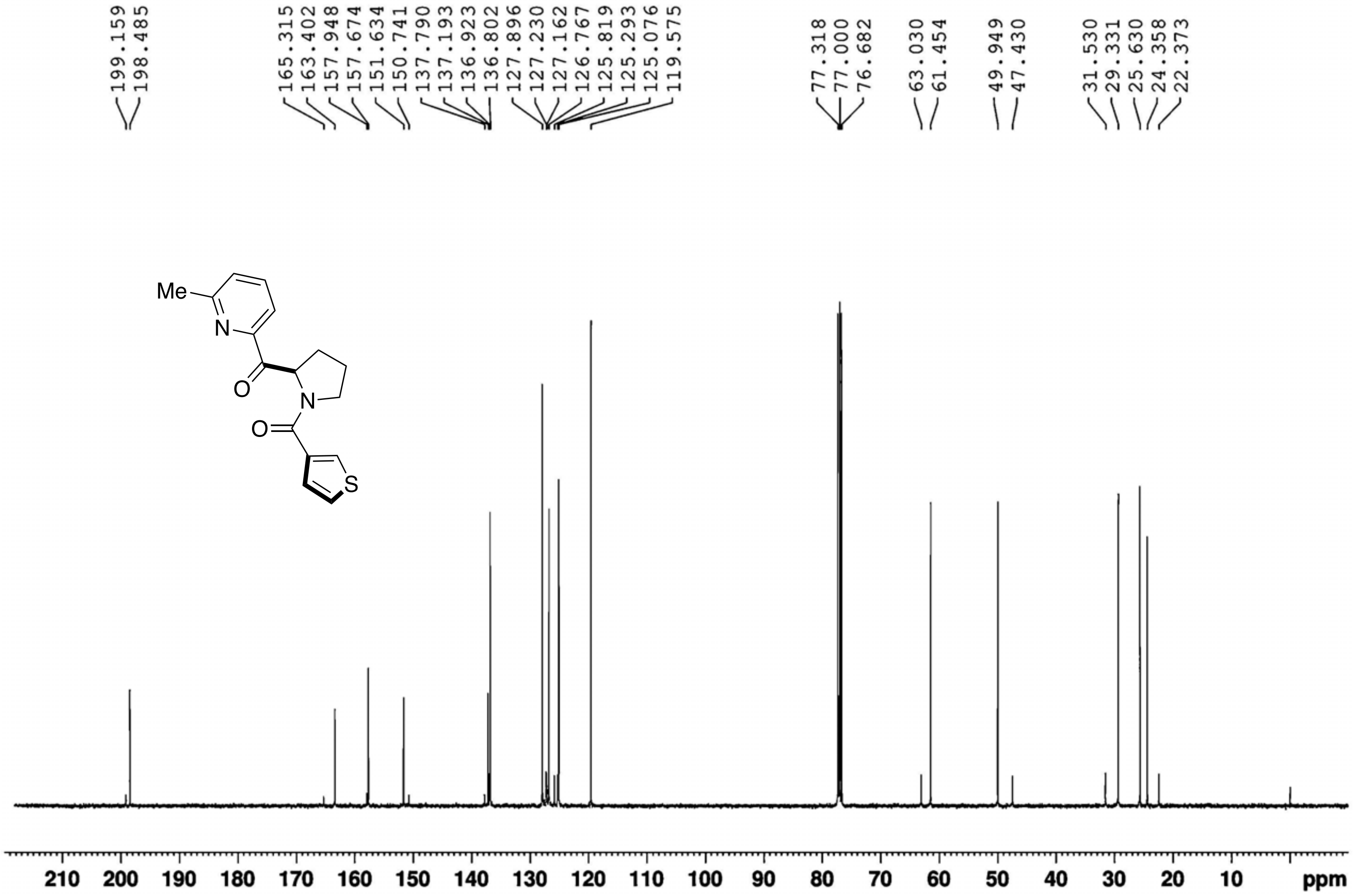

Supplementary Fig. 150 | ^{13}C NMR spectrum of **8pa**

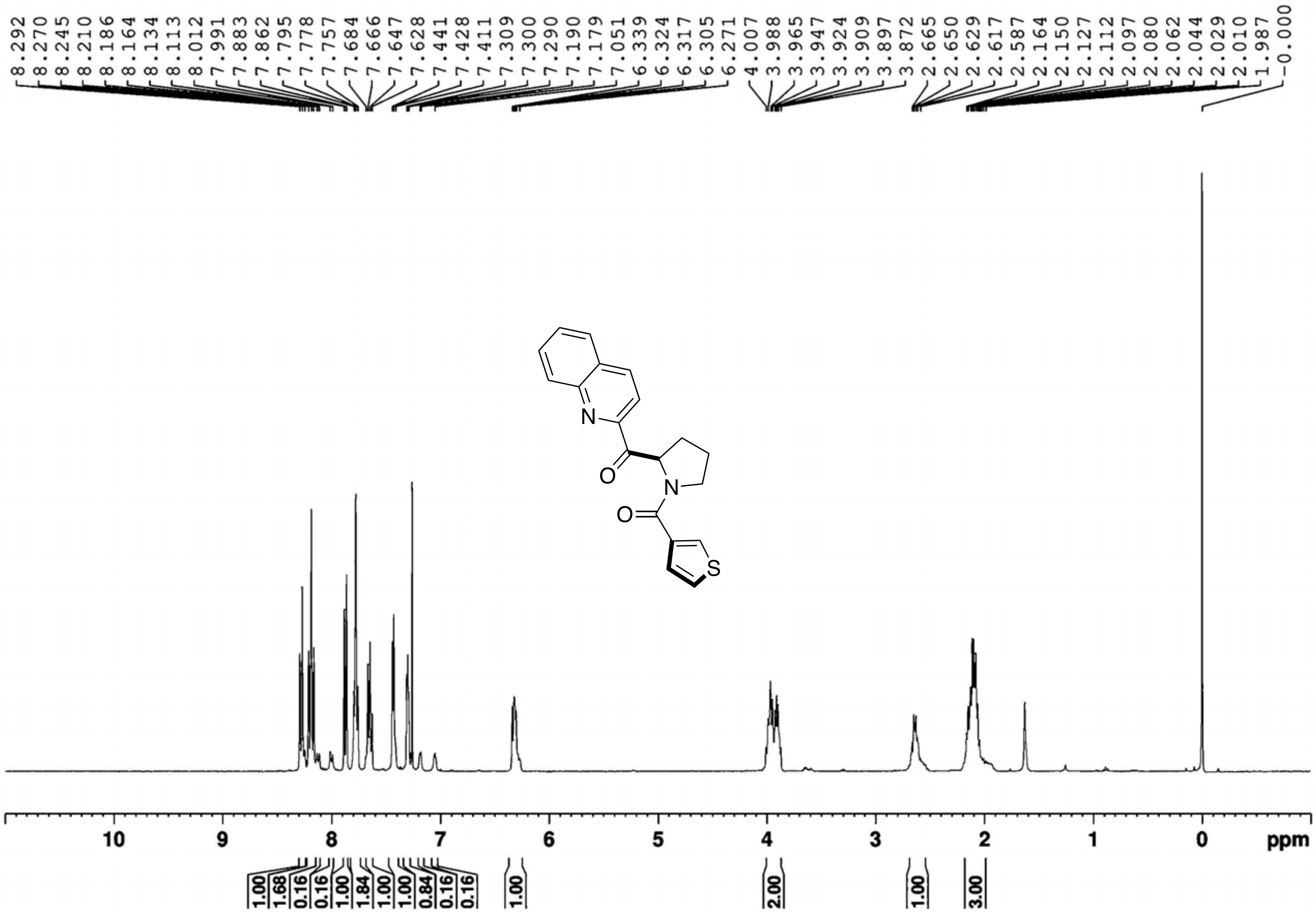

Supplementary Fig. 151 | ^{1}H NMR spectrum of **8qa**

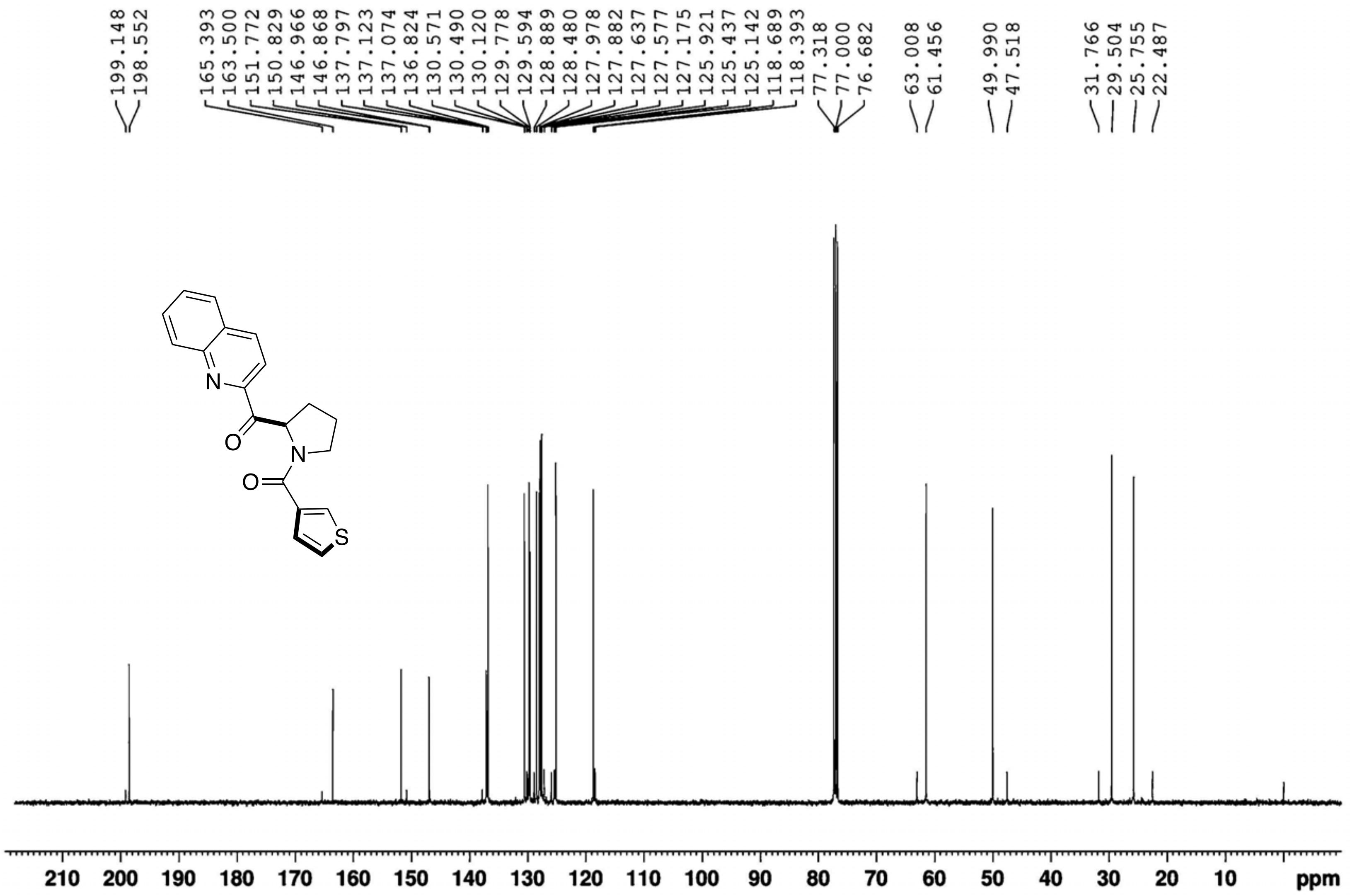

Supplementary Fig. 152 | ^{13}C NMR spectrum of **8qa**

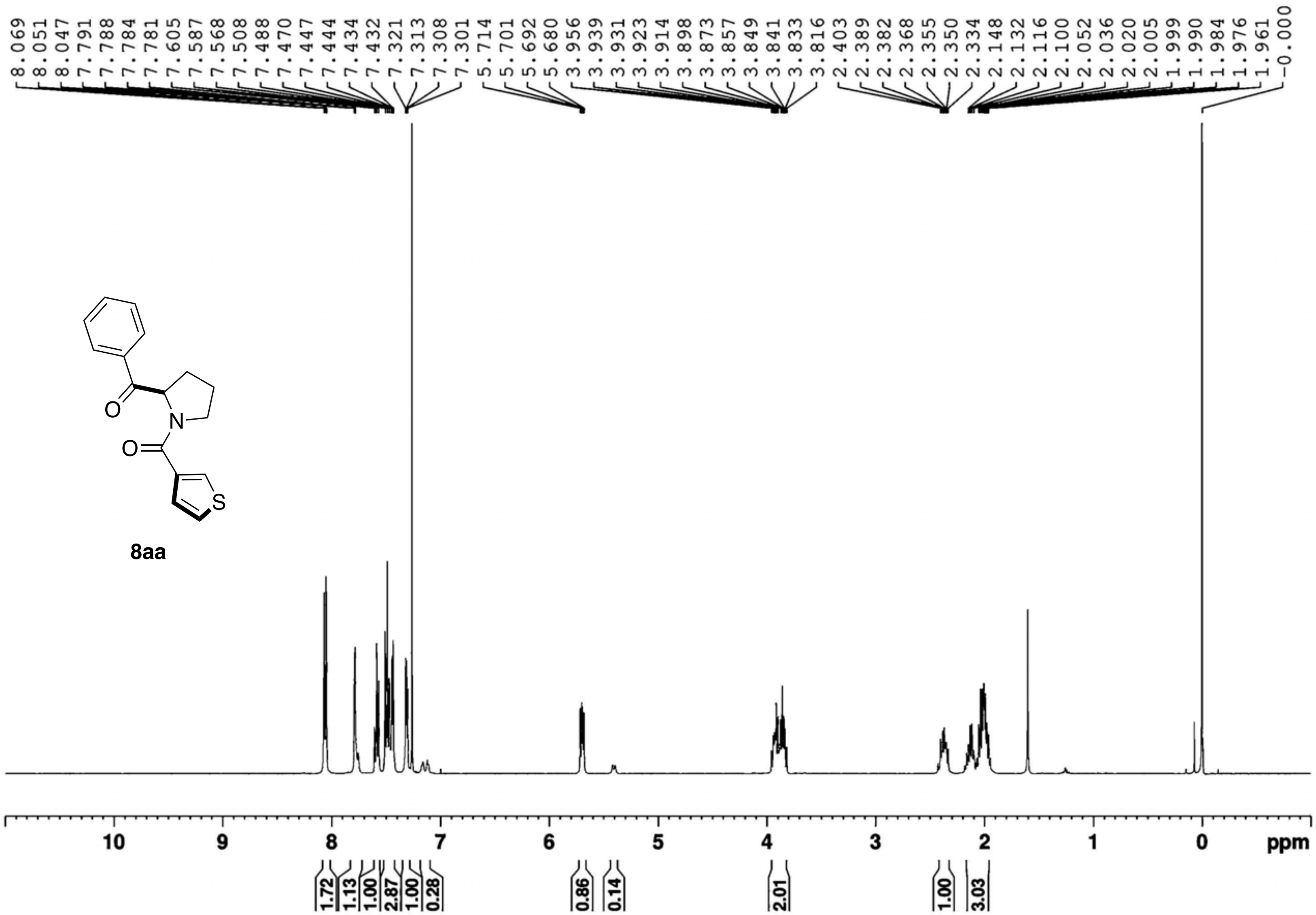

Supplementary Fig. 153 | ^{1}H NMR spectrum of **8aa**

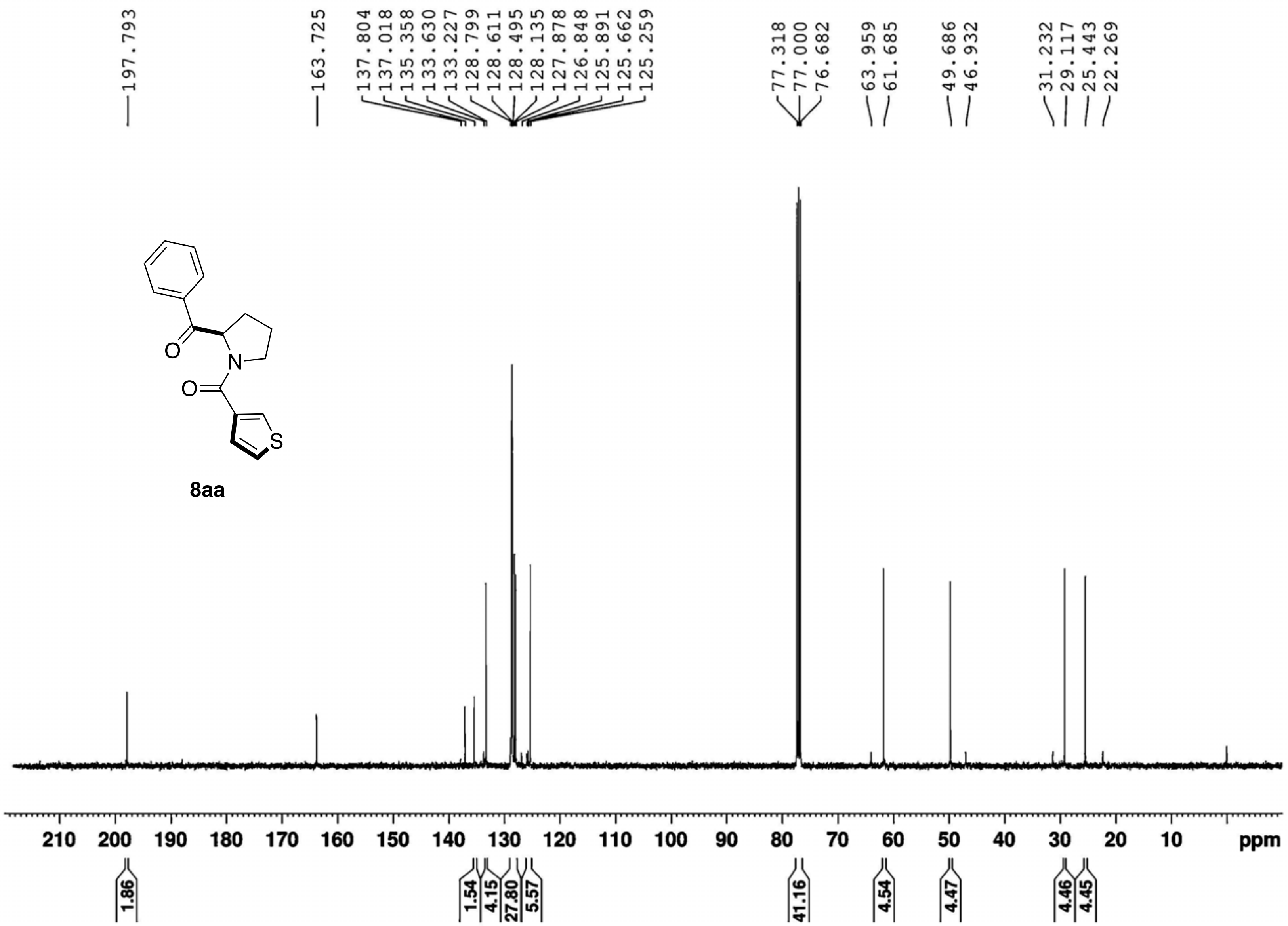

Supplementary Fig. 154 | ^{13}C NMR spectrum of **8aa**

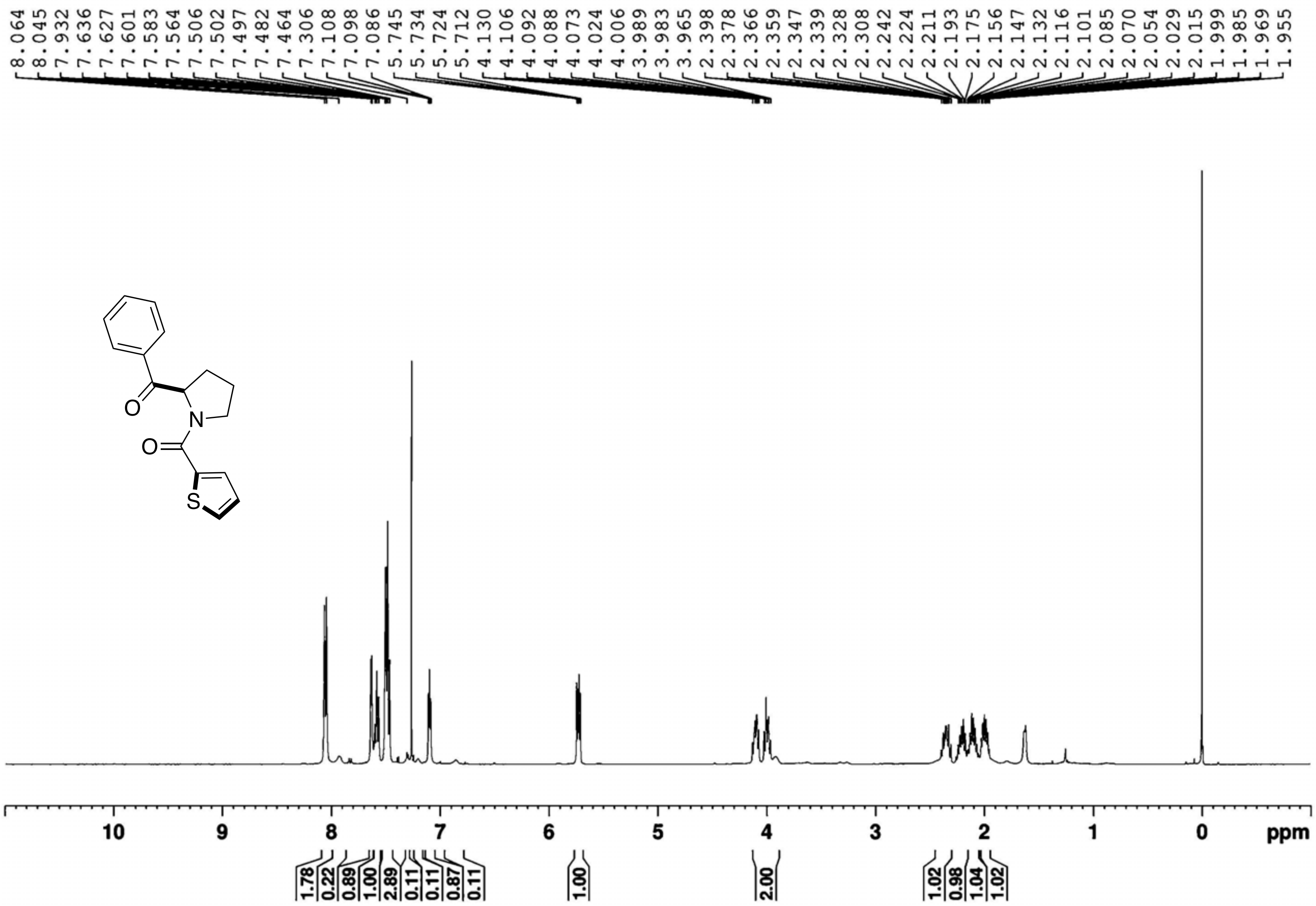

Supplementary Fig. 155 | ^{1}H NMR spectrum of **8aa-A**

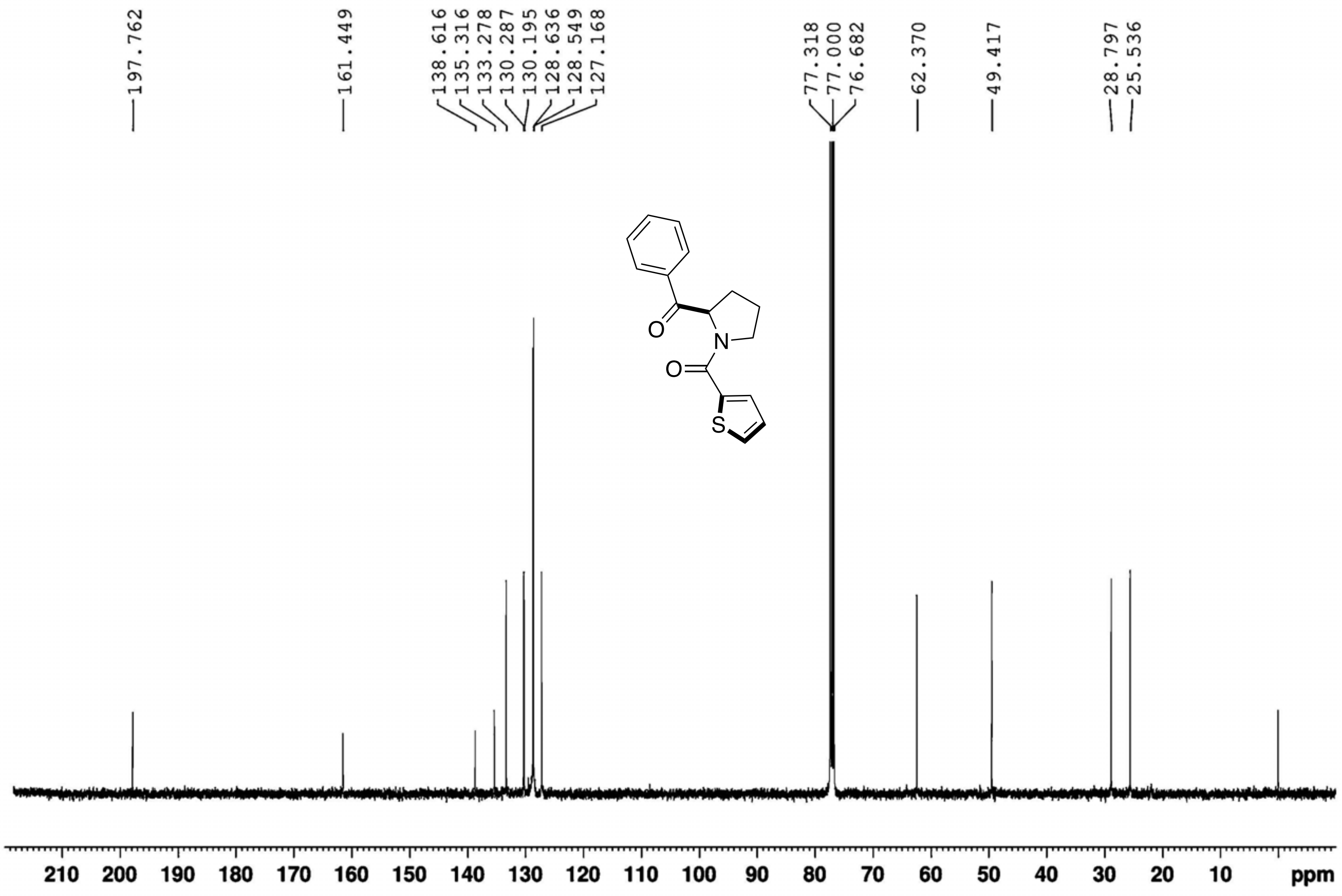

Supplementary Fig. 156 | ^{13}C NMR spectrum of **8aa-A**

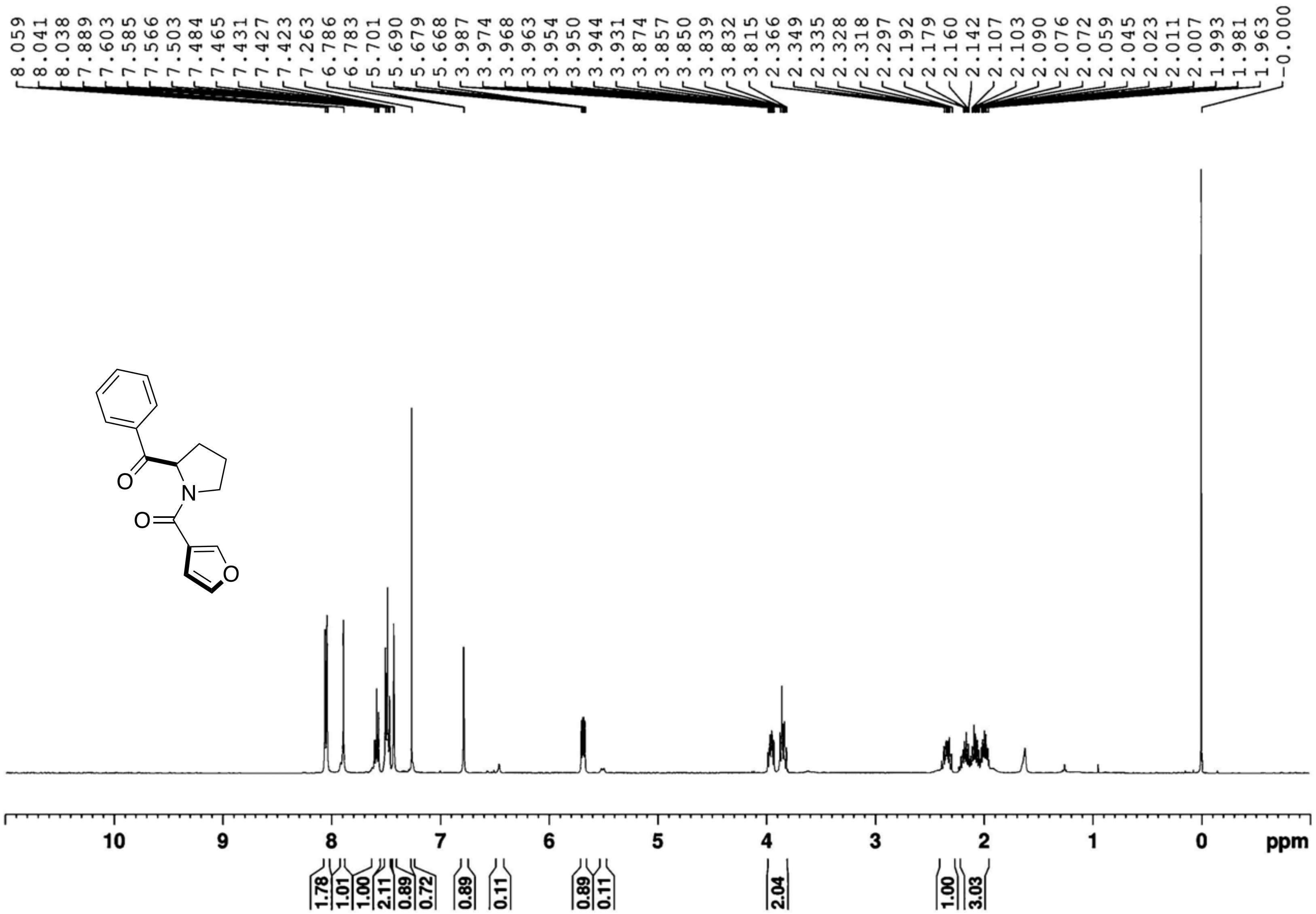

Supplementary Fig. 157 | ^{1}H NMR spectrum of **8aa-B**

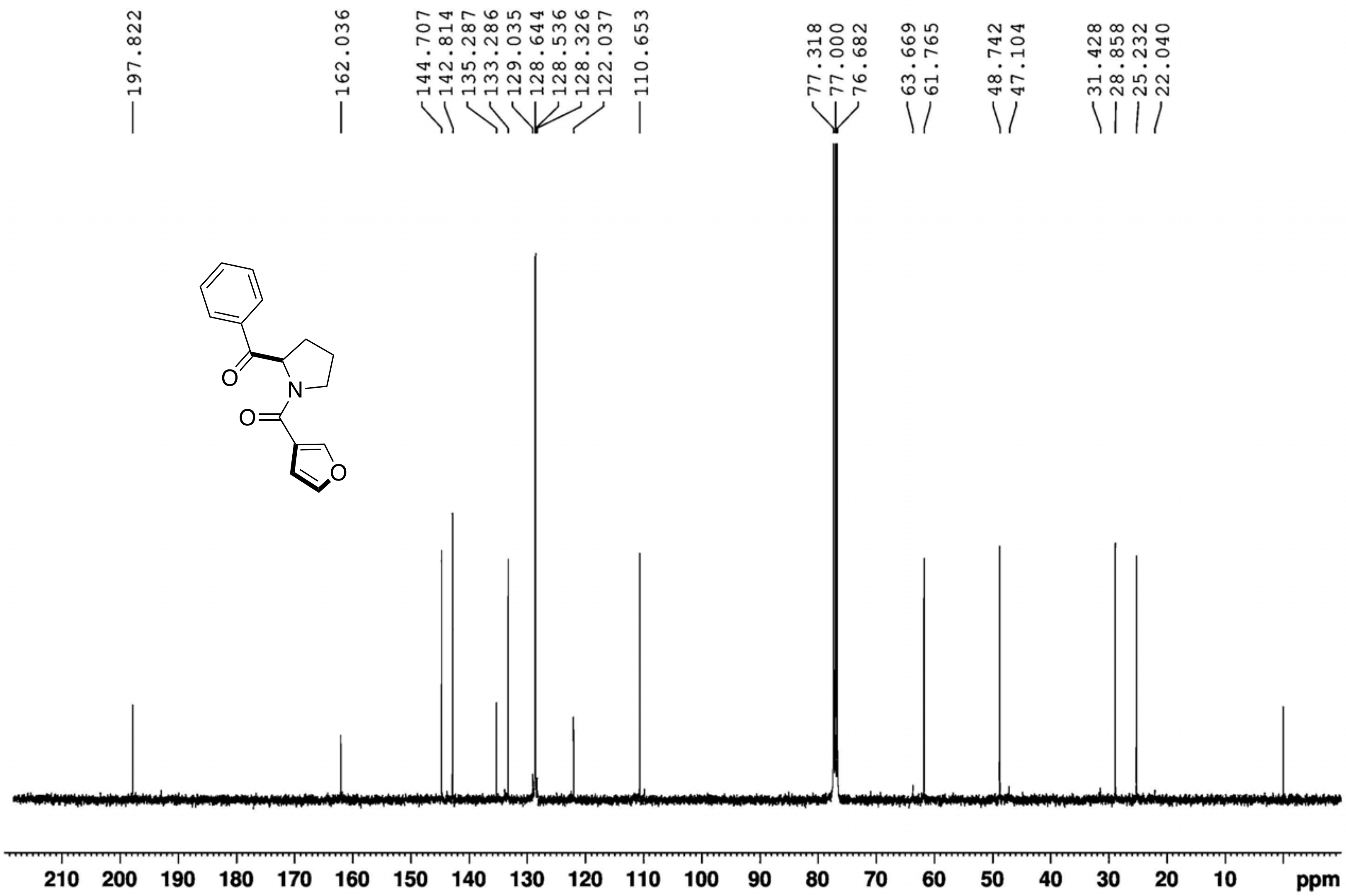

Supplementary Fig. 158 | ^{13}C NMR spectrum of **8aa-B**

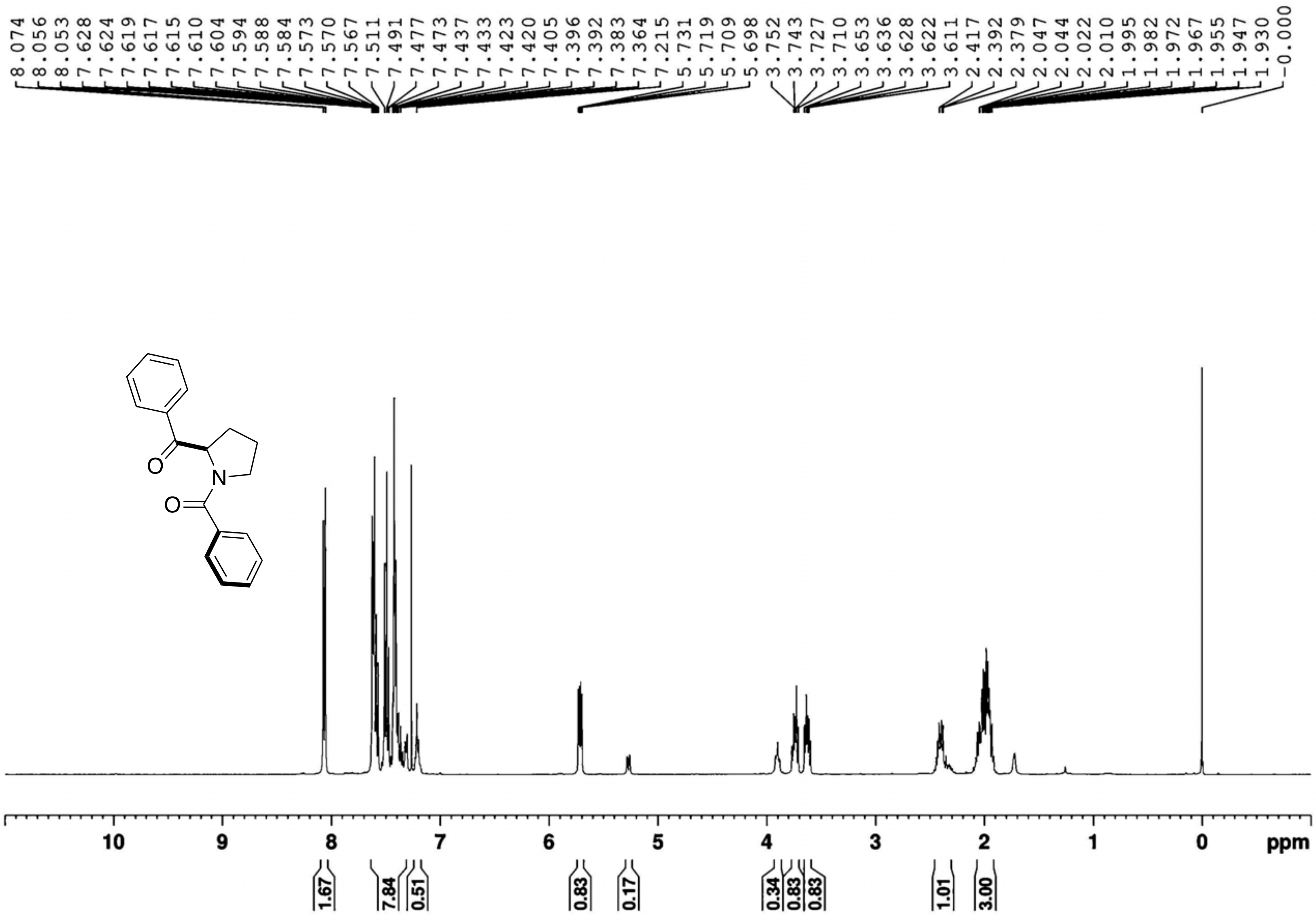

Supplementary Fig. 159 | ^{1}H NMR spectrum of **8aa-C**

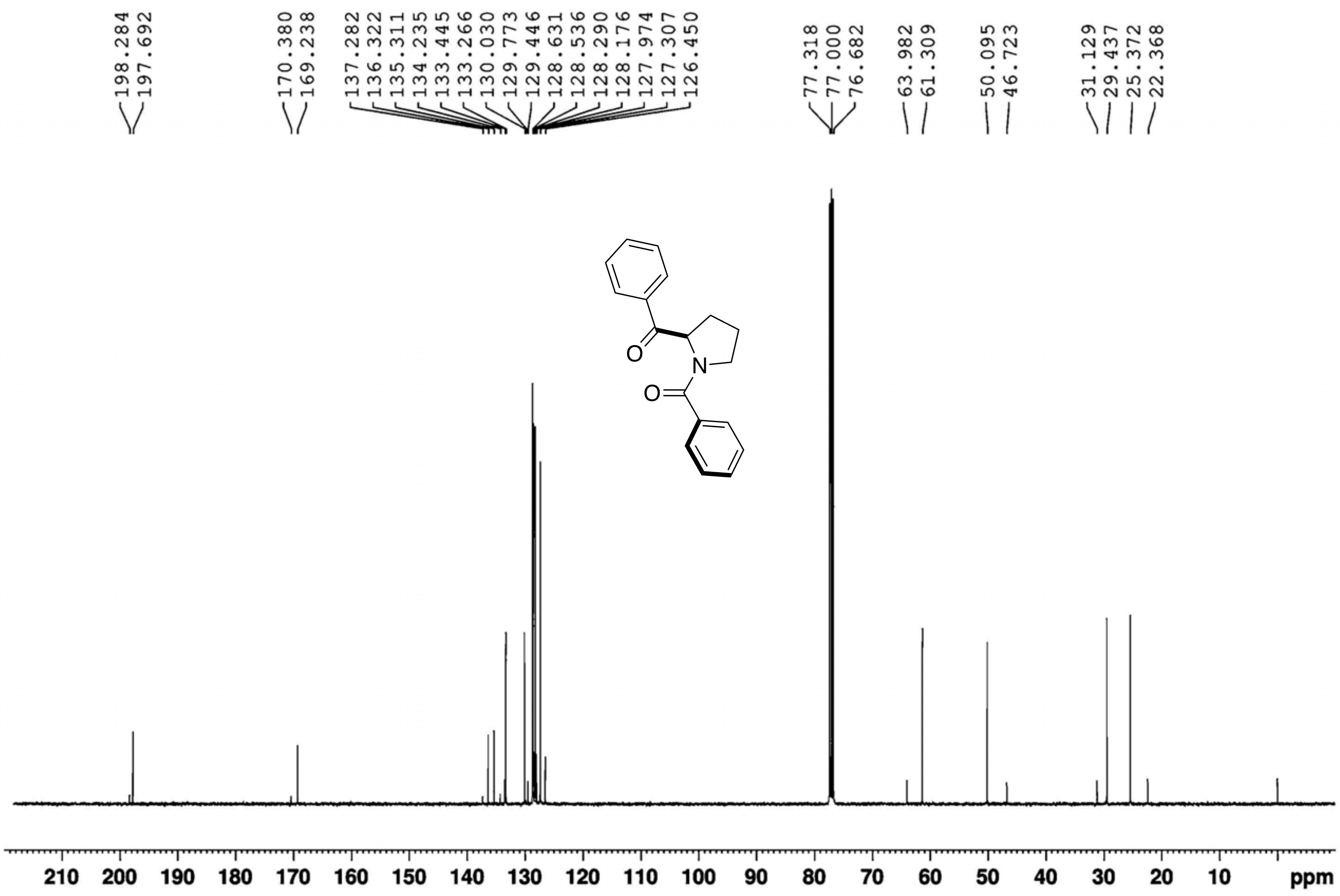

Supplementary Fig. 160 | ^{13}C NMR spectrum of **8aa-C**

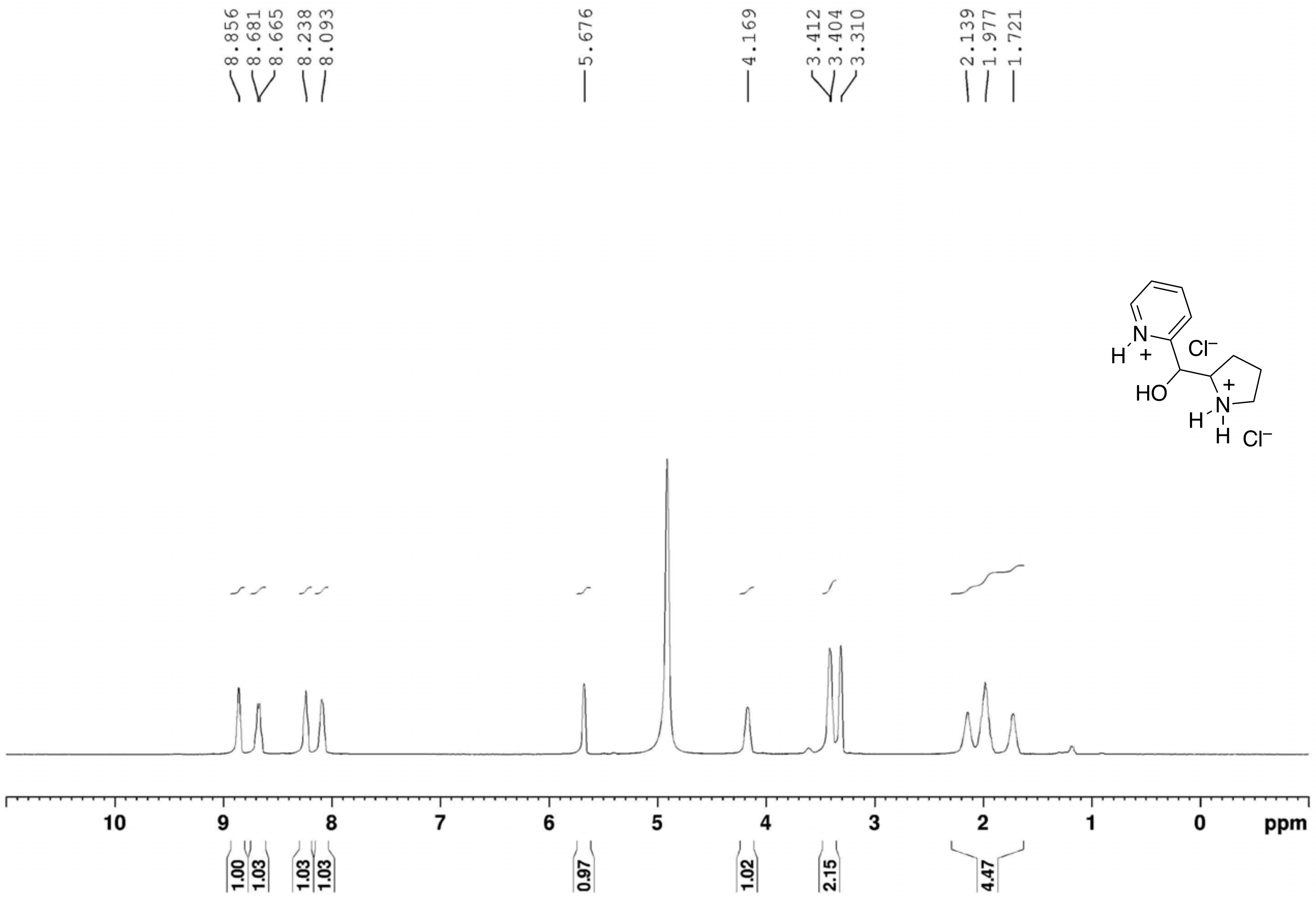

Supplementary Fig. 161 | ^{1}H NMR spectrum of **9ma**

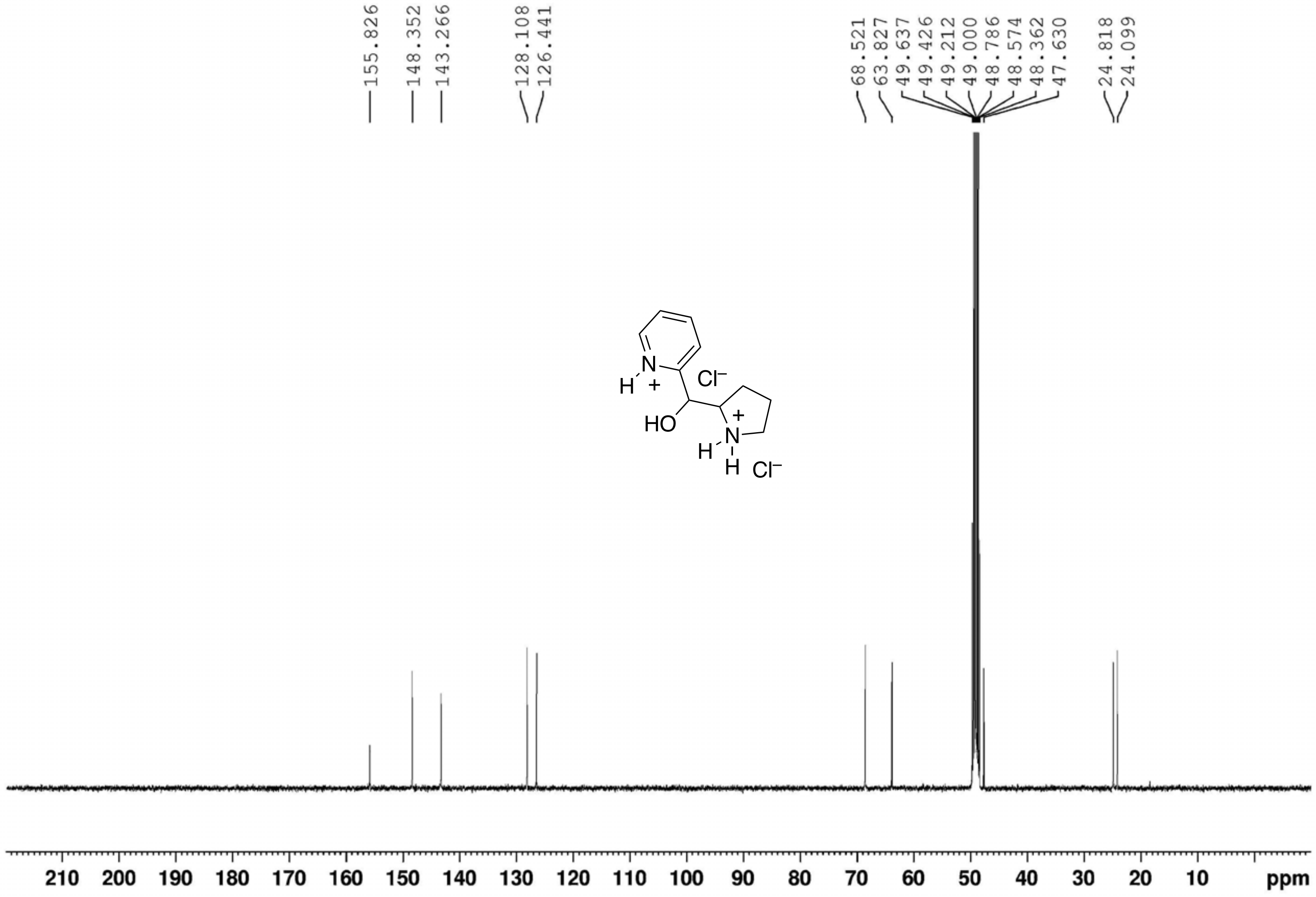

Supplementary Fig. 162 | ^{13}C NMR spectrum of **9ma**

■ Supplementary References ■

(1) Piel, I., Pawelczyk, M. D., Hirano, K., Fröhlich, R., Glorius, F. A Family of Thiazolium Salt Derived N-Heterocyclic Carbenes (NHCs) for Organocatalysis: Synthesis, Investigation and Application in Cross-Benzoin Condensation. *Eur. J. Org. Chem.* **2011**, 5475–5484 (2011).

(2) Rong, M.-G., Qin, T.-Z., Zi, W., Rhenium-Catalyzed Intramolecular Carboalkoxylation and Carboamination of Alkynes for the Synthesis of C3-Substituted Benzofurans and Indoles. *Org. Lett.* **21**, 5421–5425 (2019).

(3) Stark, T., Suhartono, M., Göbel, M. W., Lautens, M. A Palladium-Catalyzed Domino Reaction as Key Step for the Synthesis of Functionalized Aromatic Amino Acids. *Synlett* **24**, 2730–2734 (2013).

(4) Shao, M., Dongare, P., Dawe, L. N., Thompson, D. W., Zhao Y. Biscrown-Annulated TTFAQ−Dianthracene Hybrid: Synthesis, Structure, and Metal Ion Sensing. *Org. Lett.* **12**, 3050–3053 (2010).

(5) Feng, X., Li, L., Yu, X., Yamamoto, Y., Ming Bao, M.; Copper-catalyzed conversion of aryl and heteroaryl bromides into thecorresponding iodide. *Catal. Today.* **274**, 129–132 (2016).

(6) Górski, K., Mech-Piskorz, J., Leśniewska, B., Pietraszkiewicz, O., Pietraszkiewicz, M., Synthesis and Reactivity of 5-Heterotruxenes Containing Sulfur or Nitrogen as the Heteroatom. *J. Org. Chem.* **84**, 11553–11561 (2019).

(7) Hrobárik, P., Hrobáriková, V., Sigmundová, I., Zahradník, P., Fakis, M., Polyzos, I., Persephonis, P. Benzothiazoles with Tunable Electron-Withdrawing Strength and Reverse Polarity: A Route to Triphenylamine-Based Chromophores with Enhanced Two-Photon Absorption. *J. Org. Chem.* **76**, 8726–8736 (2011).

(8) Li, J., Chen, J., Sang, R., Ham, W.-S., Plutschack, M. B., Berger, F., Chabbra, S., Schnegg, A., Genicot, C., Ritter, T. Photoredox catalysis with aryl sulfonium salts enables site-selective late-stage fluorination. *Nat. Chem.* **12**, 56–62 (2020).

(9) Guilbault, A.-A., Legault, C. Y. Drastic Enhancement of Activity in Iodane-Based α-Tosyloxylation of Ketones: Iodine(III) Does the Hypervalent Twist. *ACS Catal.* **2**, 219–222 (2012).

(10) Hie, L., Nathel, N. F. F., Shah, T. K., Baker, E. L., Hong, X., Yang, Y.-F., Liu, P., Houk, K. N., Garg, N. K. Conversion of amides to esters by the nickel-catalysed activation of amide C−N bonds. *Nature* **524**, 79–83 (2015).

(11) Kabadwal, L. M., Das, J., Banerjee, D., Mn(II)-catalysed alkylation of methylene ketones with alcohols: direct access to functionalised branched products. *Chem. Commun.* **54**, 14069–14072 (2018).

(12) Gaussian 09, Revision D.01, M. J. Frisch, G. W. Trucks, H. B. Schlegel, G. E. Scuseria, M. A. Robb, J. R. Cheeseman, G. Scalmani, V. Barone, B. Mennucci, G. A. Petersson, H. Nakatsuji, M. Caricato, X. Li, H. P. Hratchian, A. F. Izmaylov, J. Bloino, G. Zheng, J. L. Sonnenberg, M. Hada, M. Ehara, K. Toyota, R. Fukuda, J. Hasegawa, M. Ishida, T. Nakajima, Y. Honda, O. Kitao, H. Nakai, T. Vreven, J. A. Montgomery, Jr., J. E. Peralta, F. Ogliaro, M. Bearpark, J. J. Heyd, E. Brothers, K. N. Kudin, V. N. Staroverov, T. Keith, R. Kobayashi, J. Normand, K. Raghavachari, A. Rendell, J. C. Burant, S. S. Iyengar, J. Tomasi, M. Cossi, N. Rega, J. M. Millam, M. Klene, J. E. Knox, J. B. Cross, V. Bakken, C. Adamo, J. Jaramillo, R. Gomperts, R. E. Stratmann, O.

Yazyev, A. J. Austin, R. Cammi, C. Pomelli, J. W. Ochterski, R. L. Martin, K. Morokuma, V. G. Zakrzewski, G. A. Voth, P. Salvador, J. J. Dannenberg, S. Dapprich, A. D. Daniels, O. Farkas, J. B. Foresman, J. V. Ortiz, J. Cioslowski, and D. J. Fox, Gaussian, Inc., Wallingford CT, 2013

(13) (a) Becke, A. D. A new mixing of Hartree–Fock and local density-functional theories. *J. Chem. Phys.* **98***,* 1372–1377 (1993). (b) Becke, A. D. Density-functional thermochemistry. III. The role of exact exchange. *J. Chem. Phys.* **98**, 5648–5652 (1993). (c) Lee, C., Yang, W., Parr, R. G. Development of the Colle-Salvetti correlation-energy formula into a functional of the electron density. *Phys. Rev. B.* **37**, 785–789 (1988). (d) Stephens, P. J., Devlin, F. J., Chabalowski, C. F., Frisch, M. J. Ab Initio Calculation of Vibrational Absorption and Circular Dichroism Spectra Using Density Functional Force Fields. *J. Phys. Chem.* **98**, 11623–11627 (1994).

(14) Hay, P. J., Wadt, W. R. Ab initio effective core potentials for molecular calculations. Potentials for the transition metal atoms Sc to Hg. *J. Chem. Phys.* **82**, 270–283 (1985).

(15) Spletstoser, J. T., White, J. M., Tunoori, A. R., Georg, G. I. Mild and Selective Hydrozirconation of Amides to Aldehydes Using Cp2Zr(H)Cl: Scope and Mechanistic Insight. *J. Am. Chem. Soc.* **129**, 3408−3419 (2007).

(16) Mattson, A. E., Zuhl, A. M., Reynolds, T. E., Scheidt, K. A. Direct Nucleophilic Acylation of Nitroalkenes Promoted by a Fluoride Anion/Thiourea Combination. *J. Am. Chem. Soc.* **128**, 4932–4933 (2006).

(17) Chiu, C. C., Jordan, F. Modeling an Elementary Step of the Enzyme Pyruvate Oxidase: Oxidation of a Thiamin Diphosphate-Bound Enamine Intermediate by a Flavin Analog. *J. Am. Chem. Soc.* **117**, 7027–7028 (1995).

(18) Nakanishi, I., Itoh, S., Suenobu. T., Fukuzumi, S. Electron transfer properties of active aldehydes derived from thiamin coenzyme analogues. *Chem. Commun.* 1927–1928 (1997).
